# Organoruthenium Glycomimetics Exhibit High Selectivity and Nanomolar Affinity for Human Galectin‑1

**DOI:** 10.1021/acs.jmedchem.5c03436

**Published:** 2026-02-05

**Authors:** Vojtěch Hamala, Martin Kurfiřt, Lucie Červenková Št́astná, Filip Dvořák, Jana Bernášková, Adéla Sýkorová, Jaroslav Kozák, Martin Zavřel, Tatiana Staroňová, Peter Šebest, Veronika Ostatná, Jakub Červený, Pavla Bojarová, Jitka Holčáková, Tomáš Hrstka, Roman Hrstka, Jindřich Karban

**Affiliations:** † Institute of Chemical Process Fundamentals of the Czech Academy of Sciences, Rozvojová 1/135, Praha 165 00, Czech Republic; ‡ Department of Organic Chemistry, University of Chemistry and Technology, Technická 5, Praha 166 28, Czech Republic; § Institute of Organic Chemistry and Biochemistry of the Czech Academy of Sciences, Flemingovo nám. 542, Praha 160 00, Czech Republic; ∥ Institute of Biophysics of the Czech Academy of Sciences, Královopolská 135, Brno 612 00, Czech Republic; ⊥ Institute of Microbiology of the Czech Academy of Sciences, Vídeňská 1083, Praha 142 00, Czech Republic; # Department of Analytical Chemistry, Faculty of Science, Charles University, Hlavova 8, Prague 128 43, Czech Republic; ∇ Research Centre for Applied Molecular Oncology, 48275 Masaryk Memorial Cancer Institute, Žlutý Kopec 7, Brno 656 53, Czech Republic

## Abstract

Human galectin-1 (*h*Gal-1) is an abundant β-galactoside-binding animal lectin that plays an essential role in promoting the immunosuppressive tumor microenvironment. Although *h*Gal-1 has been identified as a promising target for pharmacological inhibition, developing potent and selective *h*Gal-1 inhibitors has been complicated by the high degree of sequence similarity of the glycan-binding site across the galectin family. Herein, we present potent nanomolar *h*Gal-1 inhibitors with unprecedented selectivity of 2 to 3 orders of magnitude over human galectin-3 (*h*Gal-3). Their primary structural feature is the modification of a thiodigalactoside scaffold at the 3- and 3′-positions with a half-sandwich ruthenium­(II) arene complex containing a bidentate 4-(2-pyridyl)-1*H*-1,2,3-triazol-1-yl ligand. The most potent inhibitor in the series efficiently blocked the binding of *h*Gal-1 to the surface of MDA-MB-231 tumor cells, reduced their viability, and completely suppressed *h*Gal-1-induced phosphatidylserine exposure in Jurkat cells, a process previously described as preaparesis rather than classical apoptosis.

## Graphical abstract

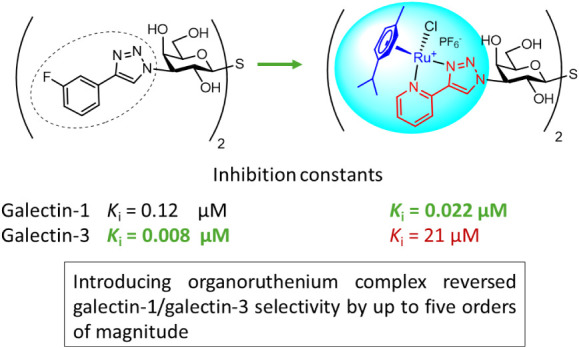

## Introduction

Lectins, a diverse group of glycan-binding proteins found ubiquitously in nature, act in vital processes in human biology, including participation in cell–pathogen interactions, modulation of the inflammatory response, autoimmune diseases, and cancer progression.^1^ Despite their significance in biomedicine, the development of small-molecule inhibitors of lectins has been lagging behind other classes of proteins,^2–5^ partially due to the challenges associated with targeting of their shallow water-exposed binding cavity^6^ and the difficulty in achieving selectivity among structurally homologous lectins.^2^ However, recent advances in glycochemistry, aided by X-ray crystallography and NMR spectroscopy, have facilitated the design and synthesis of potent lectin inhibitors,^7^ particularly those based on synthetically modified endogenous carbohydrate lectin ligands (glycomimetics).^7,8^

The challenges associated with lectin targeting also apply to galectins, a distinct family of lectins characterized by a high sequence homology and the ability to bind β-galactoside structural motifs in glycans.^9,10^ Galectins are implicated in pathologies including fibrotic diseases, inflammation, and cancer.^11–13^ In particular, human galectin-1 (*h*Gal-1) and galectin-3 (*h*Gal-3) have been the subject of extensive research due to their widespread tissue expression and biological impact.^10,14^ The bioactivity of galectins depends not only on their structure but also on their tissue and cellular localization.^9,15,16^ In addition, different galectins can exert opposing effects on disease progression,^17^ underscoring the need of selective inhibitors of individual galectins.

The design of small-molecule glycomimetic galectin inhibitors typically revolves around galactose-containing mono- and disaccharide glycomimetics modified with aromatic or heteroaromatic moieties to enhance interactions in the galectin binding site.^18^ Notably, the inhibitors based on thiodigalactoside (TDG, Figure 1A) and α-thiogalactoside (Figure 1B) scaffolds achieved submicromolar affinities. The selective *h*Gal-3 inhibitors **GB0139** (also known as TD139)^19^ and **GB1211** ^20^ (Figure 1) have advanced into clinical trials for the treatment of idiopathic pulmonary fibrosis (**GB0139**),^21^ selected carcinomas (**GB1211**), and hepatic impairment (**GB1211**).^22^ Despite significant progress in the development of *h*Gal-3 inhibitors, achieving high potency and selectivity for *h*Gal-1 remains a formidable challenge, notwithstanding the therapeutic promise of *h*Gal-1 inhibition.^23–33^ The recently reported 3-thienyl and 2-thiazolyl thiodigalactoside analogs **1** and **2** (Figure 1A)^19,34^ and 1,3-disubstituted α-thiogalactoside **4** (Figure 1B)^35^ demonstrated a nanomolar affinity and up to an over 100-fold selectivity for *h*Gal-1 over *h*Gal-3.

**Figure 1:**
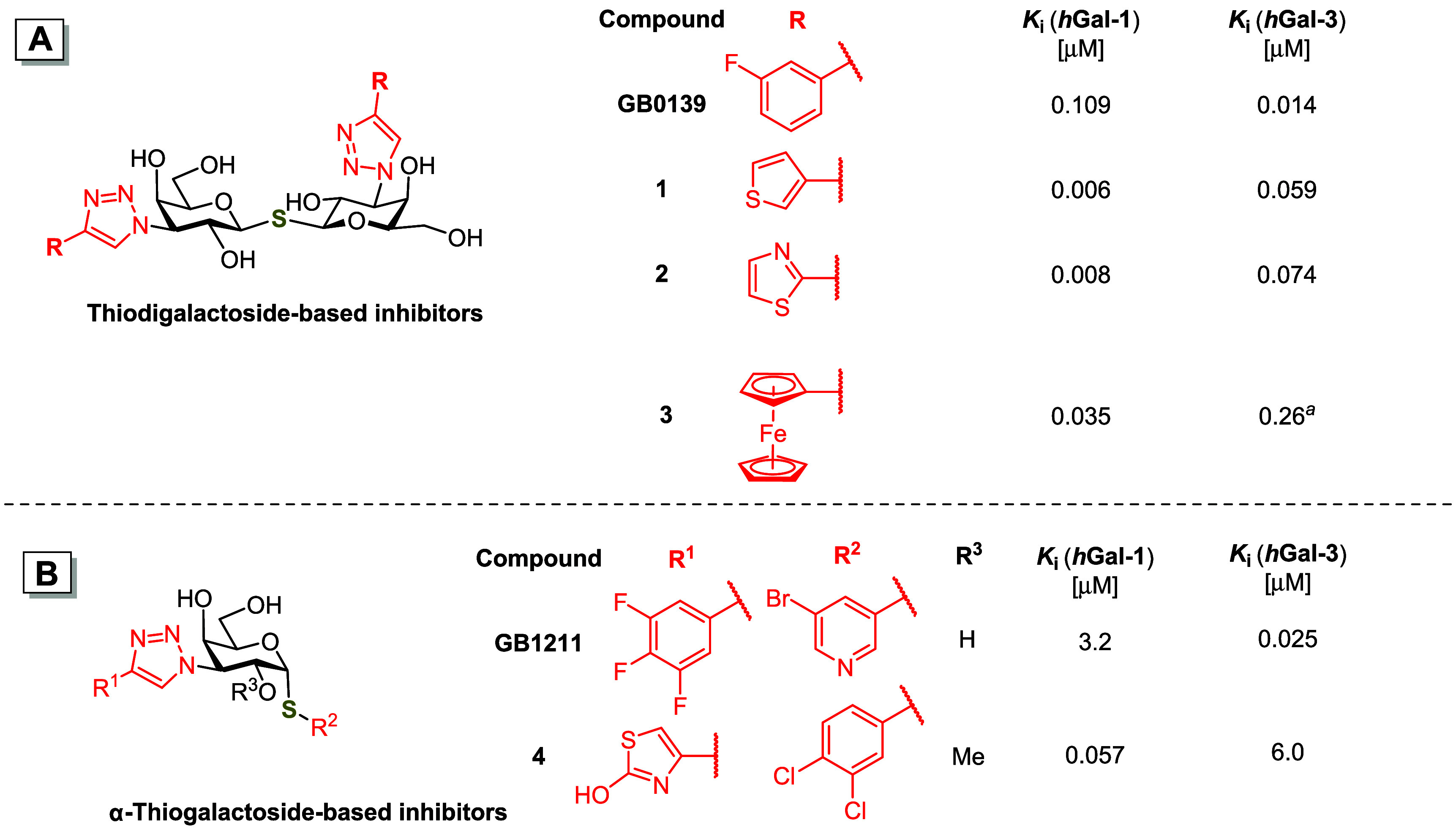
Structures and inhibitory activities of previously reported (**A**) thiodigalactoside-based galectin inhibitors **GB0139**,^19^ **1** ^34,19^ **2**,^34^ and **3**;^36^ and (**B**) α-thiogalactoside-based inhibitors **GB1211** ^20^ and **4** ^35^ determined by the fluorescence polarization assay (FP) and expressed as inhibition constant *K*_i_; ^a^ *h*Gal-3 carbohydrate recognition domain (CRD) instead of the full-length *h*Gal-3 was used to determine *K*_i_.

To describe and model the interactions of galectins with inhibitors such as **GB0139**, the binding groove of galectins can be divided into five binding subsites A–E.^10,37,38^ The key interactions between the conserved subsites C and D and the endogenous disaccharide ligands lactose (Galβ1–4Glc, Lac) and *N*-acetyllactosamine (Galβ1–4GlcNAc, LacNAc, Figure 2A) are reproduced in the binding of the thiodigalactoside scaffold of **GB0139** to these subsites, whereas the 3-fluorophenyl triazole moieties in **GB0139** provide additional interactions with the flanking subsites A, B, and E (Figure 2), collectively resulting in the high affinities of **GB0139** and of the related inhibitors to *h*Gal-3. Assuming that the planarity of the decorating phenyl moieties is not critical for the high-affinity interactions of **GB0139** with subsites A, B, and E, we propose that the aryl moieties in **GB0139** and related inhibitors could be replaced with three-dimensional arene mimetics^39^ including organotransition metal complexes.^40,41^ We hypothesized that the three-dimensional nature of organometallic complexes could more efficiently occupy galectin subsites A, B, and E, introducing additional interactions inaccessible to the planar 3-fluorophenyl in **GB0139**.^42^ In addition, the sterically demanding organotransition metal complexes may better discriminate between the binding subsites of homologous galectins than the more easily accommodated fluorinated phenyl ring.

**Figure 2:**
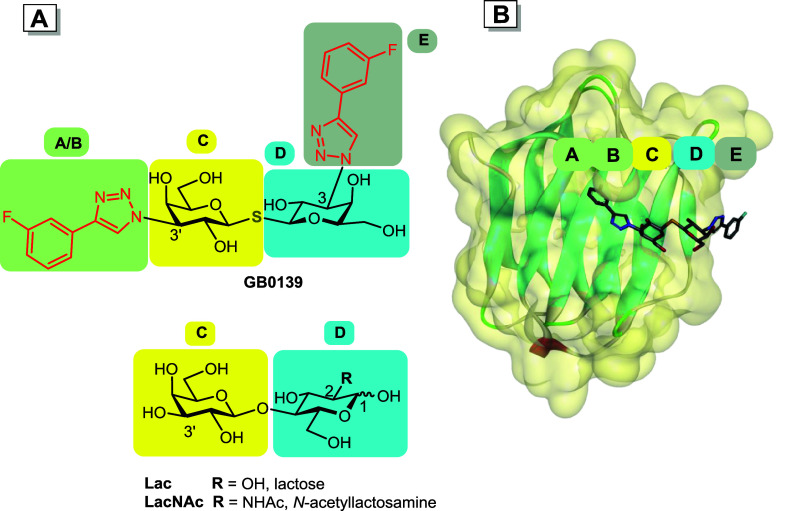
(**A**) Inhibitor **GB0139** and endogenous ligands Lac and LacNAc divided into segments interacting with the respective galectin subsites A–E, with the 3-fluorophenyl-triazole moiety in red; the numbers indicate positions on the pyranose ring; (**B**) Subsites A–E shown for the complex of the carbohydrate recognition domain (CRD) of *h*Gal-3 with **GB0139** (PDB ID: 5H9P).

We synthesized and tested sandwich complexes of ruthenium and iron and found that the diferrocenyl analog **3** displayed an approximately 3-fold higher affinity to *h*Gal-1 than **GB0139**, and a 7-fold selectivity for *h*Gal-1 over *h*Gal-3-CRD (Figure 1A).^36^ These results confirmed the validity of our concept of designing galectin inhibitors, but the affinity and selectivity gains were moderate. Herein, we report that the incorporation of a half-sandwich (piano stool) ruthenium­(II) arene complexes containing bidentate 4-(2-pyridyl)-1*H*-1,2,3-triazol-1-yl moiety (Figure 3) into the LacNAc or thiodigalactoside scaffolds tremendously improved the selectivity for *h*Gal-1 while not compromising *h*Gal-1 binding affinity. This concept has led to the discovery of potent small-molecule *h*Gal-1 inhibitors with unprecedented selectivity for *h*Gal-1 over *h*Gal-3 that, in addition, could protect T cells against *h*Gal-1-induced preaparesis and prevent *h*Gal-1 from binding to the surface of cancer cells.

**Figure 3:**
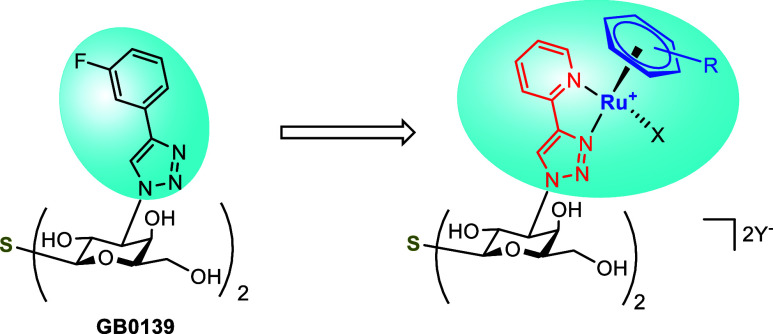
Replacement of the 3-fluorophenyl-triazole moiety in thiodigalactoside **GB0139** by a half-sandwich ruthenium­(II) complex. The 4-(3-fluorophenyl)-triazole and organoruthenium moieties are highlighted, the 4-(2-pyridyl)-1*H*-1,2,3-triazol-1-yl moiety in red, the π-coordinated arene in blue, R = alkyl(s), X = halogen, Y = counteranion.

## Results

### Synthesis of *h*Gal-1 Inhibitors

The ruthenium half-sandwich complex was attached to the 1- and 2-positions of disaccharide Lac, the 3′-position of disaccharide LacNAc, and the 3- and 3′-positions of the thiodigalactoside (TDG) scaffold. These positions were selected because their modifications do not interfere with galectin binding.^18,43–48^ First, the 4-(2-pyridyl)-1*H*-1,2,3-triazole moiety was easily introduced by copper-catalyzed cycloaddition of an acetylated monoazido- or diazido-disaccharide of general structure **7** with 2-ethynylpyridine (Scheme 1). Base-catalyzed Zemplén deacetylation yielded a disaccharide of general structure **8** that was subjected to complexation with a dihalogenido­(arene)­ruthenium­(II) dimer [Ru­(A)­X_2_]_2_ (A = arene, X = halogen) to afford target organoruthenium-modified disaccharide **9** with a chloride or iodide counteranion. A counteranion exchange by the reaction of chloride **9** with AgPF_6_ or AgBF_4_ afforded the corresponding hexafluorophosphate or tetrafluoroborate salts **10** (see Supporting Information for synthetic details). The starting peracetylated azido-disaccharides (Scheme 1B) were synthesized as previously described,^36,49–52^ except for the acetylated methyl 2,3′-diazido-β-lactoside **2,3′-diN**_**3**_ **-Lacβ-OMe** prepared by chemical glycosylation (see the Supporting Information for details).

**Scheme 1:**
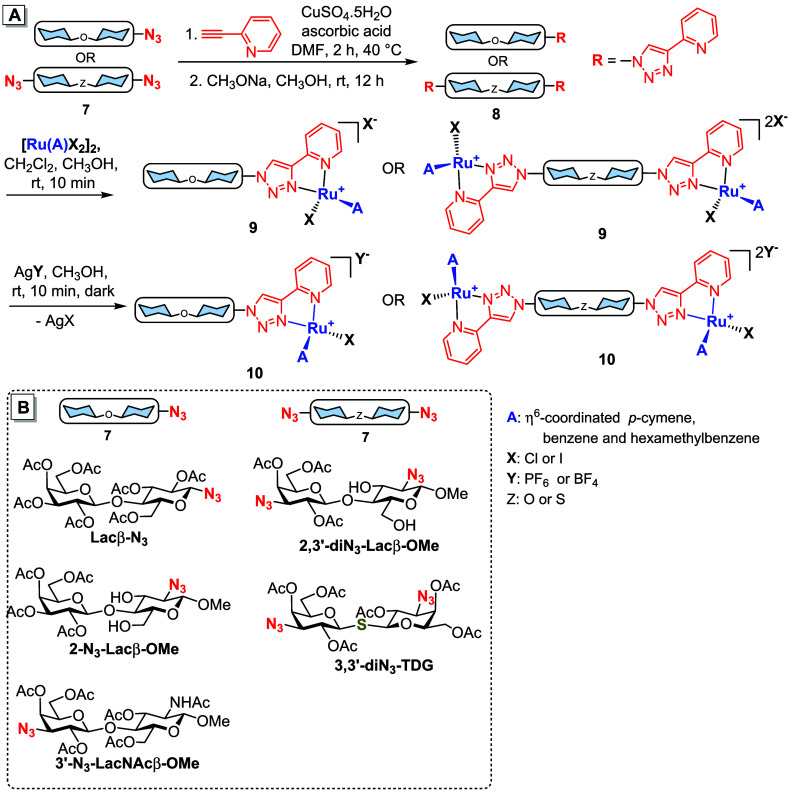
(**A**) General Synthesis of Ruthenium Piano-Stool Glycomimetics (Only One Diastereomer at the Ru Center Is Shown for Clarity); (**B**) Structures of the Starting Azido-Disaccharides

The target ruthenium complexes **11**–**13** (Figure 4) were obtained as a mixture of two diastereomers due to the introduction of a new stereogenic center at the ruthenium atom. Bisruthenium complex **14** was obtained as a mixture of four diastereomers because two new stereogenic centers were introduced to the molecule. Only three diastereomers were formed in the case of bisruthenium complexes **15**–**18**, because the *R,S* and *S,R* combinations at the two ruthenium centers were identical due to the *C*_2_ axis of symmetry. These diastereomers could be detected by ^1^H and ^13^C NMR spectroscopy. LacNAc-, Lac-, and TDG-based precursors **19**–**21** with one or two 4-(2-pyridyl)-1*H*-1,2,3-triazole moieties uncoordinated to ruthenium were used as reference compounds in affinity evaluation, along with methyl *N*-acetyl-β-lactosaminide **LacNAc**β**-OMe** ^53^ (Figure 4) and lactose.

**Figure 4:**
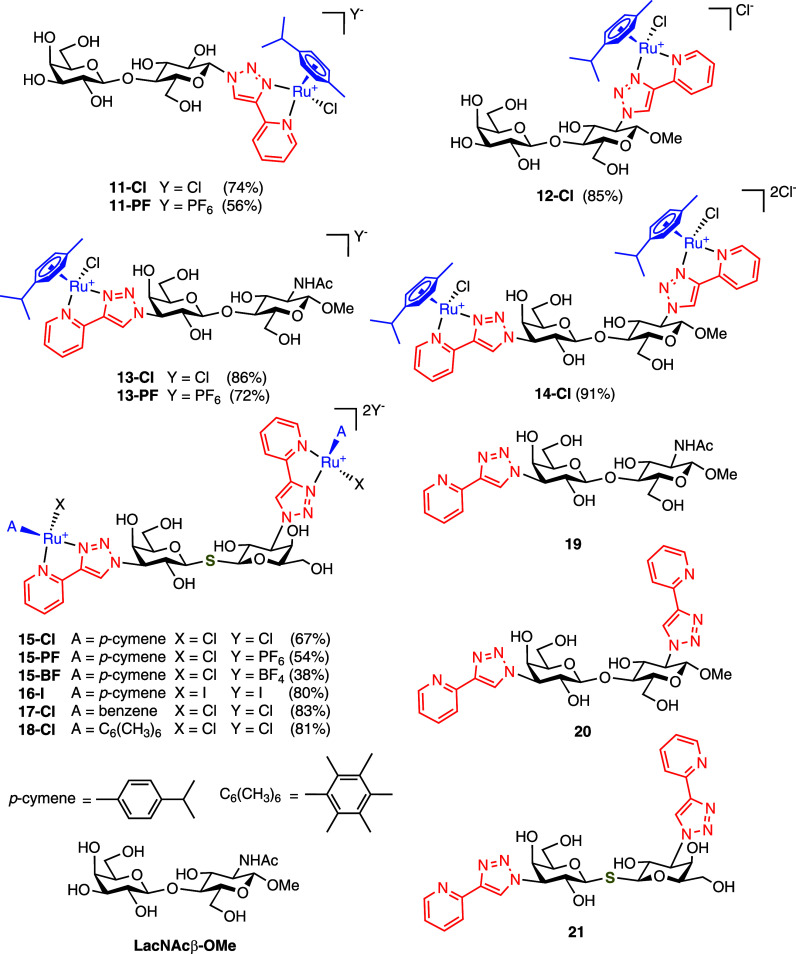
Structures of ruthenium glycomimetics (only one diastereomer at the Ru center is shown for clarity), and reference compounds **19**–**21** and **LacNAc**β**-OMe**. The values in parentheses are the yields of the complexation reaction (including counterion exchange, where applicable).

The organoruthenium complexes were stable in air and well soluble in water, methanol, or DMSO. The most potent inhibitor **15-PF** (*vide infra*) was stable in an aqueous solution containing chloride Cl^–^ ions at standard saline concentrations (*c*_NaCl_ = 154 mM), including phosphate-buffered saline (PBS buffer) and human blood serum (see the Supporting Information for spectra). The complexes generally undergo an exchange reaction of the halide ligand in aqueous solutions^54,55^ that were studied in detail for compound **13-PF** (Scheme 2 and the Supporting Information, Figure S1). Complex **13-PF** undergoes partial aquation-dechlorination in an aqueous solution leading to a dynamic equilibrium between chloro- and aqua-ruthenium species **13-PF-Cl** and **13-PF-OH**_**2**_^**+**^. Under alkaline conditions, the hydroxy-ruthenium species **13-PF-OH** ^–^ predominates (see the Supporting Information, Section B for details). The irreversible substitution of the weakly bound halide ligand with a nucleophilic group from biomolecules can compromise the galectin binding affinity. However, our ^1^H NMR monitoring of complex **15-PF** in blood serum suggests that stable coordination bonds with serum proteins do not form to a significant extent.

**Scheme 2:**
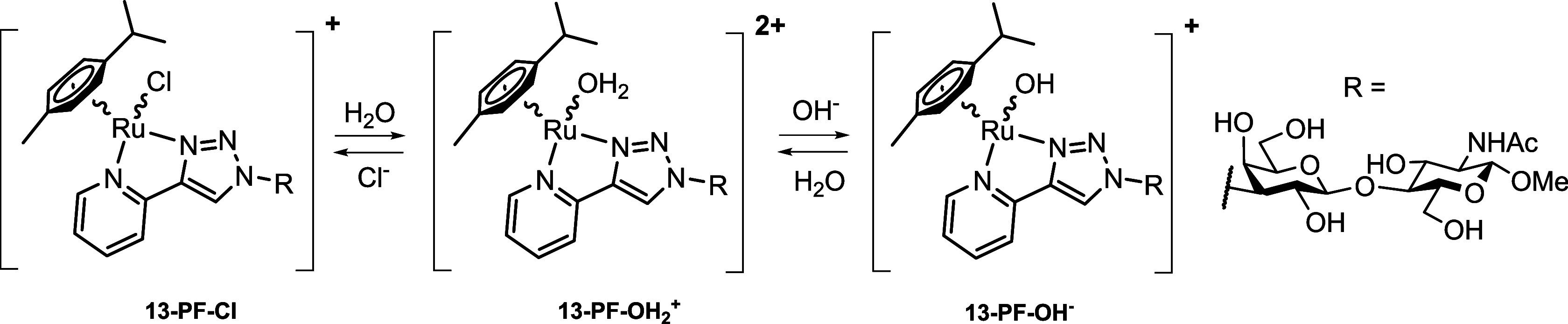
Equilibrium between Chloro-, Aqua-, and Hydroxy-Ruthenium Species

### Affinity to Galectins

#### Screening of All Galectin Inhibitors by Competitive Fluorescence Polarization Assay

The affinities of ruthenium glycomimetic inhibitors to *h*Gal-1 and to the carbohydrate recognition domain of *h*Gal-3 (denoted here as *h*Gal-3-CRD) were first determined using a competitive fluorescence polarization (FP) assay, in which a fluorescently labeled high-affinity probe competes with the inhibitor for the galectin binding site.^56^ This assay allows for rapid affinity screening with minimal consumption of the inhibitor and galectin. The inhibition constant *K*_i_ was calculated and used to estimate the inhibitory activity of compounds (see the Supporting Information, Section D, for details).

The introduction of an organoruthenium moiety at the 1- and 2-positions of Lac-derived compounds (**11-Cl, 11-PF**, **12-Cl**) enhanced the binding affinity to *h*Gal-1 considerably more (up to 20-fold) than to *h*Gal-3-CRD (up to 4.5-fold) compared with the parent unmodified disaccharides, but the found affinities still remained in the micromolar range (Supporting Information, Table S2). In contrast, the attachment of the organoruthenium complex to the 3′-position of LacNAc significantly improved the binding to *h*Gal-1 and selectivity over *h*Gal-3-CRD (Table 1). Thus, complexes **13-Cl** and **13-PF** bound *h*Gal-1 approximately 200- and 240-fold more strongly, respectively, than parent LacNAcβ-OMe, while their affinity to *h*Gal-3-CRD was reduced approximately 5-fold, resulting in a selectivity for *h*Gal-1 of 2 orders of magnitude. Both **13-Cl** (*K*_i_ = 0.9 μM) and **13-PF** (*K*i = 0.75 μM) by far outperformed the corresponding uncoordinated compound **19**, previously reported ferrocenyl-trizole complex **22**, and 3-fluorophenyl-triazole complex **23** in terms of *h*Gal-1 selectivity.^36^ Counterion exchange (Cl^–^ → PF_6_^–^) had only a marginal effect on galectin affinity (*cf*. **13-PF** vs **13-Cl**, Table 1). One additional organoruthenium complex attached to the 2-position of **13-Cl** to give 2,3′-bisruthenium complex **14-Cl** further increased the binding affinity to both galectins approximately 4-fold, reaching an astounding 200 nM *K*_i_ for *h*Gal-1 and a 290-fold selectivity over *h*Gal-3. These excellent selectivities are clearly due to the presence of the organoruthenium complex, since **20**, an immediate precursor of **14-Cl** without the ruthenium complex, was a submicromolar binder of both galectins, moderately selective for *h*Gal-3. The present results demonstrate a noteworthy observation that the attachment of the organoruthenium complex to the 3′-position of LacNAc is by far more beneficial for binding affinity and selectivity for *h*Gal-1 compared to the 1- and 2-positions of the closely related Lac scaffold.

**Table 1:**
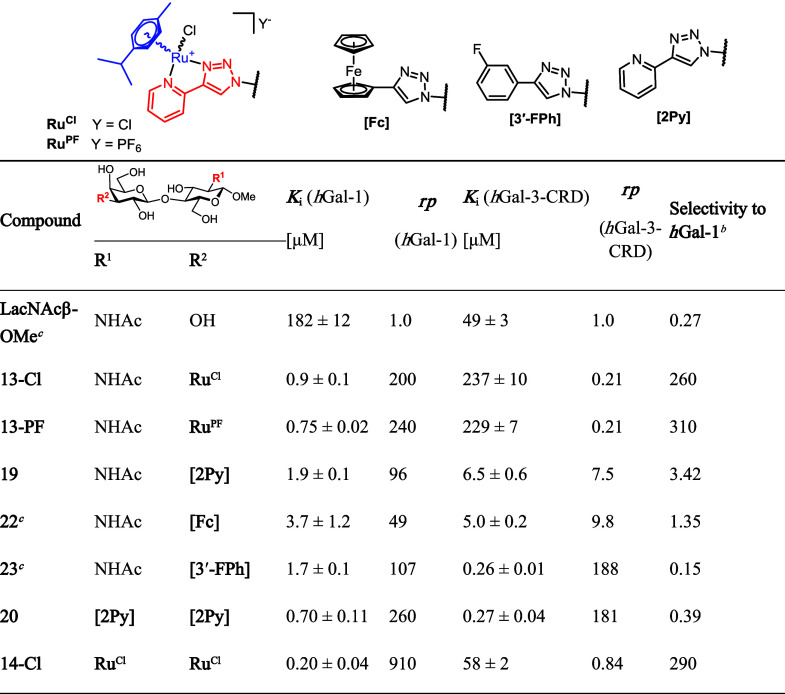
Inhibition Constant *K*_i_, Relative Potency *rp* ^a^, and Selectivity of Inhibitors Derived from LacNAc Determined by FP

a Relative potency wasdefined as *rp* = *K*_i_(LacNAcβ-OMe)/*K*_i_(inhibitor) for LacNAc-based inhibitors.

b Defined as the ratio *K*_i_(*h*Gal-3-CRD)/*K*_i_(*h*Gal-1).

c The values for **LacNAcβ-OMe**, **22**, and **23** adopted from ref. 36.

Even much more striking affinity results were achieved in the series of organoruthenium-modified thiodigalactosides (Table 2). The replacement of the 3- and 3′-fluorophenyl moieties in the state-of-the-art inhibitor **GB0139** with 2-pyridyl to give precursor **21** significantly reduced the affinity to *h*Gal-3-CRD, but only slightly to *h*Gal-1. The decrease in *h*Gal-3-CRD affinity is most likely due to the elimination of the orthogonal multipolar fluorine interactions with the polypeptide backbone of *h*Gal-3-CRD.^57^ The complexation of **21** with ruthenium to give the piano-stool complex **15-Cl** afforded a similar affinity to *h*Gal-1 as **GB0139**, but a drastically reduced affinity to *h*Gal-3-CRD by 3 orders of magnitude, producing a highly selective *h*Gal-1 inhibitor (*K*_i_ = 110 nM, 270-fold selectivity over *h*Gal-3). Obviously, the ruthenium half-sandwich complex strongly discriminates *h*Gal-3-CRD but favors *h*Gal-1 (cf. **15-Cl** vs **21**).

**Table 2:**
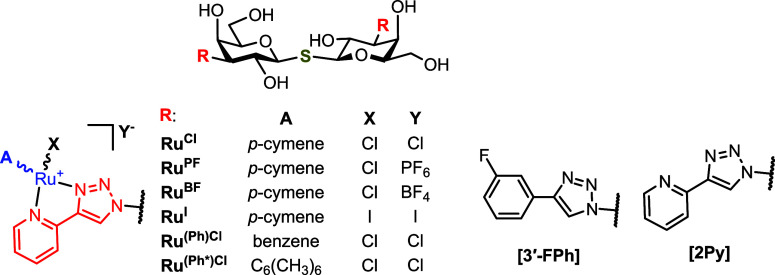
Inhibition Constant *K*_i_, Relative Potency *rp* ^a^, and Selectivity of Thiodigalactoside Analogs Determined by FP

Compound	R	*K*_i_(*h*Gal-1) [μM]	*rp* (*h*Gal-1)	*K*_i_(*h*Gal-3-CRD) [μM]	*rp* (*h*Gal-3-CRD)	Selectivity to *h*Gal-1^b^
**GB0139** ^c^	[3′-FPh]	0.12 ± 0.01	1.0	0.008 ± 0.001^d^	1.0	0.07
**21**	**[2Py]**	0.16 ± 0.05	0.7	0.25 ± 0.02	0.032	1.6
**15-Cl**	**Ru** ^**Cl**^	0.11 ± 0.01	1.1	30 ± 2	2.7 × 10^–4^	270
**15-PF**	**Ru** ^**PF**^	**0.022 ± 0.012**	**5.5**	**21 ± 2**	**3.8 × 10** ^**–4**^	**950**
**15-BF**	**Ru** ^**BF**^	0.070 ± 0.025	1.7	14 ± 1	5.7 × 10^–4^	200
**16-I**	**Ru** ^**I**^	0.019 ± 0.011	6.3	17 ± 1	4.7 × 10^–4^	890
**17-Cl**	**Ru** ^**(Ph)Cl**^	0.028 ± 0.007	4.3	2.2 ± 0.3	3.6 × 10^–3^	79
18-Cl	**Ru** ^(Ph*)Cl^	0.23 ± 0.06	0.5	31 ± 4	2.6 × 10^–4^	130

a Relative potency with respect to **GB0139**, defined as *rp* = *K*_i_(**GB0139**)/*K*_i_(inhibitor).

b Defined as the ratio *K*_i_(*h*Gal-3-CRD)/*K*_i_(*h*Gal-1).

c Reported in ref. 36.

d *K*_i_ = 0.014 ± 0.003 μM was reported for binding to full-length *h*Gal-3 instead of *h*Gal-3-CRD in ref. 19.

Introduction of iodide as a ligand and counteranion instead of chloride (compounds **16**-**I**), or counteranion exchange of chloride for hexafluorophosphate (**15-PF**), produced approximately a 5-fold increase in the affinity to *h*Gal-1, resulting in an exceptional *h*Gal-1 selectivity of 950-fold for **15-PF** over *h*Gal-3. Introducing the tetrafluoroborate counteranion (**15-BF**) produced affinity and selectivity similar to those of chloride **15-Cl**. Replacing the *p*-cymene ligand in **15-Cl** with the sterically less demanding benzene in **17-Cl** diminished the selectivity by improving the affinity to *h*Gal-3-CRD more than to *h*Gal-1. The same overall effect was reached in **18-Cl** where the bulky hexamethylbenzene substantially reduced binding to *h*Gal-1 in contrast to that of *h*Gal-3-CRD. This may suggest that balancing the steric clashes with the binding site plays an important role in modulating the inhibitory potency. In summary, the substitution of the fluorophenyl moiety in **GB0139** with the half-sandwich organoruthenium complexes resulted in potent *h*Gal-1 inhibitors with an outstanding selectivity over *h*Gal-3. The highest selectivity in the series was obtained for compound **15-PF**, reaching a *K*_i_ value of 22 nM with *h*Gal-1. This compound was much more selective than the recently reported small-molecule *h*Gal-1 inhibitors **1**–**4** (Figure 1),^19,34–36^ making it the most selective and one of the most potent *h*Gal-1 inhibitors reported to date.

#### Assessment of the Affinity of the Best Inhibitors by Intrinsic Tryptophan Fluorescence Assay

In parallel, we determined the binding affinity of the potent *h*Gal-1 inhibitors **15-PF**, **15-BF**, and **17-Cl** and the reference compounds **GB0139**, **1**, and **3** by a complementary method based on ligand-induced changes in the intrinsic fluorescence of tryptophan residues (ITF).^58^ We expressed the affinity as the dissociation constant *K*_D_ (Table 3). The *K*_D_ values obtained by ITF generally indicated stronger *h*Gal-1 binding than the *K*_i_ values from the FP assay. This discrepancy may be due to the nature of the assays themselves, as the ITF assay is a direct binding assay, unlike the competitive FP assay. The tendency of *h*Gal-1 to form dimers under ITF assay conditions may also play a role.^59^ In any case, the ITF assay confirmed the high affinity of the bisruthenium thiodigalactosides **15-PF**, **15-BF**, and **17-Cl** for *h*Gal-1, and their exceptional selectivity compared to the recently reported inhibitors **1** and **3**.^19,36^ Consistent with the FP assay, the selectivity of the complexes increased in the order **17-Cl** < **15-BF** < **15-PF**. The increased affinity observed for tetrafluoroborate and hexafluorophosphate complexes may reflect their increasing chaotropic character.^60^ In addition, the recently reported tendency of anions to form tight ion pairs with *p*-cymene ruthenium complexes may also affect the binding affinity of the associated ruthenium cation complex.^61^

**Table 3:**
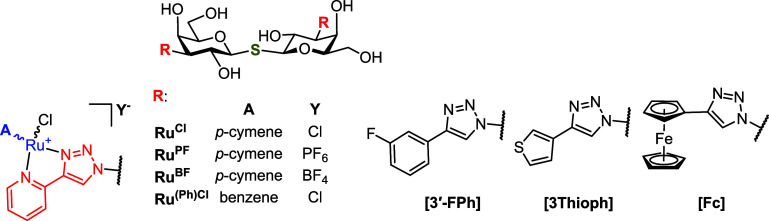
Dissociation Constant *K*_D_ and Selectivity Determined by ITF

Compounds	R	*K*_D_(*h*Gal-1) [μM]	*K*_D_(*h*Gal-3-CRD) [μM]	Selectivity to *h*Gal-1^a^
**15-PF**	**Ru** ^**PF**^	**0.0005 ± 0.0002**	**9.0 ± 2.0**	**18 000**
**15-BF**	**Ru** ^**BF**^	0.00094 ± 0.00007	3.4 ± 0.7	3 600
**17-Cl**	**Ru** ^**(Ph)Cl**^	0.0028 ± 0.0002	2.8 ± 0.5	1 000
**GB0139** ^b^	[**3′-FPh**]	0.08 ± 0.01	0.0021 ± 0.0004	0.03
**1**	**[3Thioph]**	0.0012 ± 0.0001	0.010 ± 0.002	8.3
**3** ^*b*^	**[Fc]**	0.010 ± 0.001	0.40 ± 0.10	40

a Defined as the ratio *K*_D_(*h*Gal-3)/*K*_D_(*h*Gal-1).

b Adopted from ref. 36.

#### Assessment of the Best Inhibitors by Biolayer Interferometry

To gain further insight into the inhibitory potential of the best-performing inhibitors, the binding affinity of compounds **15**-**Cl/BF**/**PF** was assessed by biolayer interferometry (BLI, Table 4). This label-free solid-phase-based technique provides real-time measurements of molecular interactions and, in addition to the dissociation constant *K*_D_, it also determines the kinetic parameters—association rate constant (*k*_a_) and dissociation rate constant (*k*_d_). The respective galectin carrying a 15-aa AVI-tag (GLNDIFEAQKIEWHE) labeled with a single molecule of biotin is immobilized on a streptavidin-coated biosensor via biotin–streptavidin interactions. To avoid compromising lectin activity, a short peptide linker was inserted between the AVI-tag and *h*Gal-1 sequence as previously published.^62^ In the case of *h*Gal-3, the AVI-tag was placed on the flexible N-terminus. The AVI-tag is monobiotinylated during protein expression by coexpressed *Bir*A biotin ligase. All measurements were evaluated using the one-to-one kinetic model, the simplest model for describing ligand–receptor binding, and were cross-validated with steady-state analysis to ensure data accuracy. Unlike the previous two methods, this method analyzes binding to full-length *h*Gal-3 instead of *h*Gal-3-CRD.

**Table 4:** Dissociation Constant (*K*_D_) and Kinetic Constants *k*_a_ and *k*_d_ of Selected Inhibitors Determined by BLI

Entry	Compound	*k*_a_ [L mol^–1^ s^–1^]	*k*_d_ (s^–1^) × 10^–4^	*K*_D_ [μM]	SS^a^ *K*_D_ [μM]
*h*Gal-1					
1	**GB0139**	68 000 ± 29 000	22 ± 10	0.032 ± 0.018	0.037 ± 0.009
2	**15**-**Cl**	2 052 000 ± 208 000	62 ± 15	0.003 ± 0.001	0.004 ± 0.001
3	**15**-**BF**	1 200 000 ± 250 000	136 ± 42	0.011 ± 0.003	0.002 ± 0.002
4	**15**-**PF**	1 670 000 ± 540 000	55 ± 36	0.003 ± 0.002	0.003 ± 0.002
*h*Gal-3					
5	**GB0139**	45 000 ± 12 000	1.2 ± 1.0	0.003 ± 0.002	0.013 ± 0.07
6	**15**-**Cl**	2590 ± 420	265 ± 7	10 ± 3	15 ± 6
7	**15**-**BF**	60 ± 11	39 ± 13	65 ± 23	12 ± 5
8	**15**-**PF**	4250 ± 250	43 ± 6	1 ± 0.1	16 ± 6

a SS = steady-state analysis.

For *h*Gal-1, all tested complexes exhibited low nanomolar affinity by BLI, with slight variations in *K*_D_ values depending on the counterion (*K*_D,**15‑Cl**_ = 3 nM, *K*_D,**15‑BF**_ = 11 nM, *K*_D,**15‑PF**_ = 3 nM, Table 4) while **GB0139** was up to an order of magnitude weaker inhibitor (*K*_D_ = 32 nM). For *h*Gal-3, BLI confirmed the previously published high binding affinity of **GB0139** and its status as one of the leading compounds (*K*_D_ = 3 nM). Among the ruthenium-based compounds, **15-PF** (with a PF_6_^–^ counterion) showed the highest affinity to *h*Gal-3 and was therefore less selective than complexes **15-Cl**/**BF**, but all of them had at least 2 orders of magnitude selectivity over *h*Gal-3. Steady-state analysis, however, revealed comparable *h*Gal-3 affinity (*K*_D_ = 12–16 μM) and 3 orders of magnitude *h*Gal-1 selectivity for all three complexes. While **GB0139** had a comparable association rate constant *k*_a_ for both *h*Gal-1 and *h*Gal-3, the organoruthenium complexes exhibited association rate constants for *h*Gal-1 that were several orders of magnitude higher than those for *h*Gal-3. This suggests that the complex of *h*Gal-3 with the organoruthenium inhibitors forms more slowly because *h*Gal-3 must undergo a conformational change in order to recognize them,^63^ consistent with the presumed steric constraints of the *h*Gal-3 binding site that disfavor binding of organoruthenium inhibitors.

### Inhibition of Galectin Binding to Cancer Cells

Complexes **15-Cl/BF/PF**, which demonstrated a high inhibitory potential in galectin-binding assays, were evaluated for their ability to scavenge *h*Gal-1 and prevent its binding to the cell surface. The inhibition of extracellular *h*Gal-1 binding to the cell surface mimics the *in vivo* behavior of inhibitors and suggests their ability to interfere with *h*Gal-1-mediated signaling pathways. We used the breast cancer cell line MDA-MB-231, which endogenously expresses several galectins, namely *h*Gal-1, *h*Gal-3, and *h*Gal-8. This cell line has been widely reported as a suitable model for *in vitro* assays investigating galectin-related interactions.^64–66^ For the competitive binding assay, we employed a previously published^62^ *h*Gal-1 protein construct carrying a monobiotinylated AVI-tag, *h*Gal-1-AVI. The biotinylated *h*Gal-1-AVI was selectively detected on the cell surface by flow cytometry using streptavidin–phycoerythrin conjugates.

In the absence of glycomimetics, direct binding assays with increasing concentrations of *h*Gal-1-AVI (Supporting Information, Figure S19A) revealed a clear interaction with the MDA-MB-231 cell surface: The binding curve had a nonsaturated character, which indicates a relatively weak binding of *h*Gal-1-AVI to the natural glycocalyx on MDA-MB-231 cells. Consequently, the *IC*_50_ values obtained for the glycomimetics in this assay were notably lower compared to other techniques, including lactose as the standard inhibitor, as it was easier to detach *h*Gal-1-AVI from the cell surface. All tested glycomimetics exhibited remarkable potency in inhibiting *h*Gal-1 binding, thus preventing it from interacting with the cell surface (Table 5 and Figure 5). Among the compounds tested, the most effective inhibitors were **15-BF** and **15-PF**, which exhibited virtually the same inhibition potential with their *IC*_50_ values within the experimental error (*IC*_50_ = 48 ± 15 and 64 ± 26 pM, respectively), while **15-Cl** with chloride counterion showed weaker inhibition (*IC*_50_ = 340 ± 170 pM). These results highlight the potential of ruthenium-based glycomimetics to efficiently inhibit the binding of *h*Gal-1 to the cancer cell surface, offering a promising approach for targeting *h*Gal-1-mediated signaling in therapeutic applications.

**Table 5:** Ability of Thiodigalactoside Analogs **15**-**Cl**/**BF**/**PF** to Inhibit Binding of *h*Gal-1-AVI to the Surface of MDA-MB-231 Cells

Entry	Ligand	*IC*_50_ [pM]	*rp* ^a^
**1**	Lac (standard)	4.3 ± 0.5 × 10^6^	1
**2**	**15**-**Cl**	340 ± 170	12 700
**3**	**15**-**PF**	64 ± 26	67 000
**4**	**15**-**BF**	48 ± 15	90 000

a Relative potency, *rp* = *IC*_50_(Lac)/*IC*_50_(glycomimetic).

**Figure 5:**
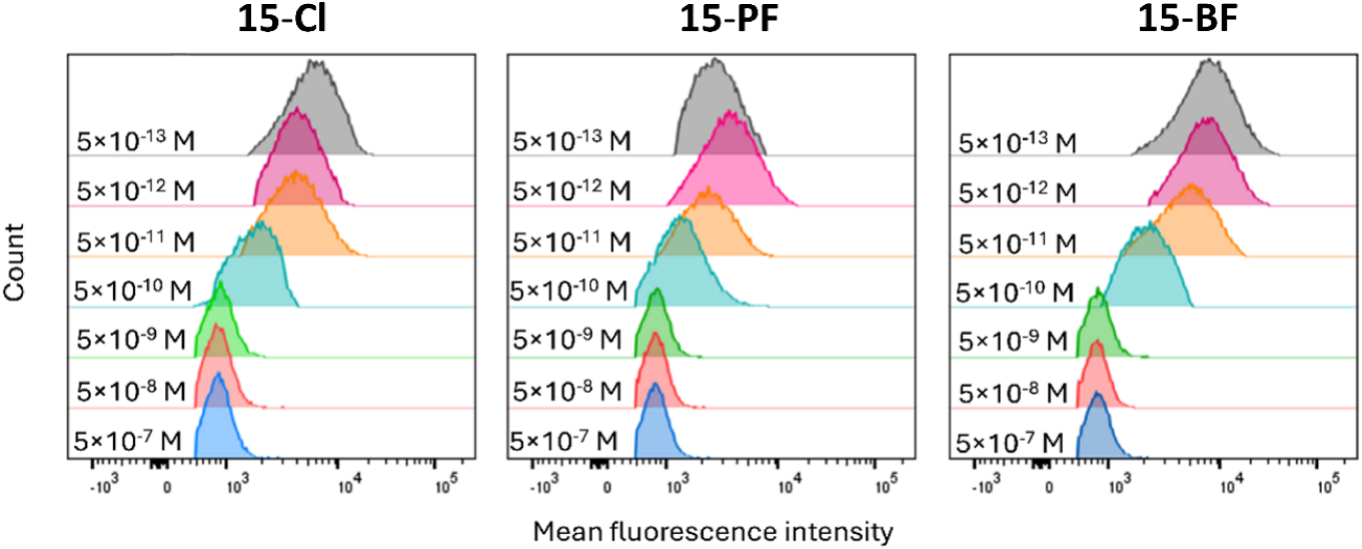
Overlay of representative flow cytometry histograms of competitive inhibition of the binding of the *in vivo* biotinylated *h*Gal-1-AVI to the MDA-MB-231 cell surface in the presence of increasing concentrations of **15**-**Cl**/**BF**/**PF** complexes. The residual galectins bound to the cell surface were stained by the streptavidin-phycoerythrin conjugate. The corresponding inhibition curves are shown in the Supporting Information, Figure S19B.

### Inhibition of *h*Gal-1-Induced Preaparesis in Jurkat Cells

The *h*Gal-1 protein promotes tumor escape from T cell-dependent antitumor immunity by inducing phosphatidylserine exposure in T cells, a process referred to as preaparesis, that occurs independently of apoptosis.^67–70^ We tested the ability of the complexes **11-Cl**, **13-PF**, **15-PF**, **16-I**, **17-Cl**, and the benchmark inhibitor thiodigalactoside **GB0139** to inhibit *h*Gal-1-induced preaparesis in Jurkat cells, a human T-lymphoblasticleukemia cell line,^71^ at molar ratios of inhibitor to *h*Gal-1 of 1:1 and 1:2 (Figure 6 and Figure S20 in the Supporting Information). The commercially available inhibitor **GB0139** fully suppressed preaparesis at both tested ratios. The weak inhibitor **11-Cl** based on C-1-substituted lactose demonstrated almost no ability to attenuate *h*Gal-1-induced preaparesis. In contrast, the more potent LacNAc-based complex **13-PF** almost completely prevented preaparesis at an equimolar ratio with *h*Gal-1 and significantly inhibited it at a ratio of 1:2. The strong TDG-based inhibitors **15-PF**, **16-I**, and **17-Cl** fully blocked preaparesis at both 1:1 and 1:2 ratios. These results demonstrate that organoruthenium inhibitors effectively block propreaparetic signaling in T cells and highlight their potential to counteract tumor immune escape.

**Figure 6:**
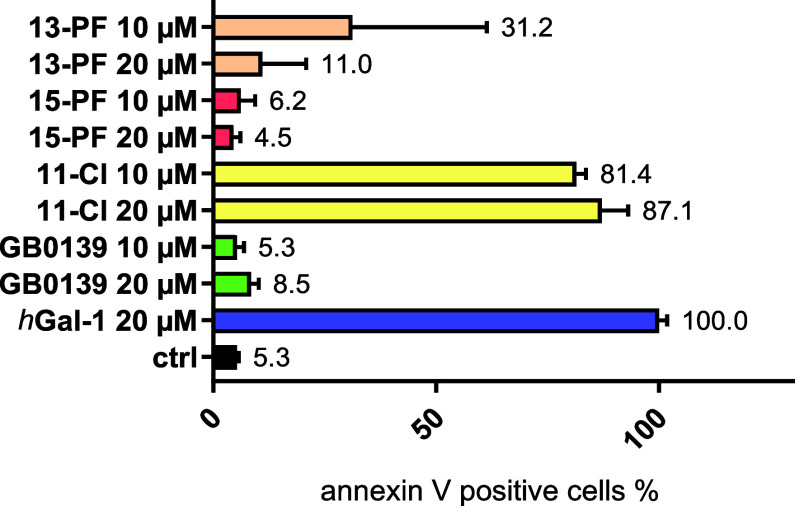
Inhibition of *h*Gal-1 (20 μM)-induced preaparesis in Jurkat T cells by glycomimetic inhibitors. Preaparesis features were assessed by flow cytometry after Annexin V-Alexa Fluor 488 staining of phosphatidylserine externalization. The data represent the mean ± standard deviation (SD) of at least three independent experiments and are normalized to the preparesis level caused by *h*Gal-1 (20 μM) alone. The results for complexes **16-I** and **17-Cl** are shown in Figure S20 in the Supporting Information. The organoruthenium inhibitors and **GB0139** alone induced a normalized increase in the Annexin V-positive cell population by a maximum of 8% (Figure S21 in the Supporting Information). Representative histograms of individual samples, including negative controls, are shown in Figures S22 and S23 in the Supporting Information.

### Cytotoxicity

The cytotoxicity of all synthesized organometallic compounds and the bispyridyl precursor **21** was evaluated using the MTT assay in the breast cancer cell line MDA-MB-231 and in noncancerous HEK-293 cells, which do not express detectable levels of *h*Gal-1 as confirmed by Western blot analysis (Figure 7 and Figures S24–S32 in the Supporting Information). Compounds **11-Cl/PF**, **12-Cl**, **13-Cl/PF**, **17-Cl**, and **21** exhibited negligible cytotoxicity for both cell lines at concentrations up to 100 μM. In contrast, the bisruthenium LacNAc analog **14-Cl** and the three most potent *h*Gal-1 inhibitors, **15-BF/PF**, and **16-I**, reduced the viability of the *h*Gal-1-expressing MDA-MB-231 cells by up to 50% at concentrations typically of 25–100 μM (Figure 7). Cytotoxicity against HEK-293 cells was negligible for most of the compounds. Only inhibitor **15-Cl** induced a slight decrease in the viability of both cell lines (Figure S29 in the Supporting Information). Surprisingly, the hexamethylbenzene inhibitor **18-Cl** exhibited a distinct cytotoxicity profile with a higher toxicity for HEK-293 cells than for MDA-MB-231 cells (Figure S31 in the Supporting Information). The higher cytotoxicity of potent *h*Gal-1 inhibitors toward *h*Gal-1-expressing MDA-MB-231 tumor cells might indicate that inhibition of *h*Gal-1, which is overexpressed in these cells, affects signaling or redox homeostasis, thereby slightly increasing the sensitivity to ruthenium complexes. This hypothesis is supported by the negligible cytotoxicity of compound **21** (Supporting Information, Figure S32), which has *h*Gal-1 inhibitory activity comparable to the cytotoxic **14-Cl** complex but lacks the ruthenium complex (*K*_i,**14‑Cl**_ = 0.20 ± 0.04 μM, *K*_i,**21**_ = 0.16 ± 0.05 μM, see Figure 7A for cytotoxicity of **14-Cl**). Moreover, the application of inhibitors to the *h*Gal-1-negative HEK-293 cell line did not result in any significant cytotoxic effect, except for complex **18-Cl**, which further supports our hypothesis. However, other cellular differences between MDA-MB-231 and HEK-293 cells may also influence the compound uptake or susceptibility. Therefore, while our results do not demonstrate a direct mechanistic role of *h*Gal-1 in ruthenium-induced cytotoxicity, they are consistent with the hypothesis that modulation of Gal-1-dependent pathways may influence cellular susceptibility to metal-based compounds.^72^

**Figure 7:**
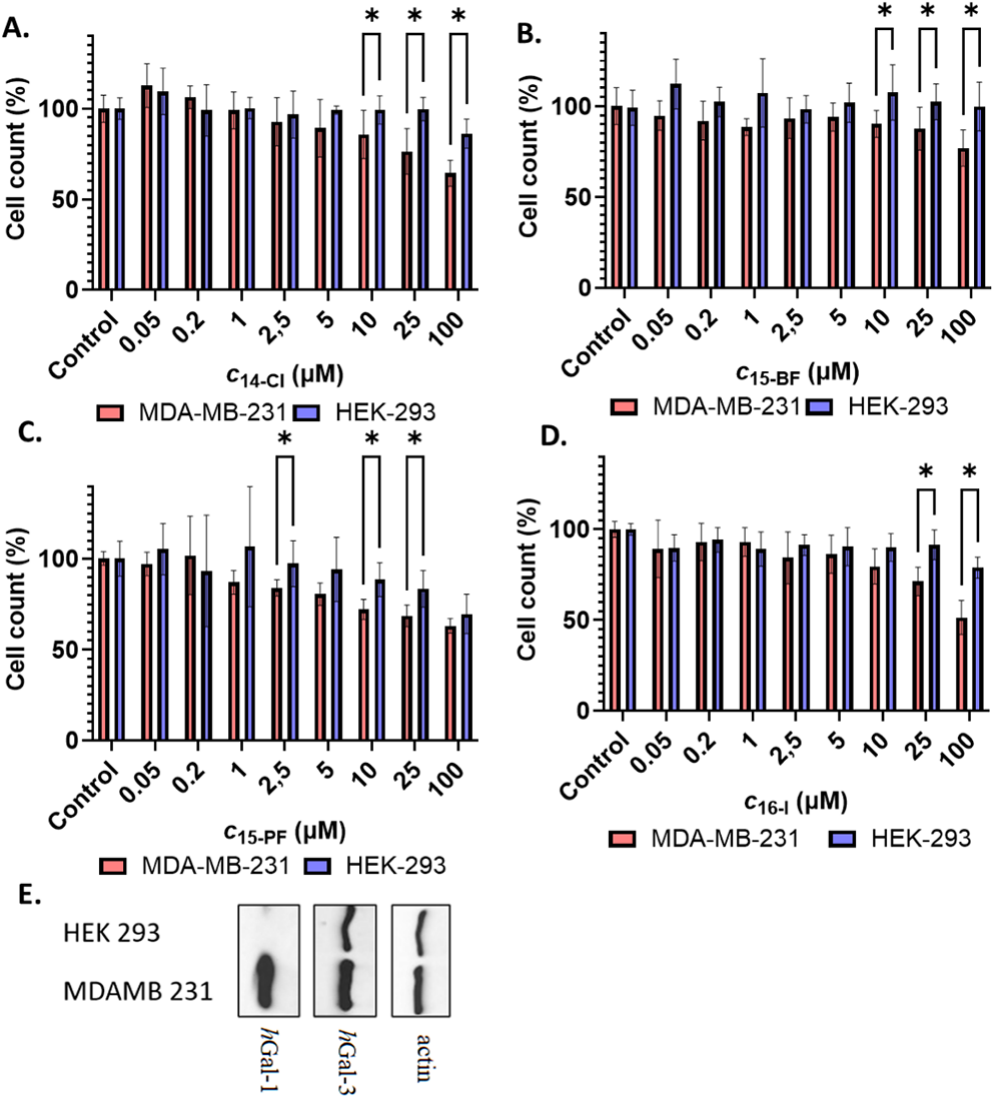
(**A**–**D**) Cytotoxicity of complexes **14-Cl**, **15-BF/PF**, and **16-I** for the breast cancer MDA-MB-231 cell line and noncancerous HEK-293 cell line after 72 h of incubation. (**E**) Western blots of *h*Gal-1 and *h*Gal-3 expression in MDA-MB-231 and HEK-293 cell lines. Asterisks indicate statistically significant differences determined by one-way ANOVA (*P* < 0.01).

## Discussion

An important breakthrough in the development of potent selective small-molecule glycomimetic *h*Gal-3 inhibitors was the introduction of an arene-triazole moiety at the 3-position of the galactopyranoside moieties of the LacNAc and thiodigalactoside scaffolds,^47,73,74^ which led to the discovery of the selective *h*Gal-3 inhibitors, including **GB0139**.^19^ On the basis of X-ray crystallography and molecular dynamics simulations,^36,38^ we hypothesized that substituting the arene moieties in **GB0139** with a three-dimensional organometallic fragment may improve selectivity and the binding affinity to *h*Gal-1 due to steric constraints of the *h*Gal-3 binding site. Investigation of diferrocenylthiodigalactoside **3** (Figure 1A)^36^ confirmed this hypothesis, but as its *h*Gal-1 selectivity was still moderate, we sought for another stable and inert organotransition metal complex. Since pseudo-octahedral ruthenium­(II) complexes are characterized by high inertness and kinetic stability,^75–77^ we prepared a series of analogs, in which the arene-triazolyl moieties in LacNAc and thiodigalactoside-based inhibitors were substituted with half-sandwich ruthenium­(II) arene complexes. The bidentate 4-(2-pyridyl)-1*H*-1,2,3-triazol-1-yl coordinating ligand in these half-sandwich organoruthenium complexes enhances their stability and inertness,^78^ and mimics the arene-triazole substituents found in potent galectin inhibitors^73^ while the ruthenium atom serves as an inert center that precisely organizes the coordinating ligands in space.

Three different types of binding assays independently revealed that the introduction of the half-sandwich organoruthenium substituent at the 3-position of the galactopyranose unit of both LacNAc and TDG increased the *h*Gal-1 selectivity by at least 2 orders of magnitude: it strongly reduced the inhibitory activity to *h*Gal-3 while improving the *h*Gal-1 inhibitory activity. Comparison with the respective uncoordinated 2-pyridyl-triazole-modified disaccharide analogs (cf. **20** and **21** with **14-Cl** and **15-Cl**, respectively) shows that while the *h*Gal-1 binding affinity is moderately (**14-Cl**) or slightly (**15-Cl**) improved upon complexation with ruthenium, the *h*Gal-3 binding affinity decreases dramatically upon coordination with ruthenium (Tables 1 and 2). The ruthenium complex probably effectively mimics the binding interactions between *h*Gal-1 and 2-pyridyl-triazole moieties found in precursors **20** and **21**; however, more importantly, it strongly disfavors binding to *h*Gal-3, which correlates with the three- to four-orders-of-magnitude lower association rate of TDG-based organoruthenium inhibitors to *h*Gal-3 (Table 5) compared with *h*Gal-1 in BLI. We hypothesize that the three-dimensional organoruthenium complex does not readily fit into the *h*Gal-3 binding site, which must undergo a conformational change to accommodate this complex,^63^ resulting in low association rates and low binding affinity. The decrease in affinity to *h*Gal-3 observed for the 3′-substituted LacNAc analogs **13-Cl** and **13-PF** compared to their uncoordinated precursor **19** suggests that the A and B subsites in the binding groove particularly discriminate between the two galectins because the modification at the 3′-position of the LacNAc scaffold positions the organoruthenium complex in galectin binding subsites A and B.^37,43,79,80^

Counterion exchange with more lipophilic anions further positively modulated the *h*Gal-1 binding affinity in the TDG series; inhibitor **15-PF** achieved an unprecedented combination of *h*Gal-1 binding affinity and selectivity, highlighting the potential of organometallic glycomimetics in lectin inhibition. Inert metal complexes have previously been used as protein binders,^41^ notably in a series of potent and selective protein kinase inhibitors based on staurosporin mimetics obtained by substitution of the tetrahydropyran moiety in staurosporin with octahedral ruthenium­(II) complexes.^81–84^ However, with the exception of our ferrocene-based *h*Gal-1 inhibitors,^36^ to our knowledge, organometallic structures have never been used in the design of lectin inhibitors, though ferrocene moieties in multivalent glycomimetics have been tested as electroactive probes.^85–87^

A cellular *in vitro* assay demonstrated that the most potent thiodigalactoside-based organoruthenium inhibitors effectively inhibit the binding of extracellular *h*Gal-1 to the surface of MDA-MB-231 cancer cells in a dose-dependent manner, a prerequisite to counteract protumor *h*Gal-1-mediated signaling. Moreover, inhibitor **15-PF** was able to completely suppress *h*Gal-1-induced preaparesis in Jurkat cells, highlighting its potential to block this important mechanism of tumor immune escape. The biological relevance of *h*Gal-1 inhibition extends beyond the preaparesis induction in T cells, because *h*Gal-1 is a key regulator of immune tolerance in the tumor microenvironment, where it promotes T cell exhaustion and expansion of regulatory T cells while excluding cytotoxic lymphocytes from the tumor niche. Inhibiting extracellular *h*Gal-1 binding to glycan structures on T cells or endothelial cells may therefore not only restore T cell viability but also enhance their trafficking and persistence in tumors. Given the capacity of **15-PF** to prevent *h*Gal-1-induced preaparesis *in vitro*, it is plausible that this compound could reprogram the immune landscape *in vivo*, ultimately potentiating antitumor immunity.

## Conclusion

Motivated by the emerging significance of selective potent galectin inhibition in medicinal chemistry research and the urgent need to develop small-molecule inhibitors selective for individual galectins, we have developed an operationally simple synthesis of potent nanomolar inhibitors of *h*Gal-1 that are by 2 to 3 orders of magnitude selective over *h*Gal-3. The unprecedented selectivity was achieved by installing a half-sandwich ruthenium­(II) *p*-cymene complex containing the 4-(2-pyridyl)-1*H*-1,2,3-triazol-1-yl substituent as a bidentate ligand at the 3- and 3′-positions of the TDG scaffold. The most potent inhibitors not only displayed strong binding to *h*Gal-1 but also effectively prevented the binding of *h*Gal-1 to the tumor cell surface and suppressed *h*Gal-1-induced preaparesis in Jurkat cells, demonstrating the potential of these inhibitors to interfere with protumorigenic *h*Gal-1 signaling pathways. It is envisaged that introducing inert organometallic complexes into glycomimetics will promote the exploration of new dimensions of the glycochemical space and accelerate the discovery of novel potent, selective inhibitors of therapeutically relevant lectins.

## Experimental Section

### General Chemistry Methods

Chemicals were used as received. Petroleum ether fraction (with boiling point 40–65 °C) was distilled before use. TLC was carried out with Sigma-Aldrich TLC Silica gel 60 F_254_, and spots were detected by UV detection at 254 nm or visualized with an anisaldehyde solution (EtOH/anisaldehyde/AcOH/H_2_SO_4_ 340:15:4:12.5, v/v). Column chromatography was performed with silica gel 60 (70–230 mesh, Material Harvest). If not stated otherwise, solutions were concentrated under reduced pressure at temperatures below 45 °C. Anhydrous sodium sulfate was used to dry solutions after aqueous workup. NMR spectra were recorded using Bruker Avance 400 (^1^H at 400.1 MHz, ^19^F at 376.4 MHz, ^13^C at 100.6 MHz) at 25 °C. The ^1^H and ^13^C NMR spectra were referenced to the solvent (δ/ppm; δH/δC: CDCl_3_, 7.26/77.16, CD_3_OD, 3.31/49.00, and DMSO-*d*_6_, 2.50/39.52). The ^19^F NMR spectra were referenced to the line of the internal standard hexafluorobenzene (δ/ppm; −163.00 in CDCl_3_, −166.62 in CD_3_OD, and −163.86 in DMSO-*d*_6_). The structural assignment of proton and carbon NMR spectra was made by a combination of 1D and 2D NMR measurements: ^1^H–^1^H gCOSY, ^1^H–^13^C gHSQC, ^1^H–^13^C gHMBC, and ^1^H–^13^C gHSQC TOCSY. HRMS analyses were done using a Bruker MicrOTOF-QIII, using ESI ionization in the positive mode. The purities of the compounds were determined by NMR and HPLC, and they were ≥95%. The copies of HPLC chromatograms for the most potent inhibitors are in the Supporting Information. The ruthenium, copper, silver, phosphor, and boron contents in the final compounds were analyzed using an atomic emission spectrometer with microwave-induced plasma (MP-OES Agilent Technologies, Inc., USA) equipped with an autosampler model SPS 3 (Agilent Technologies, Inc., USA) in a 1% HNO_3_ solution. In all cases, the copper and silver content was below the spectrometer’s detection limit, while the ruthenium, boron, and phosphor content corresponded with the calculated values. See Supporting Information, Schemes S1–S5 for the structures of synthetic intermediates **S1**–**S7**.

### General Procedure for an Azide–Alkyne Cycloaddition

Copper­(II) sulfate pentahydrate (12 mg, 0.05 mmol), ascorbic acid (8 mg, 0.05 mmol), and 2-ethynylpyridine (1.2–1.5 equiv per reacting azido group) were added into a solution of azido sugar (1 equiv) in DMF (*c* = 0.1–0.2 M) and the reaction mixture was stirred at 40 °C for 2 h. The reaction mixture was allowed to cool to room temperature; then, it was diluted with DCM (30 mL) and washed with water (30 mL), and the aqueous phase was extracted with DCM (2 × 20 mL). The combined organic phases were dried and concentrated, and the crude product was purified by column chromatography.

### General Procedure for Deacetylation

The acetylated compound was dissolved or dispersed in the mixture of DCM and MeOH 1:1 (*c* ≈ 0.01 M), and a 0.5 M solution of MeONa in MeOH was added dropwise until pH = 9–10. The reaction mixture was stirred for 12–72 h at rt until TLC (DCM/MeOH) indicated the end of the reaction and the presence of only one polar product. The reaction was neutralized with DOWEX 50W­(H^+^) ion-exchange resin, filtered, and concentrated. The crude product was purified by column chromatography if necessary.

### General Procedure for the Complexation Reaction

A dark red solution of dihalogenido­(arene)­ruthenium­(II) dimer (0.5 equiv) in DCM (*c* = 0.05 M) was added to a solution of sugar ligand (1 equiv) in a 1:1 mixture of MeOH/DCM (*c* = 0.05 M). Almost immediately, a color change from red to yellow occurred. The mixture was stirred at room temperature for 10 min. The reaction mixture was concentrated, and the residue was dissolved in a minimal amount of methanol. The organoruthenium complex precipitated by slow addition of methyl *tert*-butyl ether as a yellow solid mixture of diastereomers. A sample free from the traces of residual solvents was obtained by dissolution of the product in a small amount of methanol and removal of the residual solvents by coevaporation with chloroform under reduced pressure. The target ruthenium complexes **11**–**13** were obtained as a mixture of two diastereomers due to the introduction of a new stereogenic center at ruthenium. Bisruthenium complex **14** was obtained as a mixture of four diastereomers because two new stereocenters were introduced into the molecule. Three diastereomers were formed in the case of bisruthenium complexes **15**–**18**, because the *R*,*S* and *S*,*R* combinations at the two ruthenium centers are identical molecules due to the *C*_2_ axis of symmetry. However, the nuclei related by the *C*_2_ symmetry operation are diastereotopic in this *R*,*S* (= *S,R*) diastereomer, although they are not always distinguishable in NMR spectra. Consequently, some nuclei in samples **15**-**18**, typically the triazole proton, can produce up to four signals at very close but distinct frequencies. Whenever possible, we estimated and reported the ratio of stereoisomers of a given product from the analysis of NMR spectra.

### General Procedure for Counterion Exchange

A solution of AgPF_6_ (1.05 equiv) or AgBF_4_ (1.05 equiv) in MeOH (*c* = 0.02 M) was added dropwise into a solution of organoruthenium-chloride complex in MeOH (*c* = 0.01 M), and the reaction mixture was stirred at rt for 10 min in the dark, filtered through a syringe filter (VWR Syringe Filter, Nylon, 25 mm, 0.45 μm), and concentrated. The crude product was dissolved in a minimal amount of methanol, and the organoruthenium complex precipitated by slow addition of methyl *tert*-butyl ether as a yellow solid mixture of diastereomers. A sample free from traces of residual solvents was obtained by the dissolution of the product in a small amount of methanol and removal of the residual solvents by coevaporation with chloroform under reduced pressure.

#### 1-[4-*O*-(β-d-Galactopyranosyl)-β-d-glucopyranosyl]-4-(pyridin-2-yl)-1*H*-1,2,3-triazole (**S2**)

Copper­(II) sulfate pentahydrate (12 mg, 0.05 mmol), ascorbic acid (8 mg, 0.05 mmol), 2-ethynylpyridine (73 μL, 0.72 mmol), and β-lactosyl azide peracetate^49^ (400 mg, 0.60 mmol) were reacted according to the general procedure of azide–alkyne cycloaddition. Column chromatography in EtOAc/PE 2:1 provided O-acetylated 1-(β-lactosyl)-4-(pyridin-2-yl)-1*H*-1,2,3-triazole (**S1**, 372 mg, 80%, see the Supporting Information, Scheme S1, for the structure) as a white amorphous solid. ^1^H NMR (CD_3_OD, 400 MHz, H–H COSY): δ 8.62 (s, 1H, C*H*_Tria_), 8.59 (ddd, 1H, *J* = 4.8, 1.8, 1.2 Hz, C*H*_Py_), 8.09 (dt, 1H, *J* = 7.9, 1.2 Hz, C*H*_Py_), 7.93 (ddd, 1H, *J* = 7.9, 7.6, 1.8 Hz, C*H*_Py_), 7.19 (ddd, 1H, *J* = 7.6, 4.8, 1.2 Hz, C*H*_Py_), 6.17 (d, 1H, *J* = 9.2 Hz, H-1), 5.61 (dd, 1H, *J* = 9.6, 9.2 Hz, H-2), 5.50 (dd, 1H, *J* = 9.6, 8.2 Hz, H-3), 5.39 (d, 1H, *J* = 3.4 Hz, H-4′), 5.15 (dd, 1H, *J* = 10.4, 3.4 Hz, H-3′), 5.06 (dd, 1H, *J* = 10.4, 7.8 Hz, H-2′), 4.78 (d, 1H, *J* = 7.8 Hz, H-1′), 4.59 (dd, 1H, *J* = 11.9, 0.8 Hz, H-6a), 4.23–4.11 (m, 6H, H-4, H-5, H-6b, H-5′, H-6′a, H-6′b), 2.15, 2.10, 2.08 (3 × s, 3 × 3H, *Me*), 2.07 (2 × s, 2 × 3H, *Me*), 1.94, 1.85 (2 × s, 2 × 3H, *Me*). ^13^C­{^1^H} NMR (CD_3_OD, 101 MHz, HSQC, HMBC): δ 172.4, 172.1, 172.0, 171.7, 171.5, 171.2, 170.6 (7 × *C*O), 150.6 (*C*H_Py_, *C*_q(Py)_), 149.1 (*C*_q(Tria)_), 139.0, 124.8 (2 × *C*H_Py_), 123.2 (*C*H_Tria_), 121.7 (*C*H_Py_), 102.1 (C-1′), 86.6 (C-1), 77.1 (C-4, C-5), 74.2 (C-3), 72.5 (C-3′), 72.2 (C-2), 71.9 (C-5′), 70.7 (C-2′), 68.6 (C-4′), 63.5 (C-6), 62.4 (C-6′), 21.1, 20.71, 20.65, 20.6 (4 × *Me*), 20.5 (2 × *Me*), 20.1 (*Me*). HRMS-ESI (*m*/*z*): [M + H]^+^ calculated for C_33_H_41_N_4_O_17_, 765.2461; found 765.2460.

Compound **S2** (see the Supporting Information, Scheme S1, for the structure) was prepared according to the general procedure for deacetylation starting from **S1** (200 mg, 0.26 mmol). The reaction mixture was stirred at room temperature overnight. Neutralization with ion-exchange resin DOVEX 50W­(H^+^), filtration, and solvent evaporation yielded **S2** (122 mg, quantitative, 80% over two steps) as a yellowish amorphous solid, *R*_*f*_ 0.40 (DCM/MeOH 5:1), ${[\alpha ]}_{D}^{20}$ −21 (*c* 0.84, MeOH).^1^H NMR (CD_3_OD, 400 MHz, H–H COSY): δ 8.66 (s, 1H, C*H*_Tria_), 8.59 (br s, 1H, C*H*_Py_), 8.10 (d, 1H, *J* = 7.7 Hz, C*H*_Py_), 7.93 (dd, 1H, *J* = 7.9, 7.7 Hz, C*H*_Py_), 7.40 (br s, 1H, C*H*_Py_), 5.75 (d, 1H, *J* = 9.2 Hz, H-1), 4.46 (d, 1H, *J* = 7.6 Hz, H-1′), 4.06 (dd, 1H, *J* = 9.2, 9.0 Hz, H-2), 3.94–3.85 (m, 2H, H-6a, H-6b), 3.83–3.76 (m, 5H, H-3, H-4, H-5, H-4′, H-6′a), 3.74 (dd, 1H, *J* = 11.5, 4.5 Hz, H-6′b), 3.65 (ddd, 1H, *J* = 7.4, 4.5, 0.9 Hz, H-5′), 3.61 (dd, 1H, *J* = 9.8, 7.6 Hz, H-2′), 3.53 (dd, 1H, *J* = 9.8, 3.3 Hz, H-3′). ^13^C­{^1^H} NMR (CD_3_OD, 101 MHz, HSQC, HMBC, HSQC TOCSY): δ 150.6 (*C*H_Py_), from HMBC 148.6 (*C*_q(Tria)_), not detected (*C*_q(Py)_), 139.1 (*C*H_Py_), from HSQC 124.7 (*C*H_Py_), 123.4 (*C*H_Tria_), 121.8 (*C*H_Py_), 105.1 (C-1′), 89.5 (C-1), 79.61/79.58 (C-4/5), 77.1 (C-5′), 76.8 (C-3), 74.8 (C-3′), 73.7 (C-2), 72.5 (C-2′), 70.3 (C-4′), 62.5 (C-6′), 61.5 (C-6). HRMS-ESI (*m*/*z*): [M + H]^+^ calculated for C_19_H_27_N_4_O_10_, 471.1722; found 471.1724.

#### Complex **11-Cl**

Complex **11-Cl** was prepared according to the general complexation procedure starting from dichloro­(*p*-cymene)­ruthenium­(II) dimer (26 mg, 0.042 mmol) in DCM (1 mL) and the precursor **S2** (40 mg, 0.085 mmol) in a 1:1 MeOH/DCM mixture (2 mL). The product **11-Cl** (49 mg, 74%) precipitated from MeOH solution by the addition of methyl *tert*-butyl ether as a yellow solid mixture of two diastereomers in a ratio of 1:0.8 (^1^H NMR). ^1^H NMR (CD_3_OD, 400 MHz, H–H COSY): δ 9.42 (dd, 2 × 1H, *J* = 7.4, 1.4 Hz, C*H*_Py_), 9.26, 9.24 (2 × s, 2 × 1H, C*H*_Tria_), 8.19 (ddd, 2 × 1H, *J* = 8.1, 5.7, 1.4 Hz, C*H*_Py_), 8.12 (dd, 2 × 1H, *J* = 8.1, 3.4 Hz, C*H*_Py_), 7.68 (ddd, 2 × 1H, *J* = 7.4, 5.7, 1.5 Hz, C*H*_Py_), 6.12, 6.07 (2 × m, 2 × 2H, C*H*_*p*‑cym_), 5.95, 5.92 (2 × d, 2 × 1H, *J* = 9.2 Hz, H-1), 5.90, 5.85 (2 × m, 2 × 2H, C*H*_*p*‑cym_), 4.45 (2 × d, 2 × 1H, *J* = 7.6 Hz, H-1′), 4.05, 3.98 (2 × dd, 2 × 1H, *J* = 9.2, 7.8 Hz, H-2), 3.96 (br s, 2 × 2H, H-6a, H-6b), 3.85–3.80 (m, 5 × 2H, H-3, H-4, H-5, H-4′, H-6′a), 3.74 (2 × dd, 2 × 1H, *J* = 11.4, 4.5 Hz, H-6′b), 3.65–3.60 (m, 2 × 1H, H-5′), 3.61 (dd, 2 × 1H, *J* = 9.7, 7.6 Hz, H-2′), 3.52 (dd, 2 × 1H, *J* = 9.7, 3.3 Hz, H-3′), 2.76, 2.74 (2 × hept, 2 × 1H, *J* = 6.9 Hz, C*H*_*i*‑Pr_), 2.23 (2 × s, 2 × 3H, *Me*_*p*‑cym_), 1.16, 1.15, 1.10, 1.09 (4 × d, 4 × 3H, *J* = 6.9 Hz, *Me*_*i*‑Pr_). ^13^C­{^1^H} NMR (CD_3_OD, 101 MHz, HSQC, HMBC, HSQC TOCSY): δ 156.8 (2C*H*_Py_), 149.63, 149.62 (2 × *C*_q(Py)_), 148.2, 148.0 (2 × *C*_q(Tria)_), 141.5, 127.6 (2 × 2*C*H_Py_), 125.7, 125.1 (2 × *C*H_Tria_), 123.6 (2*C*H_Py_), 106.7, 106.6 (2 × *C*_q_CH_*i*‑Pr_), 105.2, 105.1 (2 × C-1′), 104.1, 103.8 (2 × *C*_q_Me), 91.0, 90.9 (2 × C-1), 87.4 (2*C*H_*p*‑cym_), 86.2, 86.0, 85.55, 85.49, 84.9, 84.7 (6 × *C*H_*p*‑cym_), 80.1, 80.0 (2 × C-5), 79.5 (2 × C-4), 77.24, 77.23 (2 × C-5′), 76.64, 76.59 (2 × C-3), 74.86, 74.85 (2 × C-3′), 74.1, 73.9 (2 × C-2), 72.5 (2 × C-2′), 70.3 (2 × C-4′), 62.6 (2 × C-6), 61.6, 61.3 (2 × C-6′), 32.31, 32.26 (2 × *C*H_*i*‑Pr_), 22.60, 22.56, 21.93, 21.87 (2 × *Me*_*i*‑Pr_), 18.8, 18.7 (2 × *Me*_*p*‑cym_). HRMS-ESI (*m*/*z*): [M – Cl]^+^ calculated for C_29_H_40_ClN_4_O_10_Ru, 741.1476; found 741.1486.

#### Complex **11-PF**

Complex **11-PF** was prepared according to the general procedure for counterion exchange starting from complex **11-Cl** (30 mg, 0.039 mmol) and AgPF_6_ (10 mg, 0.039 mmol). The product **11-PF** precipitated by slow addition of methyl *tert*-butyl ether as a yellow solid mixture of two diastereomers in a ratio of 1:0.9 (26 mg, 76%). ^1^H NMR (CD_3_OD, 400 MHz, H–H COSY): δ 9.41 (ddd, 2 × 1H, *J* = 5.7, 1.3, 1.6 Hz, C*H*_Py_), 9.23, 9.21 (2 × s, 2 × 1H, C*H*_Tria_), 8.19 (ddd, 2 × 1H, *J* = 7.9, 7.6, 1.3 Hz, C*H*_Py_), 8.11 (ddd, 2 × 1H, *J* = 7.9, 3.2, 1.6 Hz, C*H*_Py_), 7.68 (ddd, 2 × 1H, *J* = 7.4, 5.7, 1.5 Hz, C*H*_Py_), 6.12, 6.06 (2 × m, 2 × 2H, C*H*_*p*‑cym_), 5.94, 5.91 (2 × d, 2 × 1H, *J* = 9.2 Hz, H-1), 5.90, 5.84 (2 × m, 2 × 2H, C*H*_*p*‑cym_), 4.46 (2 × d, 2 × 1H, *J* = 7.6 Hz, H-1′), 4.04, 3.98 (2 × dd, 2 × 1H, *J* = 9.2, 8.4 Hz, H-2), 3.96 (br s, 2 × 2H, H-6), 3.86–3.80 (m, 4 × 2H, H-3, H-4, H-5, H-4′), 3.82 (2 × dd, 2 × 1H, *J* = 11.4, 7.2 Hz, H-6′a), 3.74 (2 × dd, 2 × 1H, *J* = 11.4, 4.5 Hz, H-6′b), 3.65–3.60 (m, 2 × 1H, H-5′), 3.61 (dd, 2 × 1H, *J* = 9.7, 7.6 Hz, H-2′), 3.53 (dd, 2 × 1H, *J* = 9.7, 3.3 Hz, H-3′), 2.76, 2.74 (2 × hept, 2 × 1H, *J* = 6.9 Hz, C*H*_*i*‑Pr_), 2.22 (2 × s, 2 × 3H, *Me*_*p*‑cym_), 1.16, 1.15 (2 × d, 2 × 3H, *J* = 6.9 Hz, *Me*_*i*‑Pr_), 1.09 (2 × d, 2 × 3H, *J* = 6.9 Hz, *Me*_*i*‑Pr_). ^13^C­{^1^H} NMR (CD_3_OD, 101 MHz, HSQC, HMBC, HSQC TOCSY): δ 156.8 (2 × C*H*_Py_), 149.6, 149.5 (2 × *C*_q(Py)_), 148.2, 148.0 (2 × *C*_q(Tria)_), 141.5, 127.7 (2 × 2*C*H_Py_), 125.6, 125.1 (2 × *C*H_Tria_), 123.6 (2 × *C*H_Py_), 106.7, 106.6 (2 × *C*_q_CH_*i*‑Pr_), 105.13, 105.11 (2 × C-1′), 104.0, 103.8 (2 × *C*_q_Me), 90.9, 90.8 (2 × C-1), 87.33, 87.32 (2 × *C*H_*p*‑cym_), 86.2, 86.0, 85.6, 85.5, 84.9, 84.7 (6 × *C*H_*p*‑cym_), 80.04, 79.99 (2 × C-5), 79.46, 79.45 (2 × C-4), 77.20, 77.18 (2 × C-5′), 76.6, 76.5 (2 × C-3), 74.8 (2 × C-3′), 74.1, 73.9 (2 × C-2), 72.5 (2 × C-2′), 70.3 (2 × C-4′), 62.58, 62.56 (2 × C-6), 61.34, 61.31 (2 × C-6′), 32.29, 32.25 (2 × *C*H_*i*‑Pr_), 22.58, 22.55, 21.93, 21.88 (2 × *Me*_*i*‑Pr_), 18.8, 18.7 (2 × *Me*_*p*‑cym_). ^19^F NMR (CD_3_OD, 376 MHz): δ −76.03 (d, ^1^ *J*_(F–P)_ = 707.6 Hz). ^31^P NMR (CD_3_OD, 162 MHz): δ −161.48 (hept, ^1^ *J*_(F–P)_ = 707.6 Hz). HRMS-ESI (*m*/*z*): [M – PF_6_]^+^ calculated for C_29_H_40_ClN_4_O_10_Ru, 741.1476; found 741.1479.

#### Methyl 2-Deoxy-4-*O*-(β-d-galactopyranosyl)-2-[4-(pyridin-2-yl)-1*H*-1,2,3-triazol-1-yl]-β-d-glucopyranoside (**S3**)

Copper­(II) sulfate pentahydrate (12 mg, 0.048 mmol), ascorbic acid (8 mg, 0.045 mmol), 2-ethynylpyridine (15 μL, 0.15 mmol), and methyl 2-azido-2-deoxy-4-*O*-(2,3,4,6-tetra-*O*-acetyl-β-d-galactopyranosyl)-β-d-glucopyranoside^36^ (65 mg, 0.12 mmol) reacted according to the general procedure for azide–alkyne cycloaddition. The acetyl-protected intermediate obtained after aqueous workup and concentration was not chromatographically purified but immediately subjected to deacetylation according to the general procedure. Column chromatography in DCM/MeOH 6:1 afforded product **S3** (45 mg, 79%, see the Supporting Information, Scheme S2, for the structure) as a colorless gel, *R*_f_ 0.15 (DCM/MeOH 7:1), ${[\alpha ]}_{D}^{20}$ −144 (*c* 0.30, MeOH). ^1^H NMR (CD_3_OD, 400 MHz, COSY): δ 8.58 (ddd, 1H, *J* = 4.7, 1.8, 1.2 Hz, C*H*_Py_), 8.49 (s, 1H, C*H*_Tria_), 8.08 (dt, 1H, *J* = 8.0, 1.2 Hz, C*H*_Py_), 7.92 (ddd, 1H, *J* = 8.0, 7.6, 1.8 Hz, C*H*_Py_), 7.37 (ddd, 1H, *J* = 7.6, 4.7, 1.8 Hz, C*H*_Py_), 4.99–4.97 (m, 1H, H-1), 4.44 (d, 1H, *J* = 7.5 Hz, H-1′), 4.36–4.34 (m, 2H, H-2, H-3), 4.01 (dd, 1H, *J* = 12.3, 2.7 Hz, H-6a), 3.97 (dd, 1H, *J* = 12.3, 3.8 Hz, H-6b), 3.83–3.79 (m, 1H, H-4), 3.80 (dd, 1H, *J* = 3.3, 1.0 Hz, H-4′), 3.74 (dd, 1H, *J* = 11.4, 7.7 Hz, H-6′a), 3.69 (ddd, 1H, *J* = 10.0, 3.8, 2.7 Hz, H-5), 3.66 (dd, 1H, *J* = 11.4, 4.5 Hz, H-6′b), 3.60 (ddd, 1H, *J* = 7.7, 4.5, 1.0 Hz, H-5′), 3.57 (dd, 1H, *J* = 9.9, 7.5 Hz, H-2′), 3.50 (dd, 1H, *J* = 9.9, 3.3 Hz, H-3′), 3.42 (s, 3H, O*Me*). ^13^C­{^1^H} NMR (CD_3_OD, 101 MHz, HSQC, HMBC): δ 151.1 (*C*_q(Py)_), 150.5 (*C*H_Py_), 148.1 (*C*_q(Tria)_), 138.9 (*C*H_Py_), 125.4 (*C*H_Tria_), 124.5, 121.5 (2 × *C*H_Py_), 105.3 (C-1′), 102.7 (C-1), 81.0 (C-4), 77.2 (C-5), 76.8 (C-5′), 74.8 (C-3′), 74.1 (C-3), 72.6 (C-2′), 70.3 (C-4′), 67.9 (C-2), 62.5 (C-6′), 61.7 (C-6), 57.4 (O*Me*). HRMS-ESI (*m*/*z*): [M + H]^+^ calculated for C_20_H_29_N_4_O_10_, 485.1878; found 485.1877.

#### Complex **12-Cl**

Complex **12-Cl** was prepared according to the general complexation procedure starting from dichloro­(*p*-cymene)­ruthenium­(II) dimer (20 mg, 0.033 mmol) in DCM (1 mL) and the precursor **S3** (31 mg, 0.064 mmol) in a 1:1 MeOH/DCM mixture (2 mL). The product **12-Cl** (43 mg, 85%) precipitated from MeOH solution by addition of methyl *tert*-butyl ether as a yellow solid mixture of two diastereomers (denoted here as “a” and “b”) in a ratio of 3:2. ^1^H NMR (CD_3_OD, 400 MHz, H–H COSY): δ 9.41–9.40 (m, 2 × 1H, C*H*_Py_), 9.06 (2 × s, 2 × 1H, C*H*_Tria_), 8.19–8.14, 8.11–8.08, 7.68–7.63 (3 × m, 3 × 2 × 1H, C*H*_Py_), 6.20–6.14, 6.02–6.01, 5.97–5.93, 5.79–5.76 (4 × m, 4 × 2H, C*H*_*p*‑cym_), 5.18 (d, 1H, *J* = 8.3 Hz, H-1a), 4.99–4.97 (m, 1H, H-1b), 4.55–4.50 (m, 3H, H-2a, H-2b, H-3b), 4.47 (d, 1H, *J* = 7.6 Hz, H-1′a/b), 4.46 (d, 1H, *J* = 7.5 Hz, H-1′a/b), 4.36 (dd, 1H, *J* = 10.6, 8.2 Hz, H-3a), 4.06–3.97 (m, 4H, H-6aa, H-6ab, H-6ba, H-6bb), 3.90–3.81 (m, 4H, H-4a, H-4b, H-4′a, H-4′b), 3.79–3.50 (m, 12H, H-5a, H-5b, H-2′a, H-2′b, H-3′a, H-3′b, H-5′a, H-5′b, H-6′aa, H-6′ba, H-6′ab, H-6′bb), 3.48, 3.41 (2 × s, 2 × 3H, O*Me*), 2.76–2.65 (m, 2H, C*H*_*i*‑Pr_), 2.25, 2.23 (2 × s, 2 × 3H, *Me*_*p*‑cym_), 1.17, 1.15, 1.05, 1.01 (4 × d, 4 × 3H, *J* = 6.9 Hz, *Me*_*i*‑Pr_). ^13^C­{^1^H} NMR (CD_3_OD, 101 MHz, HSQC, HMBC, HSQC TOCSY): δ 156.8, 156.7 (2 × C*H*_Py_), 149.72, 149.67 (2 × *C*_q(Py)_), 147.6, 147.5 (2 × *C*_q(Tria)_), 141.4, 127.4 (2 × 2*C*H_Py_), 127.3, 127.2 (2 × *C*H_Tria_), 123.56, 123.55 (2 × *C*H_Py_), 106.4, 106.0 (2 × *C*_q_CH_*i*‑Pr_), 105.4, 105.3 (C-1′a, C-1′b), 104.8, 104.2 (2 × *C*_q_Me), 102.2 (C-1b), 101.9 (C-1a), 87.3 (2 × *C*H_*p*‑cym_), 86.1, 86.0, 85.4, 85.2, 84.6, 84.3 (6 × *C*H_*p*‑cym_), 81.0 (C-4a), 80.9 (C-4b), 77.2, 77.1 (C-5′a, C-5′b), 76.9 (C-5a, C-5b), 74.8 (C-3′a, C-3′b), 74.0 (C-3a), 73.5 C-3b), 72.61, 72.59 (C-2′a, C-2′b), 70.23, 70.20 (C-4′a, C-4′b), 69.80, 69.78 (C-2a, C-2b), 62.5 (C-6′a, C-6′b), 61.6, 61.5 (C-6a, C-6b), 57.6 (O*Me*-b), 57.4 (O*Me*-a), 32.29, 32.27 (2 × *C*H_*i*‑Pr_), 22.64, 22.56, 21.84, 21.80 (2 × *Me*_*i*‑Pr_), 18.9, 18.8 (2 × *Me*_*p*‑cym_). HRMS-APCI (*m*/*z*): [M – Cl]^+^ calculated for C_30_H_42_N_4_O_10_Cl Ru, 755.1633; found 755.1632.

#### Methyl 4-*O*-{3-[4-(Pyridin-2-yl)-1*H*-1,2,3-triazol-1-yl]-3-deoxy-β-d-galactopyranosyl}-2-acetamido-2-deoxy-β-d-glucopyranoside (**19**)

Ac_2_O (5.0 mL, 52.9 mmol) was added to a solution of methyl 4-*O*-(3-azido-3-deoxy-β-d-galactopyranosyl)-2-acetamido-2-deoxy-β-d-glucopyranoside^36^ (135 mg, 0.32 mmol) in pyridine (7 mL), and the mixture was stirred at room temperature overnight. The reaction mixture was concentrated, and the residual volatiles were removed by coevaporation with toluene (3×). The residue was then dissolved in DMF (2 mL), copper­(II) sulfate pentahydrate (12 mg, 0.05 mmol), ascorbic acid (8 mg, 0.05 mmol), and 2-ethynylpyridine (39 μL, 0.39 mmol) were added, and the reaction mixture was stirred at 40 °C for 2 h. The reaction mixture was allowed to cool to rt, and then it was diluted with DCM (30 mL) and washed with water (30 mL); the aqueous phase was extracted twice with DCM (20 mL). The combined organic phases were dried and concentrated. Column chromatography in EtOAc provided acetylated intermediate **S4** (209 mg, 89%, see the Supporting Information, Scheme S3, for the structure) as a white amorphous solid, *R*_*f*_ 0.35 (EtOAc). ^1^H NMR (CDCl_3_, 400 MHz, H–H COSY): δ 8.54 (d, 1H, *J* = 4.7 Hz, C*H*_Py_), 8.22 (s, 1H, C*H*_Tria_), 8.09 (d, 1H, *J* = 7.9 Hz, C*H*_Py_), 7.74 (dd, 1H, *J* = 7.9, 7.6 Hz, C*H*_Py_), 7.20 (dd, 1H, *J* = 7.6, 4.7 Hz, C*H*_Py_), 5.95 (d, 1H, *J* = 9.4 Hz, N*H*), 5.64 (dd, 1H, *J* = 11.5, 7.6 Hz, H-2′), 5.52 (d, 1H, *J* = 3.3 Hz, H-4′), 5.16 (dd, 1H, *J* = 11.5, 3.3 Hz, H-3′), 5.12 (dd, 1H, *J* = 9.4, 8.6 Hz, H-3), 4.70 (d, 1H, *J* = 7.6 Hz, H-1′), 4.50 (dd, 1H, *J* = 11.9, 2.7 Hz, H-6a), 4.40 (d, 1H, *J* = 7.6 Hz, H-1), 4.17 (dd, 1H, *J* = 11.9, 5.2 Hz, H-6b), 4.13 (br s, 3H, H-5′, H-6′a, 6′b), 4.07 (td, 1H, *J* = 9.4, 7.6 Hz, H-2), 3.85 (dd, 1H, *J* = 9.0, 8.6 Hz, H-4), 3.67 (ddd, 1H, *J* = 9.0, 5.2, 2.7 Hz, H-5), 3.45 (s, 3H, O*Me*), 2.11, 2.09, 2.08, 2.05, 1.97, 1.87 (6 × s, 6 × 3H, *Me*_Ac_). ^13^C­{^1^H} NMR (CDCl_3_, 101 MHz, HSQC, HMBC): δ 170.9, 170.6, 170.54, 170.46 (4 × *C*O), 169.2 (2*C*O), 149.8 (*C*_q_), 149.6 (*C*H_Py_), 148.7 (*C*_q(Tria)_), 137.0, 123.2 (2 × *C*H_Py_), 121.6 (*C*H_Tria_), 120.3 (*C*H_Py_), 101.9 (C-1), 101.4 (C-1′), 75.8 (C-4), 72.7 (C-5), 72.5 (C-3), 72.1 (C-5′), 68.3/68.2.6 (C-2′/4′), 62.5 (C-6), 62.1 (C-3′), 61.2 (C-6′), 56.8 (O*Me*), 53.2 (C-2), 23.4, 21.00, 20.97, 20.8, 20.5, 20.4 (6 × *Me*_Ac_). HRMS-ESI (*m*/*z*): [M + H]^+^ calculated for C_32_H_42_N_5_O_15_, 736.2672; found 736.2669.

Compound **19** was prepared according to the general procedure for deacetylation starting from intermediate **S4** (209 mg, 0.28 mmol). The reaction mixture was stirred at room temperature overnight. Neutralization with acidic ion-exchange resin DOVEX 50W­(H^+^), filtration, and solvent evaporation yielded compound **19** (149 mg, quantitative yield for this step, 89% overall yield over both steps) as a yellowish amorphous solid, *R*_*f*_ 0.35 (DCM/MeOH 3:1), ${[\alpha ]}_{D}^{20}$ −11 (*c* 0.42, MeOH). ^1^H NMR (CD_3_OD, 400 MHz, COSY): δ 8.56 (br s, 2H, C*H*_Py_, C*H*_Tria_), 8.10 (d, 1H, *J* = 6.0 Hz, C*H*_Py_), 7.93 (dd, 1H, *J* = 6.0, 8.4 Hz, C*H*_Py_), 7.38 (br s, 1H, C*H*_Py_), 4.90 (dd, 1H, *J* = 11.1, 3.2 Hz, H-3′), 4.68 (d, 1H, *J* = 7.5 Hz, H-1′), 4.35 (d, 1H, *J* = 8.2 Hz, H-1), 4.22 (dd, 1H, *J* = 11.1, 7.5 Hz, H-2′), 4.09 (d, 1H, *J* = 3.1 Hz, H-4′), 3.95 (dd, 1H, *J* = 12.2, 2.5 Hz, H-6a), 3.91–3.87 (m, 2H, H-6b, H-5′), 3.80 (dd, 1H, *J* = 11.4, 7.1 Hz, H-6′a), 3.76 (dd, 1H, *J* = 10.4, 8.2 Hz, H-2), 3.75–3.64 (m, 3H, H-3, H-4, H-6′b), 3.47 (s, 3H, O*Me*), 3.43 (ddd, 1H, *J* = 9.4, 4.2, 2.5 Hz, H-5), 1.98 (s, 3H, *Me*_Ac_). ^13^C­{^1^H} NMR (CD_3_OD, 101 MHz, HSQC, HMBC): δ 173.6 (*C*O), from HMBC 151.3 (*C*_q(Py)_), 150.4 (*C*H_Py_), from HMBC 148.1 (*C*_q(Tria)_), 139.0 (*C*H_Py_), from HSQC 124.4 (*C*H_Py_), 123.8 (*C*H_Tria_), from HSQC 121.7 (*C*H_Py_), 105.3 (C-1′), 103.6 (C-1), 80.7 (C-4), 77.9 (C-5′), 76.6 (C-5), 74.4 (C-3), 69.5/69.4 (C-2′/4′), 67.7 (C-3′), 62.3 (C-6′), 61.7 (C-6), 57.1 (O*Me*), 56.6 (C-2), 22.9 (*Me*_Ac_). HRMS-ESI (*m*/*z*): [M + Na]^+^ calculated for C_22_H_31_N_5_O_10_Na, 548.1963; found 548.1971.

#### Complex **13-Cl**

Complex **13-Cl** was prepared according to the general complexation procedure starting from dichloro­(*p*-cymene)­ruthenium­(II) dimer (18 mg, 0.029 mmol) in DCM (1 mL) and disaccharide **19** (31 mg, 0.059 mmol) in a 1:1 MeOH/DCM mixture (2 mL). The product **13-Cl** (42 mg, 86%) precipitated from the MeOH solution by the addition of methyl *tert*-butyl ether as a yellow solid mixture of two diastereomers in a ratio of 1:0.6. Major diastereomer: ^1^H NMR (CD_3_OD, 400 MHz, COSY): δ 9.42–9.40 (m, 1H, C*H*_Py_), 9.13 (s, 1H, C*H*_Tria_), 8.20–8.14 (m, 2H, C*H*_Py_), 7.68–7.64 (m, 1H, C*H*_Py_), 6.12, 6.03, 5.92, 5.81 (4 × m, 4 × 1H, C*H*_*p*‑cym_), 5.16 (dd, 1H, *J* = 11.1, 3.1 Hz, H-3′), 4.74 (d, 1H, *J* = 7.6 Hz, H-1′), 4.38 (d, 1H, *J* = 8.2 Hz, H-1), 4.27 (dd, 1H, *J* = 11.1, 7.6 Hz, H-2′), 4.15 (dd, 1H, *J* = 3.1, 1.1 Hz, H-4′), 3.97–3.88 (m, 3H, H-6a, H-6b, H-5′), 3.85–3.65 (m, 5H, H-2, H-3, H-4, H-6′a, H-6′b), 3.47 (s, 3H, O*Me*), 3.45 (dt, 1H, *J* = 9.6, 3.3 Hz, H-5), 2.78–2.71 (m, 1H, C*H*_*i*‑Pr_), 2.23 (s, 3H, *Me*_*p*‑cym_), 1.99 (s, 3H, *Me*_Ac_), 1.16, 1.06 (2 × d, 2 × 3H, *J* = 6.9 Hz, *Me*_*i*‑Pr_). ^13^C­{^1^H} NMR (CD_3_OD, 101 MHz, HSQC, HMBC, HSQC TOCSY): δ 172.3 (*C*O), 155.32 (C*H*_py_), 148.60 (*C*_q(Py)_), 146.3 (*C*_q(Tria)_), 140.01, 125.9 (2 × *C*H_Py_), 124.5 (*C*H_Tria_), 122.0 (*C*H_Py_), 105.02 (*C*_q_CH_*i*‑Pr_), 103.68 (C-1′), 102.53 (*C*_q_Me), 102.15 (C-1), 85.9, 84.5, 84.1, 83.3 (4 × *C*H_*p*‑cym_), 79.3 (C-4), 76.4 (C-5′), 75.18 (C-5), 72.9 (C-3), 68.2 (C-3′), 67.9 (C-2′), 67.7 (C-4′), 60.73 (C-6′), 60.4 (C-6), 55.7 (O*Me*), 55.5 (C-2), 30.8 (*C*H_*i*‑Pr_), 21.5 (*Me*_Ac_), 21.2, 20.4 (2 × *Me*_*i*‑Pr_), 17.3 (*Me*_*p*‑cym_). Minor diastereomer: ^1^H NMR (CD_3_OD, 400 MHz, H–H COSY): δ 9.42–9.40 (m, 1H, C*H*_Py_), 9.09 (s, 1H, C*H*_Tria_), 8.20–8.14 (m, 2H, C*H*_Py_), 7.68–7.64 (m, 1H, C*H*_Py_), 6.11, 6.06, 5.89, 5.83 (4 × m, 4 × 1H, C*H*_*p*‑cym_), 5.09 (dd, 1H, *J* = 11.0, 2.9 Hz, H-3′), 4.73 (d, 1H, *J* = 7.5 Hz, H-1′), 4.37 (d, 1H, *J* = 8.2 Hz, H-1), 4.34 (dd, 1H, *J* = 11.0, 7.5 Hz, H-2′), 4.20 (dd, 1H, *J* = 2.9, 1.1 Hz, H-4′), 3.97–3.88 (m, 3H, H-6a, H-6b, H-5′), 3.85–3.65 (m, 5H, H-2, H-3, H-4, H-6′a, H-6′b), 3.47 (s, 3H, O*Me*), 3.44 (dt, 1H, *J* = 9.6, 3.3 Hz, H-5), 2.78–2.71 (m, 1H, C*H*_*i*‑Pr_), 2.22 (s, 3H, *Me*_*p*‑cym_), 1.99 (s, 3H, *Me*_Ac_), 1.15, 1.09 (2 × d, 2 × 3H, *J* = 6.9 Hz, *Me*_*i*‑Pr_). ^13^C­{^1^H} NMR (CD_3_OD, 101 MHz, HSQC, HMBC, HSQC TOCSY): δ 172.3 (*C*O), 155.29 (C*H*_Py_), 148.63 (*C*_q(Py)_), 146.2 (*C*_q(Tria)_), 140.00, 125.9 (2 × *C*H_Py_), 124.8 (*C*H_Tria_), 122.0 (*C*H_Py_), 105.01 (*C*_q_CH_*i*‑Pr_), 103.70 (C-1′), 102.46 (*C*_q_Me), 102.16 (C-1), 86.0, 84.6, 84.0, 83.2 (4 × *C*H_*p*‑cym_), 79.2 (C-4), 76.2 (C-5′), 75.20 (C-5), 73.0 (C-3), 68.5 (C-3′), 68.0 (C-4′), 67.5 (C-2′), 60.69 (C-6′), 60.3 (C-6), 55.7 (O*Me*), 55.4 (C-2), 30.9 (*C*H_*i*‑Pr_), 21.5 (*Me*_Ac_), 21.2, 20.5 (2 × *Me*_*i*‑Pr_), 17.4 (*Me*_*p*‑cym_). HRMS-ESI (*m*/*z*): [M – Cl]^+^ calculated for C_32_H_45_ClN_5_O_10_Ru, 796.1899; found 796.1904.

#### Complex **13-PF**

Complex **13-PF** was prepared according to the general procedure for counterion exchange starting from **13-Cl** (21 mg, 0.025 mmol) and AgPF_6_ (7 mg, 0.028 mmol). The product **13-PF** precipitated by the slow addition of methyl *tert*-butyl ether as a yellow solid (20 mg, 84%) mixture of two diastereomers in a ratio of 1:0.6. Major diastereomer: ^1^H NMR (DMSO-*d*_6_, 400 MHz, COSY): δ 9.47–9.46 (m, 1H, C*H*_Py_), 9.39 (s, 1H, C*H*_Tria_), 8.30–8.22 (m, 2H, C*H*_Py_), 7.84 (d, 1H, *J* = 7.5 Hz, N*H*), 7.70–7.66 (m, 1H, C*H*_Py_), 6.14, 6.07, 6.02, 5.86 (4 × m, 4 × 1H, C*H*_*p*‑cym_), 5.86 (d, 1H, *J* = 6.2 Hz, O*H*-2′), 5.73 (d, 1H, *J* = 6.5 Hz, O*H*-4′), 5.18 (dd, 1H, *J* = 11.0, 3.1 Hz, H-3′), 4.89 (t, 1H, *J* = 5.0 Hz, O*H*-6′), 4.76 (t, 1H, *J* = 6.2 Hz, O*H*-6), 4.67 (d, 1H, *J* = 7.5 Hz, H-1′), 4.61 (d, 1H, *J* = 2.1 Hz, O*H*-3), 4.28 (d, 1H, *J* = 7.6 Hz, H-1), 4.14–4.04 (m, 1H, H-2′), 3.98 (dd, 1H, *J* = 6.5, 3.1 Hz, H-4′), 3.85 (t, 1H, *J* = 5.6 Hz, H-5′), 3.80–3.76 (m, 1H, H-6a), 3.71–3.65 (m, 1H, H-6b), 3.60–3.46 (m, 5H, H-2, H-3, H-4, H-6′a, H-6′b), 3.35 (s, 3H, O*Me*), 3.33–3.29 (m, 1H, H-5), 2.70–2.61 (m, 1H, C*H*_*i*‑Pr_), 2.14 (s, 3H, *Me*_*p*‑cym_), 1.81 (s, 3H, *Me*_Ac_), 1.07, 0.93 (2 × d, 2 × 3H, *J* = 6.9 Hz, *Me*_*i*‑Pr_). ^13^C­{^1^H} NMR (DMSO-*d*_6_, 101 MHz, HSQC, HMBC, HSQC TOCSY): δ 168.9 (*C*O), 155.7 (C*H*_py_), 148.1 (*C*_q(Py)_), 145.6 (*C*_q(Tria)_), 140.2, 125.9 (2 × *C*H_Py_), 125.4 (*C*H_Tria_), 122.0 (*C*H_Py_), 104.1 (*C*_q_CH_*i*‑Pr_), 103.5 (C-1′), 102.0 (C-1), 101.7 (*C*_q_Me), 85.5, 84.5, 83.8, 82.9 (4 × *C*H_*p*‑cym_), 80.7 (C-4), 76.0 (C-5′), 75.0 (C-5), 72.4 (C-3), 67.61/67.56 (C-2′/3′), 66.8 (C-4′), 60.2 (C-6), 60.0 (C-6′), 55.8 (O*Me*), 54.7 (C-2), 30.4 (*C*H_*i*‑Pr_), 23.1 (*Me*_Ac_), 22.0, 21.2 (2 × *Me*_*i*‑Pr_), 18.1 (*Me*_*p*‑cym_). ^19^F NMR (DMSO-*d*_6_, 376 MHz): δ −71.43 (d, ^1^ *J*_(F–P)_ = 711.4 Hz). ^31^P NMR (DMSO-*d*_6_, 162 MHz): δ −161.10 (hept, ^1^ *J*_(P–F)_ = 711.4 Hz). Minor diastereomer: ^1^H NMR (DMSO-*d*_6_, 400 MHz, COSY): δ 9.47–9.46 (m, 1H, C*H*_Py_), 9.37 (s, 1H, C*H*_Tria_), 8.30–8.22 (m, 2H, C*H*_Py_), 7.84 (d, 1H, *J* = 7.5 Hz, N*H*), 7.70–7.66 (m, 1H, C*H*_Py_), 6.17, 6.13, 5.98, 5.92 (4 × m, 4 × 1H, C*H*_*p*‑cym_), 5.86 (d, 1H, *J* = 6.2 Hz, O*H*-2′), 5.65 (d, 1H, *J* = 6.4 Hz, O*H*-4′), 5.15 (dd, 1H, *J* = 11.2, 3.0 Hz, H-3′), 4.96 (t, 1H, *J* = 4.9 Hz, O*H*-6′), 4.75 (dd, 1H, *J* = 6.2, 4.1 Hz, O*H*-6), 4.67 (d, 1H, *J* = 7.5 Hz, H-1′), 4.60 (d, 1H, *J* = 2.0 Hz, O*H*-3), 4.27 (d, 1H, *J* = 7.3 Hz, H-1), 4.14–4.04 (m, 2H, H-2′, H-4′), 3.86 (dd, 1H, *J* = 5.9, 4.7 Hz, H-5′), 3.80–3.76 (m, 1H, H-6a), 3.71–3.65 (m, 1H, H-6b), 3.60–3.46 (m, 5H, H-2, H-3, H-4, H-6′a, H-6′b), 3.35 (s, 3H, O*Me*), 3.33–3.29 (m, 1H, H-5), 2.70–2.61 (m, 1H, C*H*_*i*‑Pr_), 2.12 (s, 3H, *Me*_*p*‑cym_), 1.81 (s, 3H, *Me*_Ac_), 1.07, 1.00 (2 × d, 2 × 3H, *J* = 6.9 Hz, *Me*_*i*‑Pr_). ^13^C­{^1^H} NMR (DMSO-*d*_6_, 101 MHz, HSQC, HMBC, HSQC TOCSY): δ 168.9 (*C*O), 155.7 (C*H*_Py_), 148.2 (*C*_q(Py)_), 145.3 (*C*_q(Tria)_), 140.2, 125.9 (2 × *C*H_Py_), 125.4 (*C*H_Tria_), 121.9 (*C*H_Py_), 104.0 (*C*_q_CH_*i*‑Pr_), 103.4 (C-1′), 101.9 (C-1), 101.7 (*C*_q_Me), 85.7, 85.6, 83.6, 82.8 (4 × *C*H_*p*‑cym_), 80.5 (C-4), 75.7 (C-5′), 75.0 (C-5), 72.4 (C-3), 67.61/67.56 (C-2′/3′), 67.0 (C-4′), 60.2 (C-6), 59.7 (C-6′), 55.8 (O*Me*), 54.7 (C-2), 30.4 (*C*H_*i*‑Pr_), 23.1 (*Me*_Ac_), 22.0, 21.3 (2 × *Me*_*i*‑Pr_), 18.1 (*Me*_*p*‑cym_). ^19^F NMR (DMSO-*d*_6_, 376 MHz): δ −71.43 (d, ^1^ *J*_(F–P)_ = 711.4 Hz). ^31^P NMR (DMSO-*d*_6_, 162 MHz): δ −161.10 (hept, ^1^ *J*_(P–F)_ = 711.4 Hz). HRMS-ESI (*m*/*z*): [M – PF_6_]^+^ calculated for C_32_H_45_ClN_5_O_10_Ru, 796.1899; found 796.1897.

#### Methyl 4-*O*-(2,4,6-Tri-*O*-acetyl-3-azido-3-deoxy-β-d-galactopyranosyl)-2-azido-3,6-di-*O*-benzyl-2-deoxy-β-d-glucopyranoside (**S5**)

Methyl 2-azido-3,6-di-*O*-benzyl-2-deoxy-β-d-glucopyranoside^53^ (255 mg, 0.64 mmol) and phenyl 2,4,6-tri-*O*-acetyl-3-azido-3-deoxy-1-thio-β-d-galactopyranoside^88^ (324 mg, 0.77 mmol) were codistilled with toluene (3×) and dried under vacuum using an oil pump for 15 min. Anhydrous dichloromethane (6 mL) and 3 Å molecular sieves (1 g) were added under an argon atmosphere, and the mixture was stirred at room temperature for 1 h and then cooled to 0 °C. *N*-Iodosuccinimide (314 mg, 1.40 mmol) and TfOH (20 μL, 0.23 mmol, added dropwise) were added, and the mixture was stirred at 0 °C for 1 h. The reaction mixture was diluted with DCM (30 mL); a saturated solution of NaHCO_3_ was added to quench the reaction and neutralize an excess of acid, and a 10% solution of Na_2_S_2_O_3_ (5 mL) was added to decolorize the reaction mixture. The reaction mixture was filtered and washed with water (30 mL). The aqueous phase was extracted with dichloromethane (2 × 30 mL). The combined organic phases were dried and concentrated. Column chromatography in EtOAc/PE 1:2 provided compound **S5** (390 mg, 86%, see the Supporting Information, Scheme S4, for the structure) as a colorless gel. *R*_*f*_ 0.30 (EtOAc/PE 1:2), ${[\alpha ]}_{D}^{20}$ −36 (*c* 0.60, CHCl_3_). ^1^H NMR (CDCl_3_, 400 MHz, H–H COSY): δ 7.42–7.27 (m, 10H, C*H*_Ph_), 5.27 (dd, 1H, *J* = 3.4, 1.2 Hz, H-4′), 5.03 (dd, 1H, *J* = 10.6, 7.9 Hz, H-2′), 4.95 (d, 1H, *J* = 10.6 Hz, CH*H* O-3Bn), 4.81 (d, 1H, *J* = 12.1 Hz, CH*H* O-6Bn), 4.73 (d, 1H, *J* = 10.6 Hz, CH*H* O-3Bn), 4.49 (d, 1H, *J* = 7.9 Hz, H-1′), 4.44 (d, 1H, *J* = 12.1 Hz, CH*H* O-6Bn), 4.13–4.11 (m, 1H, H-1), 3.99 (dd, 1H, *J* = 9.8, 8.9 Hz, H-4), 3.97 (dd, 1H, *J* = 11.3, 7.6 Hz, H-6′a), 3.80 (dd, 1H, *J* = 11.3, 6.1 Hz, H-6′b), 3.79 (dd, 1H, *J* = 10.9, 3.1 Hz, H-6a), 3.72 (dd, 1H, *J* = 10.9, 1.8 Hz, H-6b), 3.56 (s, 3H, *M*eO), 3.52 (dd, 1H, *J* = 7.6, 6.1, 1.2 Hz, H-5′), 3.41–3.35 (m, 2H, H-2, H-3), 3.31 (ddd, 1H, *J* = 9.8, 3.1, 1.8 Hz, H-5), 3.18 (dd, 1H, *J* = 10.6, 3.4 Hz, H-3′), 2.10, 2.06, 2.01 (3 × s, 3 × 3H, 3 × *M*e_Ac_). ^13^C­{^1^H} NMR (CDCl_3_, 101 MHz, HSQC, HMBC): δ 170.4, 170.0, 169.2 (3 × *C*O), 138.3, 138.0 (2 × *C*_q_), 128.8, 128.5, 128.33 (3 × 2*C*H_Ph_), 128.28 (*C*H_Ph_), 128.0 (2*C*H_Ph_), 127.9 (*C*H_Ph_), 103.1 (C-1), 100.3 (C-1′), 81.1 (C-3), 76.3 (C-4), 75.3 (*C*H_2_ O-3Bn), 74.9 (C-5), 73.9 (*C*H_2_ O-6Bn), 71.7 (C-5′), 70.3 (C-2′), 67.5 (C-6/4′), 65.8 (C-2), 61.7 (C-3′), 60.9 (C-6′), 57.3 (*Me*O), 20.9, 20.8, 20.7 (3 × *Me*_Ac_). HRMS-ESI (*m*/*z*): [M + Na]^+^ calculated for C_33_H_40_N_6_O_12_Na, 735.2596; found 735.2594.

#### Methyl 4-*O*-(2,4,6-Tri-*O*-acetyl-3-azido-3-deoxy-β-d-galactopyranosyl)-2-azido-2-deoxy-β-d-glucopyranoside (**2,3́′-diN_3_-Lacβ-OMe,** **S6**)

A solution of Na_2_S_2_O_4_ (454 mg, 2.61 mmol) in water (15 mL) was slowly added dropwise to a mixture of **S5** (380 mg, 0.53 mmol) and NaBrO_3_ (393 mg, 2.61 mmol) in EtOAc (20 mL) and water (10 mL).^89^ The mixture was stirred vigorously for 6 h. A change of color from colorless to orange-red and back to colorless occurred, indicating the end of the reaction. The mixture was diluted with DCM (100 mL) and washed with water (60 mL). The aqueous phase was extracted four times with DCM (50 mL). The combined organic phases were dried and concentrated. Column chromatography in EtOAc/PE 2:1 provided **S6** (255 mg, 90%, see the Supporting Information, Scheme S4, for the structure) as a colorless gel. *R*_*f*_ 0.15 (EtOAc/PE 1:1), ${[\alpha ]}_{D}^{20}$ +2 (*c* 0.81, CHCl_3_). ^1^H NMR (CDCl_3_, 400 MHz, H–H COSY): δ 5.41 (dd, 1H, *J* = 3.4, 1.0 Hz, H-4′), 5.20 (dd, 1H, *J* = 10.6, 7.9 Hz, H-2′), 4.61 (d, 1H, *J* = 7.9 Hz, H-1′), 4.28 (d, 1H, *J* = 1.5 Hz, O*H*-3), 4.26–4.20 (m, 1H, H-6′a), 4.18 (d, 1H, *J* = 8.2 Hz, H-1), 4.03–3.97 (m, 2H, H-5′, H-6′b), 3.84 (dd, 1H, *J* = 12.3, 2.1 Hz, H-6a), 3.68–3.61 (m, 4H, H-3, H-4, H-6b, O*H*-6), 3.61 (dd, 1H, *J* = 10.6, 3.4 Hz, H-3′), 3.56 (s, 3H, *M*eO), 3.35–3.32 (m, 1H, H-5), 3.29–3.24 (m, 1H, H-2), 2.18 (s, 2 × 3H, 2 × *M*e_Ac_), 2.11 (s, 3H, *M*e_Ac_). ^13^C­{^1^H} NMR (CDCl_3_, 101 MHz, HSQC, HMBC): δ 170.7 (*C*O_O‑6′Ac_), 170.0 (*C*O_O‑4′Ac_), 169.6 (*C*O_O‑2′Ac_), 102.6 (C-1), 102.2 (C-1′), 81.2 (C-4), 74.3 (C-3), 74.0 (C-5), 72.6 (C-5′), 69.8 (C-2′), 67.7 (C-4′), 65.5 (C-2), 62.2 (C-6), 61.8 (C-3′), 60.8 (C-6′), 57.6 (*Me*O), 20.8, 20.71, 20.68 (3 × *Me*_Ac_). HRMS-ESI (*m*/*z*): [M + Na]^+^ calcd for C_19_H_28_N_6_O_12_Na, 555.1657; found 555.1659.

#### Methyl 2-Deoxy-4-*O*-{3-deoxy-3-[4-(pyridin-2-yl)-1*H*-1,2,3-triazol-1-yl]-β-d-galactopyranosyl}-2-[4-(pyridin-2-yl)-1*H*-1,2,3-triazol-1-yl]-β-d-glucopyranoside (**20**)

Copper­(II) sulfate pentahydrate (12 mg, 0.05 mmol), ascorbic acid (8 mg, 0.05 mmol), 2-ethynylpyridine (58 μL, 0.57 mmol), and disaccharide **S6** (125 mg, 0.23 mmol) reacted according to the general procedure for azide–alkyne cycloaddition. The acetyl-protected intermediate obtained after the aqueous workup and concentration was not chromatographically purified but immediately subjected to deacetylation according to the general procedure. Column chromatography in DCM/MeOH 5:1 afforded **20** (94 mg, 65% over two steps) as a colorless gel, *R*_f_ 0.35 (DCM/MeOH 5:1), ${[\alpha ]}_{D}^{20}$ −1 (*c* 0.78, MeOH). ^1^H NMR (DMSO-*d*_6_, 400 MHz, COSY): δ 8.81 (s, 1H, C*H*_Tria_), 8.61 (br s, 2H, C*H*_Py_), 8.44 (s, 1H, C*H*_Tria_), 8.05–8.03, 7.93–7.87, 7.37–7.32 (3 × m, 3 × 2H, C*H*_Py_), 4.98 (d, 1H, *J* = 8.3 Hz, H-1), 4.89 (dd, 1H, *J* = 11.0, 3.1 Hz, H-3′), 4.64 (d, 1H, *J* = 7.5 Hz, H-1′), 4.39 (dd, 1H, *J* = 10.4, 8.3 Hz, H-2), 4.24 (dd, 1H, *J* = 10.4, 7.8 Hz, H-3), 4.11 (dd, 1H, *J* = 11.0, 7.5 Hz, H-2′), 3.88–3.83 (m, 2H, H-6a, H-4′), 3.81 (dd, 1H, *J* = 6.4, 5.8 Hz, H-5′), 3.76 (dd, 1H, *J* = 11.9, 4.4 Hz, H-6b), 3.69 (dd, 1H, *J* = 10.0, 7.8 Hz, H-4), 3.69–3.64 (m, 1H, H-5), 3.51–3.49 (m, 2H, H-6′a, H-6′b), 3.31 (s, 3H, O*Me*). ^13^C­{^1^H} NMR (DMSO-*d*_6_, 101 MHz, HSQC, HMBC): δ 150.2, 150.0 (2 × *C*H_Py_), 149.63, 149.61 (2 × *C*_q(Py)_), 147.0, 146.6 (2 × *C*_q(Tria)_), 137.3, 137.2 (2 × *C*H_Py_), 123.5 (*C*H_Tria_), 123.0, 122.9 (2 × *C*H_Py_), 122.5 (*C*H_Tria_), 119.4, 119.3 (2 × *C*H_Py_), 103.9 (C-1′), 100.6 (C-1), 80.5 (C-4), 76.3 (C-5′), 75.2 (C-5), 72.4 (C-3), 67.5 (C-4′), 67.3 (C-2′), 65.8 (C-2), 65.6 (C-3′), 60.2 (C-6′), 60.0 (C-6), 56.3 (O*Me*). HRMS-ESI (*m*/*z*): [M + H]^+^ calculated for C_27_H_33_N_8_O_9_, 613.2365; found 613.2361.

#### 2,3′-Bisruthenium Complex **14-Cl**

Complex **14-Cl** was prepared according to the general complexation procedure starting from dichloro­(*p*-cymene)­ruthenium­(II) dimer (34 mg, 0.055 mmol) in DCM (1 mL) and disaccharide **20** (34 mg, 0.055 mmol) in a 1:1 mixture of MeOH/DCM (2 mL). The product **14-Cl** (62 mg, 91%) precipitated from the MeOH solution by the addition of methyl *tert*-butyl ether as a yellow solid mixture of four diastereomers. ^1^H NMR (CD_3_OD, 400 MHz, H–H COSY): δ 9.42–9.41 (m, 8 × 1H, Hz, C*H*_Py_), 9.16, 9.15, 9.12, 9.11, 9.09, 9.08 (7 × s, 8 × 1H, C*H*_Tria_), 8.20–8.10 (m, 8 × 2H, C*H*_Py_), 7.69–7.64 (m, 8 × 1H, C*H*_Py_), 6.22–6.11, 6.07–6.02, 5.98–5.89, 5.85–5.78 (4 × m, 4 × 8 × 1H, C*H*_*p*‑cym_), 5.23, 5.22 (2 × d, 2 × 1H, *J* = 8.3 Hz, H-1), 5.20–5.10 (m, 4 × 1H, H-3′), 5.03–5.01 (m, 2 × 1H, H-1), 4.83, 4.82, 4.81, 4.80 (4 × d, 4 × 1H, *J* = 7.5 Hz, H-1′), 4.60–4.57 (m, 2 × 1H, H-3), 4.60–4.54 (m, 4 × 1H, H-2), 4.46–4.40 (m, 2 × 1H, H-3), 4.41–4.29 (m, 4 × 1H, H-2′), 4.23, 4.21, 4.17, 4.16 (4 × dd, 4 × 1H, *J* = 3.1, 0.9 Hz, H-4′), 4.06–3.94 (m, 4 × 4H, H-4, H-6a, H-6b, H-5′), 3.84–3.68 (m, 3 × 4H, H-5, H-6′a, H-6′b), 3.50, 3.43 (2 × s, 4 × 3H, O*Me*), 2.79–2.66 (m, 8 × 1H, C*H*_*i*‑Pr_), 2.26, 2.25, 2.23, 2.22 (4 × s, 8 × 3H, *Me*_*p*‑cym_), 1.19–1.02 (m, 8 × 6H, *Me*_*i*‑Pr_). ^13^C­{^1^H} NMR (CD_3_OD, 101 MHz, HSQC, HMBC, HSQC TOCSY): δ 156.8, 156.7 (8 × C*H*_Py_), 150.0, 149.9, 149.8, 149.7 (8 × *C*_q(Py)_), 147.77, 147.75, 147.7, 147.62, 147.57 (8 × *C*_q(Tria)_), 141.4 (8 × *C*H_Py_), 127.44, 127.41, 127.37, 127.1, 126.2, 126.0 (8 × *C*H_Py_, 8 × *C*H_Tria_), 123.6, 123.51, 123.48 (8 × *C*H_Py_), 106.5, 106.42, 106.38, 106.1 (8 × *C*_q_CH_*i*‑Pr_), 105.40, 105.35, 105.3 (4 × C-1′), 104.2, 103.9, 103.8 (8 × *C*_q_Me), 102.2, 101.9 (4 × C-1), 87.50, 87.45, 87.4, 87.3 (8 × *C*H_*p*‑cym_), 86.14, 86.05, 86.0, 85.9 (8 × *C*H_*p*‑cym_), 85.6, 85.4, 85.2 (8 × *C*H_*p*‑cym_), 84.7, 84.6, 84.3 (8 × *C*H_*p*‑cym_), 80.9 (4 × C-4), 77.8, 77.7, 77.6 (4 × C-5′), 76.8 (4 × C-5), 74.0, 73.4 (4 × C-3), 70.0, 69.91, 69.85 (4 × C-2), 69.7 (C-3′), 69.33, 69.29, 69.0 (4 × C-2′, 4 × C-4′), 62.1 (4 × C-6′), 61.5, 61.4 (4 × C-6), 57.6, 57.4 (4 × O*Me*), 32.3, 32.2 (8 × *C*H_*i*‑Pr_), 22.63, 22.57, 21.91, 21.85, 21.8 (8 × *Me*_*i*‑Pr_), 18.9, 18.78, 18.75 (8 × *Me*_*p*‑cym_). HRMS-ESI (*m*/*z*): [M – 2Cl]^2+^ calculated for C_47_H_60_Cl_2_N_8_O_9_Ru_2_, 577.0975; found 577.0977.

#### 1,1′-Sulfanediyl-bis-{3-dideoxy-3-[4-(pyridin-2-yl)-1*H*-1,2,3-triazol-1-yl]-β-d-galactopyranoside} (**21**)

Copper­(II) sulfate pentahydrate (12 mg, 0.05 mmol), ascorbic acid (8 mg, 0.05 mmol), 2-ethynylpyridine (88 μL, 0.87 mmol), and 1,1′-sulfanediyl-bis­(2,4,6-tri-*O*-acetyl-3-azido-3-deoxy-β-d-galactopyranoside)^51^ (240 mg, 0.36 mmol) reacted according to the general procedure for azide–alkyne cycloaddition. Column chromatography (EtOAc → EtOAc/CH_3_OH 10:1) provided disaccharide **S7** (203 mg, 64%, see the Supporting Information, Scheme S5, for the structure) as a white amorphous solid, *R*_*f*_ 0.30 (EtOAc). ^1^H NMR (DMSO-*d*_6_, 400 MHz, COSY): δ 8.61 (ddd, 2H, *J* = 4.8, 1.8, 1.2 Hz, C*H*_Py_), 8.61 (s, 2H, C*H*_Tria_), 8.02 (dt, 2H, *J* = 7.9, 1.2 Hz, C*H*_Py_), 7.90 (ddd, 2H, *J* = 7.9, 7.6, 1.8 Hz, C*H*_Py_), 7.36 (ddd, 2H, *J* = 7.6, 4.8, 1.2 Hz, C*H*_Py_), 5.75–5.67 (m, 2 × 2H, H-2, H-3), 5.52 (dd, 2H, *J* = 2.8, 1.2 Hz, H-4), 5.25 (d, 2H, *J* = 9.6 Hz, H-1), 4.34 (ddd, 2H, *J* = 6.7, 5.9, 1.2 Hz, H-5), 4.14 (dd, 2H, *J* = 11.4, 6.7 Hz, H-6), 4.08 (dd, 2H, *J* = 11.4, 5.9 Hz, H-6′), 2.05, 2.02, 1.87 (3 × s, 3 × 6H, *Me*). ^13^C­{^1^H} NMR (DMSO-*d*_6_, 101 MHz, HSQC, HMBC): δ 170.0, 169.3, 169.0 (6 × *C*O), 149.7 (2 × *C*H_Py_), 149.5 (2 × *C*_q(Py)_), 147.3 (2 × *C*_q(Tria)_), 137.3, 123.3 (2 × 2*C*H_Py_), 122.9 (2 × *C*H_Tria_), 119.6 (2 × *C*H_Py_), 81.6 (2 × C-1), 74.6 (2 × C-5), 68.8 (2 × C-4), 66.8 (2 × C-2), 61.6 (2 × C-3), 61.4 (2 × C-6), 20.6, 20.3, 20.2 (6 × *Me*). HRMS-ESI (*m*/*z*): [M + Na]^+^ calculated for C_38_H_42_N_8_O_14_SNa, 889.2433; found 889.2437.

A 0.1 M solution of sodium methanolate in methanol was added dropwise to a solution of disaccharide **S7** (180 mg, 0.21 mmol) in a mixture of MeOH (10 mL) and DCM (10 mL) until pH = 9. The reaction mixture was stirred overnight at rt. It was neutralized with ion-exchange resin DOVEX 50W­(H^+^), filtered, and concentrated to yield **21** (126 mg, 99%, 63% over two steps) as a white amorphous solid, *R*_*f*_ 0.10 (CH_2_Cl_2_/CH_3_OH 7:1). ^1^H NMR (DMSO-*d*_6_, 400 MHz, COSY): δ 8.60 (dt, 2H, *J* = 4.8, 1.5 Hz, C*H*_Py_), 8.47 (s, 2H, C*H*_Tria_), 8.04 (dt, 2H, *J* = 8.0, 1.2 Hz, C*H*_Py_), 7.90 (ddd, 2H, *J* = 8.0, 7.6, 1.5 Hz, C*H*_Py_), 7.34 (ddd, 2H, *J* = 7.6, 4.8, 1.2 Hz, C*H*_Py_), 5.53 (d, 2H, *J* = 6.9 Hz, O*H*-2), 5.32 (d, 2H, *J* = 7.4 Hz, O*H*-4), 4.96 (d, 2H, *J* = 9.5 Hz, H-1), 4.92 (dd, 2H, *J* = 10.6, 3.1 Hz, H-3), 4.77 (br s, 2H, O*H*-6), 4.19 (ddd, 2H, *J* = 10.6, 9.5, 6.9 Hz, H-2), 3.99 (dd, 2H, *J* = 7.4, 3.1 Hz, H-4), 3.74 (t, 2H, *J* = 6.4 Hz, H-5), 3.56–3.54 (m, 2 × 2H, H-6, H-6′). ^13^C­{^1^H} NMR (, 101 MHz, HSQC, HMBC): δ 150.2 (2 × *C*_q(Py)_), 149.6 (2 × *C*H_Py_), 146.6 (2 × *C*_q(Tria)_), 137.2, 122.8 (2 × 2*C*H_Py_), 122.5 (2 × *C*H_Tria_), 119.3 (2 × *C*H_Py_), 83.5 (2 × C-1), 79.3 (2 × C-5), 67.6 (2 × C-4), 67.0 (2 × C-3), 66.9 (2 × C-2), 60.1 (2 × C-6). HRMS-ESI (*m*/*z*): [M + Na]^+^ calculated for C_26_H_30_N_8_O_8_SNa, 637.1800; found 637.1812.

#### Bisruthenium TDG Complex **15-Cl**

Complex **15-Cl** was prepared according to the general complexation procedure starting from dichloro­(*p*-cymene)­ruthenium­(II) dimer (39 mg, 0.064 mmol) in DCM (1 mL) and disaccharide **21** (39 mg, 0.063 mmol) in a 1:1 MeOH/DCM mixture (2 mL). The product **15-Cl** (52 mg, 67%) precipitated from MeOH solution by the addition of methyl *tert*-butyl ether as a yellow solid mixture of three diastereomers in approximately a 1:1:0.3 ratio. ^1^H NMR (CD_3_OD, 400 MHz, COSY): δ 9.42–9.41 (m, 6H, C*H*_Py_), 9.15, 9.14, 9.13, 9.12 (4 × s, 6H, C*H*_Tria_), 8.18–8.09 (m, 12H, C*H*_Py_), 7.67–7.63 (m, 6H, C*H*_Py_), 6.14–6.12, 6.08–6.03, 5.94–5.90, 5.85–5.81 (4 × m, 4 × 6H, C*H*_*p*‑cym_), 5.23, 5.22, 5.18 (3 × dd, 6H, *J* = 10.6, 2.9 Hz, H-3), 5.03, 5.02, 5.01 (3 × d, 6H, *J* = 9.6 Hz, H-1), 4.80–4.70 (m, 6H, H-2), 4.33, 4.32, 4.28, 4.27 (4d, 6H, *J* = 2.9 Hz, H-4), 3.98–3.93 (m, 6H, H-5), 3.88–3.72 (m, 12H, H-6, H-6′), 2.76 (hept, 6H, *J* = 6.8 Hz, C*H*_*i*‑Pr_), 2.23 (s, 9H, *Me*_*p*‑cym_), 2.21 (2 × s, 9H, *Me*_*p*‑cym_), 1.17, 1.16, 1.12, 1.07 (4 × d, 36H, *J* = 6.9 Hz, *Me*_*i*‑Pr_). ^13^C­{^1^H} NMR (CD_3_OD, 101 MHz, HSQC, HMBC, HSQC TOCSY): δ 156.8, 156.74, 156.70 (6 × C*H*_Py_), 150.0, 149.9 (6 × *C*_q(Py)_), 147.73, 147.71, 147.6 (3 × 2*C*_q(Tria)_), 141.5, 141.41, 141.35, 127.4, 127.3 (12 × *C*H_Py_), 126.0, 125.9, 125.72, 125.67 (6 × *C*H_Tria_), 123.49, 123.46, 123.4, 123.3 (6 × *C*H_Py_), 106.51, 106.47, 106.45 (6 ×*C*_q_CH_*i*‑Pr_), 103.9, 103.7, 103.6 (3 × 2*C*_q_Me), 87.5, 87.4, 87.3 (3 × 2*C*H_*p*‑cym_), 86.33, 86.29, 86.2 (3 × 2C-1), 86.02, 86.00, 85.9 (6 × *C*H_*p*‑cym_), 85.6, 85.44, 85.42 (6 × *C*H_*p*‑cym_), 84.8, 84.68, 84.65 (3 × 2*C*H_*p*‑cym_), 81.12, 81.07, 81.05 (3 × 2C-5), 71.03, 71.00, (6 × C-3), 69.58, 69.55, 69.4 (3 × 2C-4), 68.4, 68.19, 68.16 (6 × C-2), 62.53, 62.51 (6 × C-6), 32.32, 32.25 (6 × *C*H_*i*‑Pr_), 22.67, 22.66, 22.6 (3 × 2*Me*_*i*‑Pr_), 21.96, 21.95, 21.8 (6 × *Me*_*i*‑Pr_), 18.80, 18.77, 18.75 (3 × 2*Me*_*p*‑cym_). HRMS-ESI (*m*/*z*): [M – 2Cl]^2+^ calculated for C_46_H_58_Cl_2_N_8_O_8_Ru_2_S, 578.0782; found 578.0786.

#### Bisruthenium TDG Complex **15-BF**

Complex **15-BF** was prepared according to the general procedure for counterion exchange starting from complex **15-Cl** (59 mg, 0.048 mmol) and AgBF_4_ (19 mg, 0.098 mmol). The product **15-BF** precipitated by the slow addition of methyl *tert*-butyl ether as a yellow solid (38 mg, 59%) mixture of three diastereomers in a ratio of approximately 1.5:1:1. ^1^H NMR (CD_3_OD, 400 MHz, COSY): δ 9.43–9.41 (m, 3 × 2H, C*H*_Py_), 9.16, 9.14, 9.13, 9.12 (4 × s, 6H, C*H*_Tria_), 8.18–8.10 (m, 3 × 4H, C*H*_Py_), 7.67–7.64 (m, 3 × 2H, C*H*_Py_), 6.14–6.12, 6.08–6.03, 5.94–5.91, 5.86–5.82 (4 × m, 4 × 6H, C*H*_*p*‑cym_), 5.23 (2 × dd, 3H, *J* = 10.6, 2.9 Hz, H-3), 5.22, 5.18 (2 × dd, 3H, *J* = 10.6, 2.9 Hz, H-3), 5.03, 5.01, 5.00 (3 × d, 3 × 2H, *J* = 9.5 Hz, H-1), 4.77, 4.73, 4.72, 4.71 (4 × dd, 6H, *J* = 10.6, 9.5 Hz, H-2), 4.33, 4.32, 4.28, 4.27 (4 × d, 6H, *J* = 2.9 Hz, H-4), 3.98–3.93 (m, 6H, H-5), 3.88–3.73 (m, 12H, H-6, H-6′), 2.82–2.70 (m, 6H, C*H*_*i*‑Pr_), 2.24, 2.22. 2.21 (3 × s, 3 × 6H, *Me*_*p*‑cym_), 1.17, 1.16, 1.12, 1.11, 1.07 (5 × d, 3 × 12H, *J* = 6.9 Hz, *Me*_*i*‑Pr_). ^13^C­{^1^H} NMR (CD_3_OD, 101 MHz, HSQC, HMBC, HSQC TOCSY): δ 156.8, 156.74, 156.70 (3 × 2C*H*_Py_), 150.0, 149.9 (6*C*_q(Py)_), 147.73, 147.71, 147.6 (3 × 2*C*_q(Tria)_), 141.5, 141.42, 141.36 (6C*H*_Py_), 127.4, 127.3 (6C*H*_Py_), 126.02, 125.97, 125.74, 125.69 (6*C*H_Tria_), 123.54, 123.50, 123.4 (3 × 2C*H*_Py_), 106.51, 106.49, 106.47, 106.45 (6*C*_q_CH_*i*‑Pr_), 103.9, 103.70, 103.68, 103.6 (6*C*_q_Me), 87.5, 87.4, 87.3 (3 × 2*C*H_*p*‑cym_), 86.30, 86.27, 86.2 (6C-1), 86.03, 86.00, 85.9, 85.6, 85.4, 84.8, 84.68, 84.65 (18*C*H_*p*‑cym_), 81.12, 81.07, 81.05 (3 × 2C-5), 71.1, 71.0, 70.8 (3 × 2C-3), 69.6, 69.5, 69.4 (3 × 2C-4), 68.4, 68.20, 68.16 (3 × 2C-2), 62.5 (6C-6), 33.32, 33.26 (6*C*H_*i*‑Pr_), 22.7, 22.6, 22.0, 21.8 (6 × 2*Me*_*i*‑Pr_), 18.80, 18.78, 18.76 (3 × 2*Me*_*p*‑cym_). ^19^F NMR (MeOH-*d*_4_, 376 MHz): δ −155.45 (br s, ^11^BF_4_), −155.50 (br s, ^10^BF_4_). HRMS-ESI (*m*/*z*): [M – 2BF_4_]^2+^ calculated for C_46_H_58_Cl_2_N_8_O_8_Ru_2_S, 578.0782; found 578.0784.

#### Bisruthenium TDG Complex **15-PF**

Complex **15-PF** was prepared according to the general procedure for counterion exchange starting from complex **15-Cl** (48 mg, 0.039 mmol) and AgPF_6_ (21 mg, 0.083 mmol). The product **15-PF** precipitated by slow addition of methyl *tert*-butyl ether as a yellow solid (46 mg, 81%) mixture of three diastereomers in a ratio of approximately 2:2:1. ^1^H NMR (CD_3_OD, 400 MHz, ^1^H–^1^H COSY): δ 9.41 (d, 3 × 2H, *J* = 5.7 Hz, C*H*_Py_), 9.09, 9.07 (2 × s, 6H, C*H*_Tria_), 8.18–8.106 (m, 12H, C*H*_Py_), 7.65 (m, 3 × 2H, C*H*_Py_), 6.13–6.10 (m, 6H, C*H*_*p*‑cym_), 6.06, 6.03, 5.92, 5.88 (4 × m, 4 × 3H, C*H*_*p*‑cym_), 5.84–5.82 (m, 6H, C*H*_*p*‑cym_), 5.22, 5.21, 5.216 (3 × dd, 3 × 2H, *J* = 10.7, 2.9 Hz, H-3), 5.01 (d, 2H, *J* = 9.2 Hz, H-1), 5.00 (2 × d, 4H, *J* = 9.2 Hz, H-1), 4.77–4.68 (m, 3 × 2H, H-2), 4.32, 4.31, 4.27, 4.26 (4 × d, 6H, *J* = 2.9 Hz, H-4), 3.96–3.93 (m, 3 × 2H, H-5), 3.88–3.71 (m, 3 × 4H, H-6, H-6′), 2.76 (hept, 3 × 2H, *J* = 6.8 Hz, C*H*_*i*‑Pr_), 2.23, 2.21, 2.20 (3 × s, 18H, *Me*_*p*‑cym_), 1.16 (d, 18H, *J* = 6.8 Hz, *Me*_*i*‑Pr_), 1.11, 1.07 (2 × d, 2 × 9H, *J* = 6.8 Hz, *Me*_*i*‑Pr_). ^13^C­{^1^H} NMR (CD_3_OD, 101 MHz, ^1^H–^13^C HSQC, ^1^H–^13^C HMBC): δ 156.79, 156.76, 156.7 (3 × 2C*H*_Py_), 149.98, 149.97, 149.9 (3 × 2*C*_q(Py)_), 147.74, 147.72, 147.6 (3 × 2*C*_q(Tria)_), 141.5, 141.41, 141.37, 127.40, 127.35 (5 × 2C*H*_Py_), 125.87, 125.85, 125.70, 125.66 (6*C*H_Tria_), 123.48, 123.45, 123.3 (3 × 2C*H*_Py_), 106.51 (1*C*_q_CH_*i*-Pr_), 106.50 (4*C*_q_CH_*i*‑Pr_), 106.48 (1*C*_q_CH_*i*‑Pr_), 103.86, 103.85, 103.64 (3 × 2*C*_q_Me), 87.53, 87.47, 87.3 (3 × 2*C*H_*p*‑cym_), 86.34, 86.3 (6C-1), 86.03, 86.01, 85.9, 85.6, 85.41, 85.38, 84.8, 84.66, 84.65 (18*C*H_*p*‑cym_), 81.12, 81.08 (6C-5), 71.0, 70.8 (6C-3), 69.60, 69.59, 69.4 (3 × 2C-4), 68.44, 68.40, 68.24, 68.20 (3 × 2C-2), 62.58, 62.55 (6C-6), 32.31, 32.25 (6*C*H_*i*‑Pr_), 22.66, 22.65, 22.60, 22.0, 21.9, 21.8 (6 × 2*Me*_*i*‑Pr_), 18.86 (2*Me*_*p*‑cym_), 18.7 (2 × 2*Me*_*p*‑cym_). ^31^P­{^1^H} NMR (CD_3_OD, 162 MHz): δ −159.28 (hept, ^1^ *J*_(P–F)_ = 708.4 Hz). ^19^F NMR (CD_3_OD, 376 MHz): δ −74.57 (d, ^1^ *J*_(F–P)_ = 708.4 Hz).

^1^H NMR (D_2_O, 400 MHz, COSY): δ 9.40 (dd, 3 × 2H, *J* = 5.7, 1.4 Hz, C*H*_Py_), 9.06, 9.03 (3 × s, 6H, C*H*_Tria_), 8.18 (ddd, 3 × 2H, *J* = 7.7, 7.4, 1.4 Hz, C*H*_Py_), 8.09 (dd, 3 × 2H, *J* = 7.7, 1.5 Hz, C*H*_Py_), 7.68 (ddd, 3 × 2H, *J* = 7.4, 5.7, 1.5 Hz, C*H*_Py_), 6.22, 6.19 (2 × m, 6H, C*H*_*p*‑cym_), 6.11, 6.10 (2 × m, 3H, C*H*_*p*‑cym_), 6.07 (m, 3H, C*H*_*p*‑cym_), 5.99, 5.95 (2 × m, 6H, C*H*_*p*‑cym_), 5.87 (2 × m, 3H, C*H*_*p*‑cym_), 5.85 (m, 3H, C*H*_*p*‑cym_), 5.31, 5.28 (2 × dd, 6H, *J* = 10.7, 3.0 Hz, H-3), 5.24, 5.23, 5.22 (3 × d, 6H, *J* = 9.8 Hz, H-1), 4.60, 4.59, 4.51, 4.50 (4 × dd, 6H, *J* = 10.7, 9.8 Hz, H-2), 4.41, 4.40 (2 × d, 6H, *J* = 3.0 Hz, H-4), 4.14–4.10 (m, 6H, H-5), 3.93–3.87 (m, 6H, H-6), 3.84–3.79 (m, 6H, H-6′), 2.73–2.59 (m, 6H, C*H*_*i*‑Pr_), 2.22, 2.21 (2 × s, 18H, *Me*_*p*‑cym_), 1.09, 1.08, 1.03, 0.99 (4 × d, 36H, *J* = 6.9 Hz, *Me*_*i*‑Pr_). ^13^C­{^1^H} NMR (D_2_O, 101 MHz, HSQC, HMBC, HSQC TOCSY): δ 155.8, 155.7 (6C*H*_Py_), 148.5 (6*C*_q(Py)_), 147.5, 147.4 (6*C*_q(Tria)_), 141.2 (6C*H*_Py_), 127.3 (6C*H*_Py_), 125.7, 125.2 (6*C*H_Tria_), 123.5 (6C*H*_Py_), 105.3, 105.2 (6*C*_q_CH_*i*‑Pr_), 103.9, 103.72, 103.70 (6*C*_q_Me), 86.7, 86.6 (6*C*H_*p*‑cym_), 85.84, 85.81, 85.6 (6*C*H_*p*‑cym_), 84.83, 84.80 (6C-1), 84.9, 84.64, 84.62 (6*C*H_*p*‑cym_), 83.78, 83.76 (6*C*H_*p*‑cym_), 80.2, 80.1 (6C-5), 69.5, 69.3 (6C-3), 68.6, 68.4 (6C-4), 67.5, 67.4, 67.3 (3 × 2C-2), 61.63, 61.61, 61.60 (3 × 2C-6), 31.4, 31.3, 31.2 (3 × 2*C*H_*i*‑Pr_), 21.92, 21.90, 21.89, 21.4, 21.3 (12*Me*_*i*‑Pr_), 18.6 (6*Me*_*p*‑cym_). ^31^P­{^1^H} NMR (D_2_O, 162 MHz): δ −161.97 (hept, ^1^ *J*_(P–F)_ = 708.9 Hz). ^19^F NMR (D_2_O, 376 MHz): δ −73.32 (d, ^1^ *J*_(F–P)_ = 708.9 Hz). HRMS-ESI (*m*/*z*): [M – 2PF_6_]^2+^ calculated for C_46_H_58_Cl_2_N_8_O_8_Ru_2_S, 578.0781; found 578.0787.

#### Bisruthenium TDG Complex **16-I**

Complex **16-I** was prepared according to the general complexation procedure starting from the diiodo­(*p*-cymene)­ruthenium­(II) dimer (19 mg, 0.019 mmol) in dichloromethane (1 mL) and disaccharide **21** (12 mg, 0.020 mmol) in a mixture of MeOH/DCM 1:1 (1 mL). The product **16-I** (25 mg, 80%) precipitated from MeOH solution by the addition of methyl *tert*-butyl ether as a red solid mixture of three diastereomers in a ratio of approximately 4:4:1. ^1^H NMR (CD_3_OD, 400 MHz, COSY): δ 9.35–9.32 (m, 3 × 2H, C*H*_Py_), 9.19 (s, 1H, C*H*_Tria_), 9.17, 9.15 (2 × s, 2 × 2H, C*H*_Tria_), 9.13 (s, 1H, C*H*_Tria_), 8.25–8.08 (m, 3 × 4H, C*H*_Py_), 7.60–7.58 (m, 3 × 2H, C*H*_Py_), 6.09–5.98, 5.94–5.84 (2 × m, 2 × 12H, C*H*_*p*‑cym_), 5.23, 5.20 (3 × dd, 3 × 2H, *J* = 10.7, 2.9 Hz, H-3), 5.02 (d, 3H, *J* = 9.6 Hz, H-1), 4.97, 4.96 (2 × d, 3H, *J* = 9.6 Hz, H-1), 4.81–4.69 (m, 3 × 2H, H-2), 4.38, 4.36, 4.26, 4.25 (4 × d, 6H, *J* = 2.9 Hz, H-4), 3.99–3.92 (m, 3 × 2H, H-5), 3.84–3.71 (m, 3 × 4H, H-6, H-6′), 3.06–2.90 (m, 3 × 2H, C*H*_*i*‑Pr_), 2.39, 2.33. 2.32 (3 × s, 3 × 6H, *Me*_*p*‑cym_), 1.22–1.09 (m, 3 × 12H, *Me*_*i*‑Pr_). ^13^C­{^1^H} NMR (CD_3_OD, 101 MHz, HSQC, HMBC): δ 158.1, 158.0, 157.9 (3 × 2C*H*_Py_), 150.1, 150.0, 149.9, 149.8 (6*C*_q(Py)_), 147.58, 147.56, 147.43. 147.41 (6*C*_q(Tria)_), 141.0, 140.94, 140.91, 140.8 (6C*H*_Py_), 127.00, 126.98, 126.96, 126.9 (6C*H*_Py_), 125.8, 125.6 (2 × 3*C*H_Tria_), 123.7, 123.3, 123.2 (3 × 2C*H*_Py_), 108.5, 108.4, 108.0, 107.9 (6*C*_q_CH_*i*‑Pr_), 102.6 (3 × 2*C*_q_Me), 87.4, 86.74, 86.71, 86.69, 86.6, 86.4, 85.77, 85.75, 85.7, 85.6 (24*C*H_*p*‑cym_, 6C-1), 81.2, 81.1, 81.0 (3 × 2C-5), 70.7, 70.6 (6C-3), 69.4, 69.3 (6C-4), 68.6, 68.1, 68.0 (3 × 2C-2), 62.6, 62.61, 62.57 (3 × 2C-6), 33.1, 33.0, 32.9 (3 × 2*C*H_*i*‑Pr_), 23.2, 23.1, 22.9, 22.1, 22.0, 21.9 (6 × 2*Me*_*i*‑Pr_), 20.1, 19.9 (6*Me*_*p*‑cym_). HRMS-ESI (*m*/*z*): [M – 2I]^2+^ calculated for C_46_H_58_I_2_N_8_O_8_Ru_2_S 670.0143; found 670.0135.

#### Bisruthenium TDG Complex **17-Cl**

Complex **17-Cl** was prepared according to the general complexation procedure starting from dichloro­(benzene)­ruthenium­(II) dimer (23 mg, 0.046 mmol) in dichloromethane (1 mL) and disaccharide **21** (28 mg, 0.046 mmol) in a 1:1 MeOH/DCM mixture (2 mL). The product **17-Cl** (42 mg, 83%) precipitated from the MeOH solution by the addition of methyl *tert*-butyl ether as a yellow solid mixture of three diastereomers. ^1^H NMR (DMSO-*d*_6_, 400 MHz, COSY): δ 9.59–9.57 (m, 3 × 2H, C*H*_Py_), 9.42 (s, 2H, C*H*_Tria_), 9.40 (2 × s, 2 × 2H, C*H*_Tria_), 8.30–8.22 (m, 3 × 4H, C*H*_Py_), 7.69–7.66 (m, 3 × 2H, C*H*_Py_), 6.19, 6.18 (2 × s, 3 × 12H, C*H*_*PhH*_), 5.80–5.77 (m, 3 × 2H, O*H*-2), 5.72–5.65 (m, 3 × 2H, O*H*-4), 5.23, 5.20 (2 × dd, 3 × 2H, *J* = 10.6, 2.9 Hz, H-3), 5.04, 5.03 (2 × d, 3 × 2H, *J* = 9.5 Hz, H-1), 4.92, 4.85 (2 × t, 3 × 2H, *J* = 6.0 Hz, O*H*-6), 4.30–4.20 (m, 8H, H-2, H-4), 4.12–4.06 (m, 2 × 2H, H-4), 3.82–3.79 (m, 3 × 2H, H-5), 3.61–3.57 (m, 3 × 4H, H-6, H-6′). ^13^C­{^1^H} NMR (DMSO-*d*_6_, 101 MHz, HSQC, HMBC, HSQC TOCSY): δ 156.0 (3 × 2C*H*_Py_), 148.28, 148.25 (6*C*_q(Py)_), 145.7, 145.5 (6*C*_q(Tria)_), 141.3, 125.7 (2 × 6C*H*_Py_), 125.5, 125.4 (6*C*H_Tria_), 122.1, 122.0 (6C*H*_Py_), 86.0 (3 × 12*C*H_*PhH*_), 83.3, 83.21, 83.16, 83.1 (6C-1), 79.0, 78.8 (6C-5), 69.1, 69.0 (6C-3), 67.1 (6C-4), 66.8, 66.6 (6C-2), 59.9, 59.6 (6C-6). HRMS-ESI (*m*/*z*): [M – 2Cl]^2+^ calculated for C_38_H_42_Cl_2_N_8_O_8_Ru_2_S, 522.0154; found 522.0158.

#### Bisruthenium TDG Complex **18-Cl**

Complex **18-Cl** was prepared according to the general complexation procedure starting from dichloro­(hexamethylbenzene)­ruthenium­(II) dimer (33 mg, 0.049 mmol) in dichloromethane (1 mL) and disaccharide **21** (30 mg, 0.049 mmol) in a mixture of MeOH/DCM 1:1 (2 mL). The product **18-Cl** (51 mg, 81%) precipitated from the MeOH solution by the addition of methyl *tert*-butyl ether as a yellow solid mixture of three diastereomers. ^1^H NMR (DMSO-*d*_6_, 400 MHz, COSY): δ 9.43, 9.42, 9.41 (3 × s, 3 × 2H, C*H*_Tria_), 8.91–8.89 (m, 3 × 2H, C*H*_Py_), 8.30–8.18 (m, 3 × 4H, C*H*_Py_), 7.71–7.67 (m, 3 × 2H, C*H*_Py_), 5.80–5.72 (m, 3 × 4H, O*H*-2, O*H*-4), 5.25 (dd, 3 × 2H, *J* = 10.6, 3.2 Hz, H-3), 5.08, 5.04 (3 × d, 3 × 2H, *J* = 9.5 Hz, H-1), 4.99, 4.96, 4.88, 4.85 (4 × t, 6H, *J* = 6.1 Hz, O*H*-6), 4.26–4.09 (m, 3 × 4H, H-2, H-4), 3.84, 3.79 (2 × t, 6H, *J* = 6.7 Hz, H-5), 3.60–3.58 (m, 3 × 4H, H-6, H-6′), 2.11, 2.10, 2.02 (3 × s, 3 × 36H, *Me*). ^13^C­{^1^H} NMR (DMSO-*d*_6_, 101 MHz, HSQC, HMBC, HSQC TOCSY): δ 153.1 (3 × 2C*H*_Py_), 148.04, 147.95, 147.94 (3 × 2*C*_q(Py)_), 145.5, 145.2 (6*C*_q(Tria)_), 139.7 (3 × 2C*H*_Py_), 126.0 (3 × 2C*H*_Py_), 125.2 (3 × 2*C*H_Tria_), 121.6, 121.5 (6C*H*_Py_), 96.1, 94.8 (3 × 12*C*_q_Me), 83.0, 82.92, 82.85 (3 × 2C-1), 79.1, 78.8 (6C-5), 69.0 (6C-3), 67.5 (6C-2), 67.2, 66.8, 66.5 (3 × 2C-4), 59.8, 59.6 (6C-6), 16.6, 15.4, 15.28, 15.26 (36*Me*). HRMS-ESI (*m*/*z*): [M – 2Cl]^2+^ calculated for C_50_H_66_Cl_2_N_8_O_8_Ru_2_S 606.1096; found 606.1098.

### Bioanalytical Methods

Detailed experimental procedures for the determination of the affinity to galectins by fluorescence polarization and intrinsic tryptophan fluorescence are described in Supporting Information, Section D. The experimental procedures for the determination of cell viability are described in Supporting Information, Section G.

### Production of Galectins

Preparation and purification of *h*Gal-1, *h*Gal-3-CRD, and monobiotinylated protein constructs of *h*Gal-1 and full-length *h*Gal-3 (*h*Gal-1-AVI, *h*Gal-3-AVI) are described in detail in the Supporting Information, Section C.

### Biolayer Interferometry

The kinetic analysis of the best-performing ruthenium complex-based inhibitors targeting galectin constructs (*h*Gal-1, *h*Gal-3) was performed by using biolayer interferometry. The galectin constructs carried a 15-amino acid AVI-tag at the N-terminus, selectively monobiotinylated during heterologous production in *E. coli*. Experiments were conducted under controlled conditions (25 ± 0.1 °C, 1000 rpm) using an Octet Red96e instrument (FortéBio, Fremont, CA, USA). Optimized galectin concentrations of 10 μg/mL (*h*Gal-1) or 50 μg/mL (*h*Gal-3) were used across all of the experiments. Biotinylated galectin constructs were diluted to their respective optimal concentrations in PBS containing 0.05% Tween-20 and immobilized (300 s) on streptavidin-coated biosensors (Octet SA Biosensors, Sartorius, Göttingen, Germany) via biotin–streptavidin coupling. The interactions between the immobilized constructs and serially diluted inhibitors (62.5–500 μM for *h*Gal-3; 4.9–400 nM for *h*Gal-1) were monitored over a total of 1050 s, including an association phase of 450 s and a dissociation phase of 600 s. Immobilization did not affect galectin activity. BLI data were analyzed using Octet Analysis Studio (Sartorius, Göttingen, Germany). Background signals from nonspecific interactions (<10% of the total response) and sensor drift were corrected using a double-reference subtraction method. Kinetic data were evaluated using a Langmuir 1:1 kinetic model, and all data sets were further validated through steady-state analysis as an additional method of kinetic data evaluation.

### Inhibition of Galectin Binding to Cancer Cells

The cell binding inhibition assay was performed similarly to the previous studies with other galectins.^90,91^ Ruthenium complex-based inhibitors or lactose (used as a positive control) were serially diluted in a PBS/BSA buffer (50 mM NaH_2_PO_4_, 150 mM NaCl, and 1% w/v bovine serum albumin, pH 7.5). These were then premixed with Gal-1-AVI (final concentration 5 μg/mL, BSA calibrated) on ice for 30 min. The mixtures were combined with a suspension of MDA-MB-231 breast cancer cells (7.5 × 10^4^ cells in a 100 μL final volume) in PBS/BSA buffer. The resulting mixture was incubated in U-bottom 96-well tissue culture plates (TPP Techno Plastic Products, CH) for 60 min on ice. After incubation, the cells were washed with PBS/BSA buffer and centrifuged (200 × *g*, 3 min, 4 °C). The surface-bound Gal-1-AVI was labeled with a streptavidin–phycoerythrin conjugate (1:249 dilution; 50 μL; BioLegend, USA) for 45 min on ice. Following another wash with PBS/BSA, the cells were resuspended in PBS/BSA buffer and analyzed by the flow cytometer ZE5 Cell Analyzer (Bio-Rad, CA, USA) using Hoechst 33258 stain (1 mg/mL). Quadruplicate results were obtained. During data analysis with FlowJo software (Tree Star, Ashland, OR, USA), cell aggregates and dead cells were excluded through appropriate gating. The half-maximal inhibitory concentrations (*IC*_50_) were calculated from the nonlinear fitting of the mean fluorescence intensities of phycoerythrin-stained cells against glycomimetic concentrations with values reported along with standard deviations (SD). Under these assay conditions, lactose (positive control) had an *IC*_50_ value of 4.3 ± 0.5 μM.

### Jurkat Preaparesis Assay

Jurkat cells (1 × 10^6^/mL) were cultured for 30 min at 37 °C in the presence of 20 μM recombinant *h*Gal-1. For preaparesis analysis, cells were subsequently stained with Annexin V–Alexa Fluor (Invitrogen, cat. no. R37174) according to the manufacturer’s instructions, and signals of 10 000 cells were measured (FacsVerse flow cytometer (BD Biosciences)) and analyzed (BD FACSuiteTM Software). The effect of *h*Gal-1 inhibitors was determined by incubating *h*Gal-1 with equimolar amounts of inhibitors for 1 h at 4 °C, before addition to the cell culture. All experiments were performed in three to five biological replicates, each in three parallels. Results are expressed as the percentage of Annexin V positive cells out of the total cell population. Inhibition of *h*Gal-1-induced preaparesis in Jurkat cells by complexes **16-I** and **17-Cl** is displayed in the Supporting Information, Figure S20. Representative flow cytometry histograms of Jurkat cells stained with Annexin V–Alexa Fluor 488 including negative controls are shown in the Supporting Information, Figures S21–S23.

## Supplementary Material





## Notes

The following files are available free of charge. The Supporting Information is available free of charge at https://pubs.acs.org/doi/10.1021/acs.jmedchem.5c03436.

Schemes of compound synthesis, ligand exchange behavior, production of galectins, determination of the affinity by competitive fluorescence polarization and intrinsic tryptophan fluorescence, determination of cell viability, supplementary figures and tables, and copies of NMR spectra and HPLC traces (PDF)
Molecular formula strings (CSV)

## Abbreviations

ANOVA: analysis of variance
BLI: biolayer interferometry
CRD: carbohydrate recognition domain
C_q_: quaternary carbon
DCM: dichloromethane
EtOAc: ethyl acetate
FP: fluorescence polarization
*h*Gal-1: human galectin
*h*Gal-3: human galectin-3
*h*Gal-1-AVI: *h*Gal-1 protein construct carrying a monobiotinylated AVI-tag
*K*_D_: dissociation constant
*k*_a_: association rate constant
*k*_d_: dissociation rate constant
ITF: intrinsic fluorescence of tryptophan residues
Lac: lactose
LacNAc: *N*-acetyllactosamine
MP-OES: microwave plasma-optical emission spectrometry
*p*-cym: *p*-cymene
PE: petroleum ether
*rp*: relative potency
SD: standard deviation
SS: steady-state analysis
TDG: thiodigalactoside
TOCSY: total correlation NMR spectroscopy
Tria: 1,2,3-triazole
