# Supplementary material for: Organoruthenium Glycomimetics Exhibit High Selectivity and Nanomolar Affinity for Human Galectin‑1
Source: J Med Chem. 2026 Feb 5;69(4):4789–809. doi: 10.1021/acs.jmedchem.5c03436 (PMC12951563; doi:10.1021/acs.jmedchem.5c03436)
Supplement: Supplementary file 1 [file jm5c03436_si_001.pdf]

SUPPORTING INFORMATION

Organoruthenium Glycomimetics Exhibit High Selectivity and Nanomolar Affinity for Human Galectin-1.

Vojtěch Hamala,[a] Martin Kurfiřt,[a] Lucie Červenková Šťastná,[a] Filip Dvořák,[a] Jana Bernášková,[a] Adéla Sýkorová,[a] Jaroslav Kozák,[c] Martin Zavřel,[c] Tatiana Staroňová,[d] Peter Šebest,[d] Veronika Ostatná,[d] Jakub Červený,[e,f] Pavla Bojarová,[e] Jitka Holčáková,[g] Tomáš Hrstka,[g] Roman Hrstka,[*,g] Jindřich Karban,[*,a]

[a] *Institute of Chemical Process Fundamentals of the Czech Academy of Sciences, Rozvojová 1/135, 165 00 Praha, Czech Republic.*
[b] *Department of Organic Chemistry, University of Chemistry and Technology, Technická 5, 166 28 Praha, Czech Republic.*
[c] *Institute of Organic Chemistry and Biochemistry of the Czech Academy of Sciences, Flemingovo nám. 542, 160 00 Praha, Czech Republic*
[d] *Institute of Biophysics of the Czech Academy of Sciences, Královopolská 135, 612 00 Brno, Czech Republic*
[e] *Institute of Microbiology of the Czech Academy of Sciences, Vídeňská 1083, 142 00 Praha, Czech Republic*
[f] *Department of Analytical Chemistry, Faculty of Science, Charles University, Hlavova 8, 128 43 Praha 2, Czech Republic*
[g] *Research Centre for Applied Molecular Oncology, Masaryk Memorial Cancer Institute, Žlutý kopec 7, 656 53 Brno, Czech Republic.*
[*]E-Mail: roman.hrstka@mou.cz, karban@icpf.cas.cz

Experimental procedures

A. Synthesis Schemes S3
B. Investigation of ligand exchange behavior S6
C. Production of galectins S8
D. Determination of the affinity to galectins S10
E. Inhibition of galectin binding to cancer cells. S21
F. Jurkat apoptosis assay S22
G. Cytotoxicity testing S24
H. Copies of NMR spectra S30
NMR COMPOUND 11-Cl S30
NMR COMPOUND 11-PF S36
NMR COMPOUND 12-Cl S44
NMR COMPOUND 13-Cl S50

NMR COMPOUND 13-PF S56
NMR COMPOUND 14-Cl S64
NMR COMPOUND 15-Cl S70
NMR COMPOUND 15-BF S76
NMR COMPOUND 15-PF S83
NMR COMPOUND 16-I S91
NMR COMPOUND 17-Cl S96
NMR COMPOUND 18-Cl S102
NMR COMPOUND 19 .... S108
NMR COMPOUND 20 .... S113
NMR COMPOUND 21 .... S118
NMR COMPOUND S3 .... S123
NMR COMPOUND S5 .... S128
NMR COMPOUND S6 .... S133
Stability of 15-PF in physiological solution S138
Stability of 15-PF in PBS buffer S139
Stability of 15-PF in blood serum S140
I. HPLC traces S141
J. References S146

A. Synthesis Schemes

Lactose-based inhibitors **11-Cl** and **11-PF**.

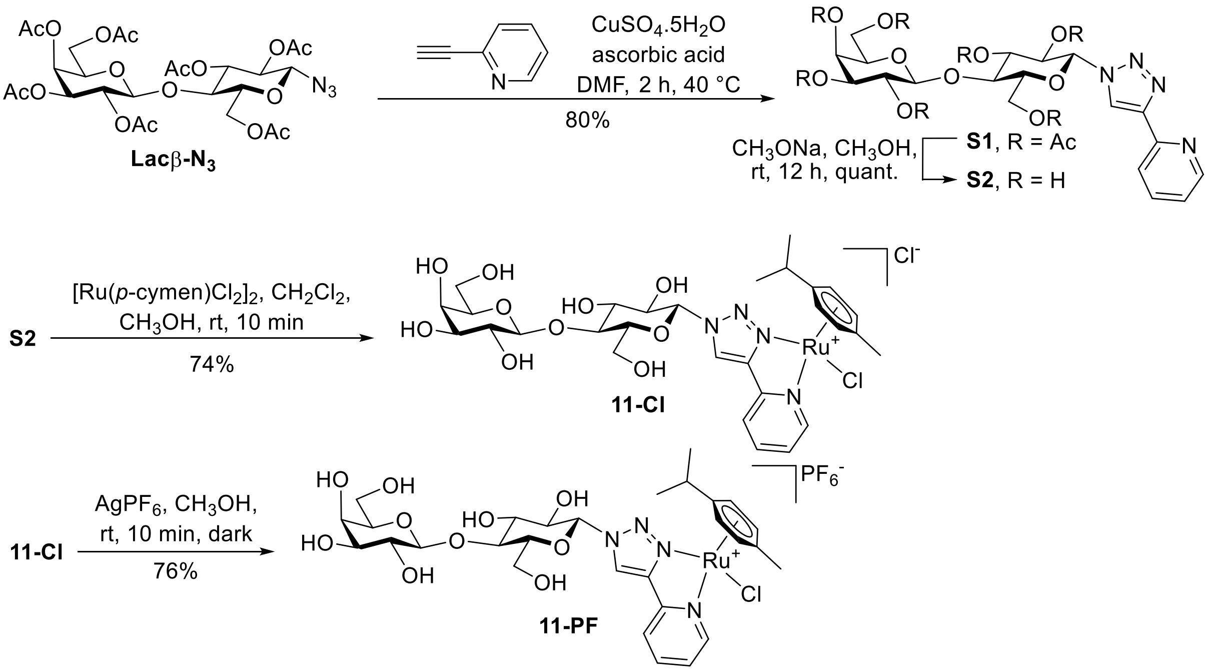

Scheme S1: Preparation of lactose-based inhibitors **11-Cl** and **11-PF**.

Lactose-based inhibitor **12-Cl**.

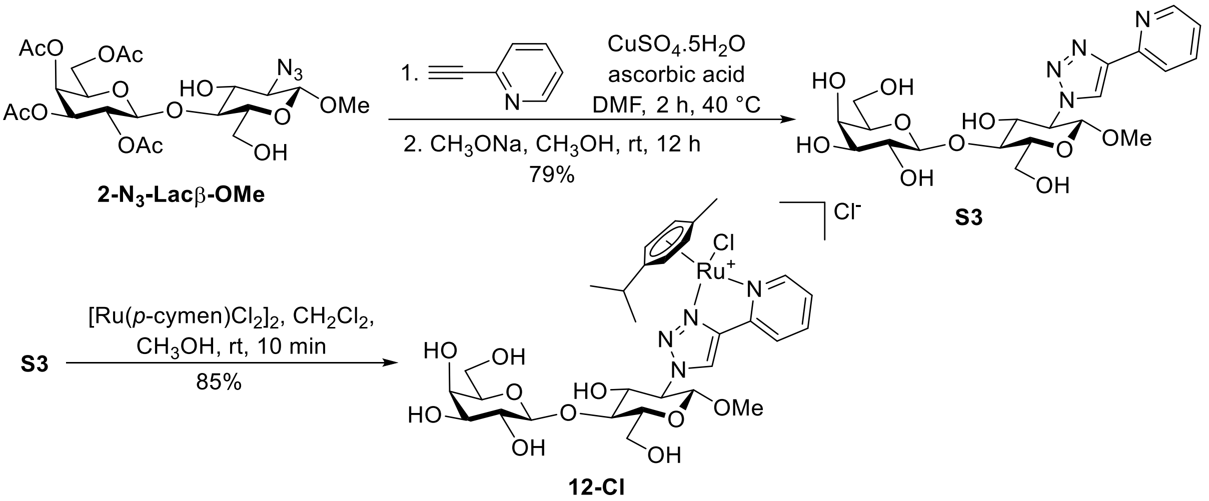

Scheme S2: Preparation of lactose-based inhibitor **12-Cl**.

LacNAc-based inhibitors **13-Cl** and **13-PF**.

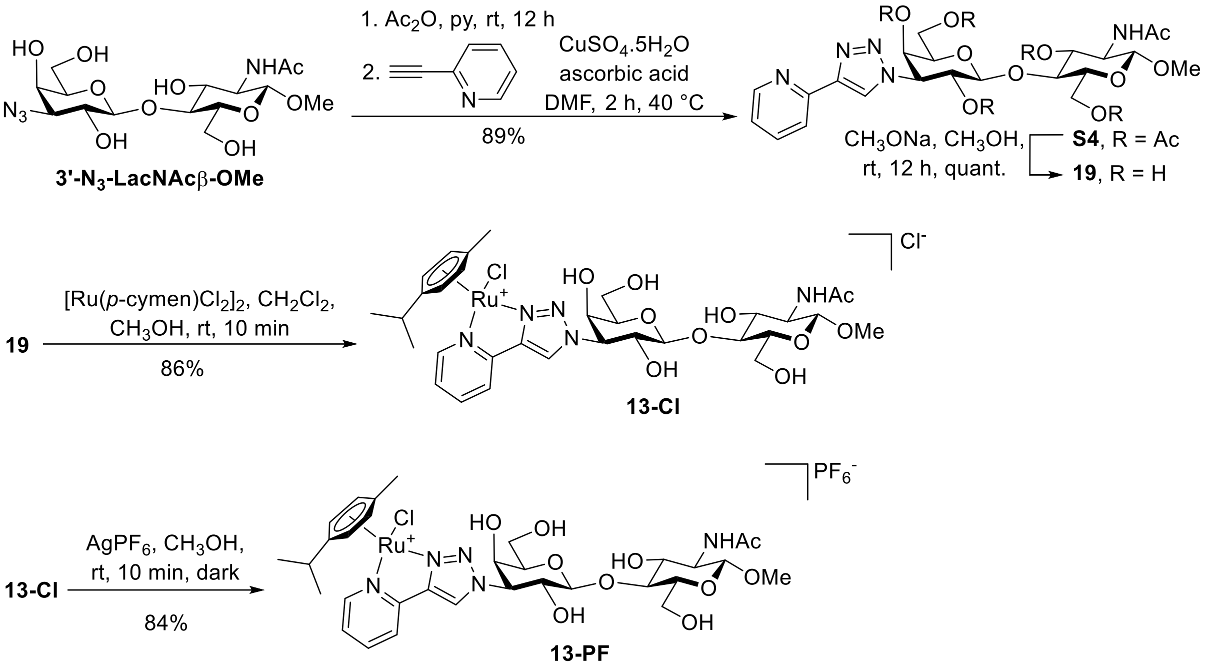

Scheme S3: Preparation of LacNAc-based inhibitors **13-Cl** and **13-PF**.

LacNAc-based inhibitor **14-Cl**.

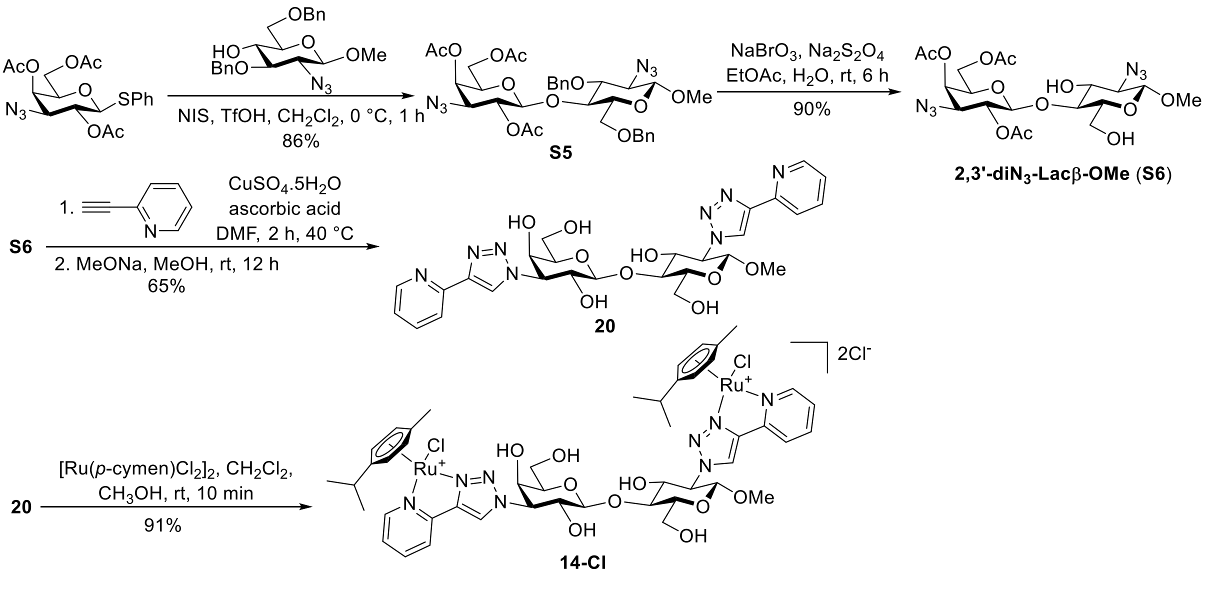

Scheme S4: Preparation of LacNAc-based inhibitor **14-Cl**.

Thiodigalactoside-based inhibitors **15-Cl**, **15-BF**, **15-PF**, **16-I**, **17-Cl** and **18-Cl**.

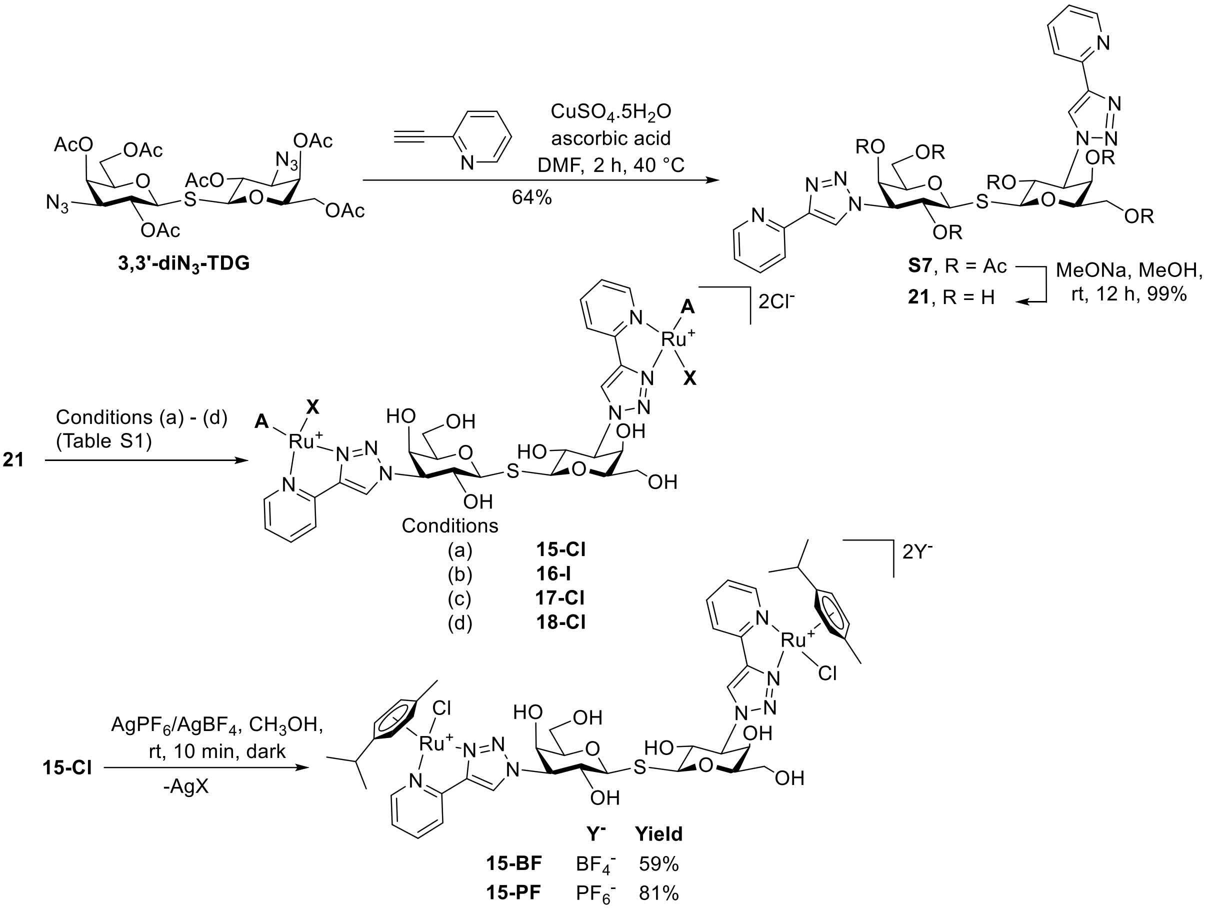

Scheme S5: Preparation of the thiodigalactoside-based inhibitors, see Table S1 for **A** and **X**.

Table S1: Reaction condition and yields of complexation reaction of thiodigalactoside-based inhibitors **15-Cl**, **16-I**, **17-Cl**, and **18-Cl**.

Compound	A	X	Conditions	Yield
**15-Cl**	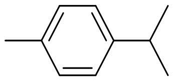	Cl	(a) [Ru(*p*-cymene)Cl_2]$_2$, CH_2Cl_2, CH_3OH, rt, 10 min	67%
**16-I**	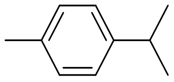	I	(b) [Ru(*p*-cymene)I_2]$_2$, CH_2Cl_2, CH_3OH, rt, 10 min	80%
**17-Cl**	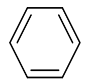	Cl	(c) [Ru(benzene)Cl_2]$_2$, CH_2Cl_2, CH_3OH, rt, 10 min	83%
**18-Cl**	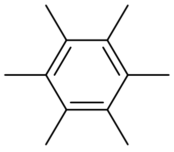	Cl	(d) [Ru(hexamethylbenzene)Cl_2]$_2$, CH_2Cl_2, CH_3OH, rt, 10 min	81%

B. Investigation of ligand exchange behavior

We investigated the ligand exchange behavior of compound **13-PF** in a solvent mixture of H_2O/D_2O (90:10, v/v) by ^{1}H NMR spectroscopy. NMR spectra were recorded on a Bruker Avance 400 spectrometer (1H at 400.1 MHz) at 25 °C using the zgesgp pulse sequence for water suppression (excitation sculpting with gradients). The length of the 90° high-power pulse (P1) was calibrated for each sample using the pulsecal keyword. The power level of the 180° shape pulse (Squa100.1000) was calibrated according to the P1 length using the shapetool utility in the Topspin software. The experiments were performed using the 1 s relaxation delay. The ^{1}H NMR resonances were referenced to an external standard (acetone in D_2O, δH: 2.22 ppm). We used Norell© Standard Series TM 5 mm NMR tubes for all experiments. The volume of all samples was 500 µL. The spectra were processed in MestreNova software (version 12.0.3) by 64 K zero-filling, 0.3 Hz exponential apodization, and automatic baseline correction (Whittaker smoother).

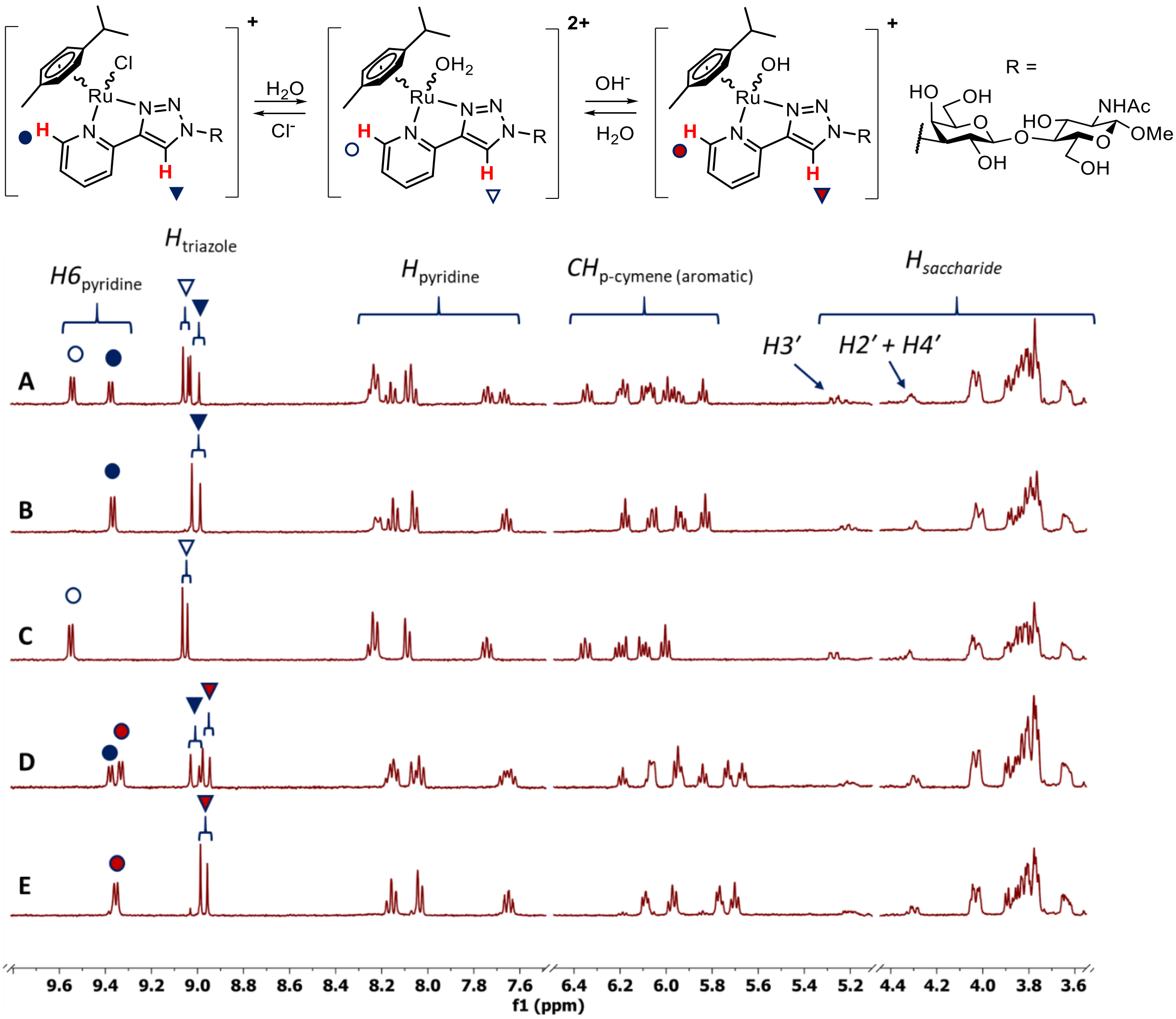

Figure S1. Cutouts of water-suppressed ^{1}H NMR spectra. (**A**) Spectrum of **13-PF** in H_2O/D_2O (90/10 *v/v*) solution (1 mM); (**B**) Spectrum of **13-PF** (1 mM) after addition of NaCl (150 mM); (**C**) Spectrum of **13-PF** (1 mM) after addition of $AgPF_6$ (2 mM); (**D**) Spectrum of **13-PF** (1 mM) acquired

immediately after addition of NaOH (1 mM); (**E**) Spectrum of **13-PF** (1 mM) acquired 24 h after the addition of NaOH (1 mM); ▼,▽,▼ denote signals of the triazole −CH= proton, ●,○,● denote signals of the C6-H pyridine proton.

We studied compound **13-PF** in detail using ^{1}H NMR spectroscopy with excitation sculpting (ES) water suppression. The study revealed a dynamic equilibrium between chloro-, aqua- and hydroxoruthenium species (Fig. S1), influenced by pH and chloride ion concentration. Ligand exchange induced a perturbation of the ^{1}H NMR signals corresponding to protons on the triazole, pyridine and cymene moieties. Slight changes observed for the proton signals at the 2′-, 3′- and 4′-positions of the galactopyranoside moiety had their intensity reduced by the solvent suppression scheme due to their proximity to the water signal. Individual complexes were identified by observing the triazole −CH= (▼,▽,▼), and pyridine C6−H protons (●,○,●) (Fig. S1). The signals of the triazole −CH= protons were particularly sensitive to subtle changes at the ruthenium center and allowed the individual diastereomers of the chloro-, aqua-, and hydroxoruthenium species to be distinguished.

The ^{1}H NMR spectrum of **13-PF** in H_2O/D_2O (90/10 *v/v*) solvent mixture showed two pyridine C6−H doublets (●,○, splitting is due to coupling to the vicinal proton), and two pairs of triazole −CH= singlets (▼,▽), where each singlet in the pair represents one of the two possible diastereomers (Fig. S1-A). This pattern is consistent with partial aquation-dechlorination of the chloro-ruthenium complex in aqueous solution, in agreement with previous reports.[1-3] Accordingly, the signals were assigned to the two diastereomers of the chloro-ruthenium complex (●,▼) and the corresponding two diastereomers of the aqua-ruthenium complex (○,▽). Addition of sodium chloride (c = 150 mM) resulted in the immediate disappearance of the signals assigned to the aqua-ruthenium complex (Fig. S1-B), confirming the chemical equilibrium between the chloro- and aqua-complexes. Similarly, addition of 2 mM silver hexafluorophosphate caused disappearance of the signals of the chloro-ruthenium complex (Fig. S1-C), validating the assignment. Adding aqueous sodium hydroxide to a solution of **13-PF** in H_2O/D_2O mixture (90/10 *v*/*v*, the concentration of NaOH reached approximately 1 mM) resulted in the disappearance of the aqua-ruthenium complex and the immediate occurrence of a new set of signals (●,▼), which were assigned to the hydroxoruthenium complex in a mixture with the chloro-ruthenium species (●,▼) (Fig S1-D). Subsequently, the ^{1}H NMR spectrum acquired 24 h after the addition of sodium hydroxide revealed the predominant presence of the two diastereomers of the hydroxoruthenium complex (●,▼) (Fig S1-E).

C. Production of galectins

Preparation and purification of human galectin-1 (*h*Gal-1)

The coding sequence of *h*Gal-1 (NM_002305.4) was cloned using Gateway recombination technology (Invitrogen) into the pDEST15 vector containing an N-terminal His_6-GST tag cleavable by TEV protease (Tobacco Etch Virus nuclear-inclusion-a endopeptidase). The cloned gene was expressed in *E. coli* BL21 RILP. The bacteria were cultured in 100 mL of LB medium (10 g/L tryptone, 5 g/L yeast extract, 5 g/L NaCl) with ampicillin (100 µg/mL) and chloramphenicol (30 µg/mL) overnight at 37 °C, 220 rpm. The culture was diluted to a total volume of 2.5 liters in LB medium (supplemented with ampicillin and chloramphenicol) to an optical density at $OD_{600} = 0.1$ and cultured for 2–3 hours. Upon reaching an optical density at $A_{600}=0.5$, gene expression was induced by addition of isopropyl-β-D-thiogalactopyranoside to the culture (final concentration 1 mM). The bacterial culture was further cultured for 4 hours at 30 °C, 140 rpm, and then pelleted by centrifugation (4000 g/4 °C/10 min). Bacterial pellets were resuspended in lysis buffer (50 mM Tris-HCl, pH 7.5; 0.15 M NaCl, 0.75% CHAPS). Cell suspensions were subsequently enriched with lysozyme (1 mg/mL), phenylmethylsulfonyl fluoride (1 mM), turbonuclease (1:1000), and $MgCl_2$ (1 mM), and then sonicated with an ultrasonic sonicator Sonoplus, probe VS 70T, for 15 cycles (10 s sonication, 50 s pause, 35W) at 4 °C. Bacterial lysates were obtained by centrifugation for 30 minutes at 10,000 g/4 °C. His6-GST-tagged proteins were captured on a GSTrap glutathione agarose column (GE Healthcare), eluted with binding buffer (50 mM Tris-HCl, pH 7.5; 0.15 M NaCl) enriched with 20 mM glutathione, and subjected to TEV protease cleavage (1 mg of protease per 10 mg of purified protein) overnight at 4 °C. To remove His_6-GST and His_6-TEV protease, protein mixtures were applied to a HisTrap column (GE Healthcare). Fractions containing purified protein were collected in a 96-well plate via a fractionator. After concentration, protein was solubilized in final testing buffers (25 mM Tris-HCl pH 8.0, 300 mM NaCl and 1mM TCEP) using Zeba spin desalting columns that separate molecules with a molecular weight up to 10 kDa (Thermo Fisher Scientific). The purity of the isolated protein was verified by SDS-PAGE electrophoresis and staining with Coomassie Brilliant Blue. The TEV protease was prepared in the laboratory following the method from lit.[4]

Preparation and purification of human galectin-3-CRD (*h*Gal-3-CRD)

The DNA sequence of galectin-3 CRD domain (AA 113-250) was amplified by PCR (oligonucleotides pET_G3D_F 5‘-TTTAAGAAGGAGATATACATATGCCACTGATTGTGCCTTA-3‘ and pET_G3D_R 5‘-GGTGCTCGAGTGCGGCCGCATCATATCATGGTATATGAAG -3‘) from plasmid LGALS3 (Origene; RC208785) and subcloned into NdeI and HindIII restricted vector pET-22b(+)using Gibson assembly. G3D was expressed in *E. coli* strain BL21 (DE3). The expression was induced in cells grown in LB media at 37 °C and 180 rpm to the growth density of approximately $OD_{600} = 0.5$ using 0.4 mM IPTG and further grown at 22 °C for 16 hours. The cell pellet from 1 liter of LB media (approximately 3 ml) was resuspended in 20 mL of Lysis buffer (0.1 M Na-phosphate buffer pH 7.4,

0.5 M NaCl, 5 mM β-mercaptoethanol, 5 mM $MgCl_2$, 0.5 mg/ml lysozyme and 125 U Novagen® Benzonase) and sonicated for 2 minutes (MSE Soniprep 150; 75% power and amplitude 14 microns). After incubation on ice for 30 minutes, the cell lysate was spun for 30 minutes at 20 000 × g. The cleared lysate was run through chromatographic column containing apropriate amount of lactosyl-Sepharose (binding capacity approximately 10 mg/mL), prepared as described before.[5] The column was washed with 5 column volumes of Wash buffer (50 mM Na-phosphate buffer pH 7.4, 1 M NaCl and 5 mM β-mercaptoethanol) and galectin was eluted using appropriate amount of elution buffer (50 mM Na-phosphate buffer pH 7.4, 0.1 M NaCl, 5 mM β-mercaptoethanol and 0.1 M β-lactose). The galectin was further purified by gel filtration (column SuperdexTM 75 10/300 GL, GE Healthcare) using phosphate buffered saline (PBS) containing 5 mM β-mercaptoethanol as mobile phase. The eluted protein was concentrated (Amicon® Ultra – 15, Ultracel® - 10K; Merck Millipore), 10 × dialyzed 1:200 against PBS containing 5 mM β-mercaptoethanol in D-tubeTM Dialyzer Mega (MWCO 3.5 kDa; Merck Millipore) and further stored at 4 °C.

Preparation and purification of monobiotinylated protein constructs of full-length human galectin-1 and galectin-3 (*h*Gal-1-AVI, *h*Gal-3-AVI)

For biolayer interferometry (BLI) and flow cytometry experiments, selectively mono-biotinylated galectin constructs were produced to be attached to the biosensor or visualized by a streptavidin-conjugated antibody. *In vivo* monobiotinylation during heterologous expression was directed to a single lysine in the AVI-tag sequence (GLNDIFEAQ**K**IEWHE) under the catalysis by a co-expressed biotin ligase. The AVI-tag was added to the flexible N-terminal domain of Gal-3, while a 15-residue linker was introduced between the AVI-tag and Gal-1 to preserve its lectin activity.

The AVI-tagged constructs were expressed in *E. coli* BL21(λDE3) cells carrying a plasmid with an IPTG-inducible gene *birA* encoding for biotin ligase as described previously.[6,7] The transformed *E. coli* cells were cultured in MDO medium (20 g/L yeast extract, 20 g/L glycerol, 1 g/L KH_2PO_4, 3 g/L K_2HPO_4, 2 g/L NH_4Cl, 0.5 g/L Na_2SO_4) supplemented with ampicillin (150 μg/mL) and chloramphenicol (10 μg/mL) and cultivated at 37 °C and 140 rpm. When OD_{600} reached 0.6, D-biotin (50 μM; 12 μg/mL) was added along with 1 mM IPTG. The cells were grown for an additional 4 h at 37 °C and harvested by centrifugation (8880 × g, 20 min, 4 °C).

For the purification of galectins, harvested cells were suspended in an equilibration buffer (20 mM phosphate/500 mM NaCl/20 mM imidazole pH 7.4). Phenylmethylsulfonyl fluoride (PMSF, 1% *v/v*) was added to prevent cleavage by proteases. The suspension was sonicated using UltraSonic Processor UP50H (Ultrasound Technologies, Caldicot, UK) for 6 cycles (1 min pulse, 2 min break on ice). After centrifugation (20 230 × g, 20 min, 4 °C), the cell-free extract was loaded on an equilibrated Ni-NTA column (GE Medical Systems, Prague, Czech Republic). Bound galectins were eluted with an elution buffer containing 500 mM imidazole. Fractions were analyzed for protein content using Bradford assay

calibrated for bovine serum albumin (BSA), pooled and dialyzed overnight in PBS buffer pH 7.5 (7 L) containing 2 mM EDTA, followed by 4 h dialysis in PBS buffer (7 L). The purity of prepared galectins was confirmed on SDS-PAGE, *h*Gal-1 and *h*Gal-3 proteins were stable at 4 °C for approximately two months. The success of biotinylation of AVI-tagged galectins was confirmed by Western Blot. The lectin activity of AVI-tagged galectins was confirmed to be the same as of His-tagged galectins.

D. Determination of the affinity to galectins

Competitive fluorescence polarization (FP)

FP experiments were performed analogically to the procedures reported previously.[8–10] with Tecan Spark multiplate reader (Tecan, Switzerland) equipped with fluorescence polarization filters using excitation 485 ± 20 nm and emission 535 ± 20 nm in 384 well black polystyrene microplate (Corning 4514, USA) with the temperature set up to 25 °C. 3-Deoxy-3-[4-(fluorescein-5-yl-carbonylaminomethyl)-1*H*-1,2,3-triazol-1-yl]-β-D-galactopyranosyl 3-deoxy-3-[4-(3-fluorophenyl)-1*H*-1,2,3-triazol-1-yl]-1-thio-β-D-galactopyranoside, prepared as described previously,[8] was used as an fluorescence probe. Affinities to *h*Gal-1 were determined in PBS buffer with 4% DMSO and 0.01% Triton X-100 using protein concentration 500 nM, fluorescence probe concentration 100 nM and at least ten different concentrations (depending on the expected affinity) of the tested compound in triplicates. Affinities to *h*Gal-3-CRD were determined in PBS buffer with 4% *v/v* DMSO and 0.01% *v/v* Triton X-100 using protein concentration 50 nM, fluorescence probe concentration 5 nM and at least ten different concentrations (depending of the expected affinity) of tested compound in triplicates. The fluorescence probe K_D (Table S1) for each galectin was determined by direct fluorescence polarization titration (Fig. S2) using the same batch of galectins later employed for compound measurements. Compounds were pre-spotted into the 384 well plate using Echo 650 (Backmann Coulter, USA) liquid handler and 5 µL of galectin and probe mixture was added using Certus Flex (Fritz Gyger AG, Switzerland) liquid handler. FP was measured after 30–60 minutes incubation at 25 °C. Raw anisotropy data from the reader were plotted against concentration using GraphPad Prism software (version 10.0.2, GraphPad, USA) and nonlinear fitting "*[Inhibitor] vs. response -- Variable slope (four parameters)*" analysis was used to calculate the IC_{50} values (concentration, where half of the inhibition is achieved) for each titration. The IC_{50} values from each titration experiment were then used to calculate inhibition constant K_i using an online calculator (http://websites.umich.edu/~shaomengwanglab/software/calc_ki/index.html).[11] The titration curves are shown in the Figs. S3–S8. The triplicate measurements for each compound are shown in one graph using the error bars. The calculated K_i of standard **Lac** as well as compounds **11-Cl**, **11-PF** and **12-Cl** are shown in Table S2.

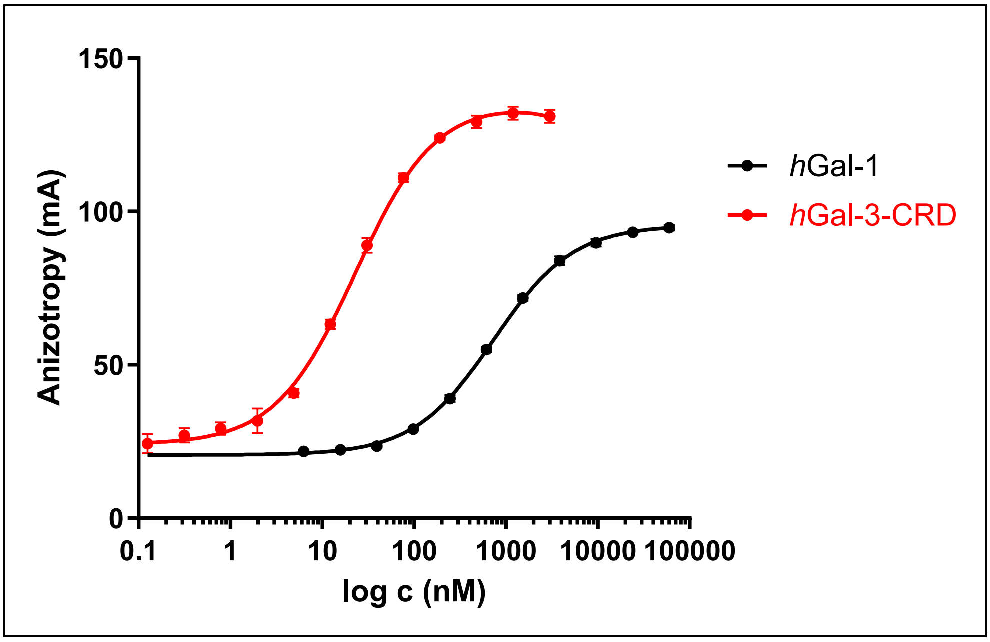

Figure S2. Direct fluorescence polarization titration curves of the fluorescence probe with *h*Gal-1 and *h*Gal-3-CRD.

Table S1: Calculated K_D (nM) of the fluorescence probe to *h*Gal-1 and *h*Gal-3-CRD.

	***h*Gal-1**	***h*Gal-3-CRD**
**K_D (nM)**	734	23.1

Table S2. Apparent inhibition constant K_i, relative potency *rp*[a] and selectivity values of inhibitors derived from **Lac** determined by competitive fluorescence polarization.

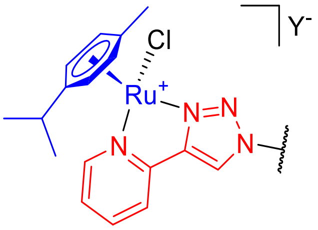

Ru^Cl Y = Cl
Ru^PF Y = PF_6

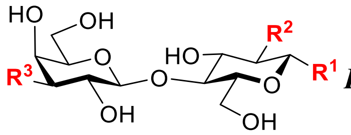

**Compound**	**R¹**	**R²**	**R³**	***K*i (*h*Gal-1) [μM]**	***rp* (*h*Gal-1)**	***K*i (*h*Gal-3-CRD) [μM]**	***rp* (*h*Gal-3-CRD)**	**Selectivity to *h*Gal-1**[b]
**Lac**[c]	OH	OH	OH	334 ± 28	1.0	94 ± 7	1.0	0.28
**11-Cl**	**Ru^Cl**	OH	OH	61 ± 8	5.5	65 ± 11	1.4	1.1
**11-PF**	**Ru^PF**	OH	OH	78 ± 6	4.3	68 ± 3	1.4	1.3
**12-Cl**	OMe	**Ru^Cl**	OH	16 ± 2	20.9	21 ± 1	4.5	1.4

[a] Relative potency was defined as $rp = K_i$(**Lac**)/K_i(inhibitor) for **Lac**-derived inhibitors, [b] defined as the ratio K_i(*h*Gal-3-CRD)/K_i(*h*Gal-1); [c] The values adopted from Ref.[12]

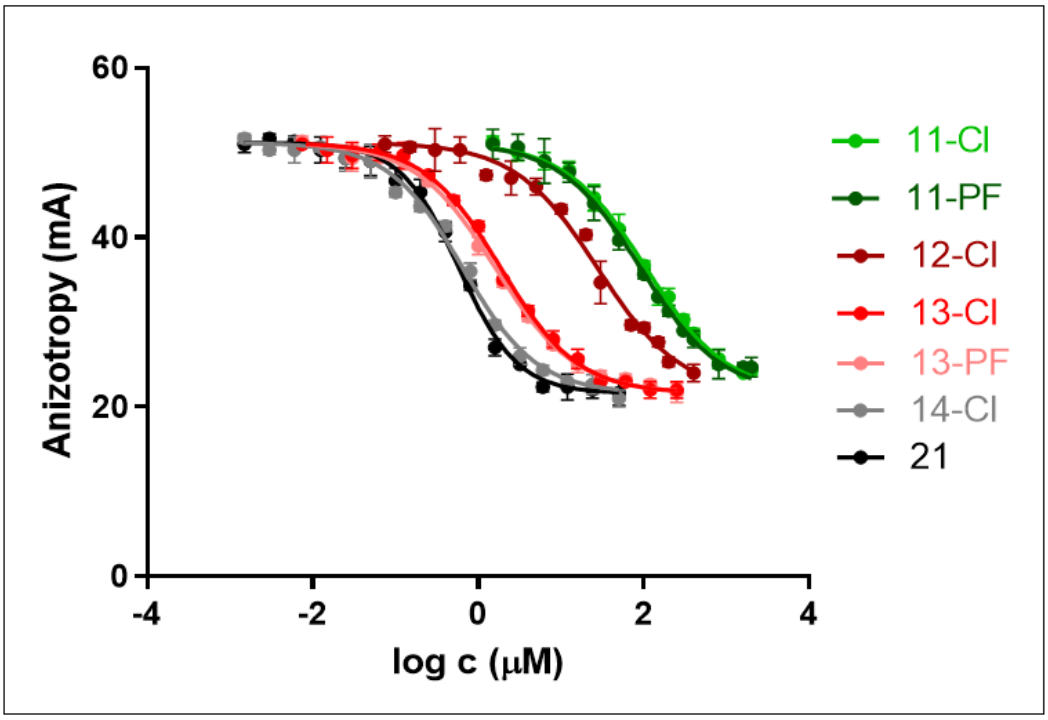

Figure S3: Fluorescence polarization titration curves of complexes **11–14** and non-organometallic compound **21** with *h*Gal-1.

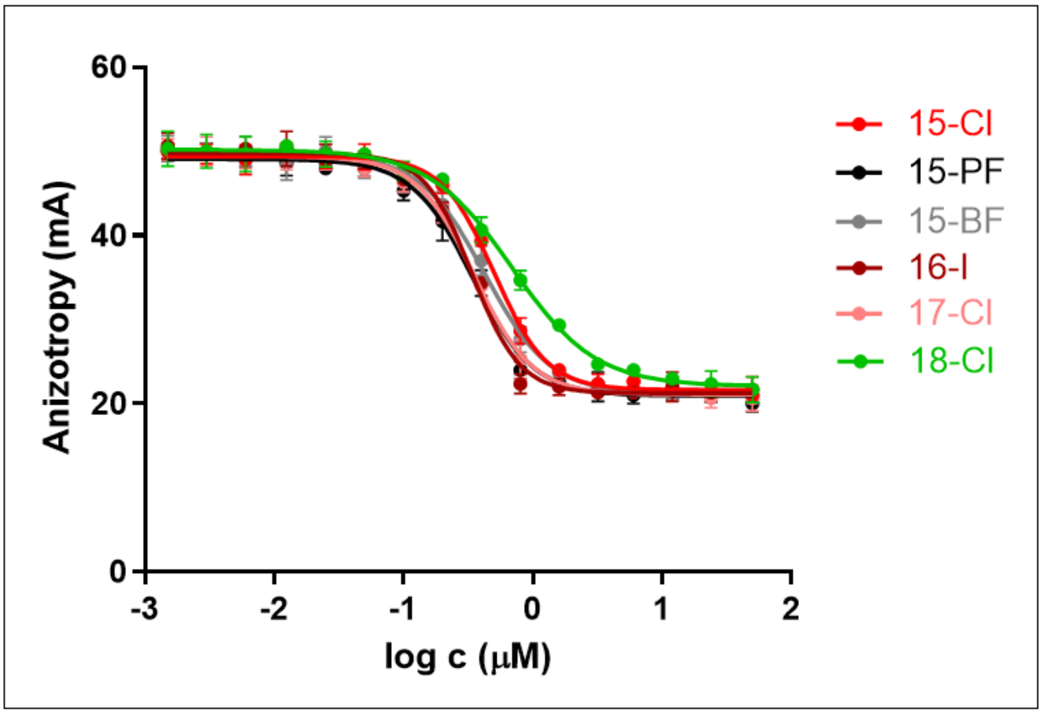

Figure S4: Fluorescence polarization titration curves of complexes **15–18** with *h*Gal-1.

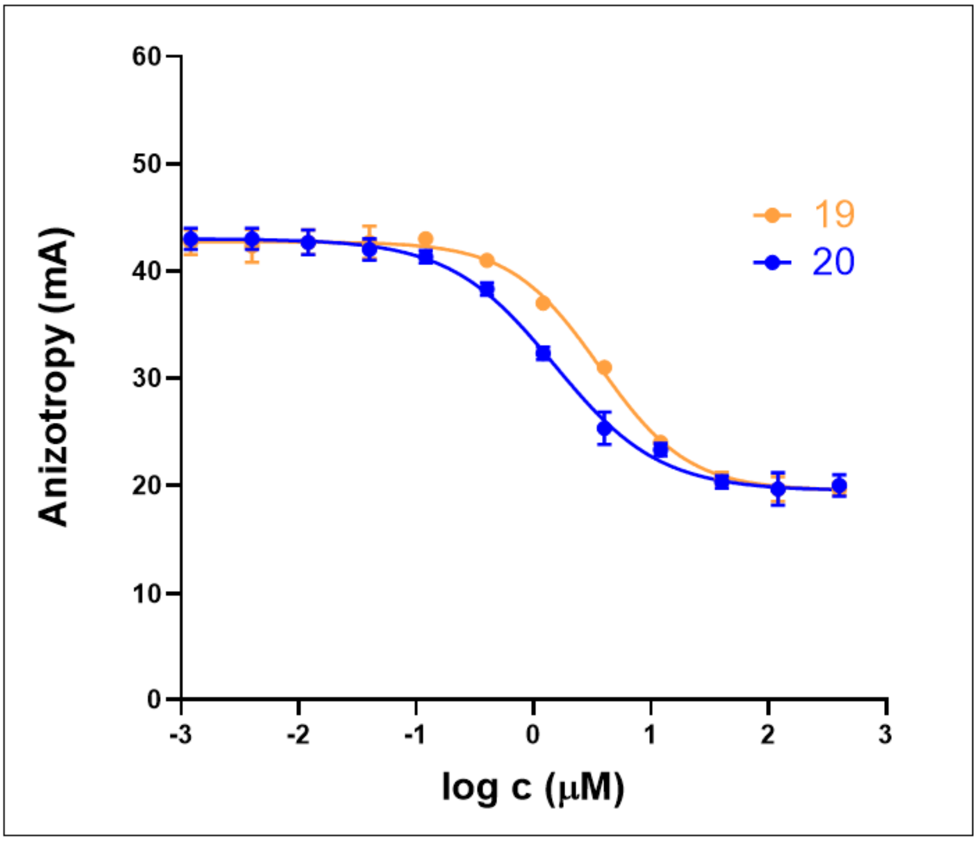

Figure S5: Fluorescence polarization titration curves of compounds **19** and **20** with *h*Gal-1.

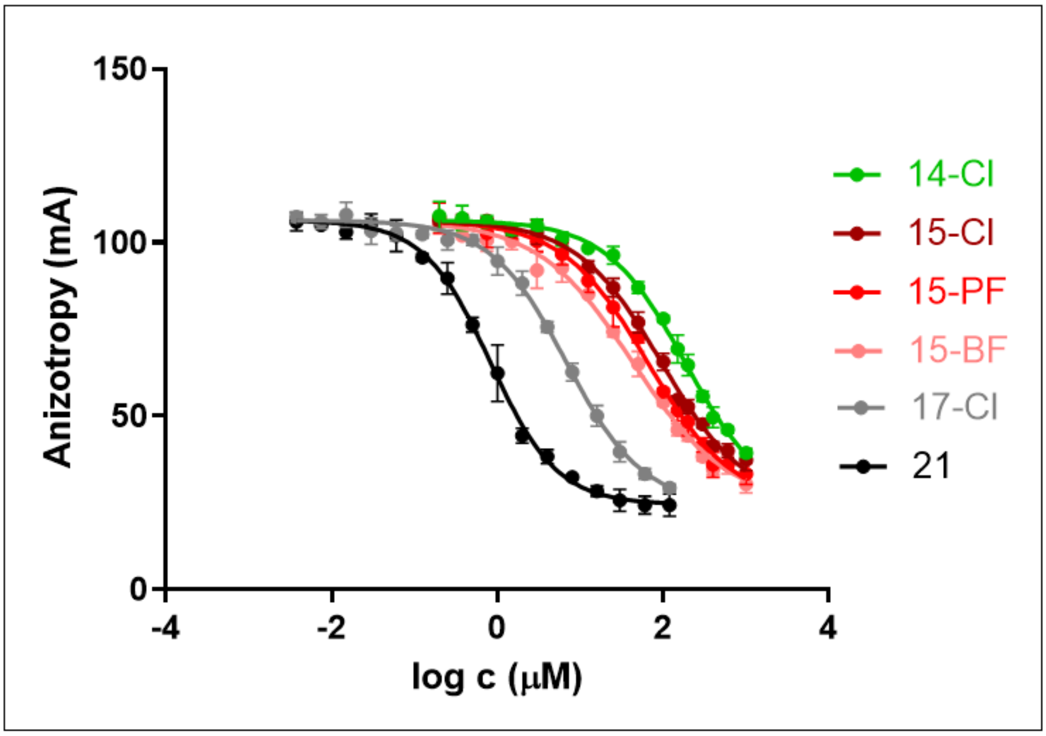

Figure S6: Fluorescence polarization titration curves of complexes **14–16** and nonorganometallic compound **21** with *h*Gal-3-CRD.

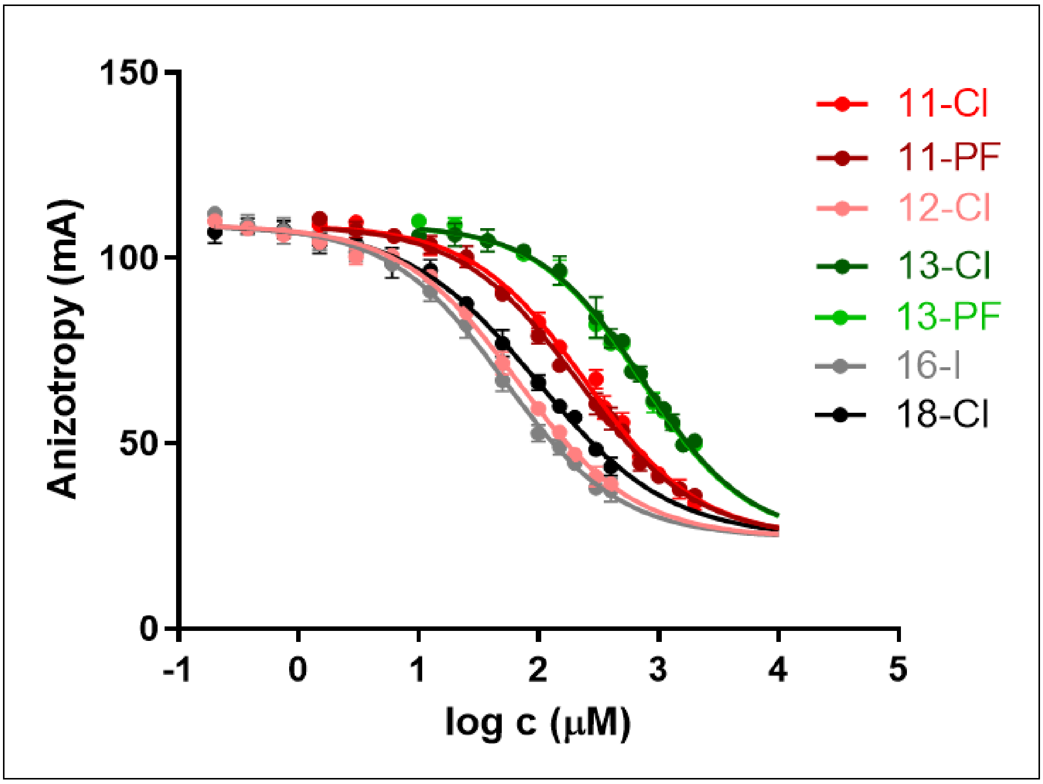

Figure S7: Fluorescence polarization titration curves of complexes **11–13** and **17–18** with *h*Gal-3-CRD.

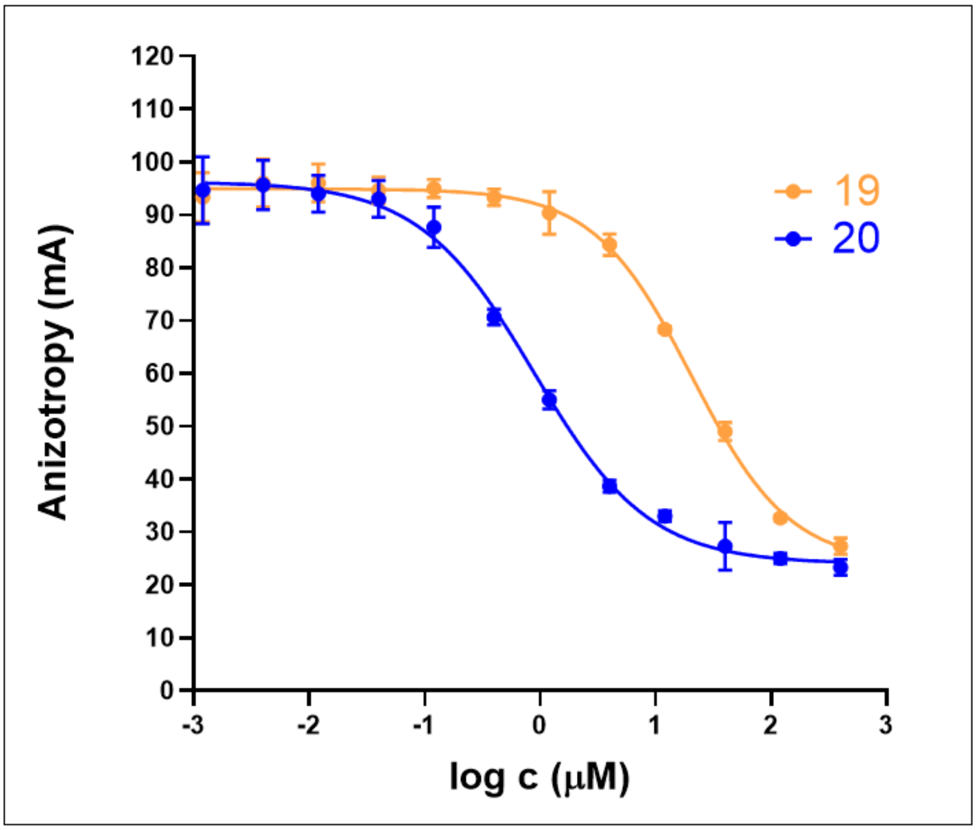

Figure S8: Fluorescence polarization titration curves of compounds **19** and **20** with *h*Gal-3-CRD.

Intrinsic tryptophan fluorescence

The excitation of 9 µM *h*Gal-1, resp. 3 µM *h*Gal-3-CRD, in phosphate-buffered saline (PBS) (pH = 7.4) with 6 mM trehalose, was done at 285 nm, and emission spectra were recorded from 302 to 400 nm. All emission spectra were registered using an ISS PC1 photon counting spectrofluorometer (ISS, USA) in a quartz cuvette with a 1 cm path length and at a temperature of 25 °C. The excitation and emission slit widths were fixed at 4 nm each. Fluorescence intensity was normalized; the fluorescence intensity maximum of *h*Gal-1/*h*Gal-3-CRD with a saccharide ligand, I_A, was subtracted from the fluorescence intensity maximum of free galectin titrated with an adequate amount of PBS, I_B. The obtained value was divided by the fluorescence intensity max of free *h*Gal, i.e. $(I_B - I_A)/I_B$. The binding constant, K_D value, was calculated using the One Site -Specific Binding's model $(Y = B_{max} * X)/(K_D + X)$, where X is the ligand concentration, Y is the fluorescence intensity, B_{max} is the maximum specific binding and K_D is the equilibrium binding constant) in Origin Software.[13] The titration curves are shown in the Figs. S9–S16.

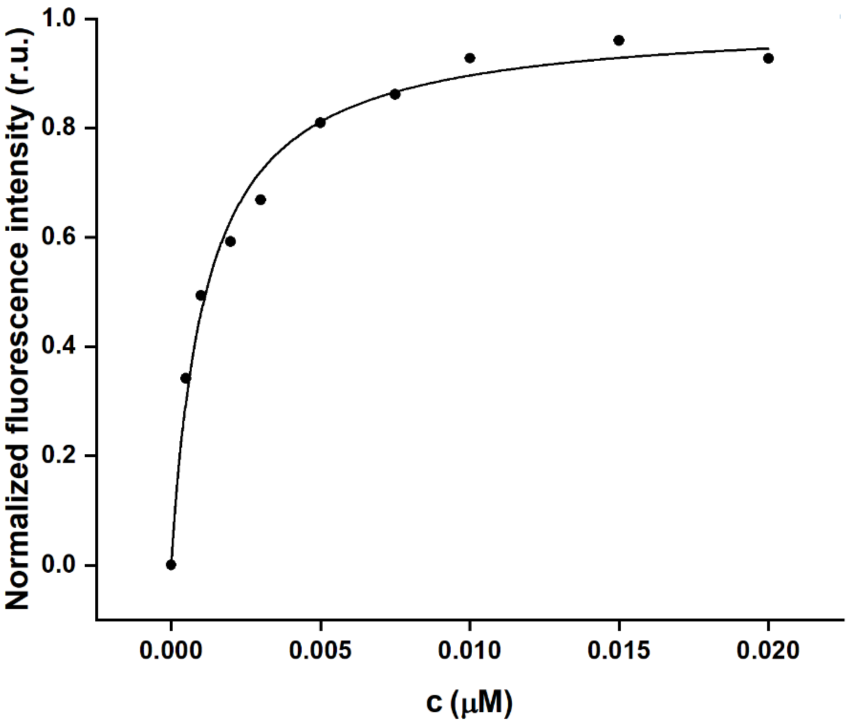

Figure S9: Normalized fluorescence intensity of *h*Gal-1 with **1**.

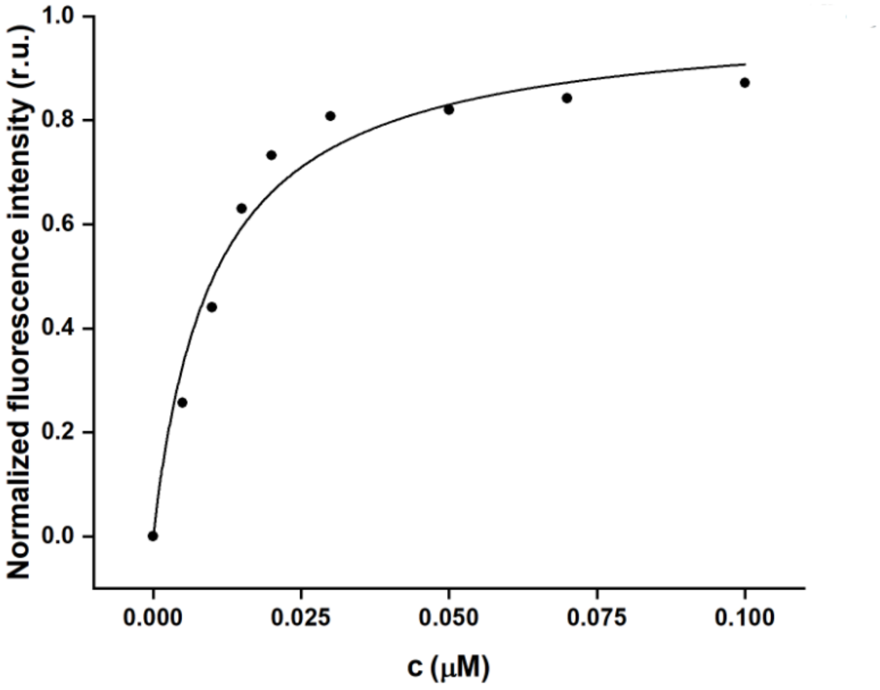

Figure S10: Normalized fluorescence intensity of *h*Gal-3-CRD with **1**.

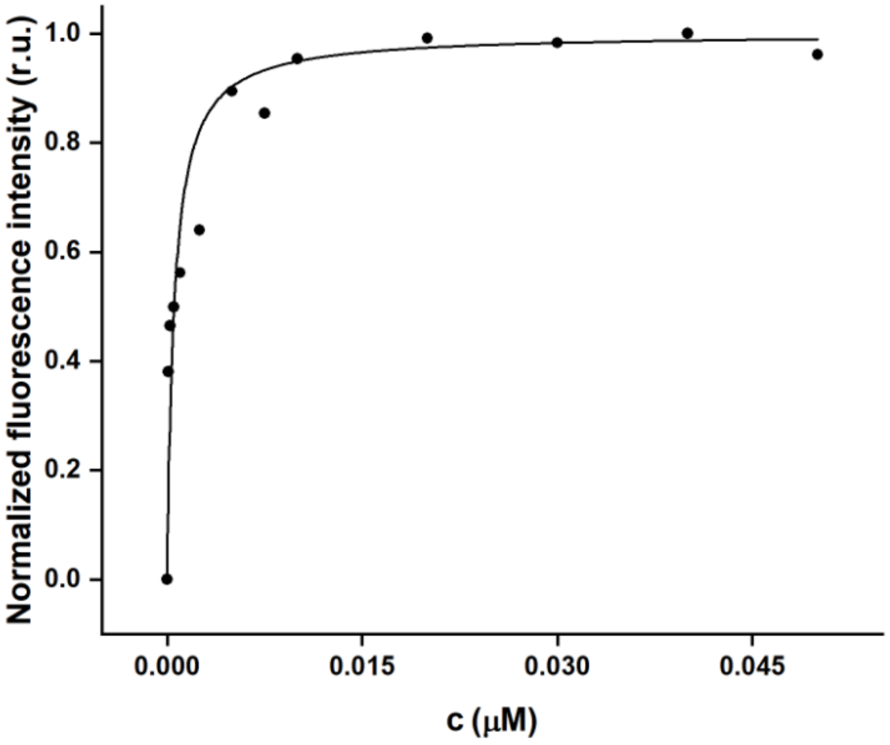

Figure S11: Normalized fluorescence intensity of *h*Gal-1with **15-PF**.

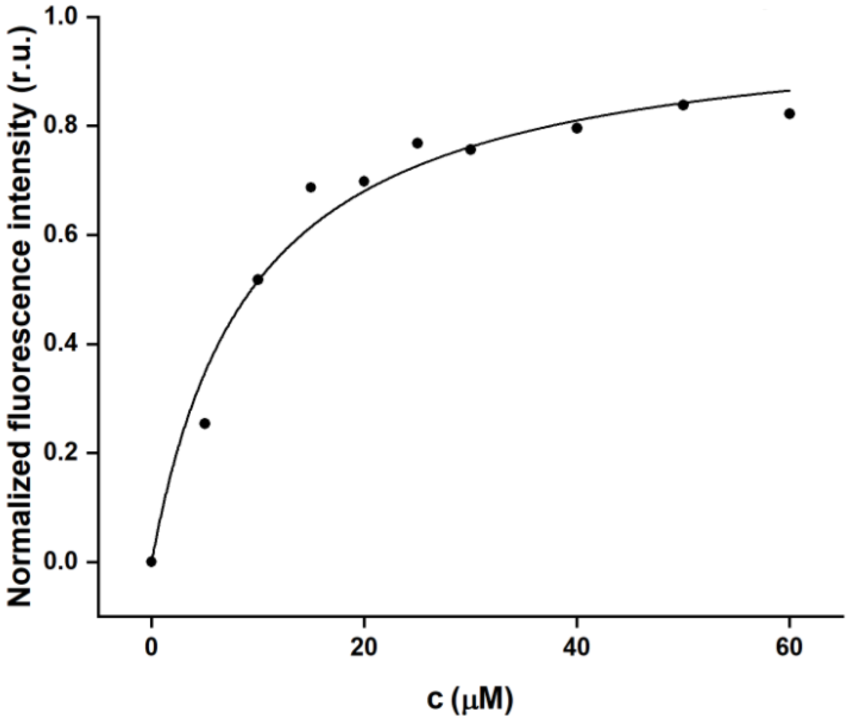

Figure S12: Normalized fluorescence intensity of *h*Gal-3-CRD with **15-PF**.

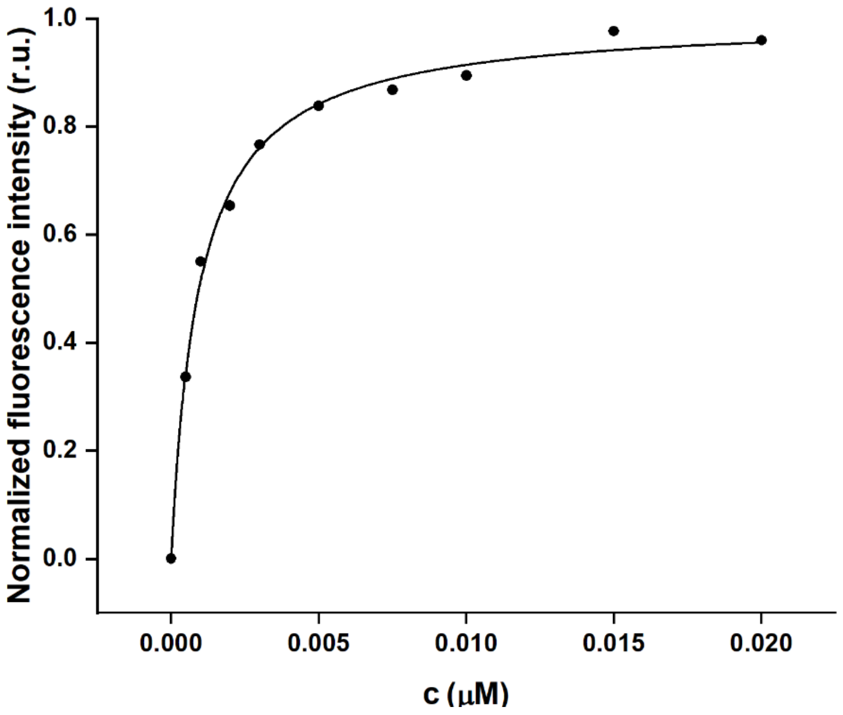

Figure S13: Normalized fluorescence intensity of *h*Gal-1with **15-BF**.

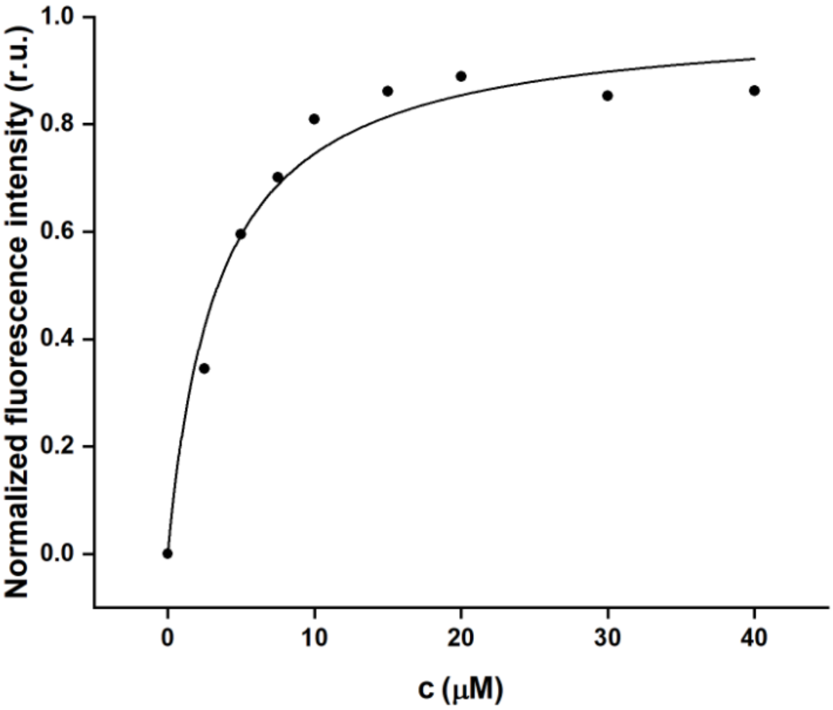

Figure S14: Normalized fluorescence intensity of *h*Gal-3-CRD with **15-BF**.

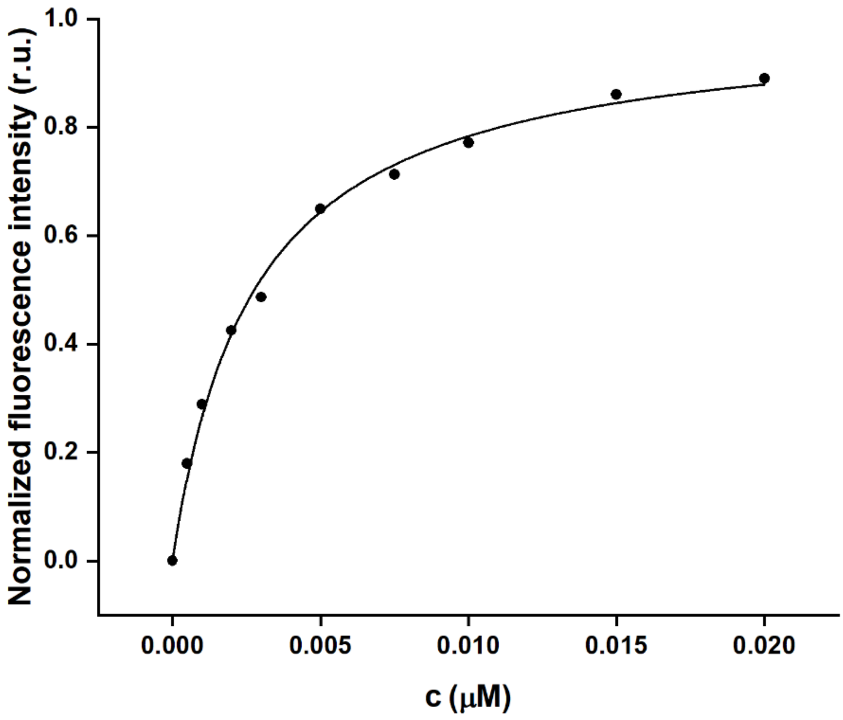

Figure S15: Normalized fluorescence intensity of *h*Gal-1with **17-Cl**.

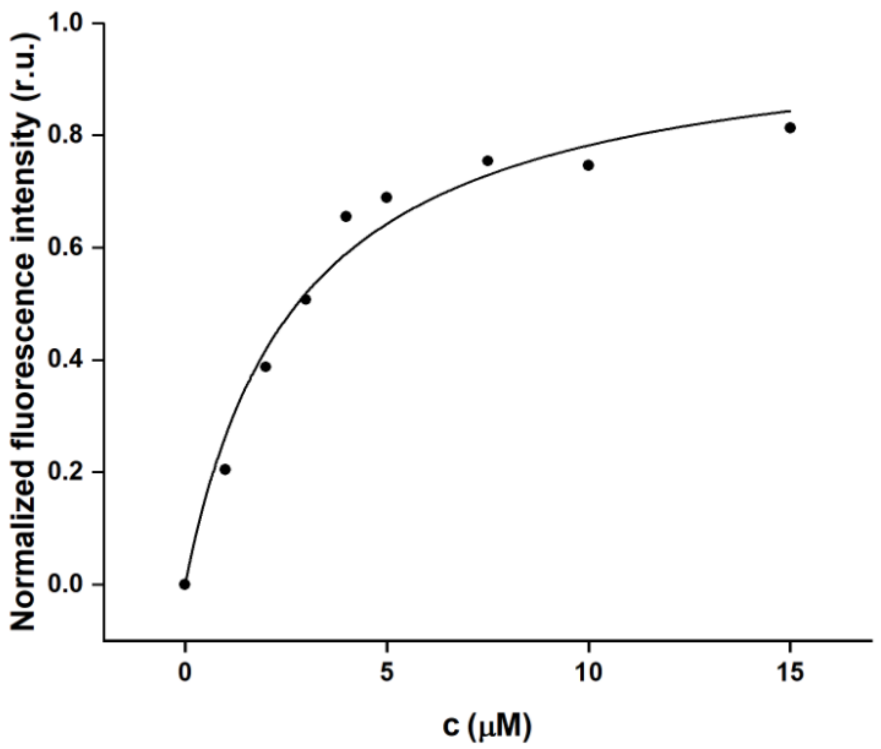

Figure S16: Normalized fluorescence intensity of *h*Gal-3-CRD with **17-Cl**.

Bio-layer interferometry

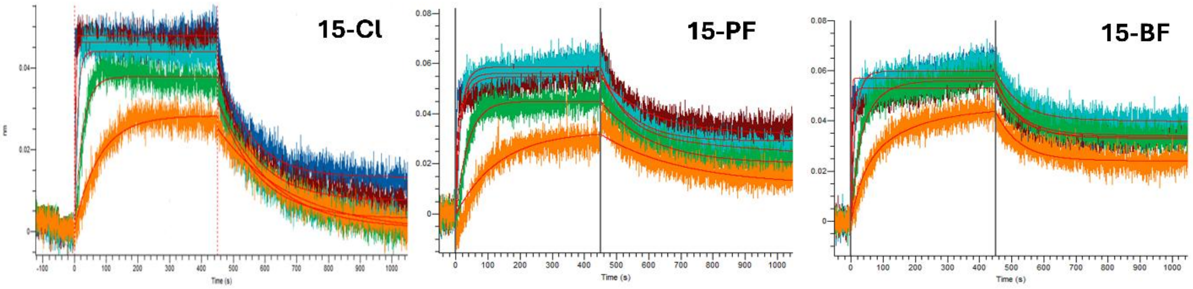

Figure S17: Sensograms of kinetic analysis of *h*Gal-1 with **15-Cl, 15-PF, and 15-BF**.

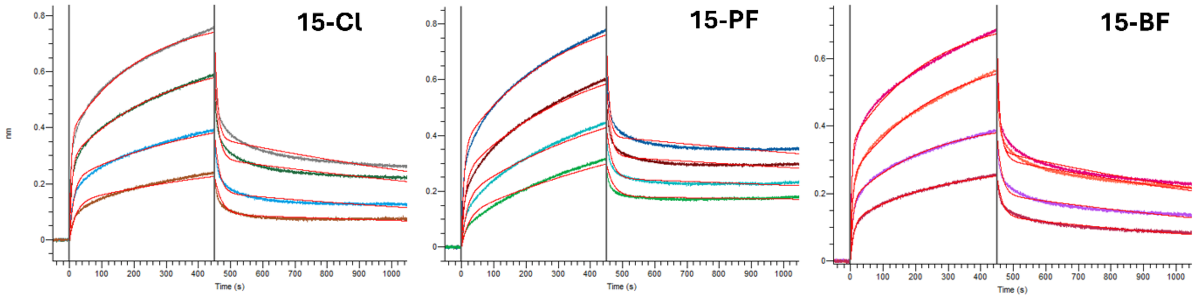

Figure S18: Sensograms of kinetic analysis of *h*Gal-3 with **15-Cl, 15-PF**, and **15-BF**.

E. Inhibition of galectin binding to cancer cells.

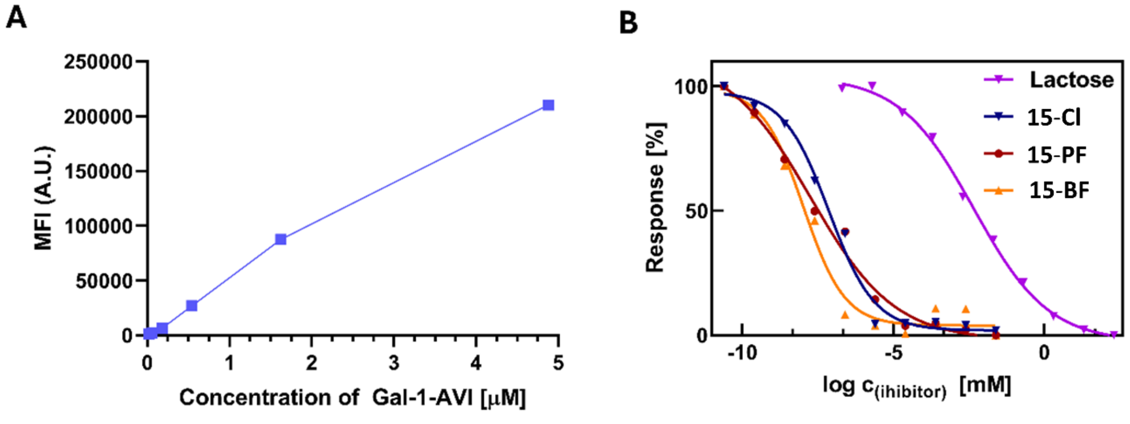

Figure S19: A. Direct binding of biotinylated construct Gal-1-AVI to the surface of MDA-MB-231 cell line. Varying concentrations of Gal-1-AVI in PBS/ BSA buffer (50 mM NaH_2PO_4/ 150 mM NaCl/ 1% *v/w* BSA, pH 7.5; total volume 100 μL) were added to the suspension of MDA-MB-231 cells (7.5^{4} cells/well) and incubated on ice for 1 h. The cells were washed with PBS/BSA buffer, and the surface-bound AVI-tagged galectin was labelled with the streptavidin-phycoerythrin conjugate (1/250; 100 μL; eBioscience, ThermoFisher Scientific, Carlsbad, CA, USA). The strength of binding of AVI-tagged galectin to the surface of MDA-MB-231 cells was analyzed by flow cytometry and quantified as a mean

fluorescence intensity at 575 nm. At least three independent experiments were performed in duplicate. **B.** Dose-response curves of competitive inhibition of competitive inhibition of binding of Gal-1-AVI to the surface of MDA-MB-231 cells by selected ruthenium-based glycomimetics. Compound **15-CL** is shown in **blue**, **15-PF** in **red** and **15-BF** in **orange**.

F. Jurkat apoptosis assay

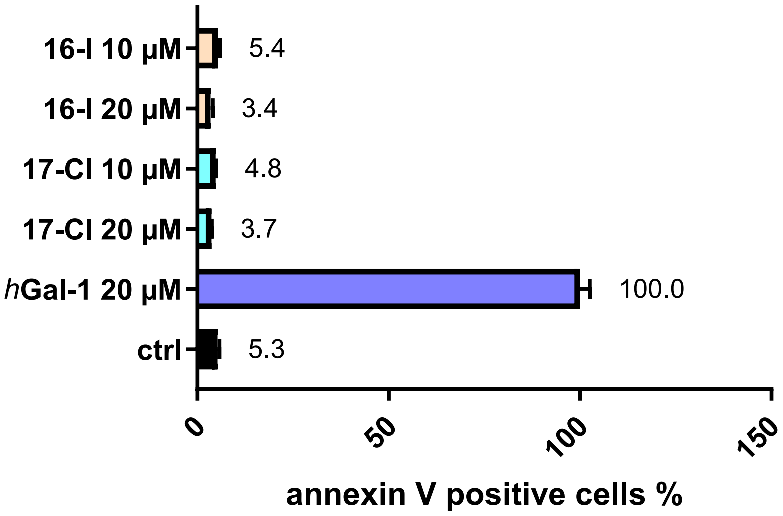

Figure S20: Inhibition of *h*Gal-1 induced apoptosis in Jurkat cells by glycomimetic inhibitors **16-I** and **17-Cl**. Apoptosis was assessed by flow cytometry after Annexin V-Alexa Fluor 488 staining and it is normalized to apoptosis level caused by *h*Gal-1 (20 µM) alone. The data represent the mean ± SD of five independent experiments.

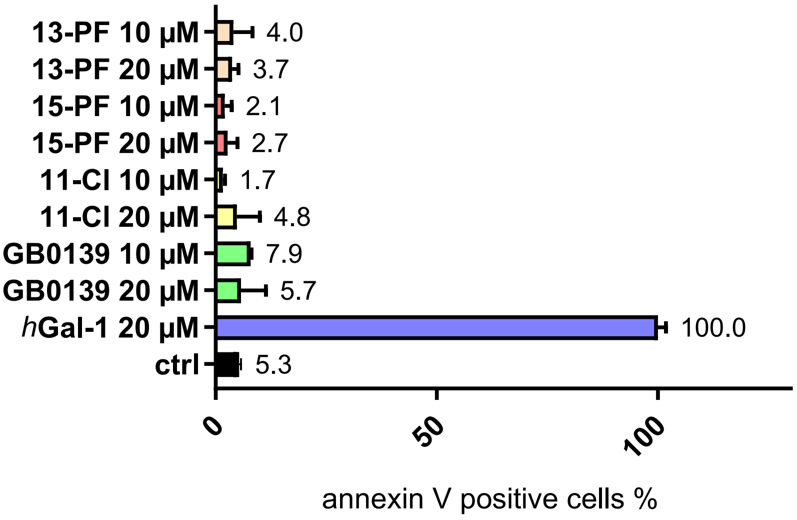

Figure S21: Apoptosis of Jurkat cells treated with glycomimetics only. Apoptosis was assessed by flow cytometry after Annexin V-Alexa Fluor 488 staining and it is normalized to apoptosis level caused by *h*Gal-1 (20 µM) alone. The data represent the mean ± SD of three independent experiments.

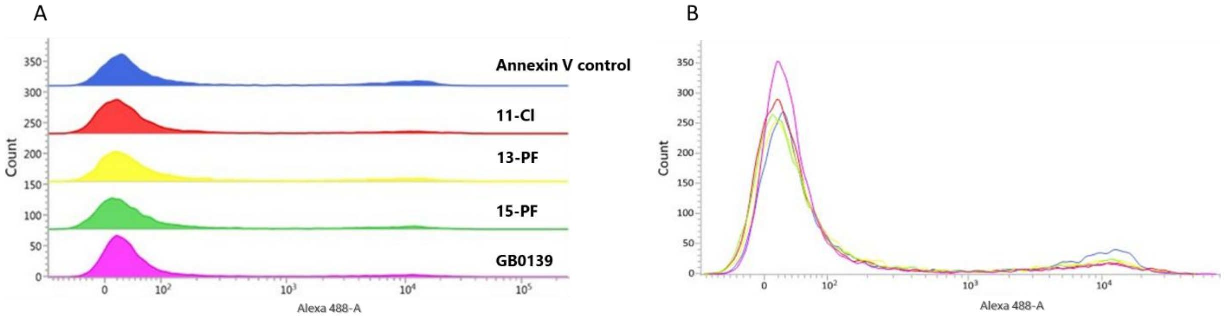

Figure S22: Representative histograms illustrating Annexin V-Alexa Fluor 488 staining of treatment with glycomimetic inhibitors alone. The x-axis represents the intensity of Alexa Fluor 488 fluorescence corresponding to Annexin V binding, and the y-axis indicates the number of events (number of cells). Each histogram (A) or curve (B) represents an individual control treated with the glycomimetic inhibitor at 20 µM concentration.

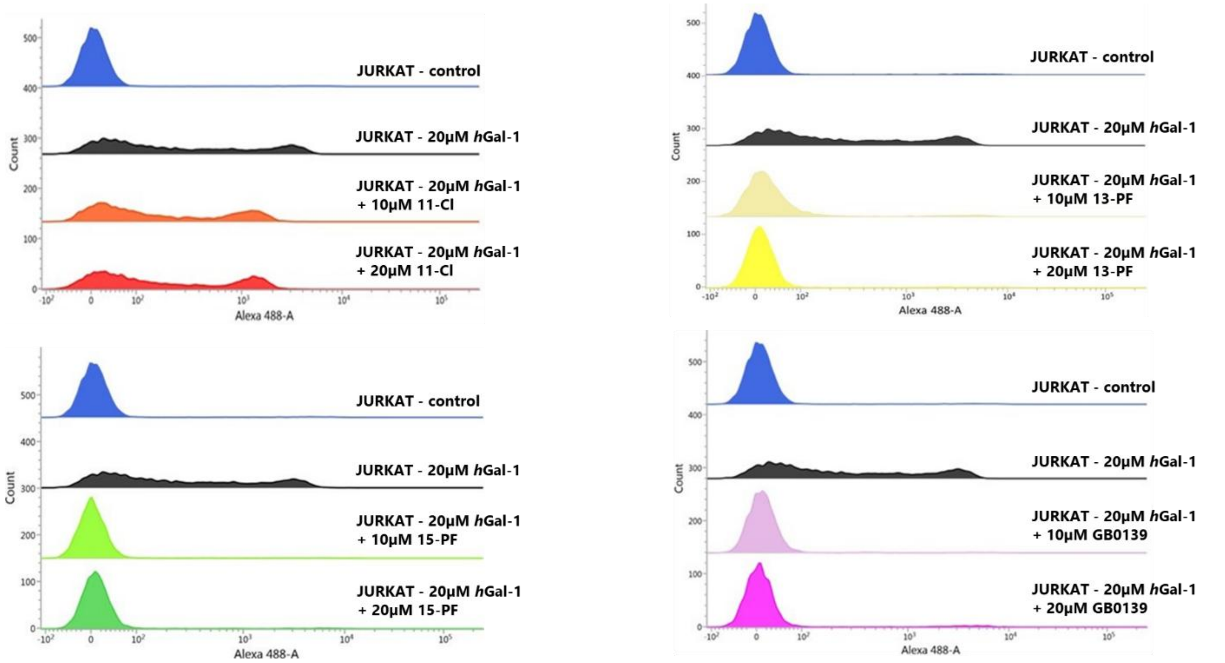

Figure S23: Representative flow cytometry histograms of Jurkat cells stained with Annexin V-Alexa Fluor 488. The x-axis represents the intensity of Alexa Fluor 488 fluorescence corresponding to Annexin V binding, and the y-axis indicates the number of events (number of cells). Cells were treated with TRIS-NaCl solvent (blue), *h*Gal-1 alone (black), or of *h*Gal-1 combined with inhibitors (**11-Cl**, **13-PF**,

15-PF, and **GB0139**) at two concentration ratios (1:0.5 and 1:1). A rightward shift in fluorescence intensity indicates increased Annexin V binding, corresponding to phosphatidylserine externalization on the outer leaflet of the plasma membrane - an early marker of apoptosis. *h*Gal-1 treatment alone induces a clear shift, whereas co-treatment with most inhibitors prevents this shift, suggesting effective inhibition of *h*Gal-1 activity. Only the inhibitor **11-Cl** fails to block this effect, as evidenced by the persistence of high Annexin V signal comparable to *h*Gal-1 alone.

G. Cytotoxicity testing

Both cell lines were obtained from American Type Culture Collection (ATCC), for the experiments we used cells in 10–20 passages. The cells were maintained at 37 °C in humidified atmosphere with 5% CO_2, and tested for mycoplasma contamination with negative results. MDA-MB-231 and HEK-293 cells were cultured in high glucose Dulbecco's modified Eagle's medium supplemented with 10% fetal bovine serum (Gibco, ThermoFisher Scientific, Waltham, MA, USA), 300 μg/mL of L-glutamine (Sigma-Aldrich), and 100 μg/mL of HyClone Penicillin– Streptomycin 100X solution (BioSera, Nuaille, France). The culture medium was changed during each cell passage every 2–3 days. Cells were mycoplasma free throughout the experiments. Cells were grown to 60–80% confluence prior to seeding in a 96-well plate at a density of 5000 cells/well for cytotoxicity testing. The next day, these cells were exposed to selected compounds at concentrations from 0.05 to 100 μM for 72 h. The cell viability was measured using colorimetric MTT assay as described previously.[14] Briefly, 20 μL per well of MTT (thiazolyl blue tetrazolium bromide, 2.5 mg/mL; Sigma-Aldrich Co., St. Louis, MO, USA) was added and incubated for 3 h under culture conditions. After 3 h the medium was removed, the formazan product was dissolved in 50 μL of DMSO (SERVA Electrophoresis GmbH, Heidelberg, Germany), and optical densities were measured at 595 nm using a microplate spectrophotometer reader (Tecan GENios, TECAN Austria GmbH). Data from MTT assay were analyzed in GraphPad Prism software. Errors were calculated as standard deviations (SD). All experiments were performed independently at least five times.

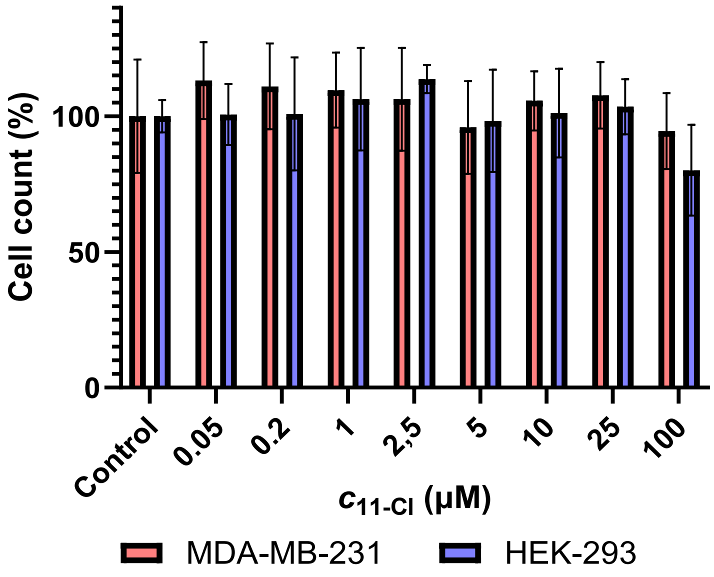

Figure S24: Cell viability of MDA-MB-231 and HEK-293 cells after 72 h treatment with **11-Cl.**

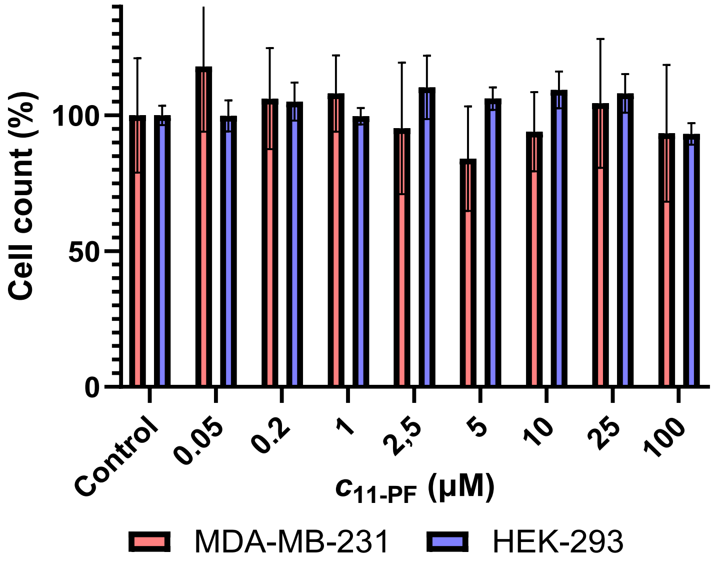

Figure S25: Cell viability of MDA-MB-231 and HEK-293 cells after 72 h treatment with **11-PF.**

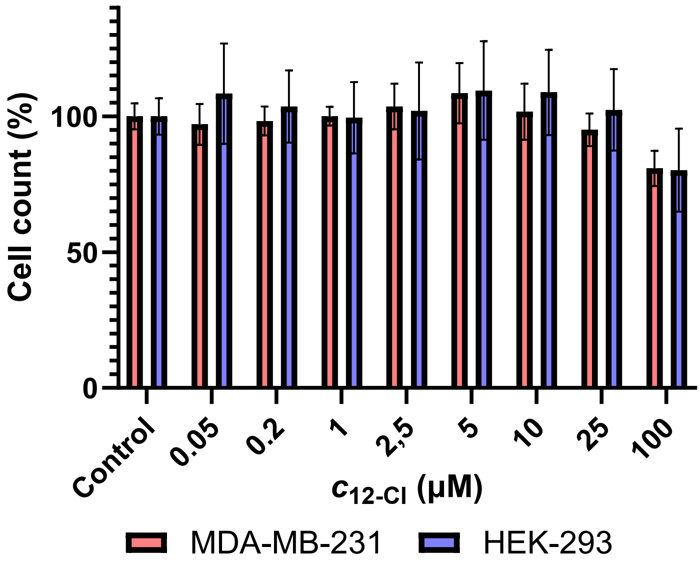

Figure S26: Cell viability of MDA-MB-231 and HEK-293 cells after 72 h treatment with **12-Cl.**

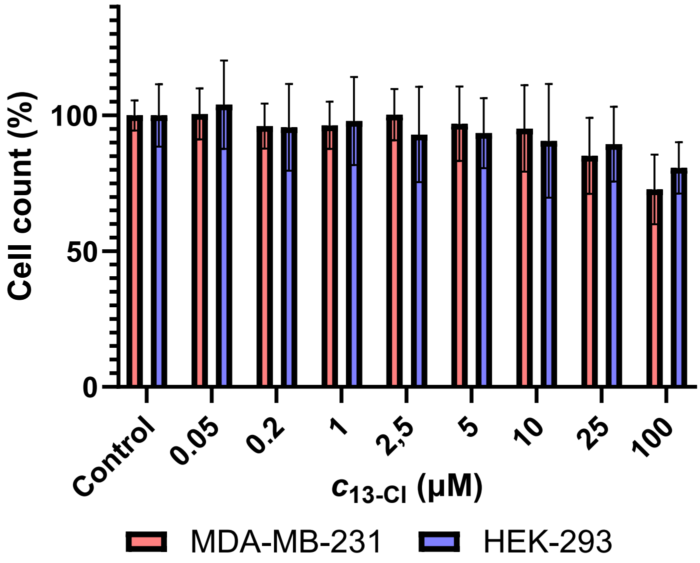

Figure S27: Cell viability of MDA-MB-231 and HEK-293 cells after 72 h treatment with **13-Cl.**

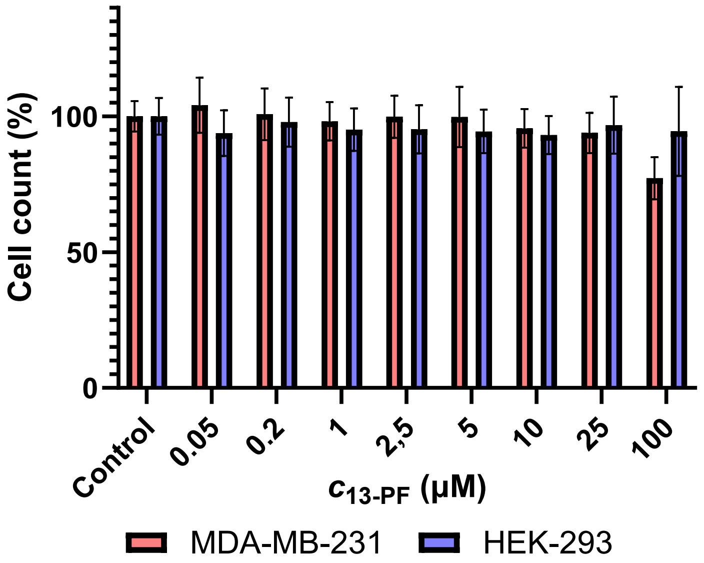

Figure S28: Cell viability of MDA-MB-231 and HEK-293 cells after 72 h treatment with **13-PF.**

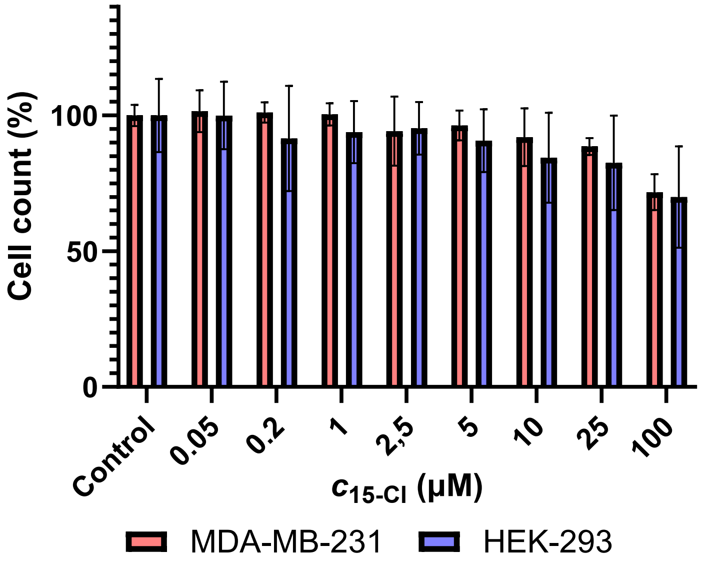

Figure S29: Cell viability of MDA-MB-231 and HEK-293 cells after 72 h treatment with **15-Cl.**

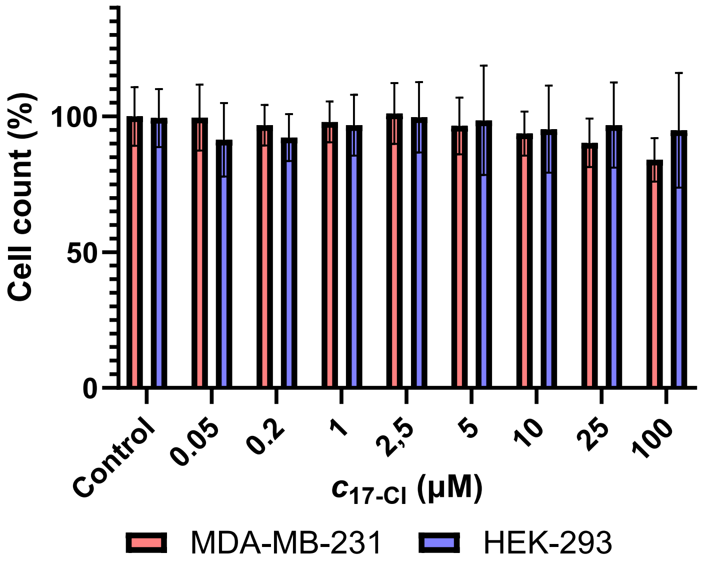

Figure S30: Cell viability of MDA-MB-231 and HEK-293 cells after 72 h treatment with **17-Cl.**

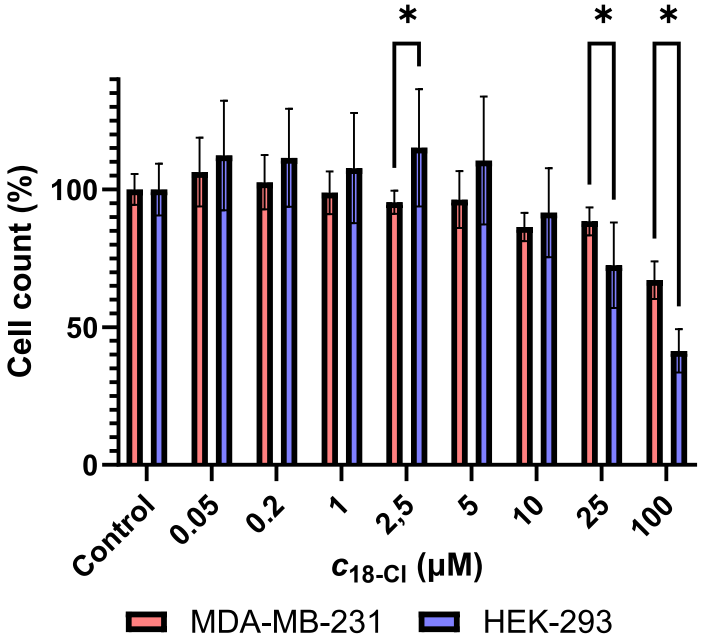

Figure S31: Cell viability of MDA-MB-231 and HEK-293 cells after 72 h treatment with **18-Cl.**
*$P < 0.01$

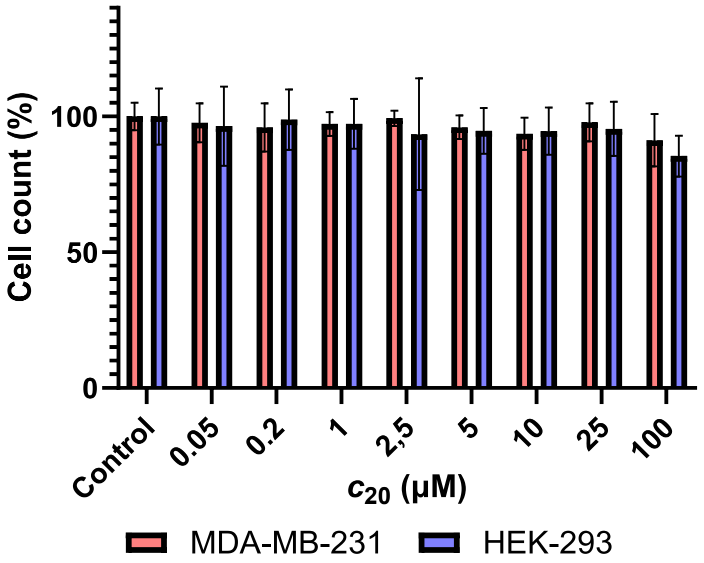

Figure S32: Cell viability of MDA-MB-231 and HEK-293 cells after 72 h treatment with **21.**

H. Copies of NMR spectra

NMR COMPOUND 11-Cl

^{1}H NMR (400 MHz, CD_3OD) 11-Cl

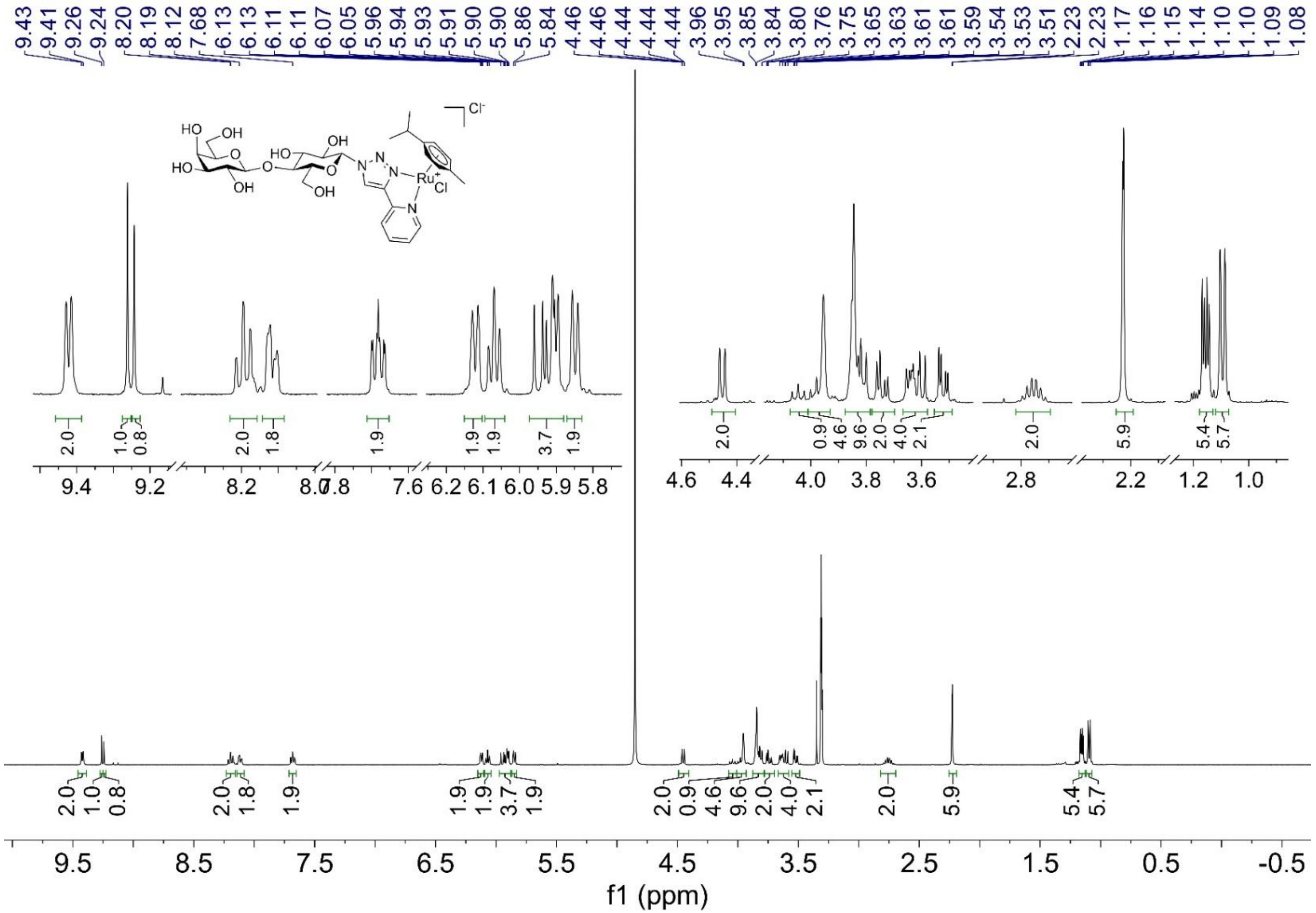

^{13}C NMR (101 MHz, CD$_3$OD) 11-Cl

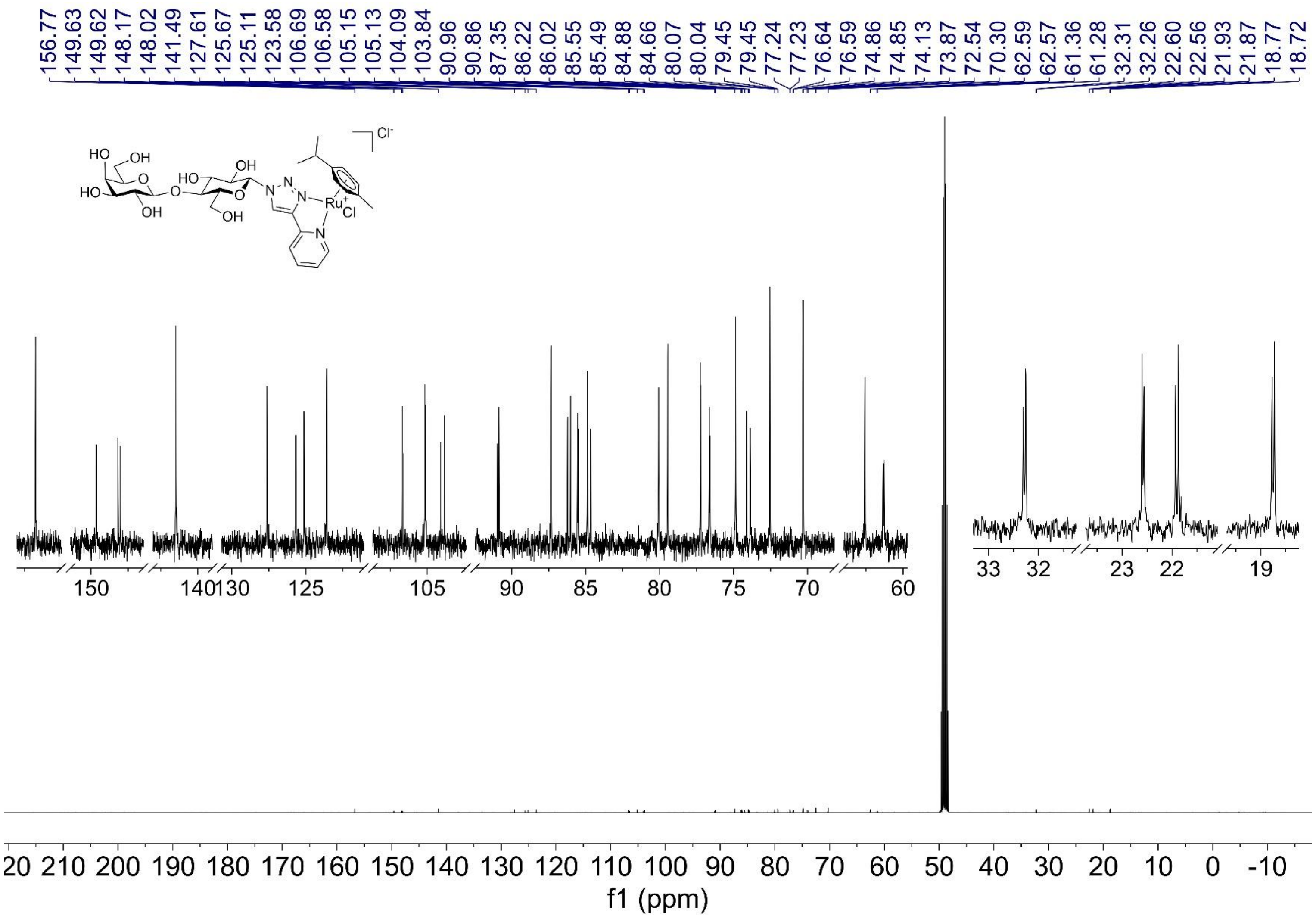

^{1}H-^{1}H COSY 11-Cl

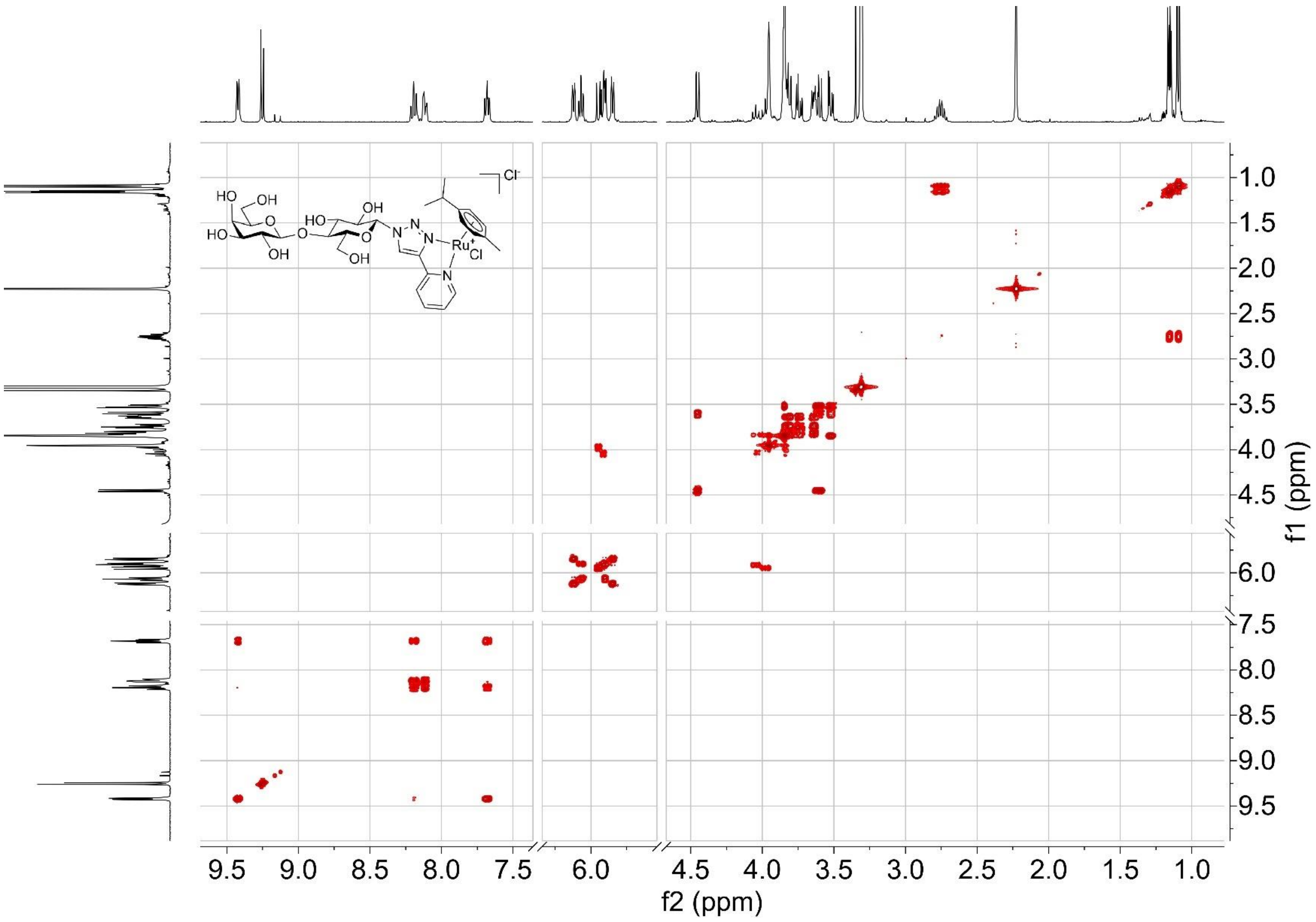

^{1}H-^{13}C HSQC 11-Cl

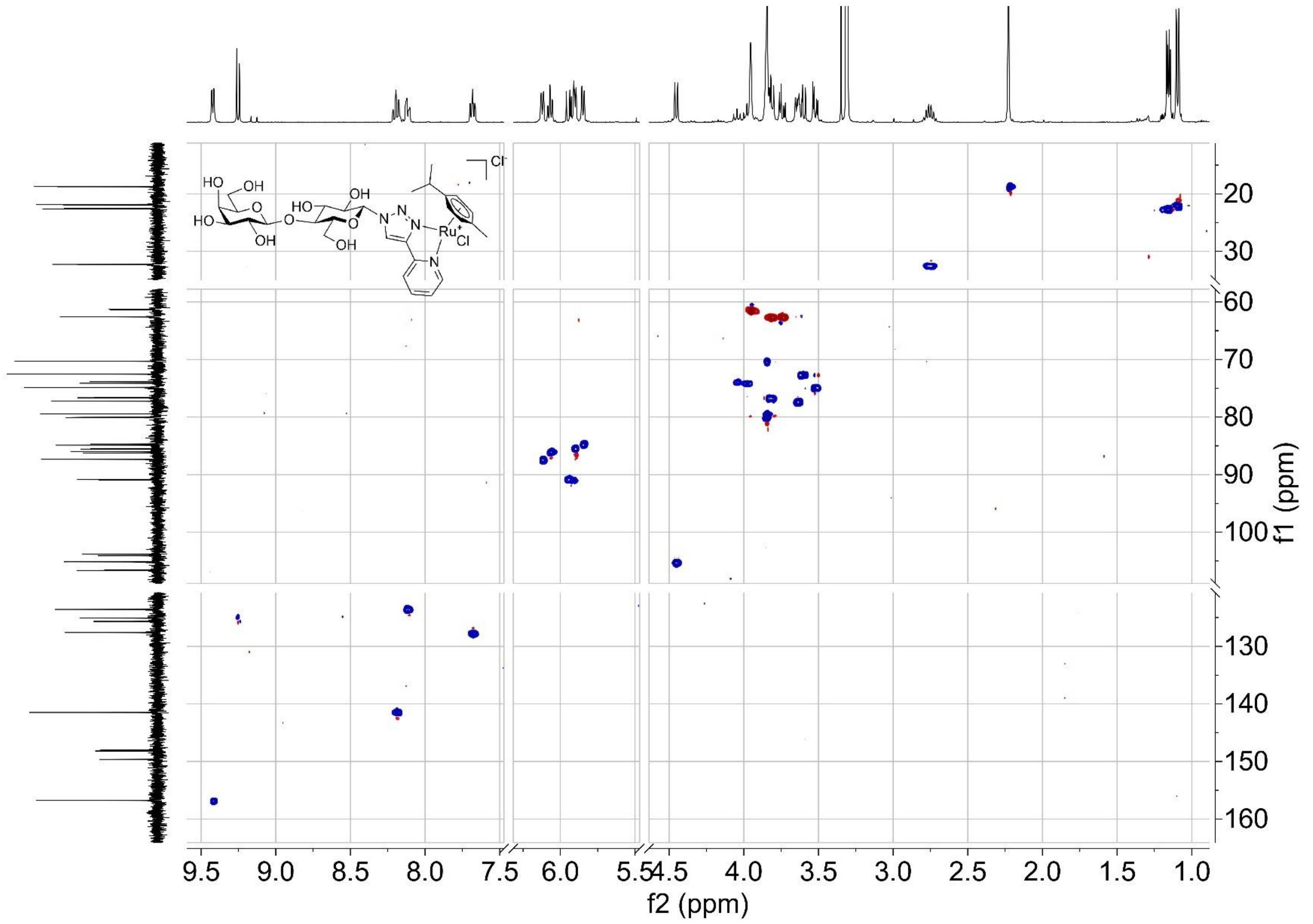

^{1}H-^{13}C HMBC 11-Cl

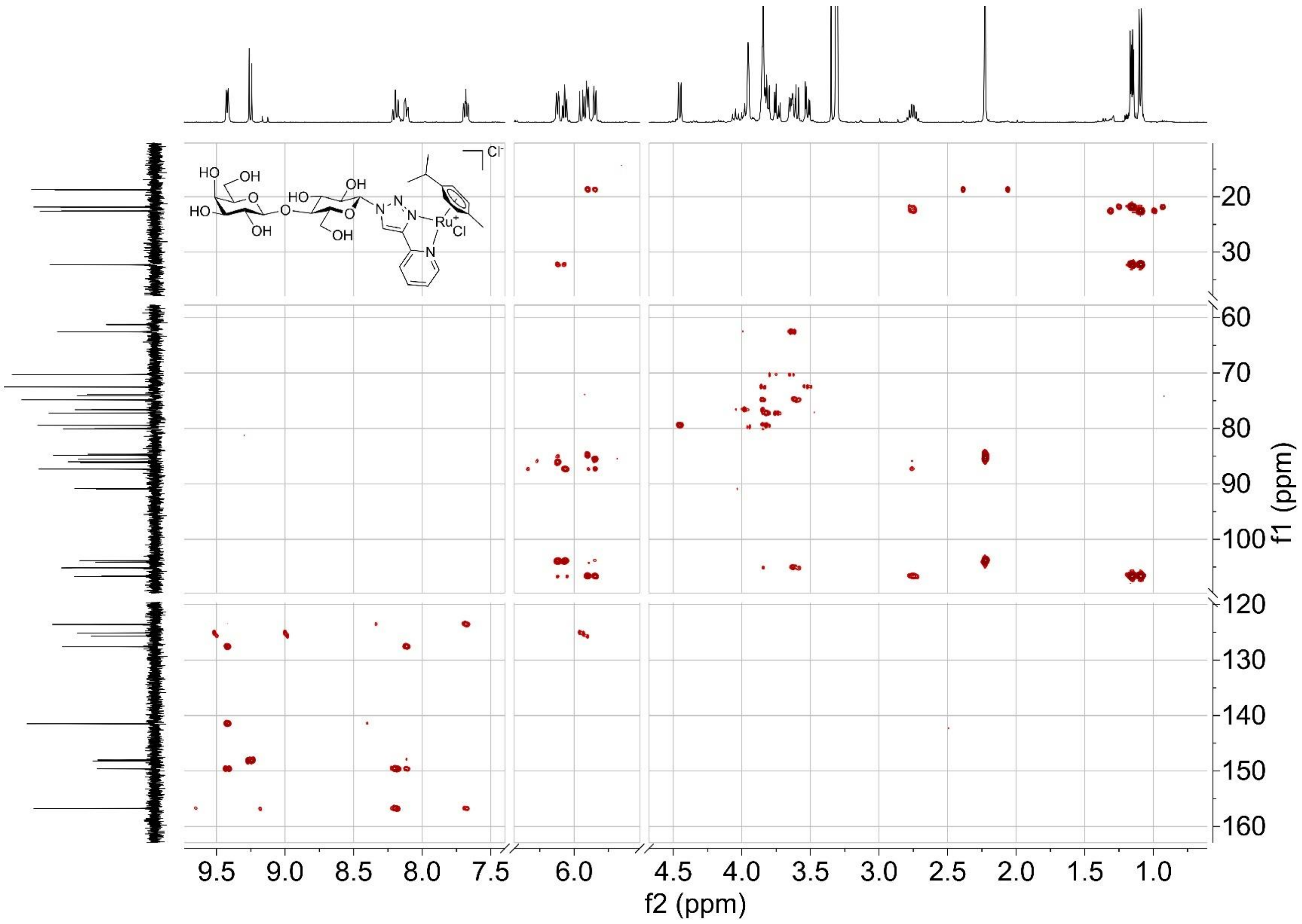

^{1}H-^{13}C HSQCTOCSY 11-Cl

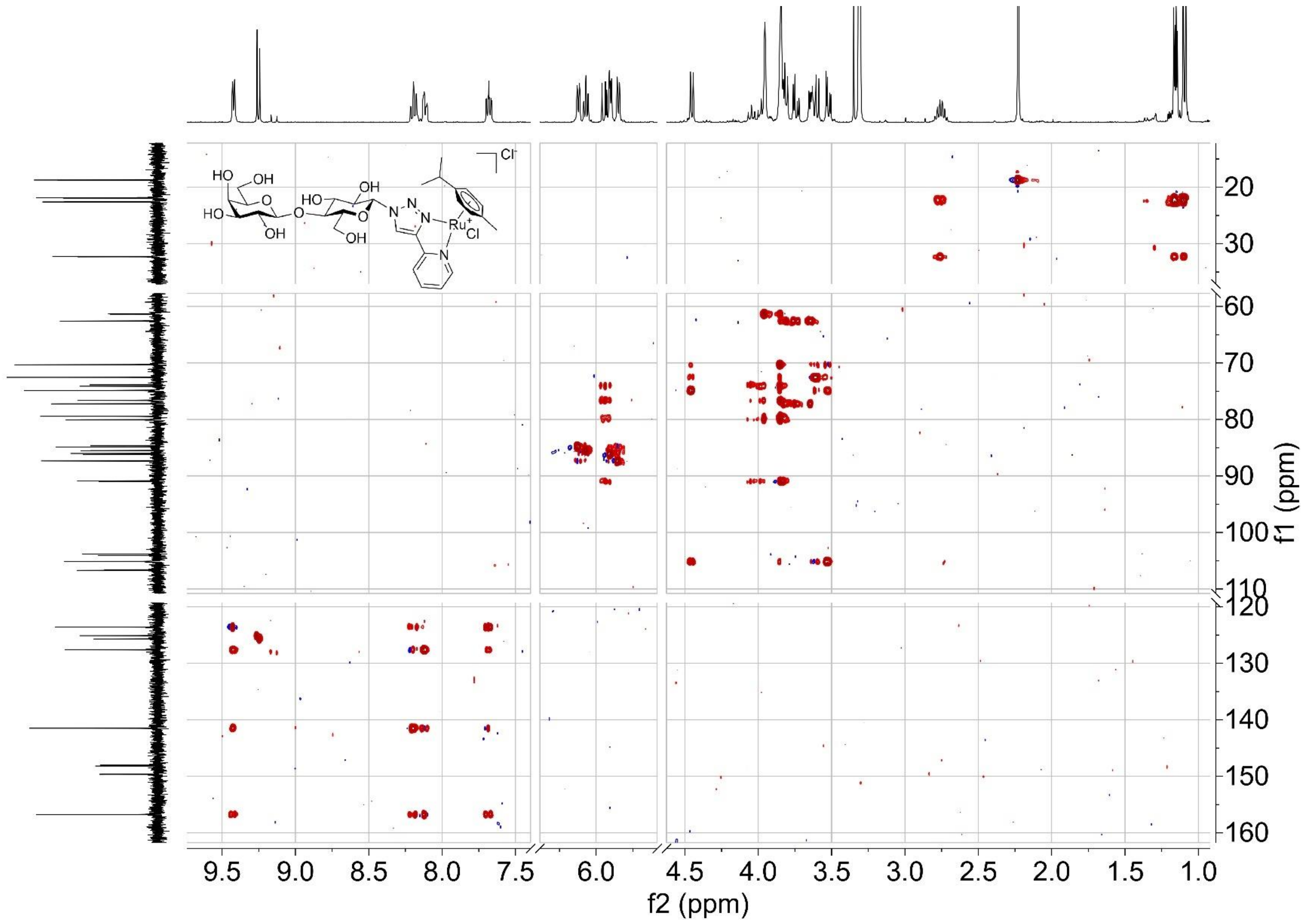

NMR COMPOUND 11-PF

^{1}H NMR (400 MHz, CD_3OD) 11-PF

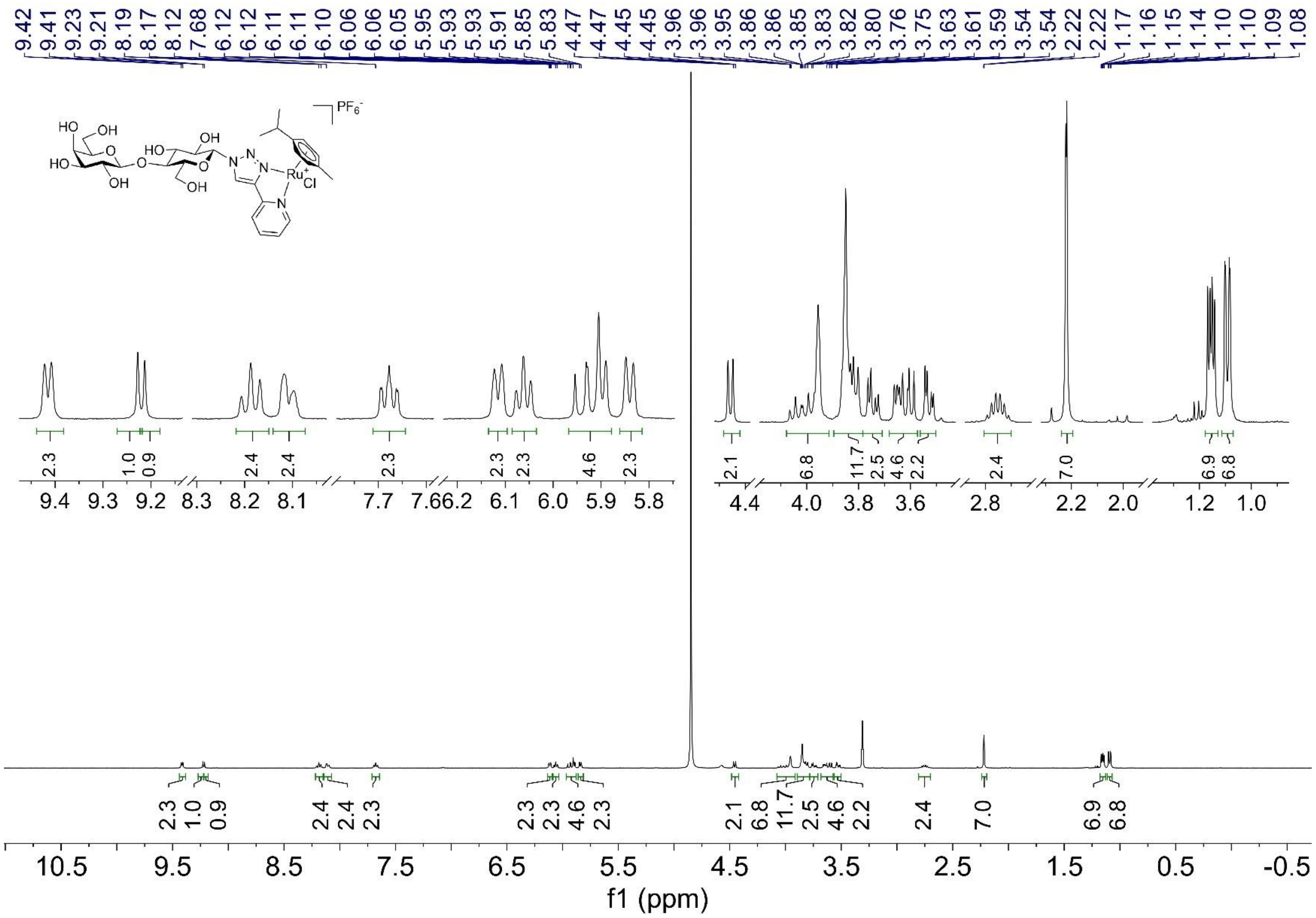

^{13}C NMR (101 MHz, CD_3OD) 11-PF

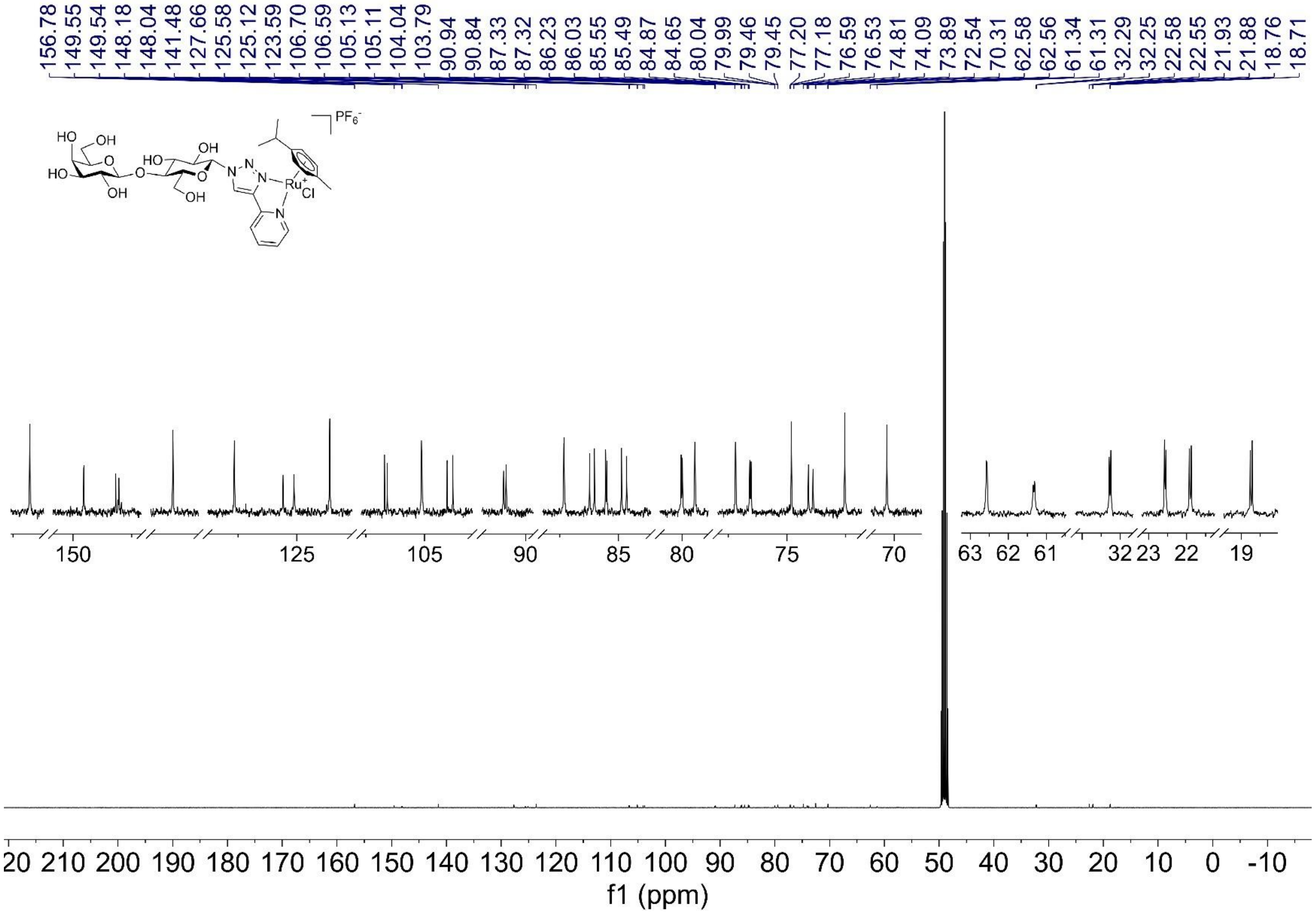

^{19}F NMR (376 MHz, CD_3OD) 11-PF

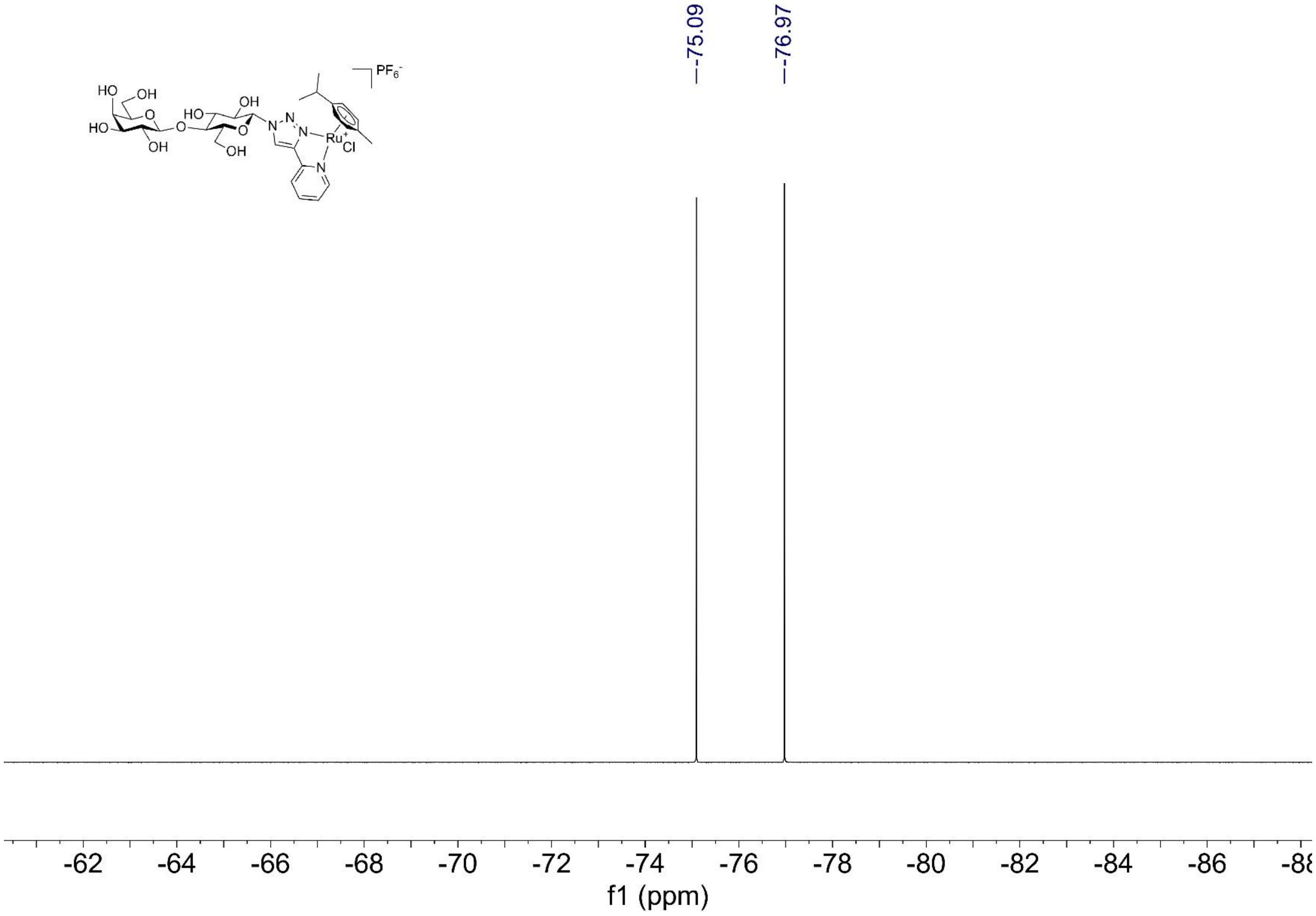

^{31}P NMR (162 MHz, CD_3OD) 11-PF

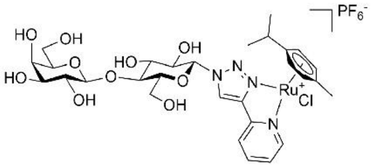

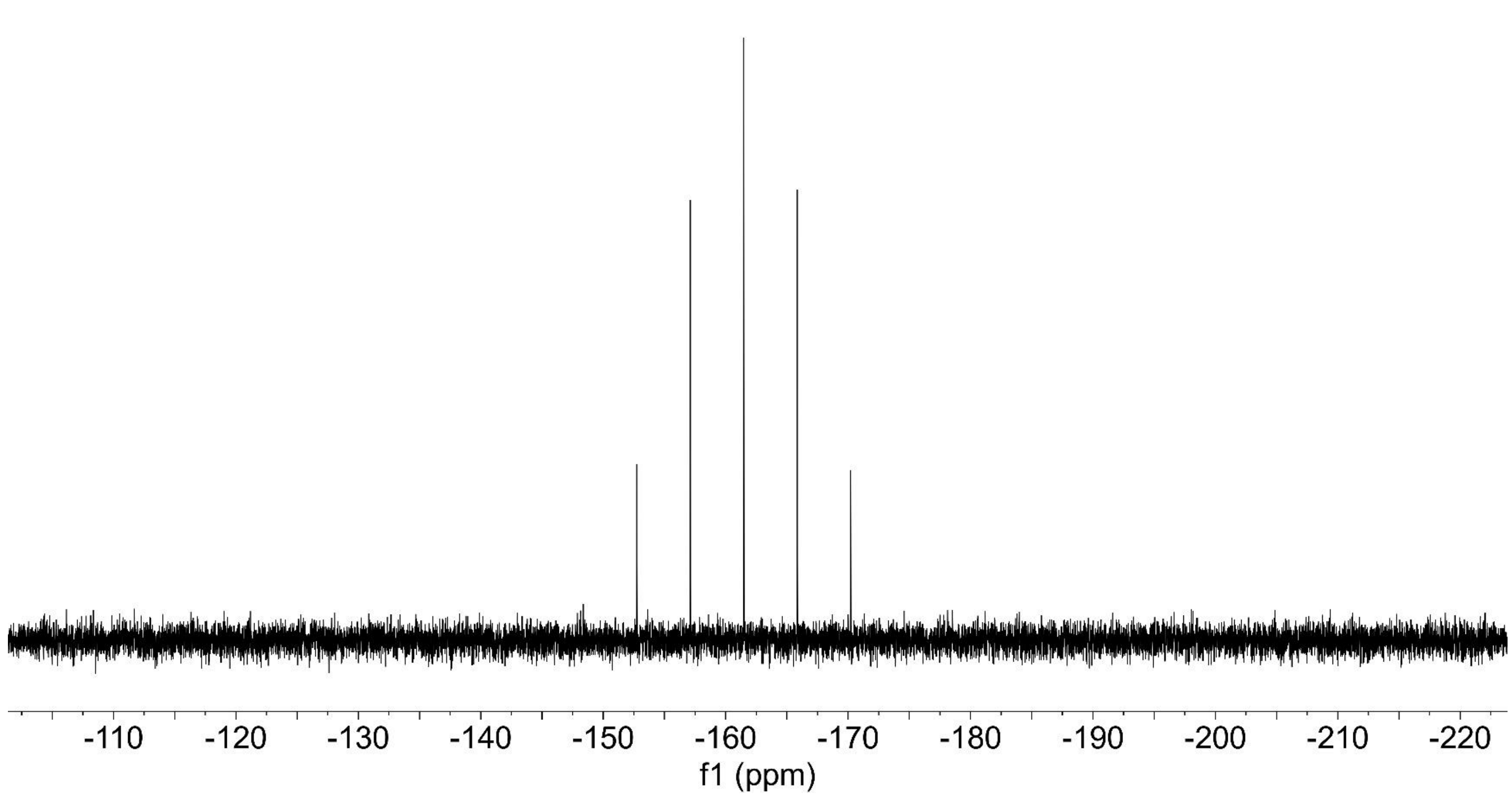

^{1}H-^{1}H COSY 11-PF

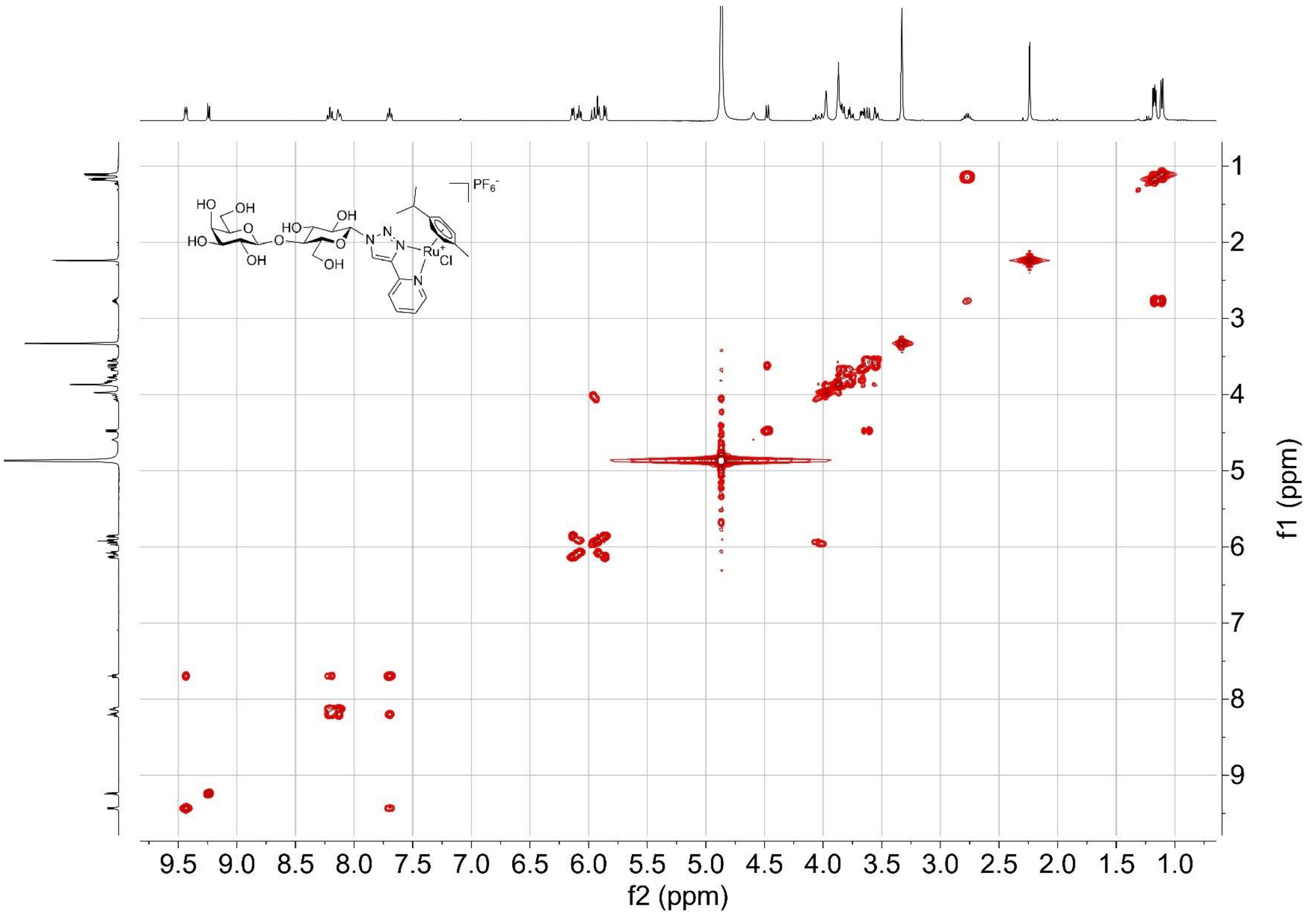

^{1}H-^{13}C HSQC 11-PF

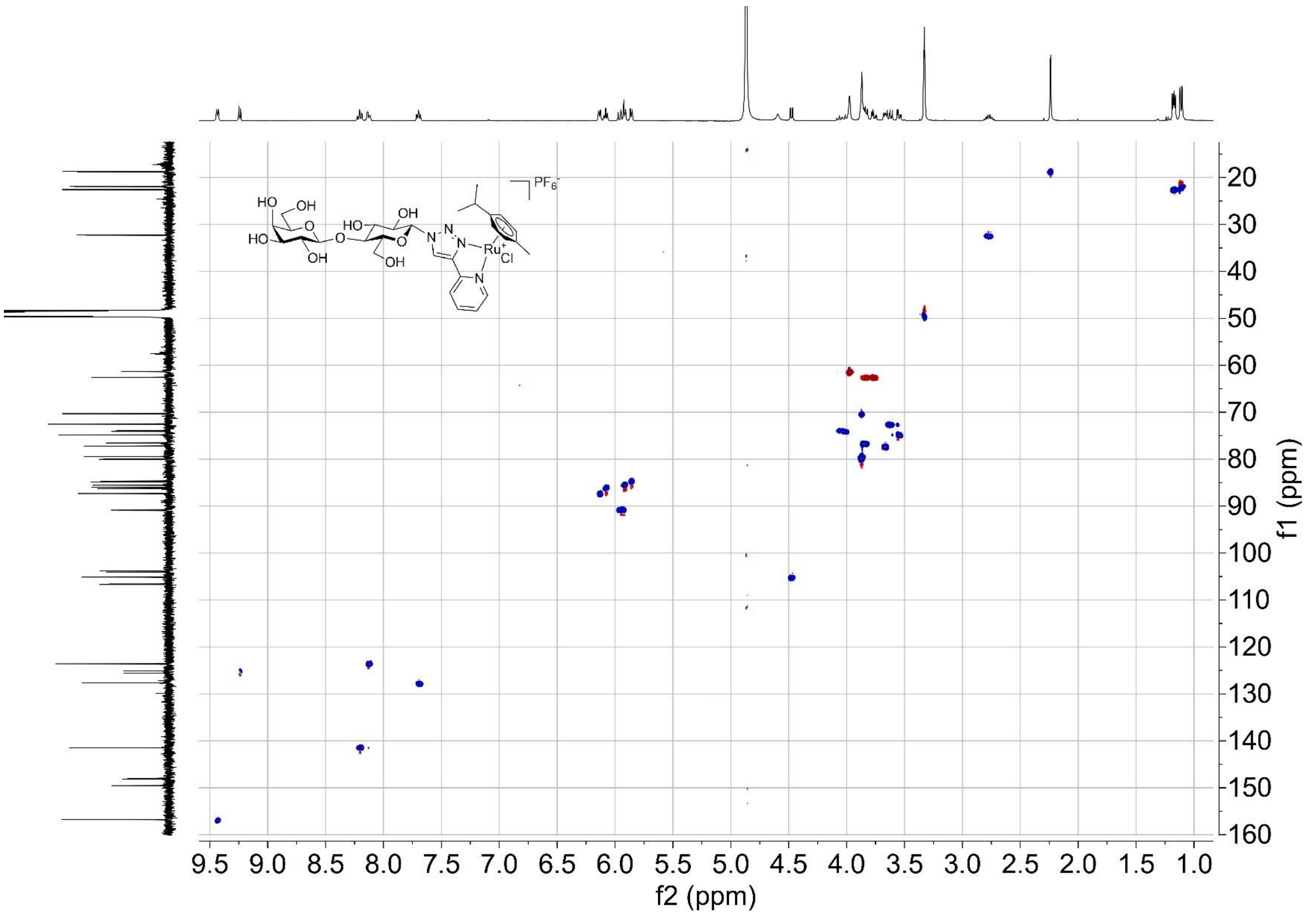

^{1}H-^{13}C HMBC 11-PF

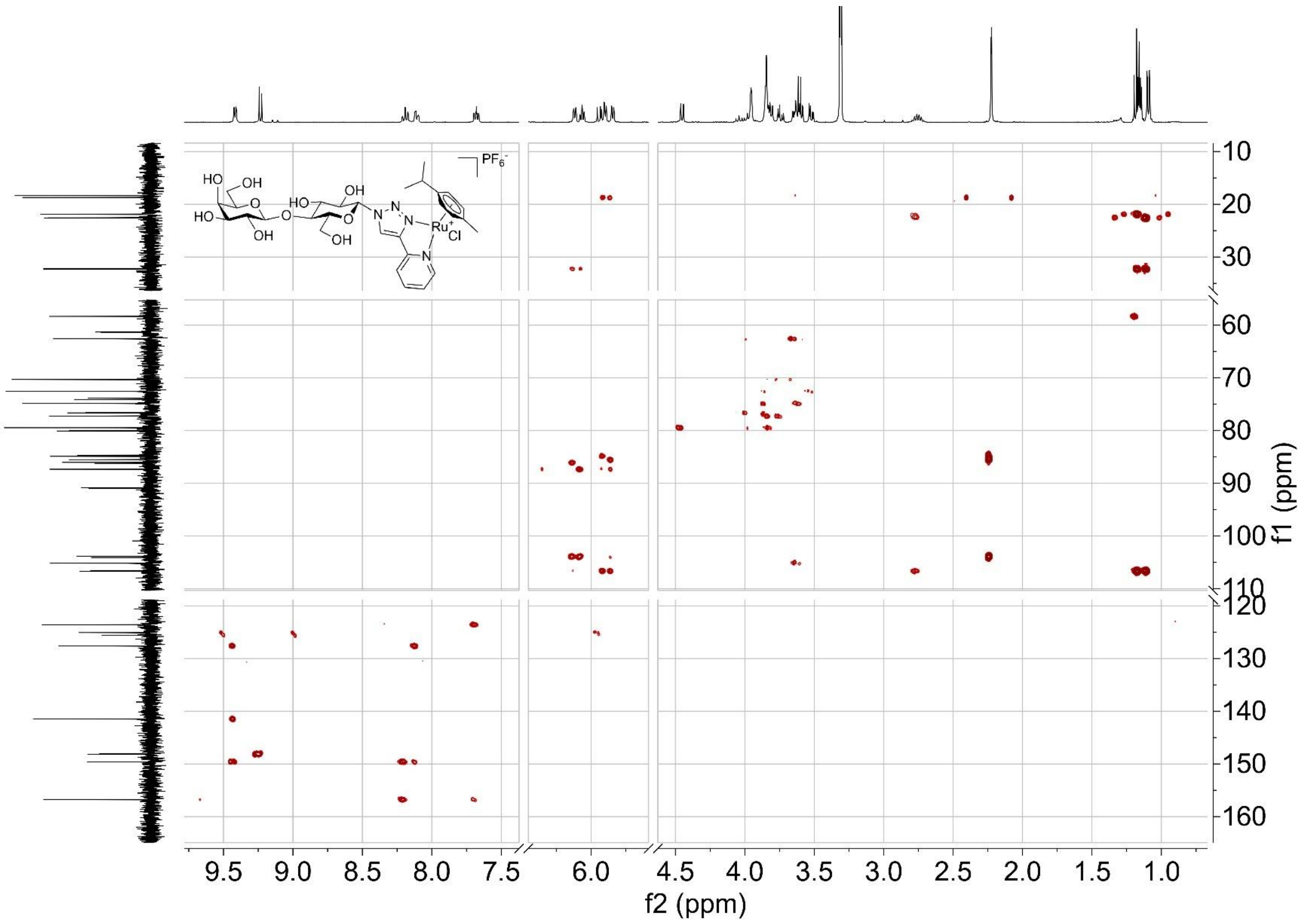

^{1}H-^{13}C HSQCTOCSY 11-PF

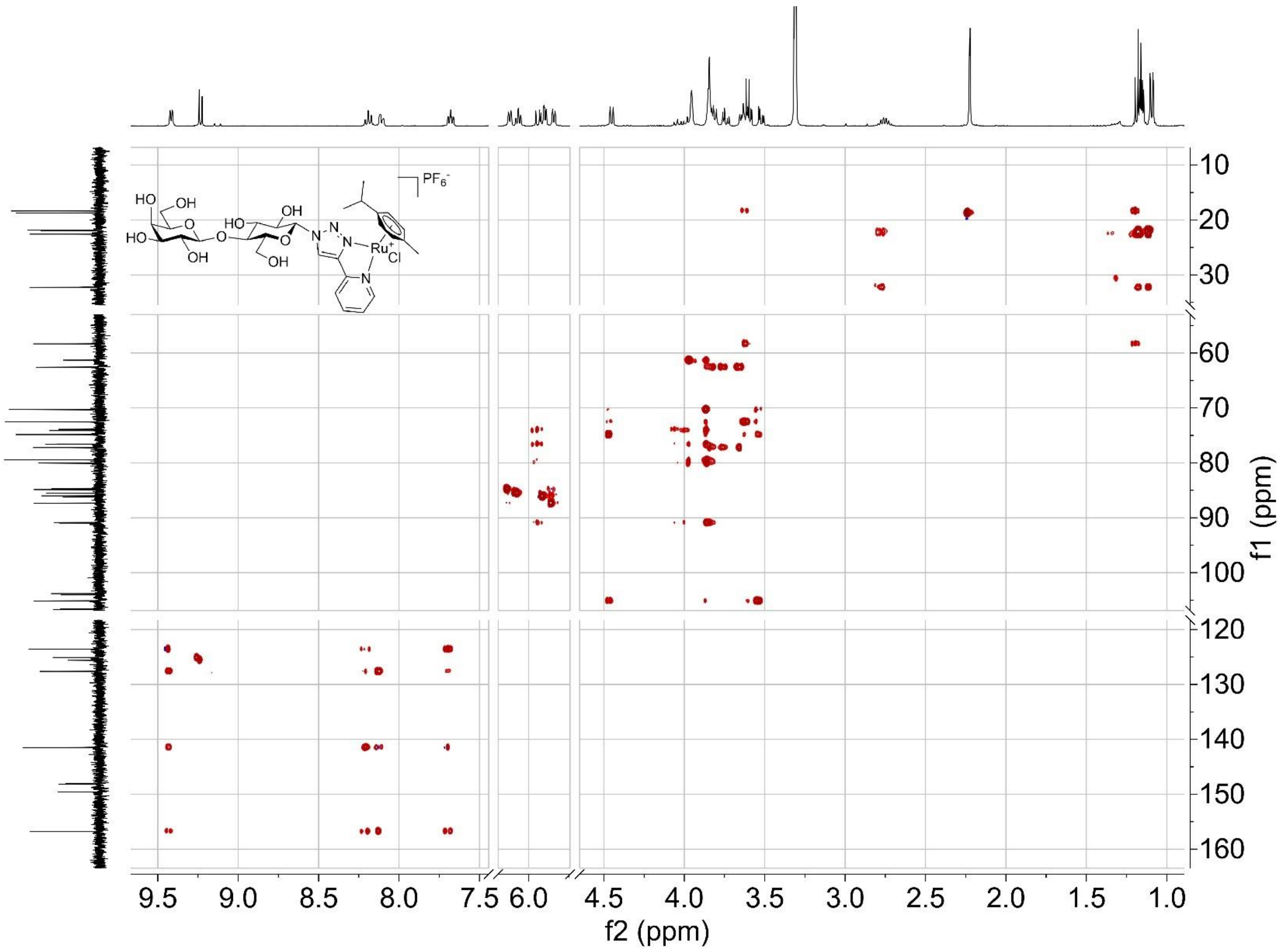

NMR COMPOUND 12-Cl

^{1}H NMR (400 MHz, CD$_3$OD) 12-Cl

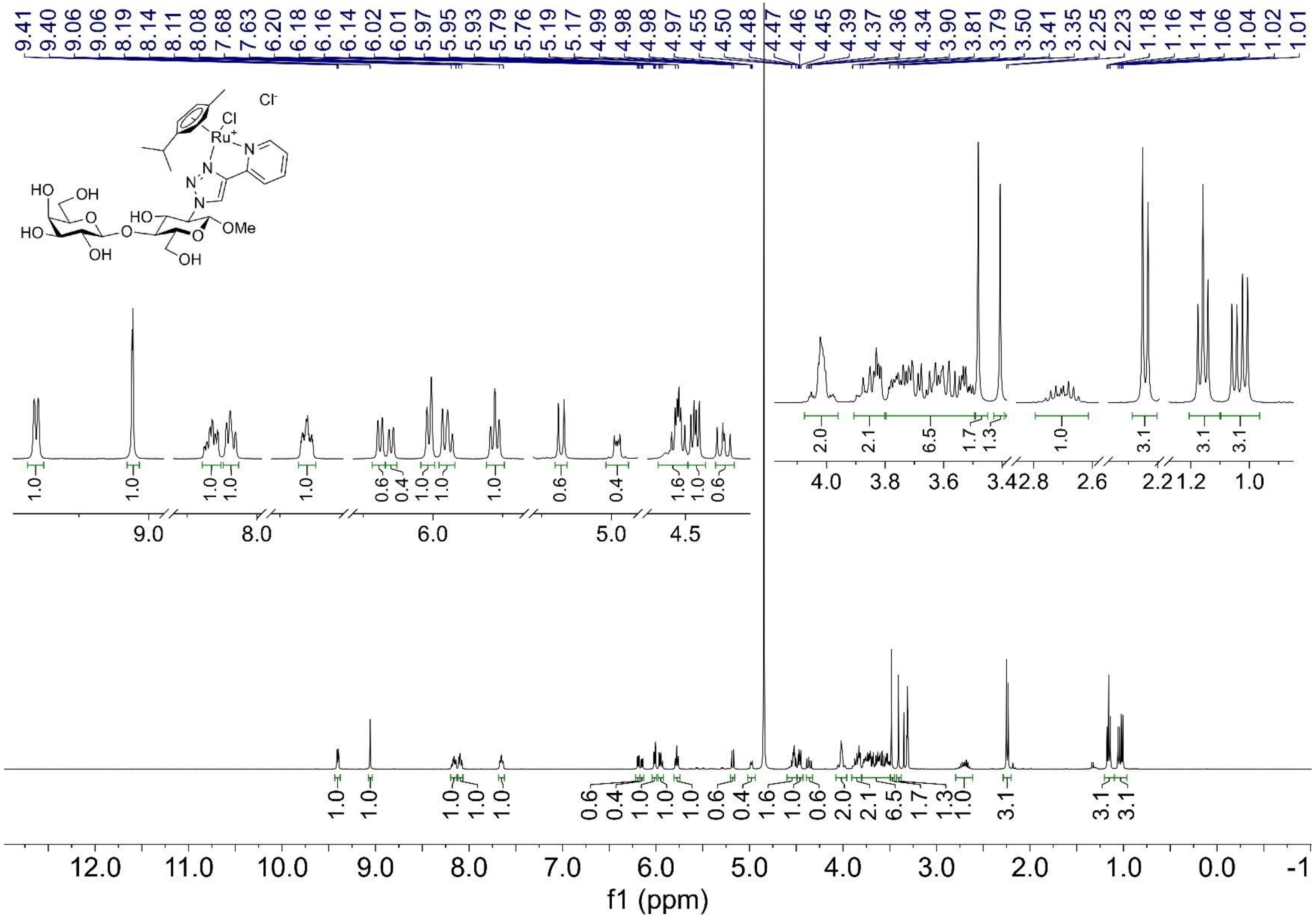

^{13}C NMR (101 MHz, CD_3OD) 12-Cl

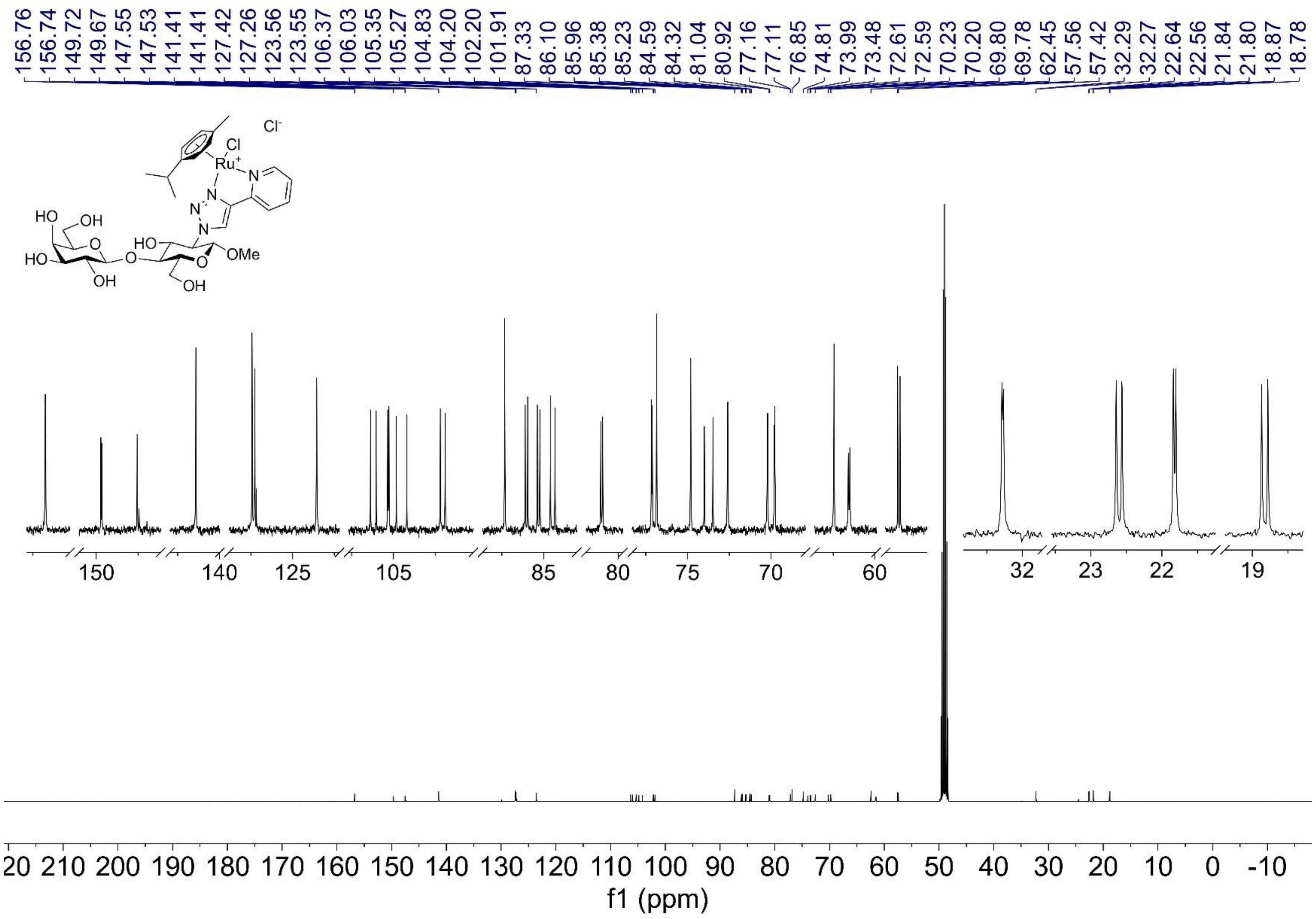

^{1}H-^{1}H COSY 12-Cl

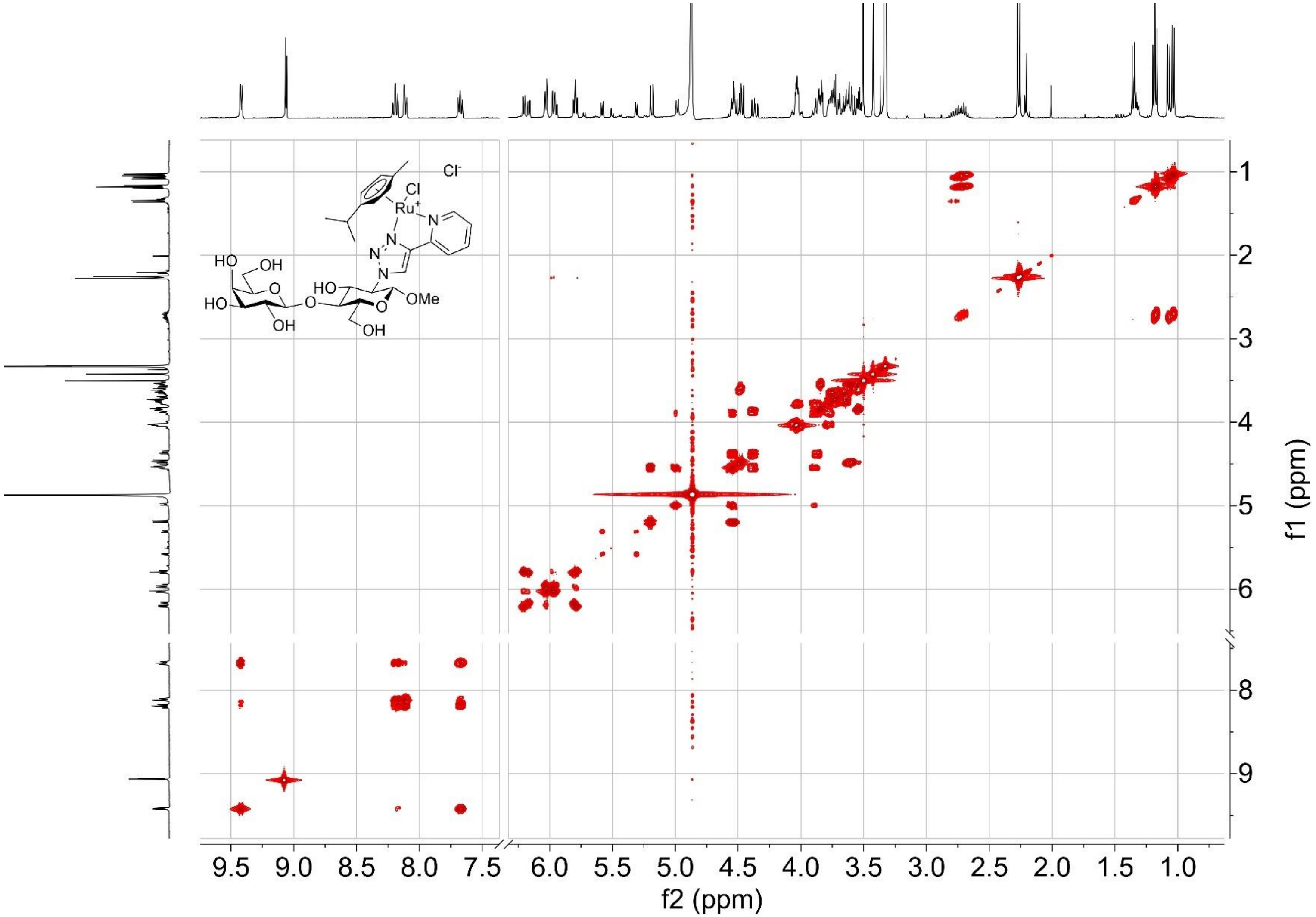

¹H-¹³C HSQC 12-Cl

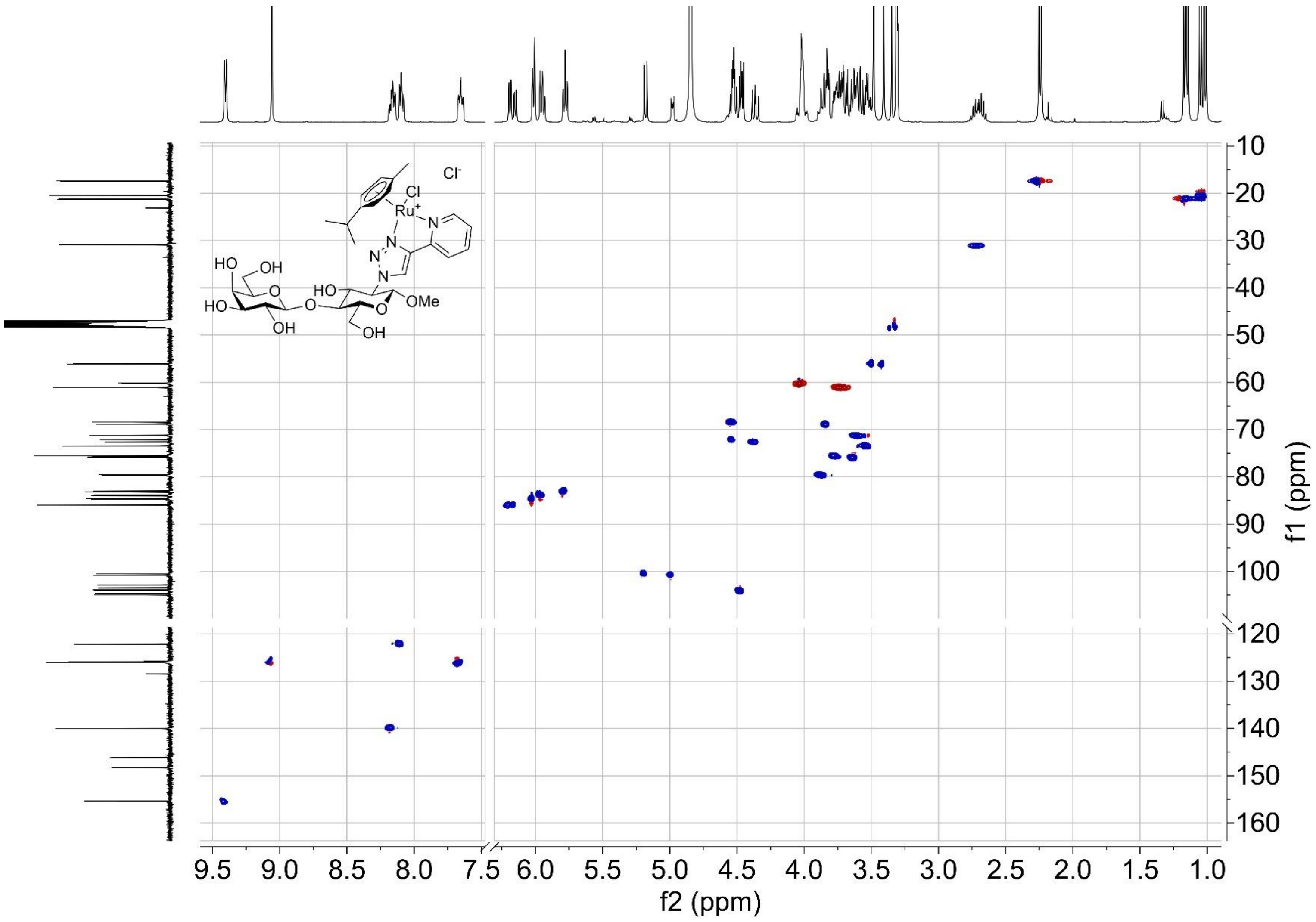

^{1}H-^{13}C HMBC 12-Cl

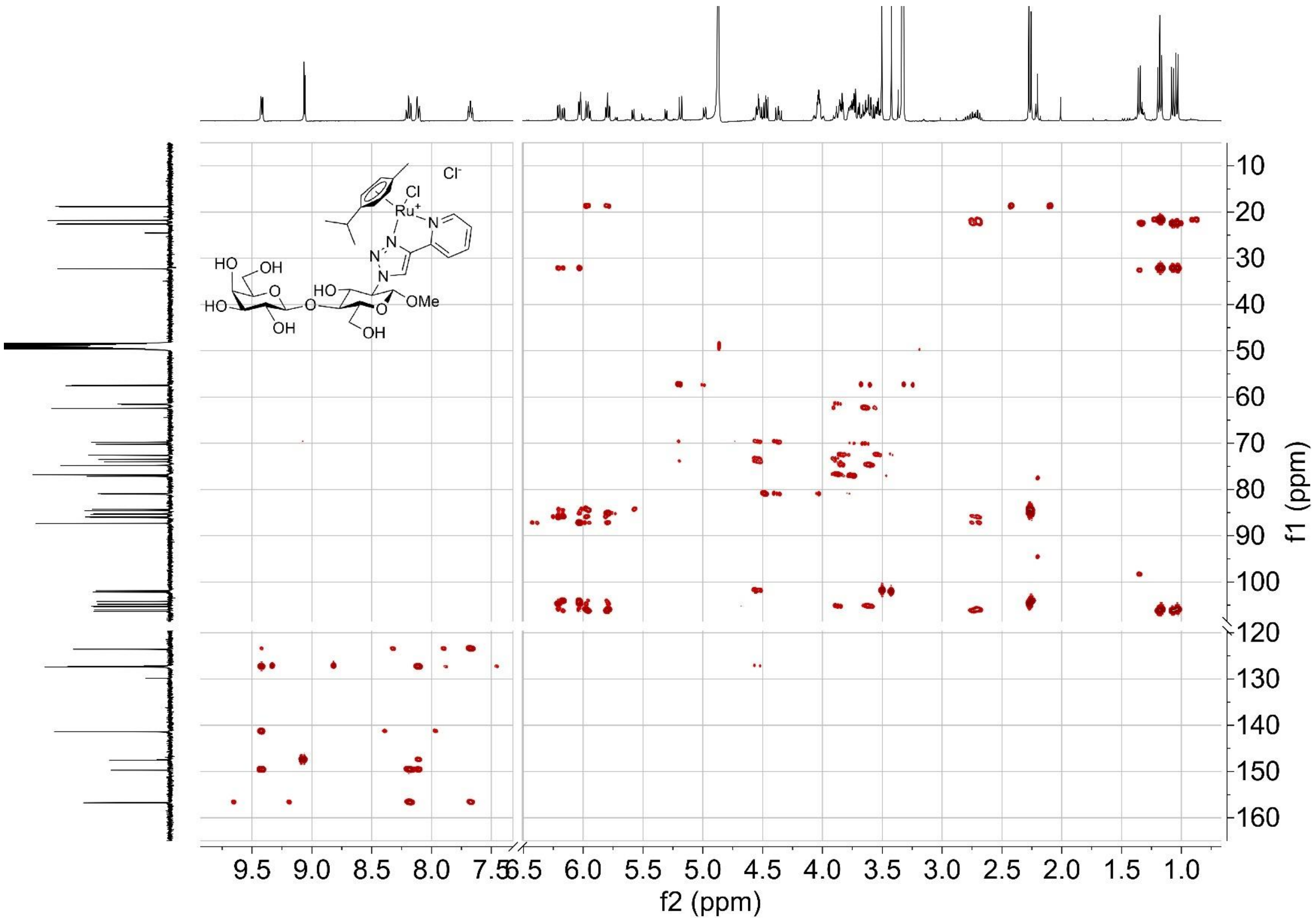

^{1}H-^{13}C HSQCTOCSY 12-Cl

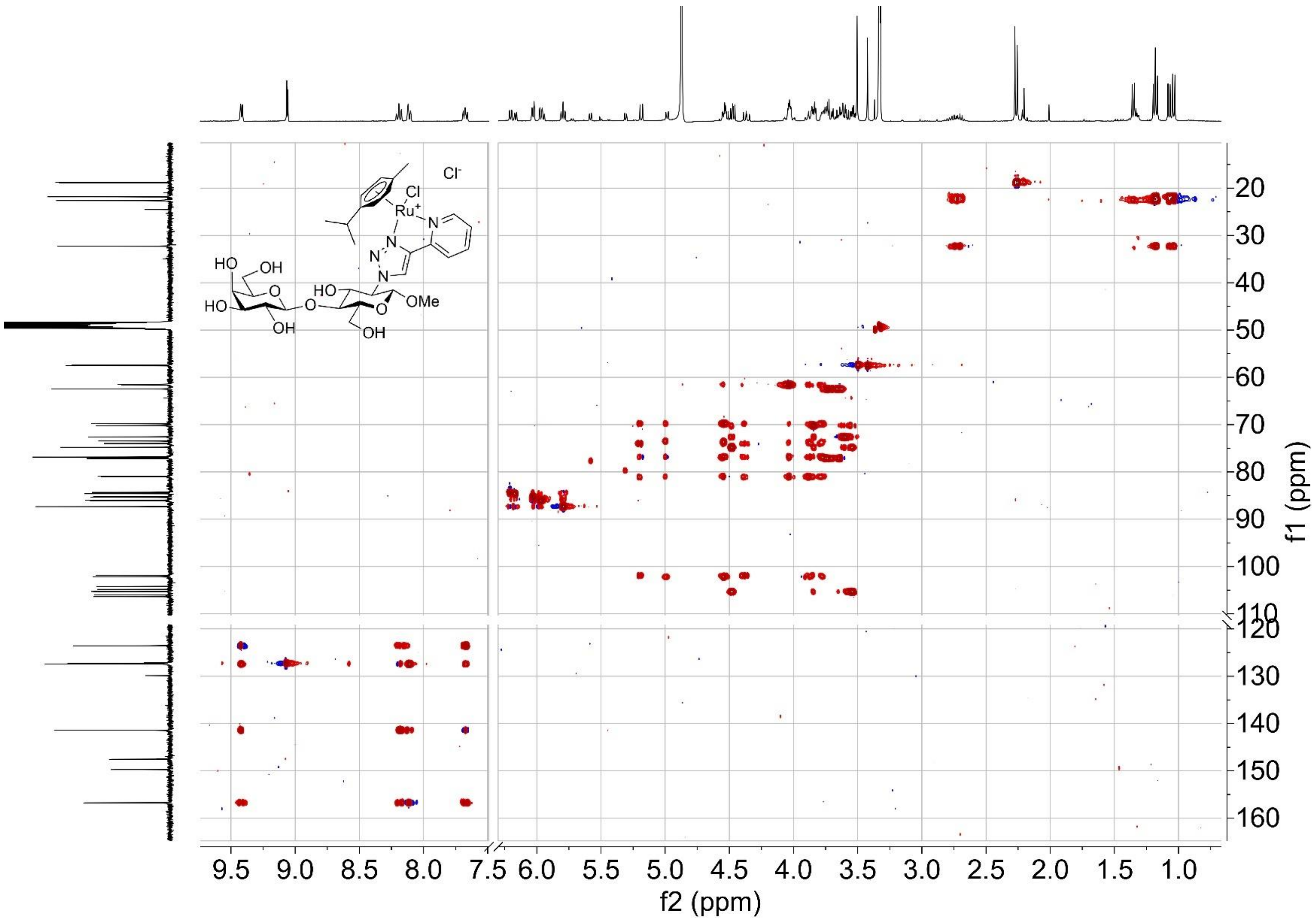

NMR COMPOUND 13-Cl

^{1}H NMR (400 MHz, CD_3OD) 13-Cl

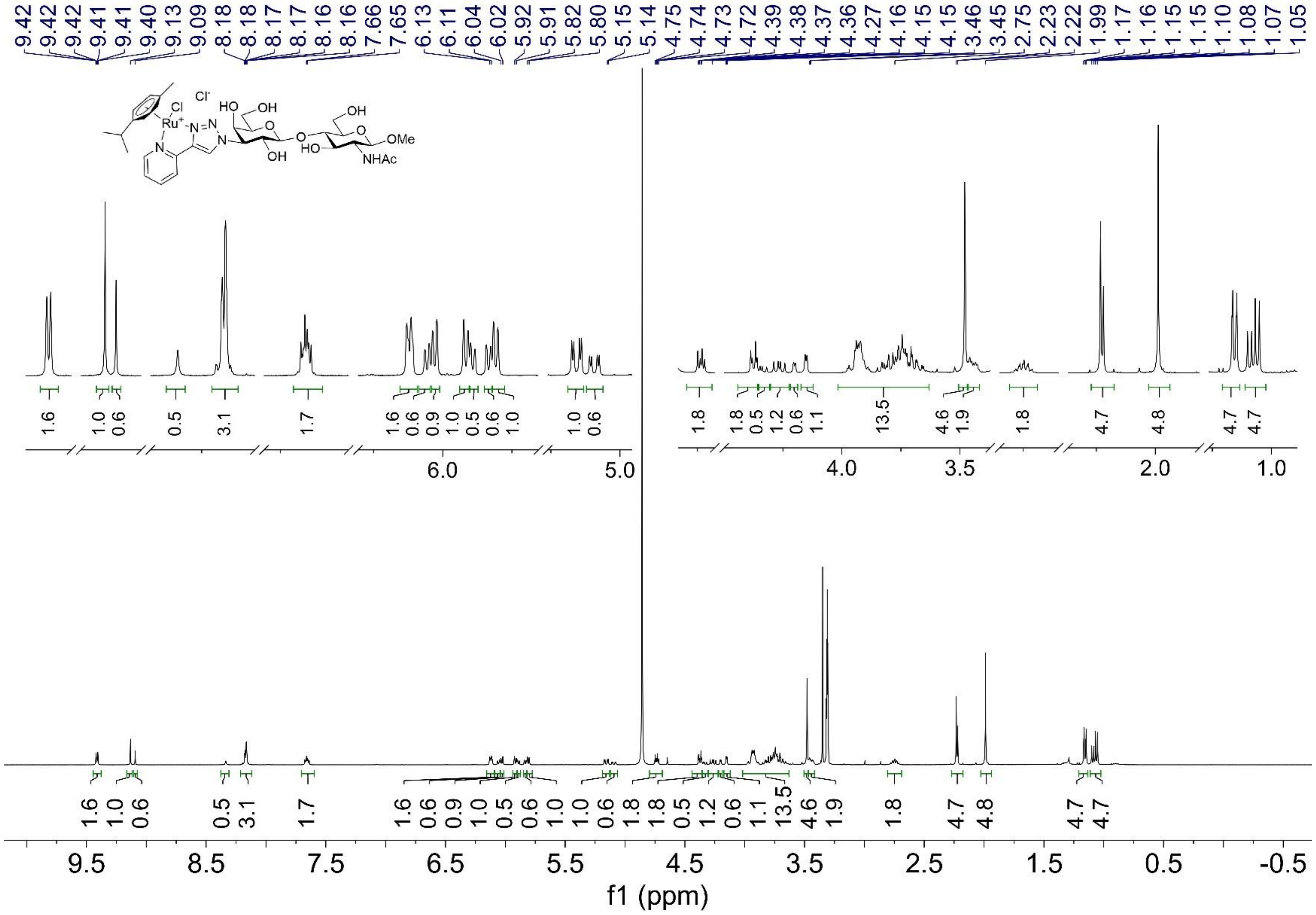

^{13}C NMR (101 MHz, CD_3OD) 13-Cl

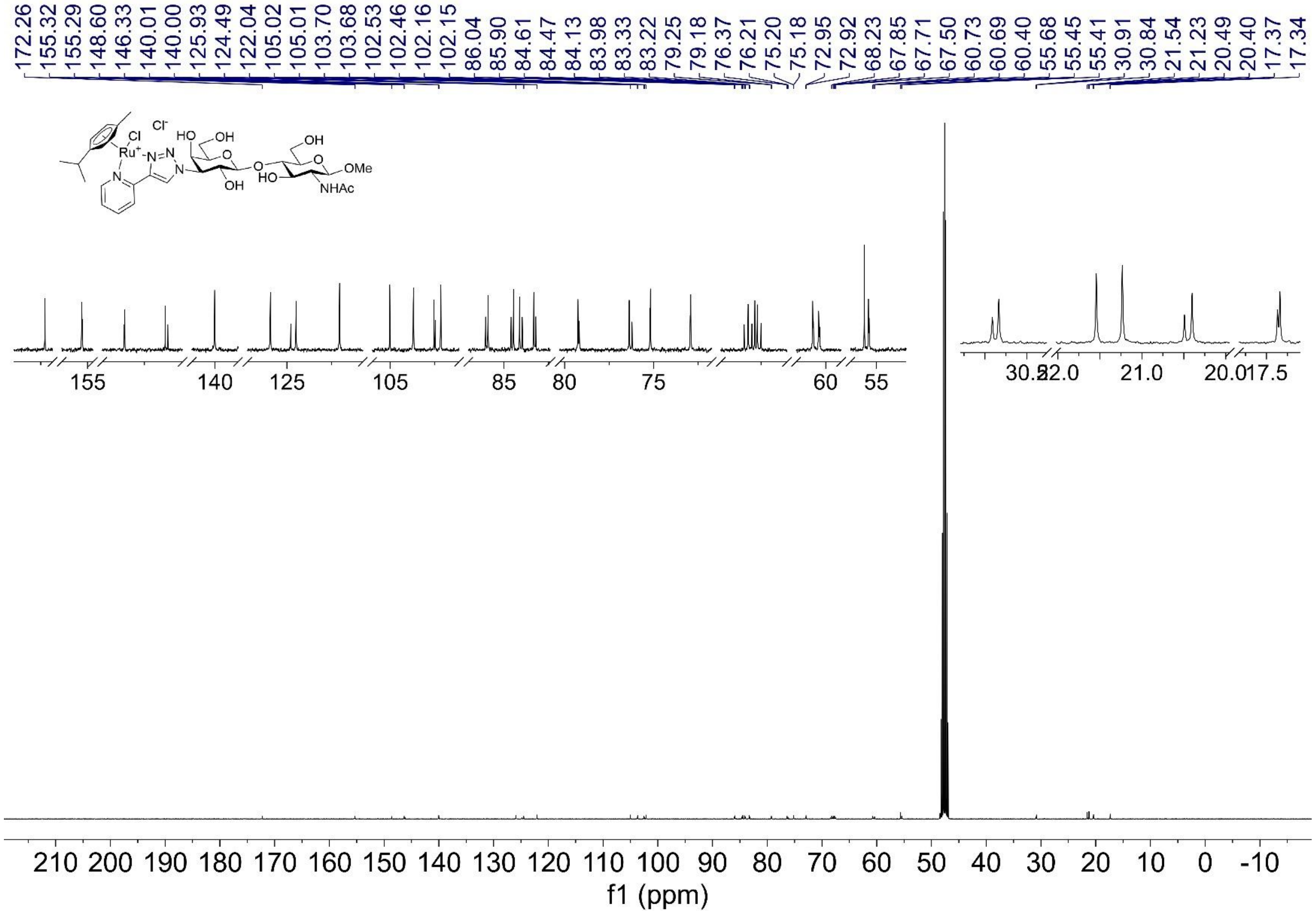

^{1}H-^{1}H COSY 13-Cl

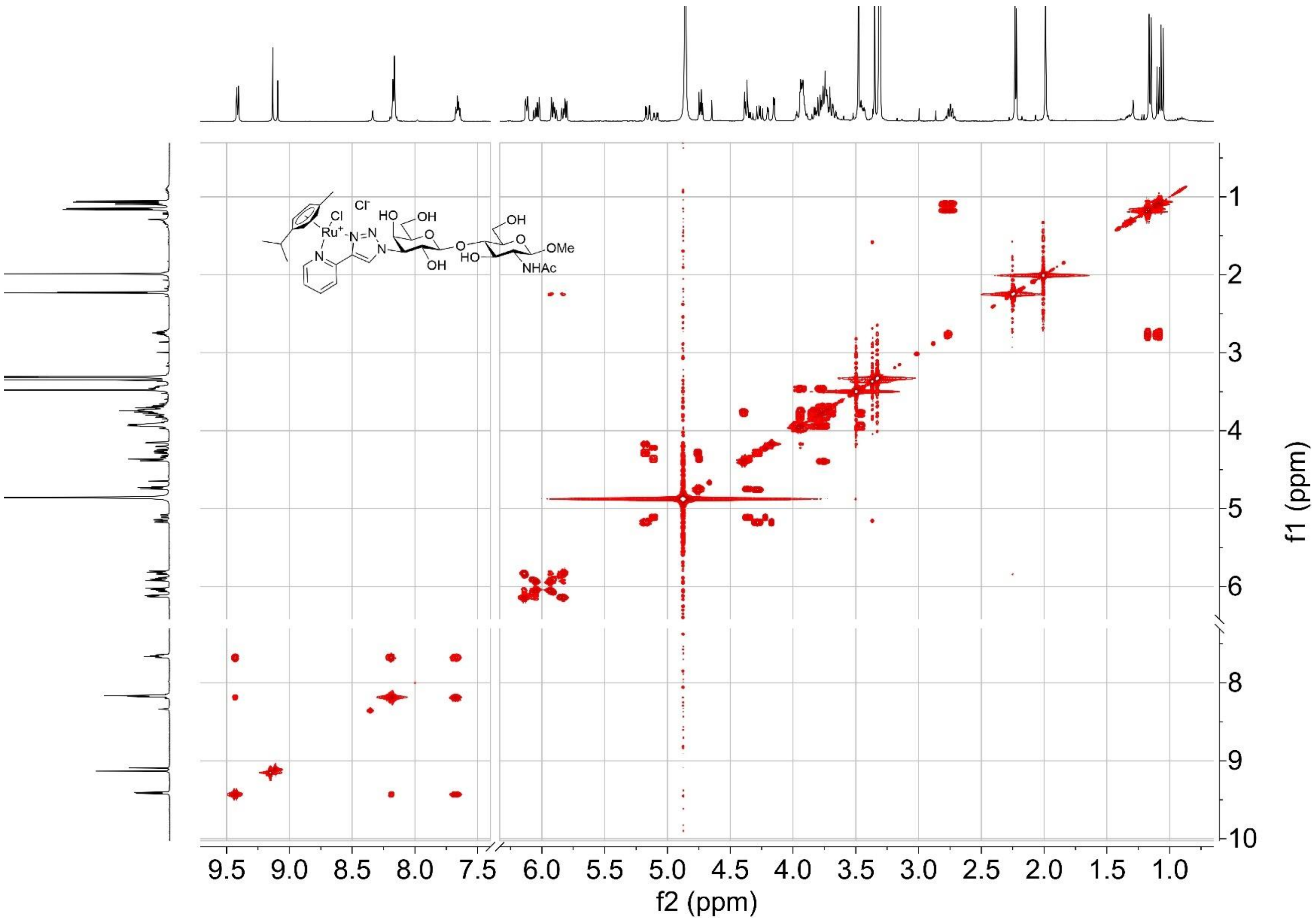

^{1}H-^{13}C HSQC 13-Cl

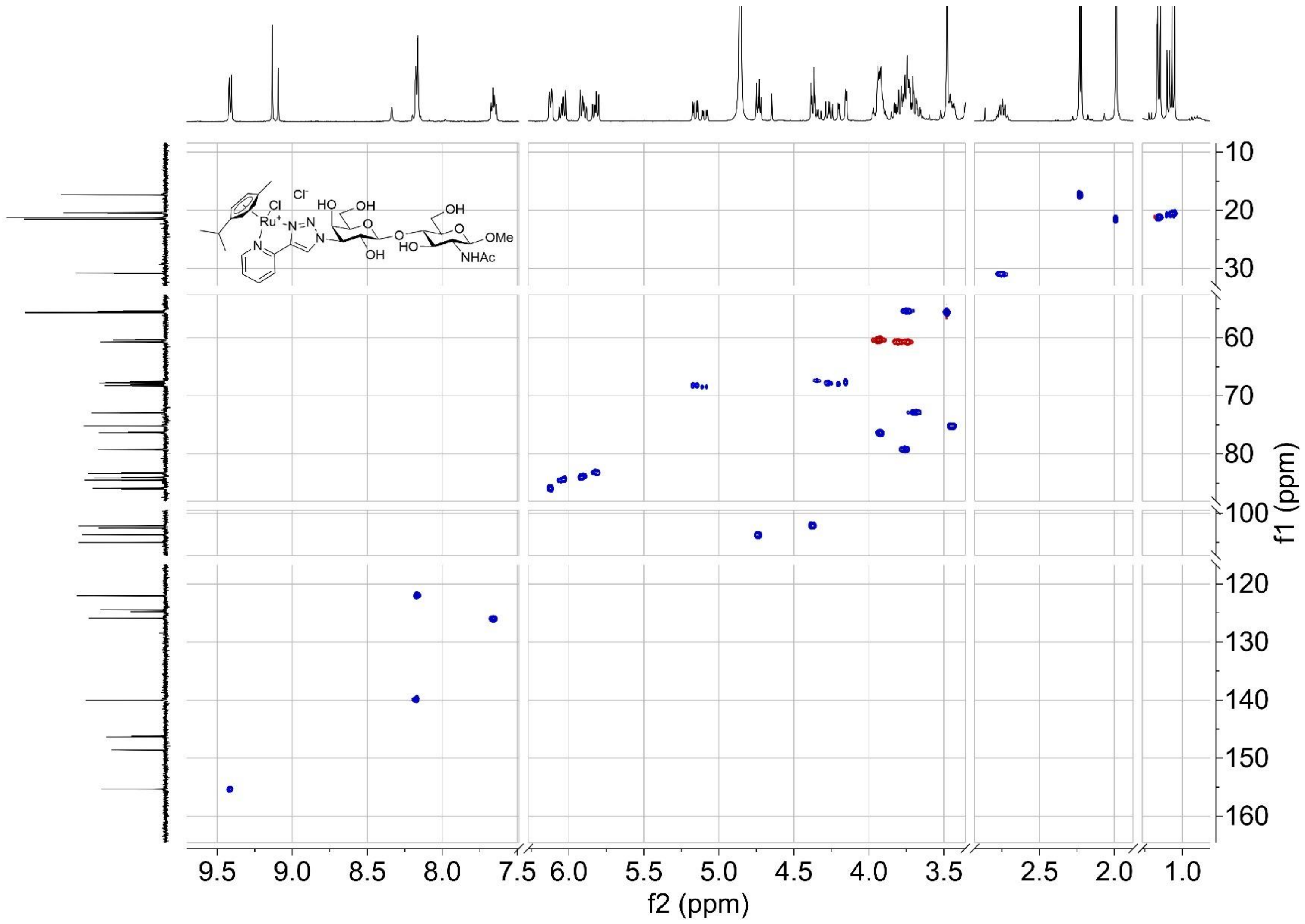

^{1}H-^{13}C HMBC 13-Cl

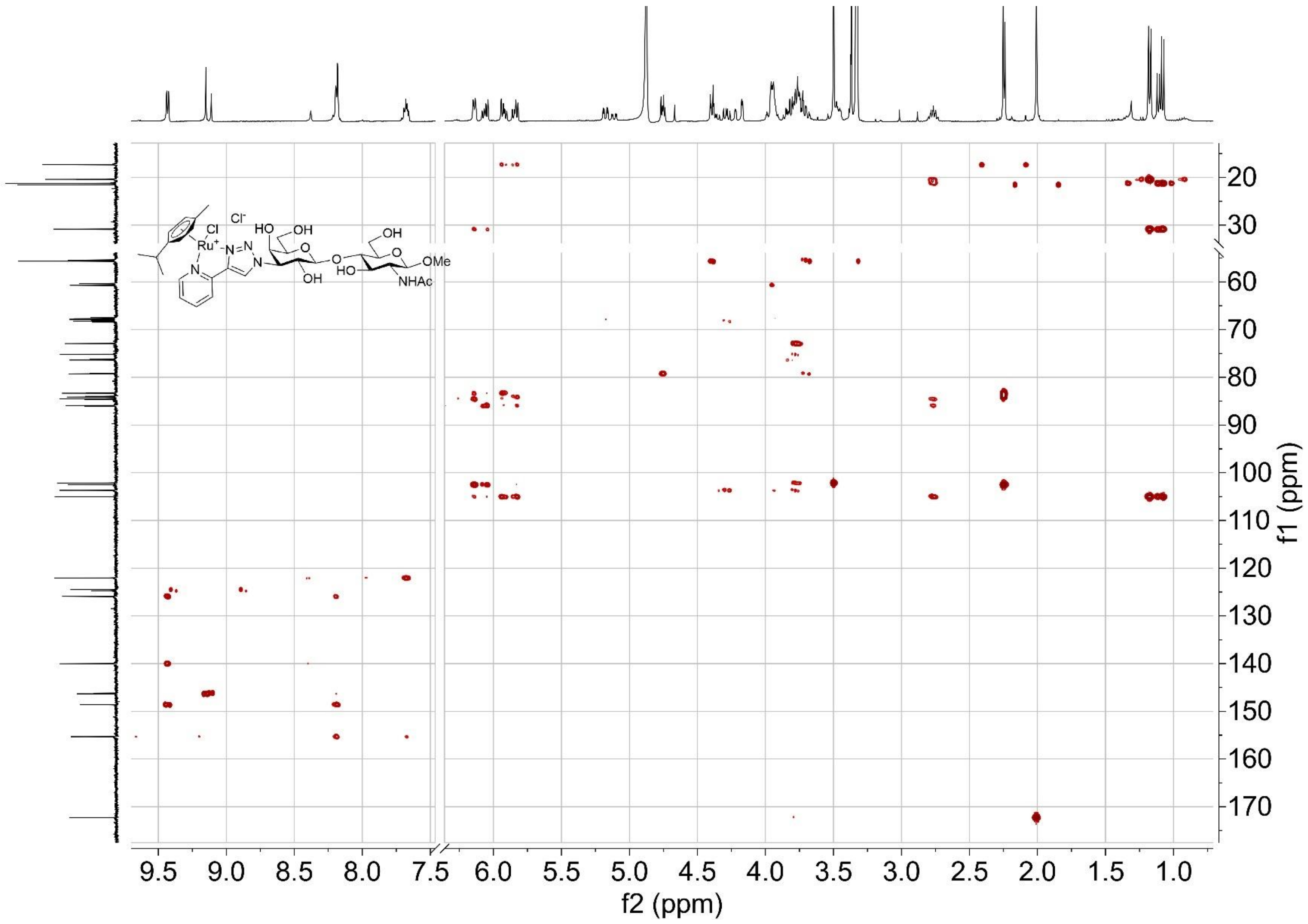

^1H-^{13}C HSQCTOCSY 13-Cl

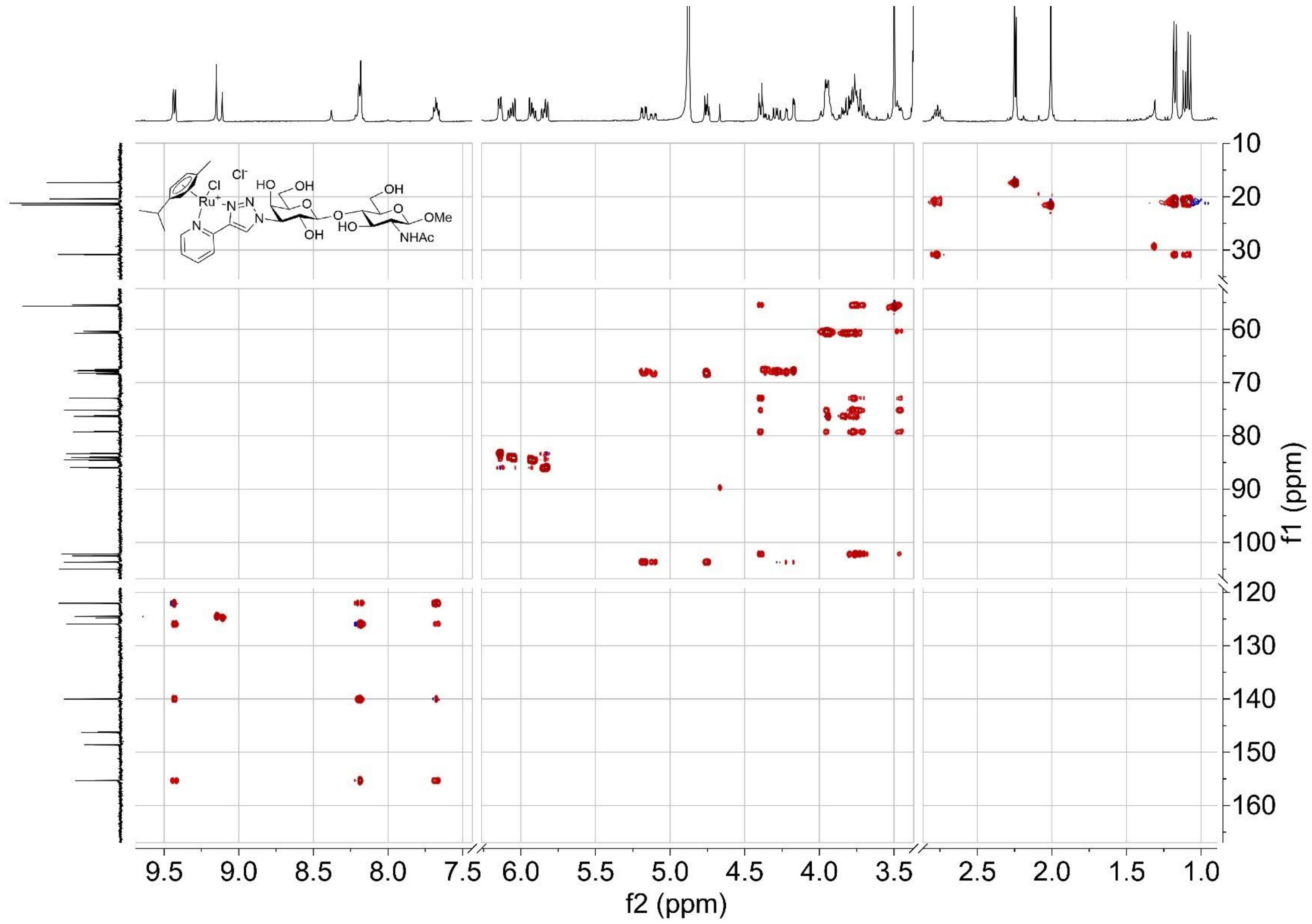

NMR COMPOUND 13-PF

^{1}H NMR (400 MHz, DMSO-d_6) 13-PF

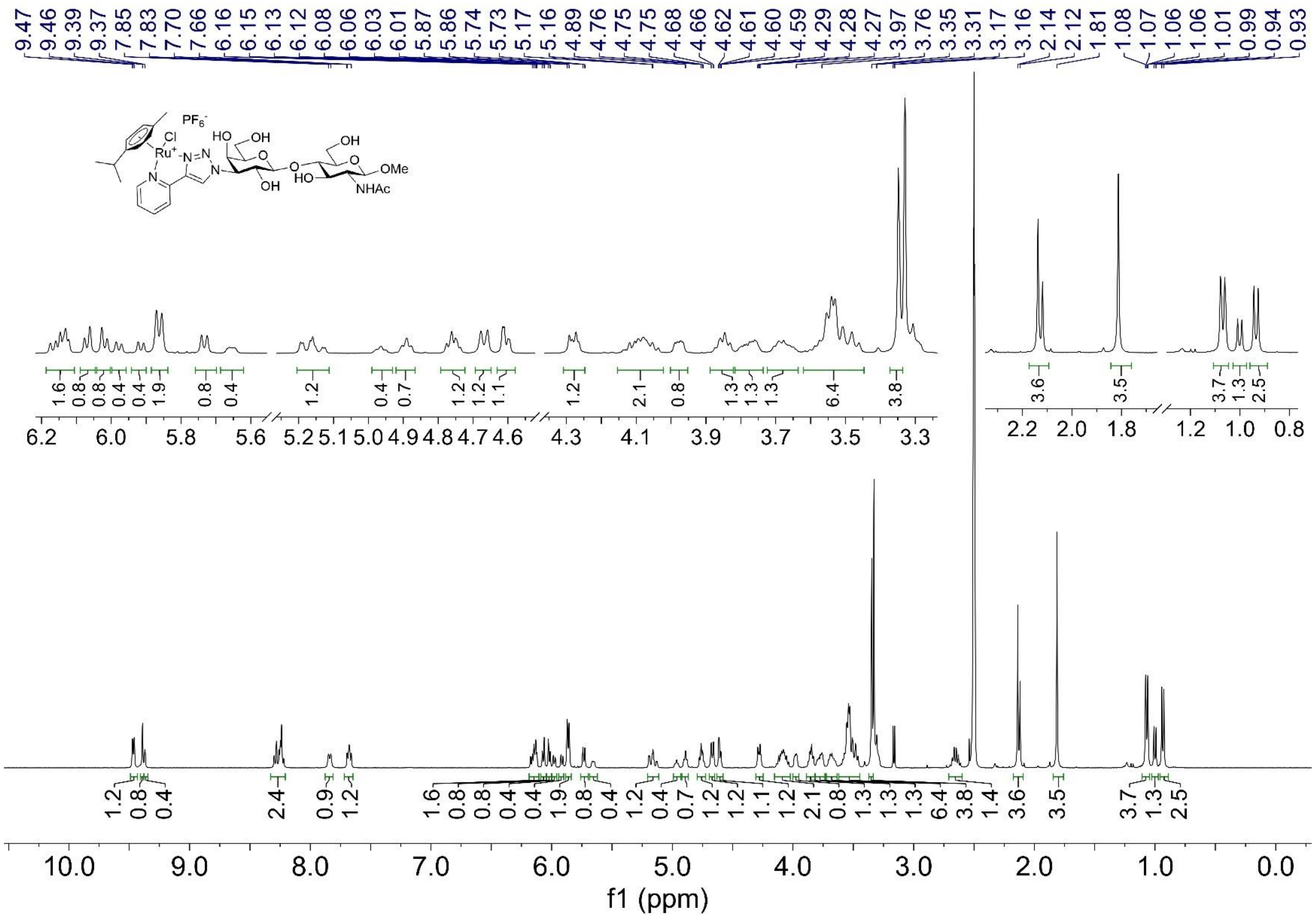

^{13}C NMR (101 MHz, DMSO-d_6) 13-PF

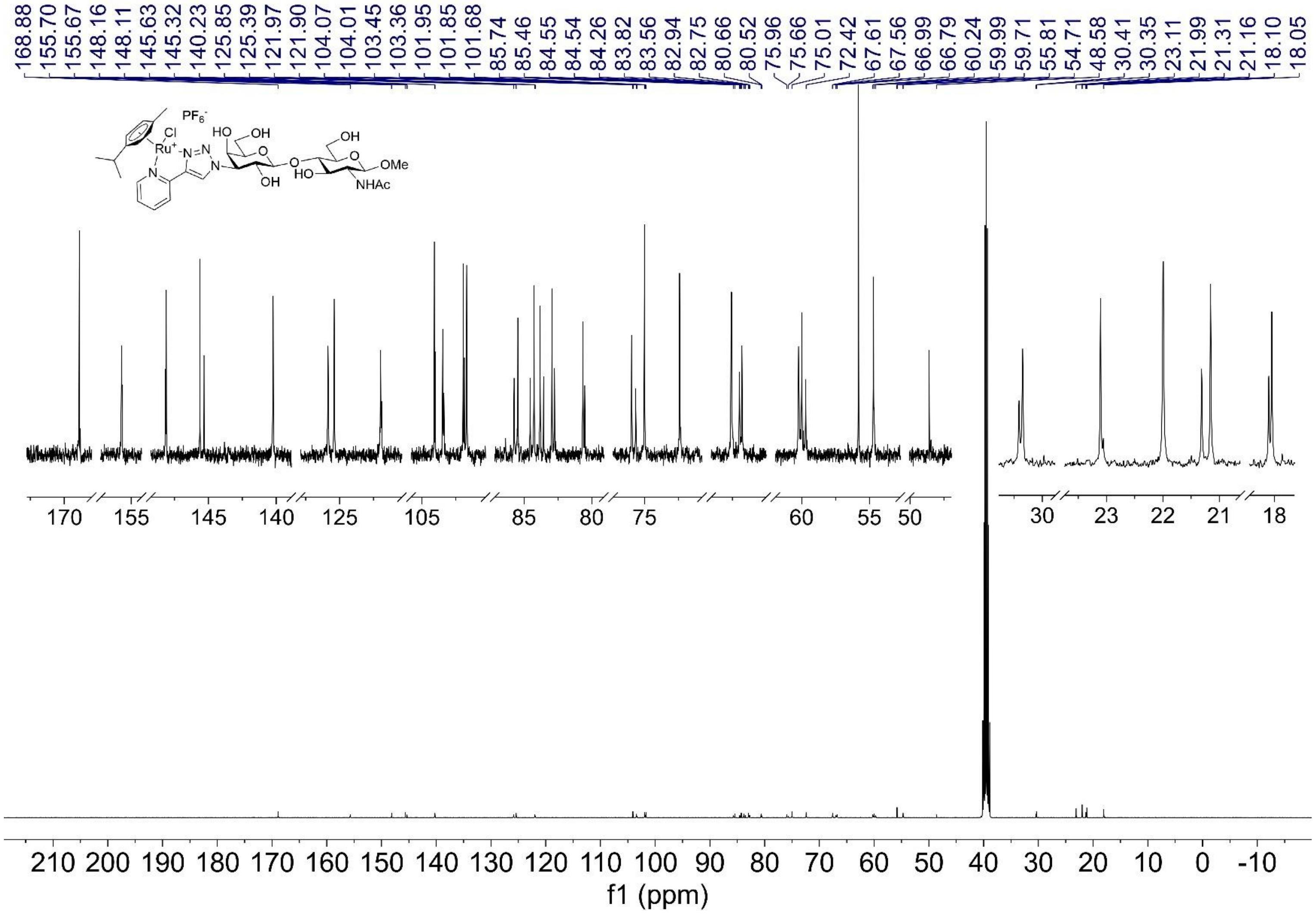

^{19}F NMR (376 MHz, DMSO-d_6) 13-PF

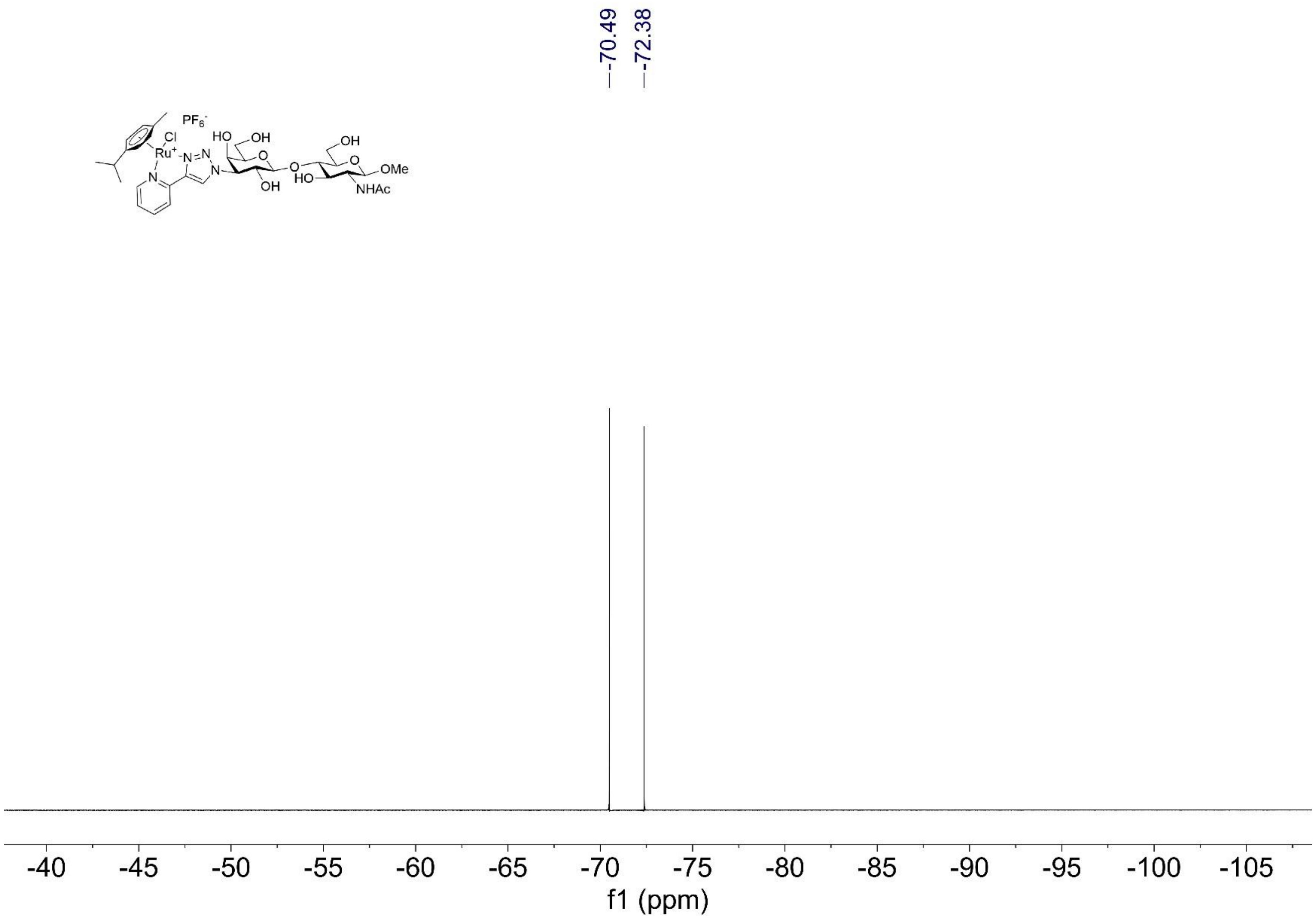

^{31}P NMR (162 MHz, DMSO-d_6) 13-PF

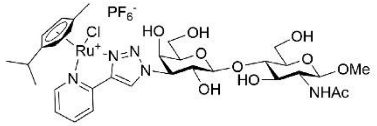

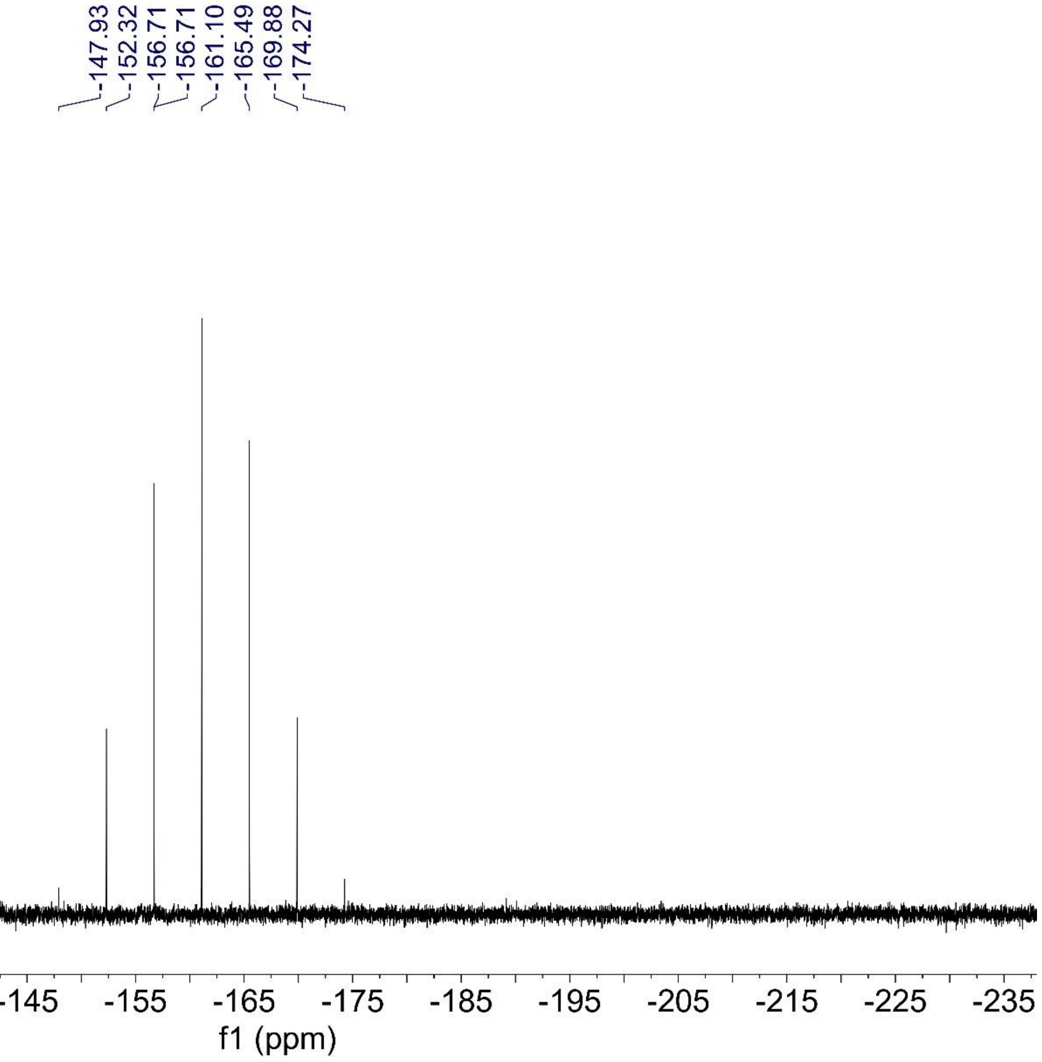

^{1}H-^{1}H COSY 13-PF

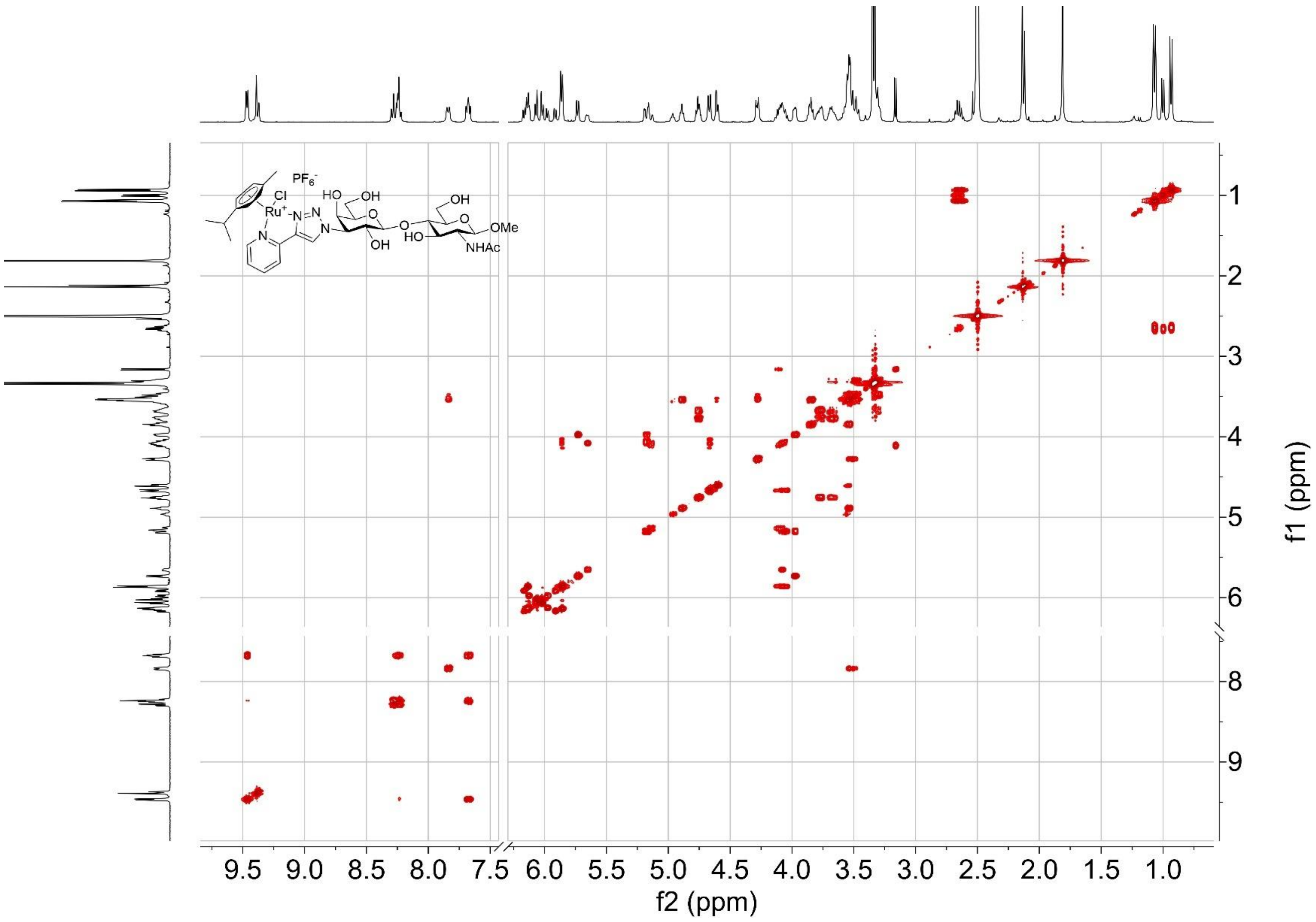

^{1}H-^{13}C HSQC 13-PF

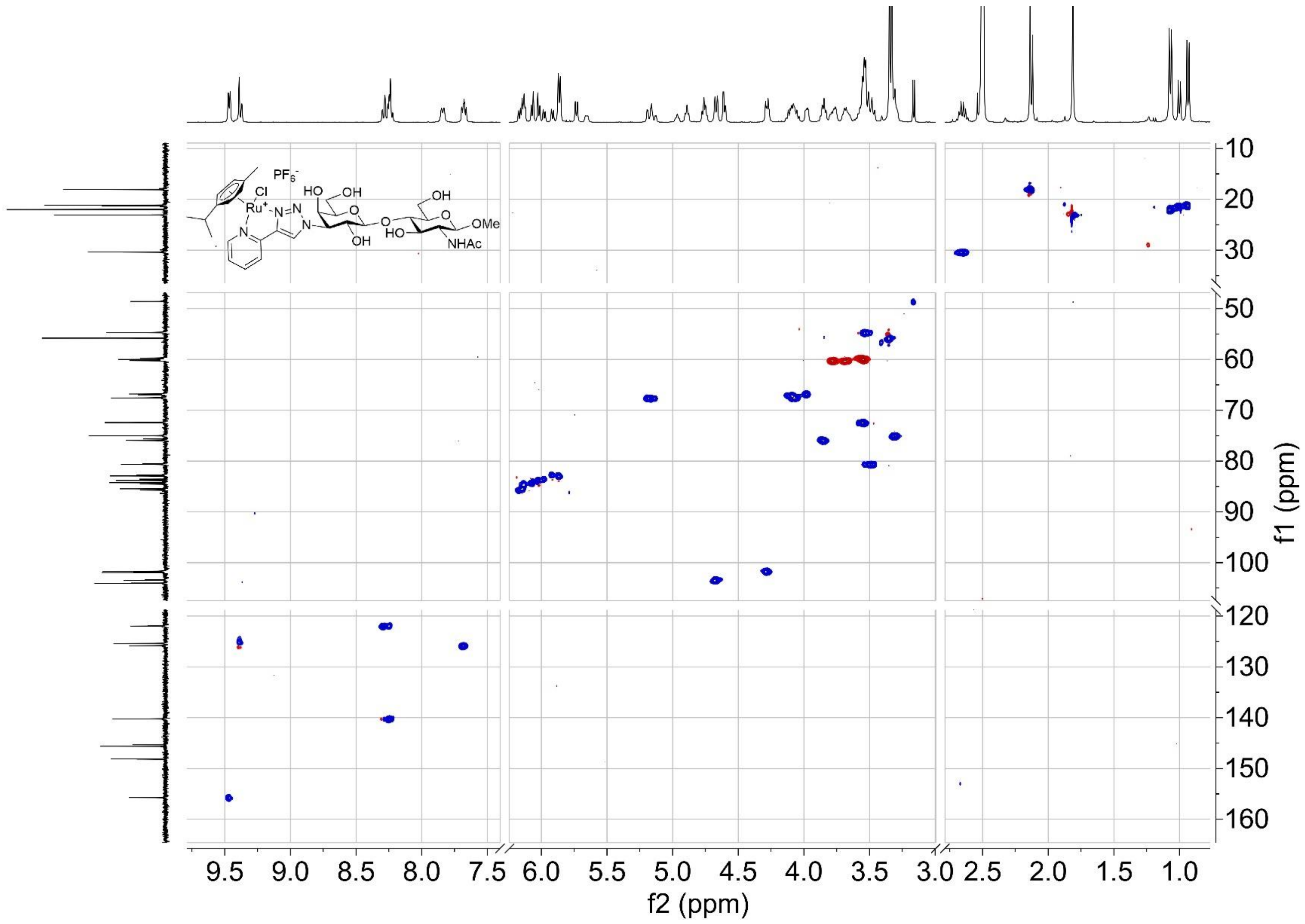

^{1}H-^{13}C HMBC 13-PF

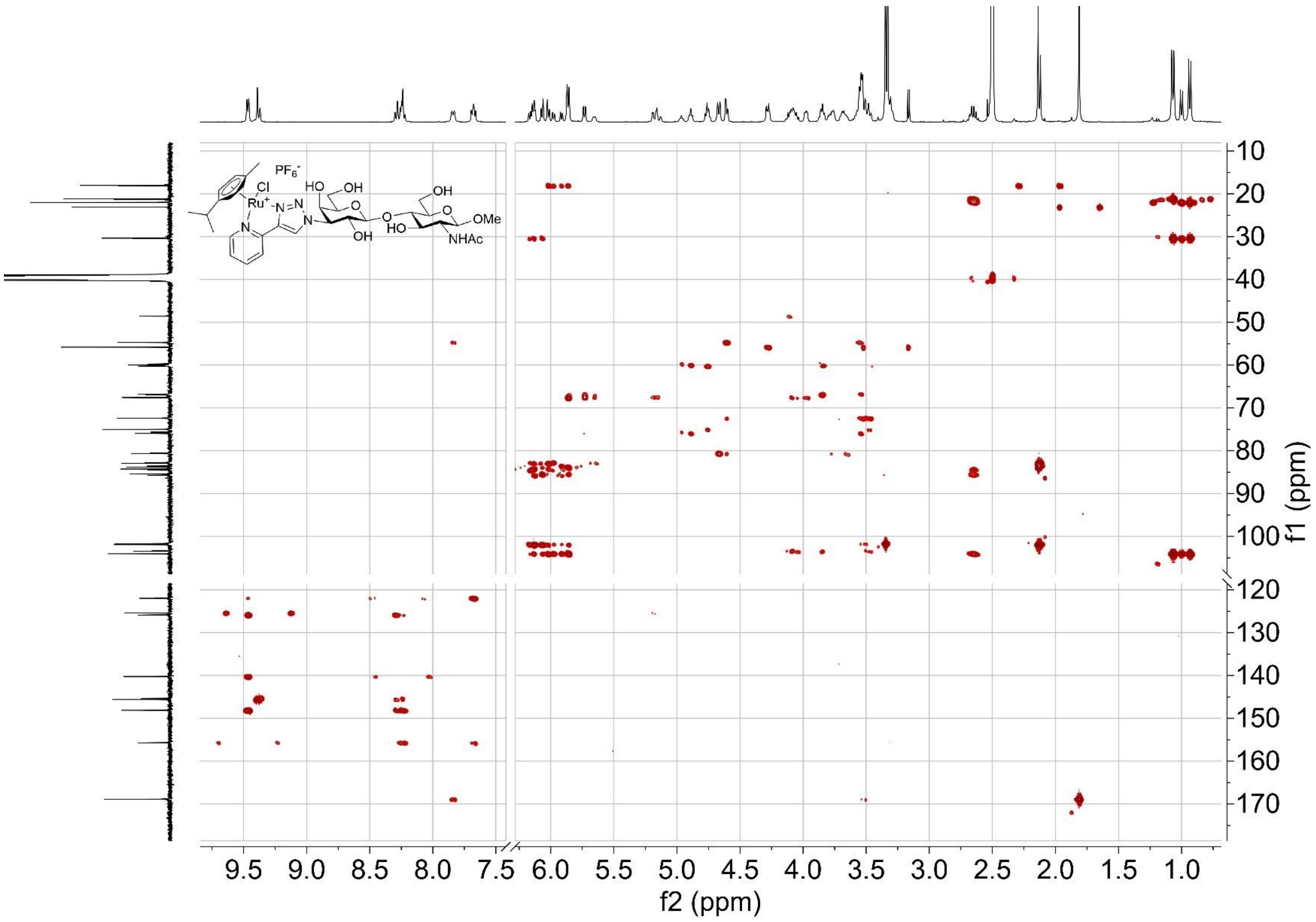

^{1}H-^{13}C HSQCTOCSY 13-PF

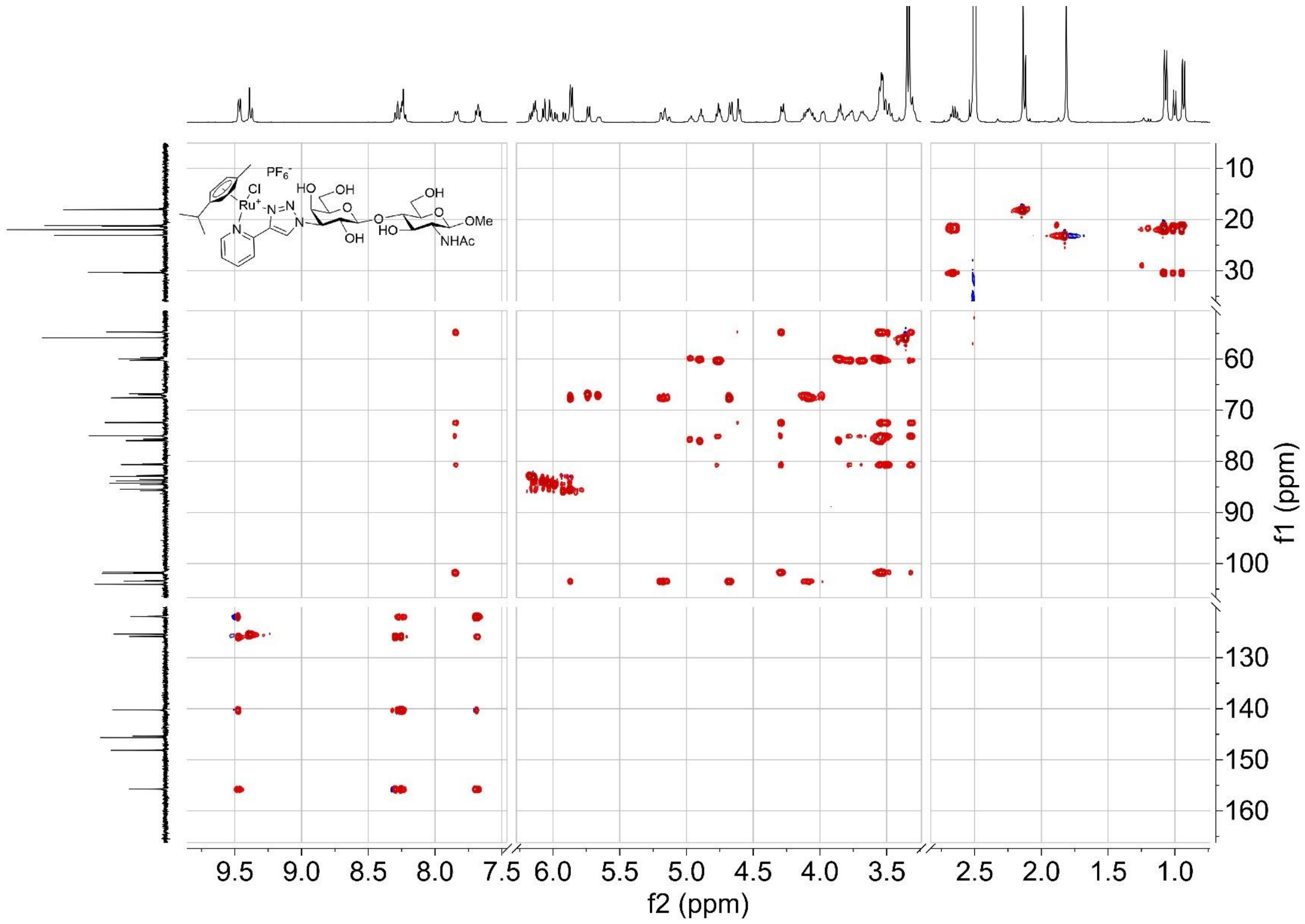

NMR COMPOUND 14-Cl

^{1}H NMR (400 MHz, CD_3OD) 14-Cl

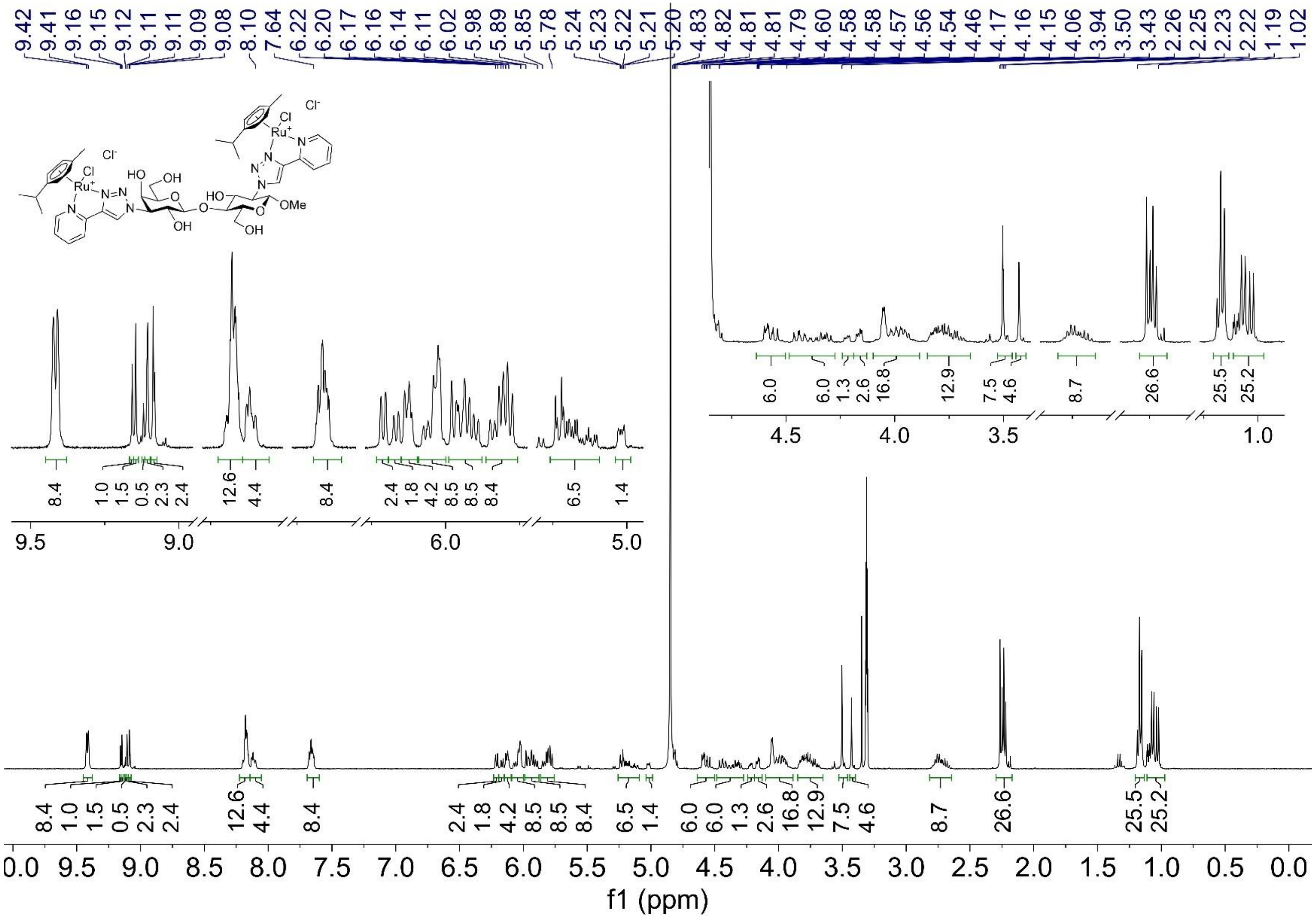

^{13}C NMR (101 MHz, CD_3OD) 14-Cl

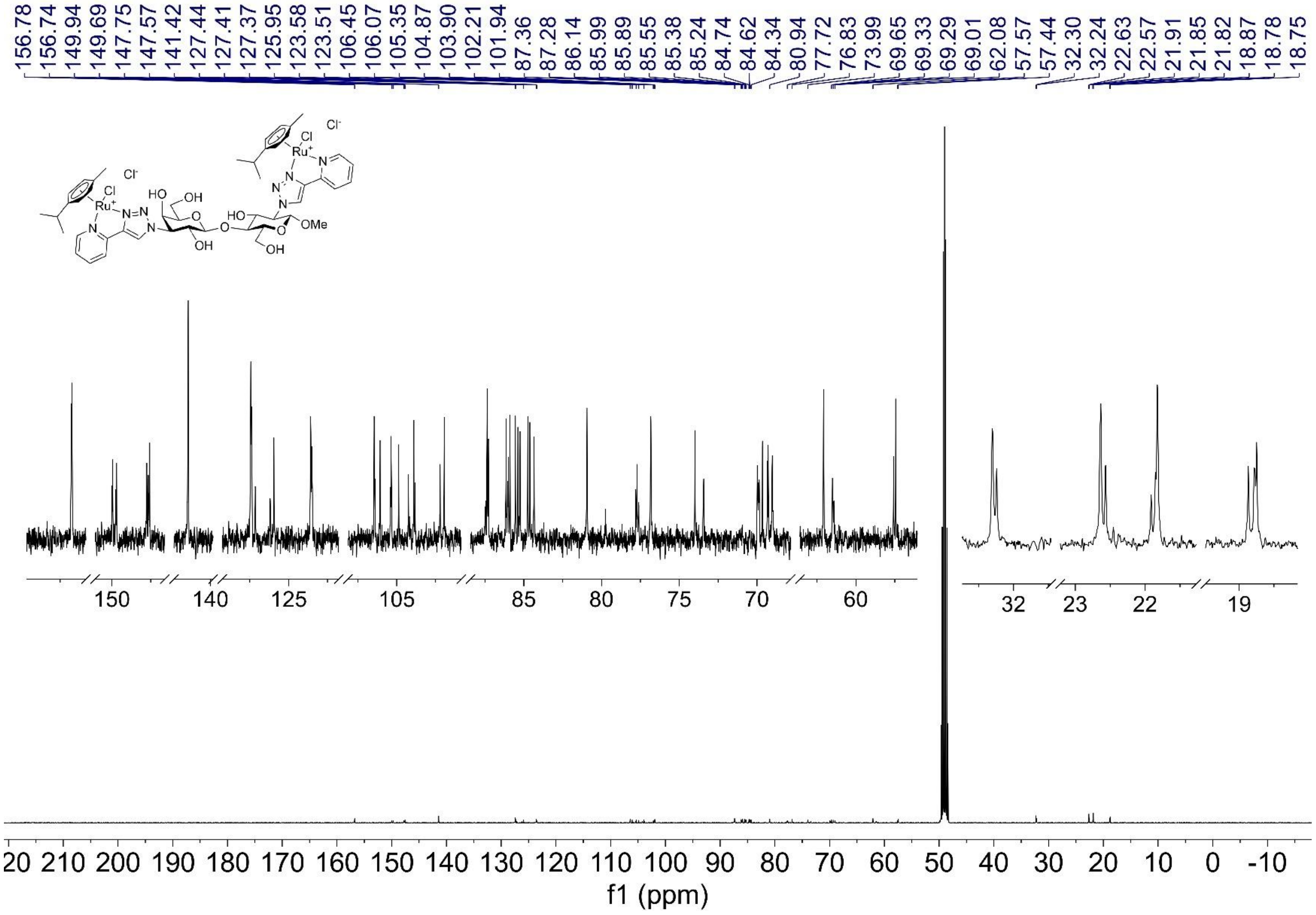

^{1}H-^{1}H COSY 14-Cl

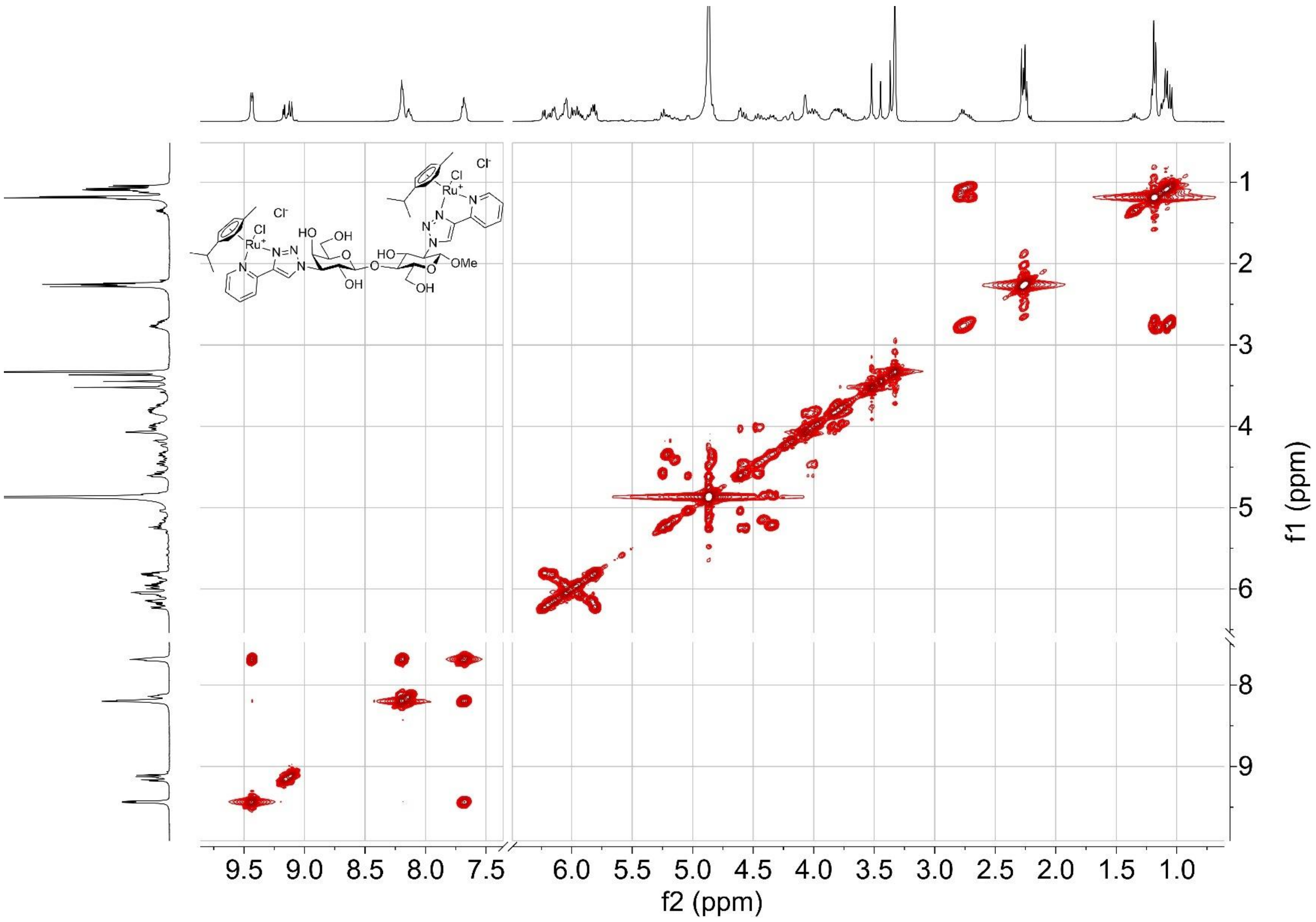

^{1}H-^{13}C HSQC 14-Cl

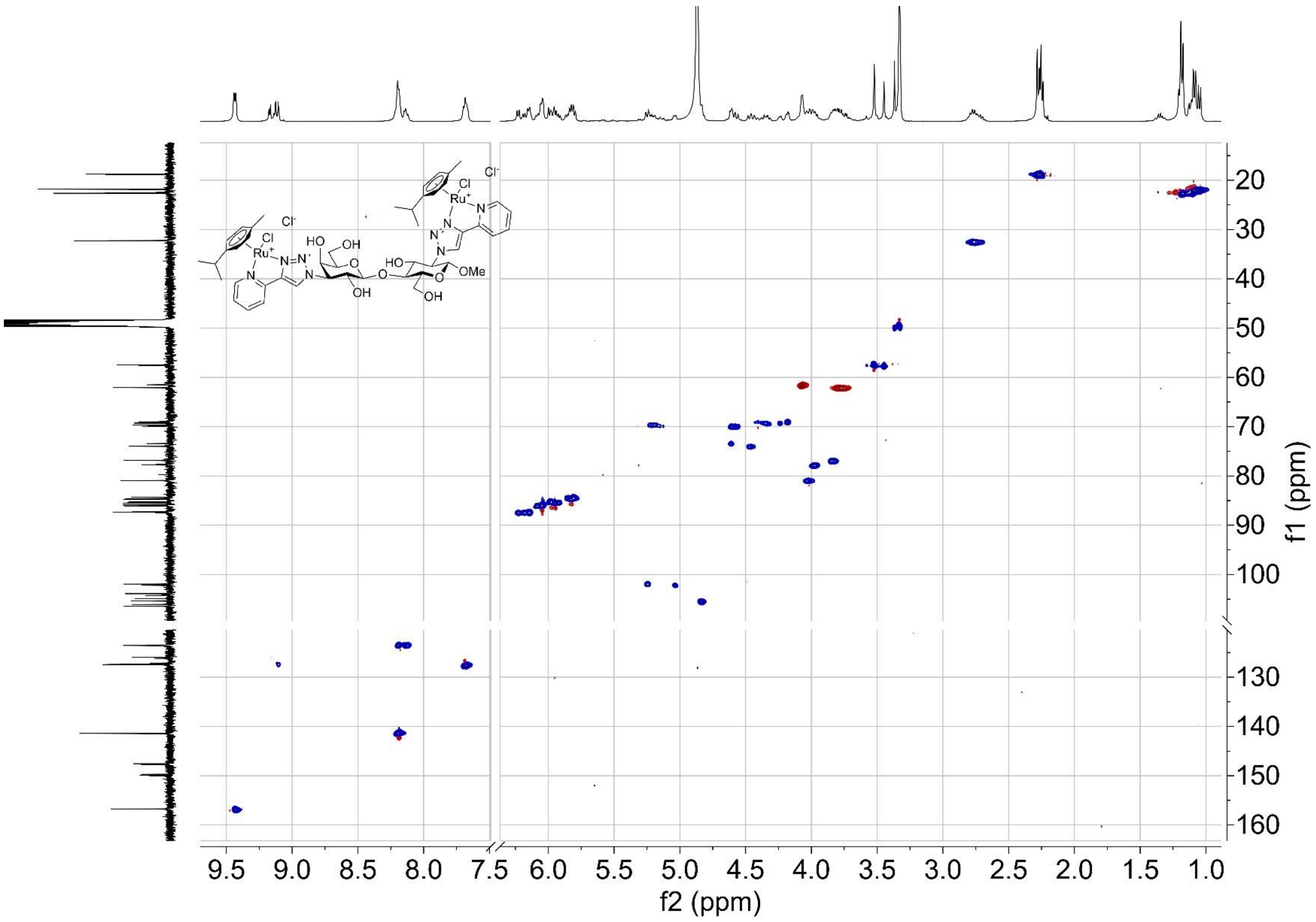

^{1}H-^{13}C HMBC 14-Cl

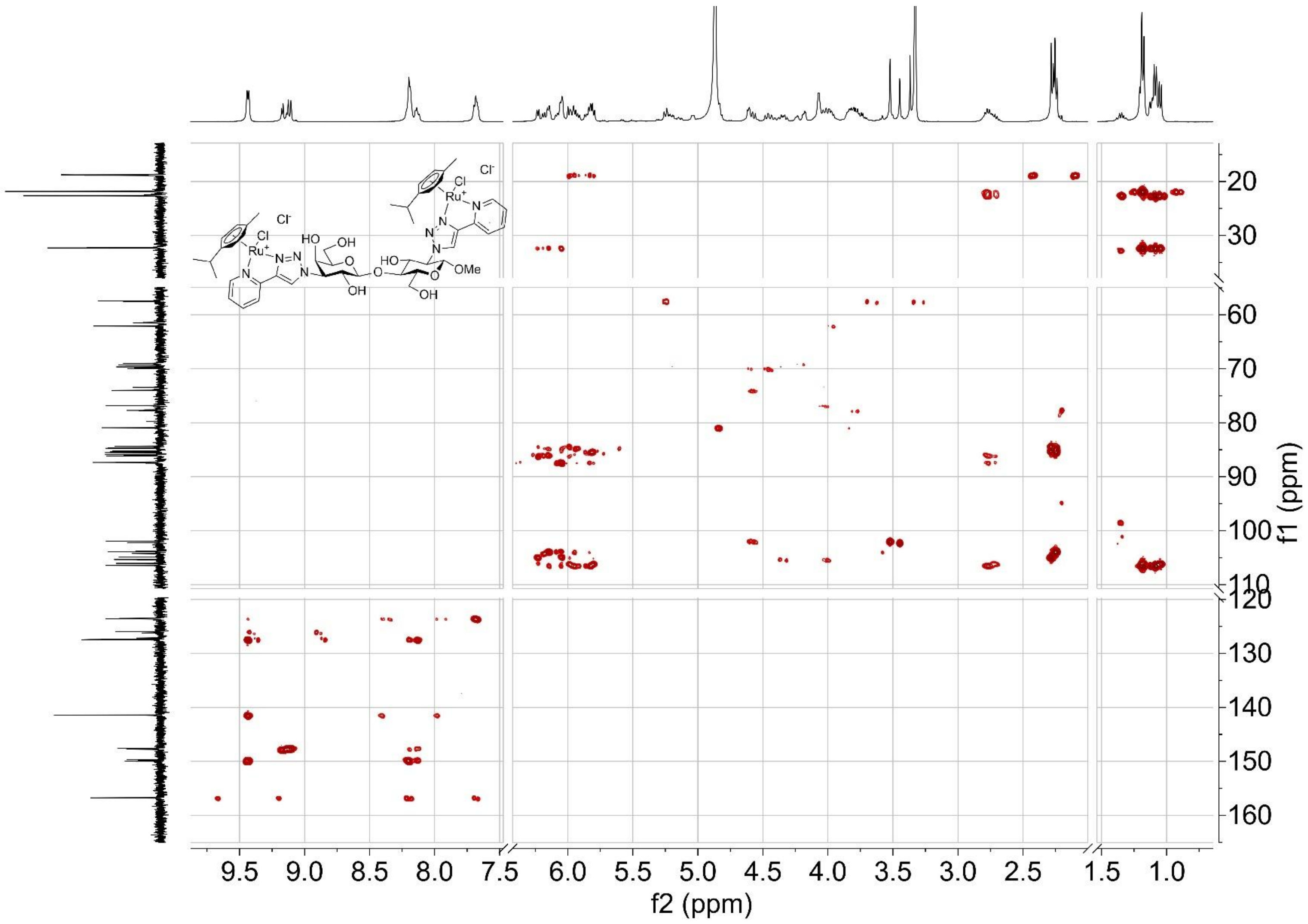

^{1}H-^{13}C HSQCTOCSY 14-Cl

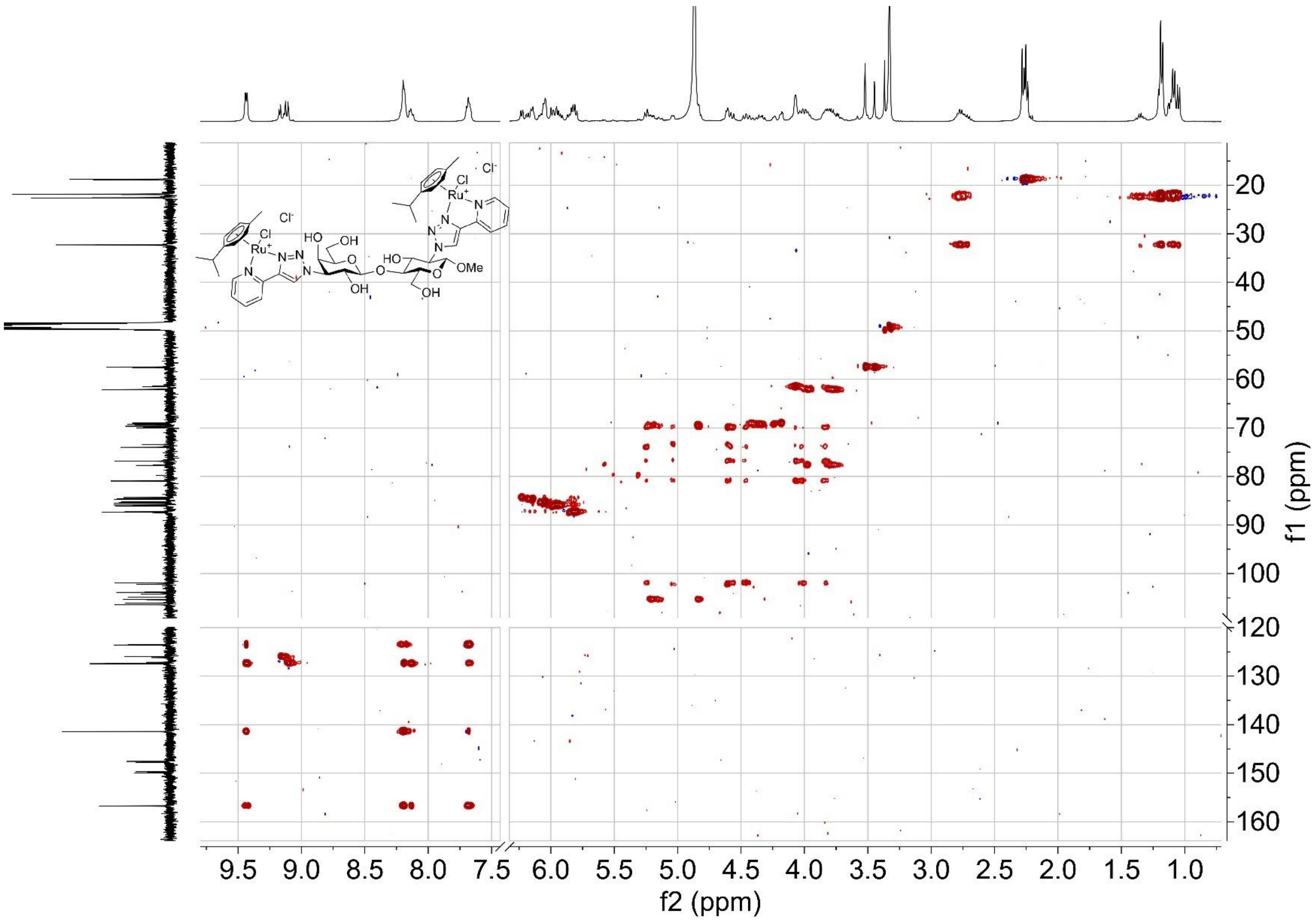

NMR COMPOUND 15-Cl

^{1}H NMR (400 MHz, CD_3OD) 15-Cl

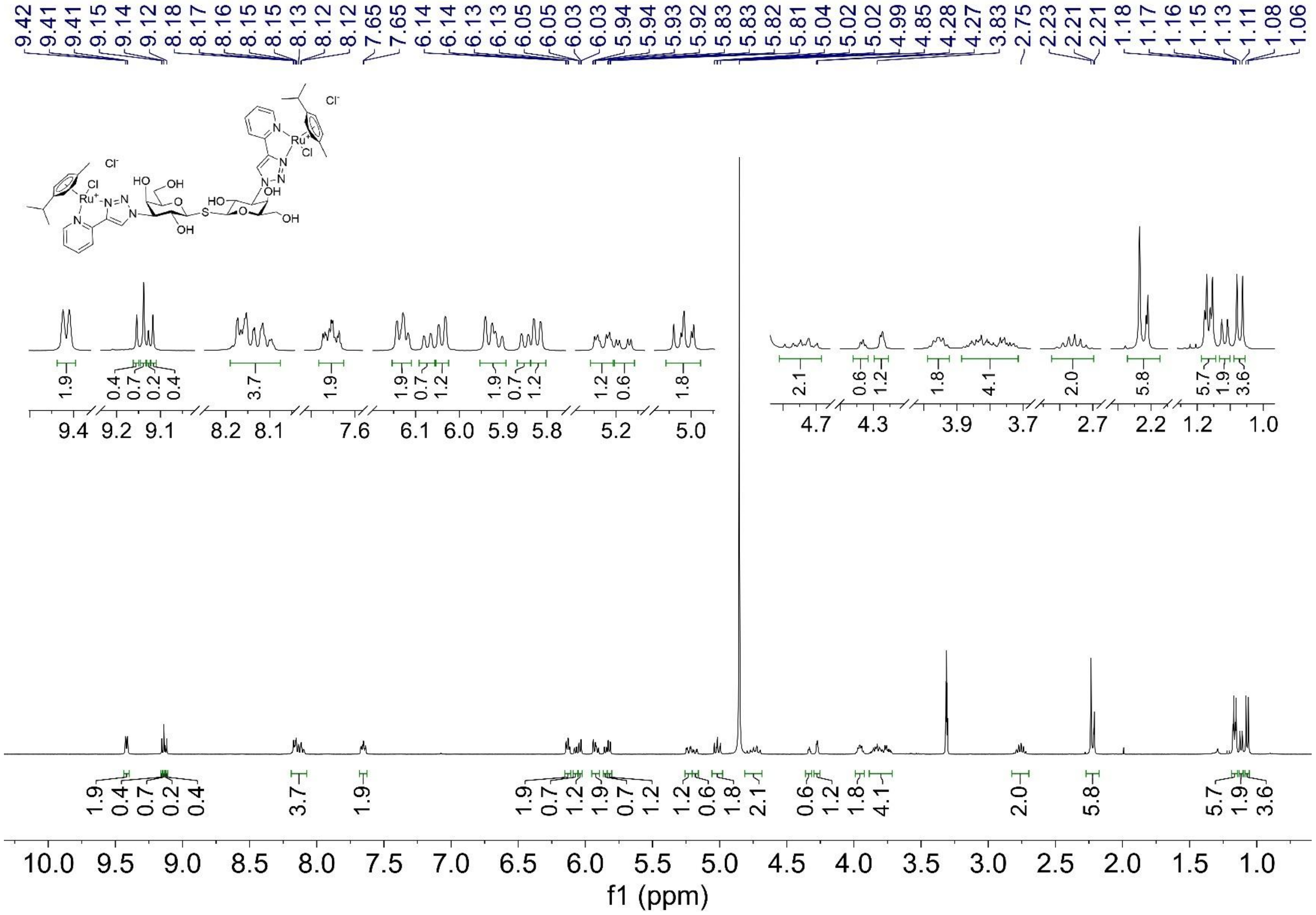

^{13}C NMR (101 MHz, CD_3OD) 15-Cl

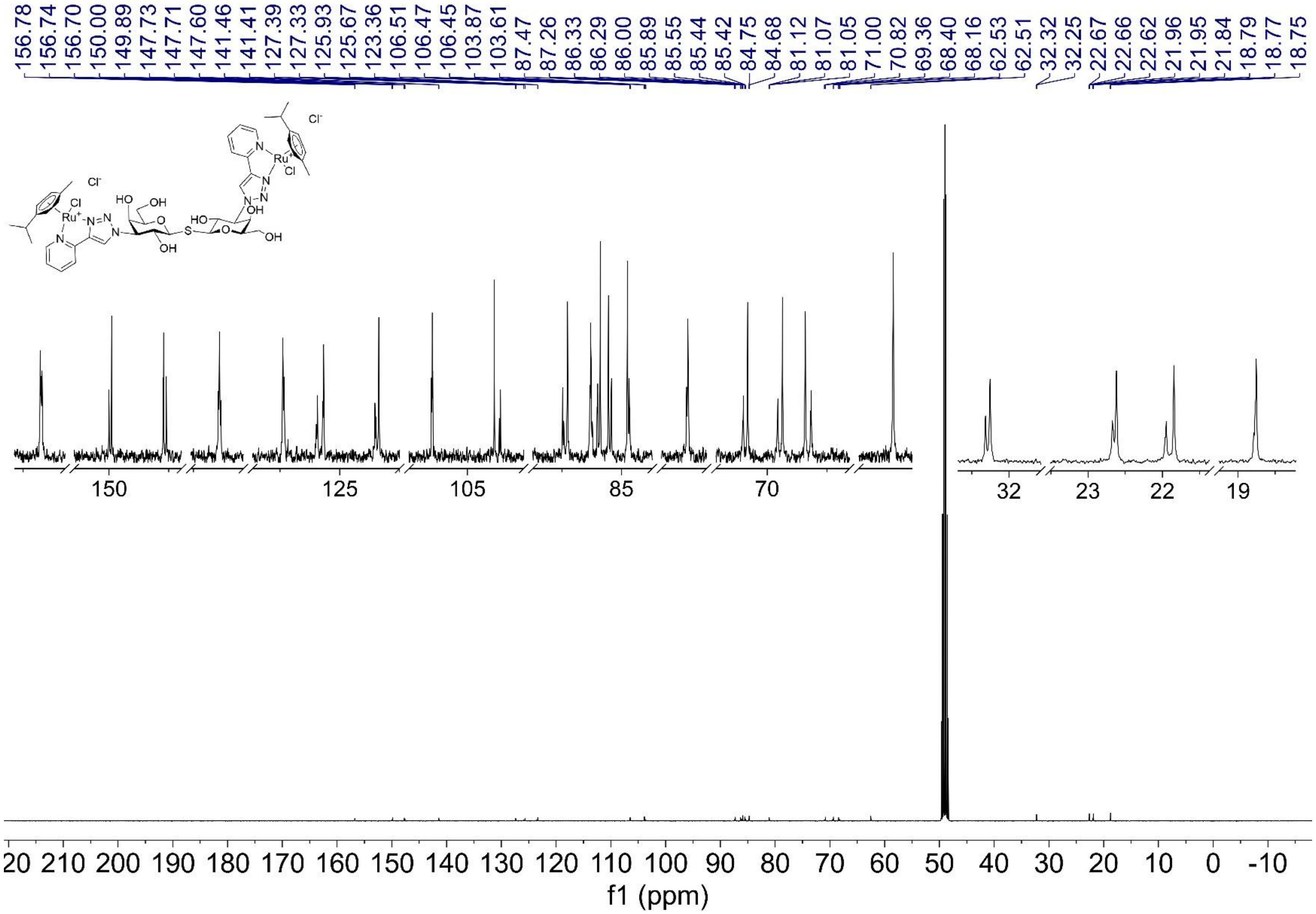

^{1}H-^{1}H COSY 15-Cl

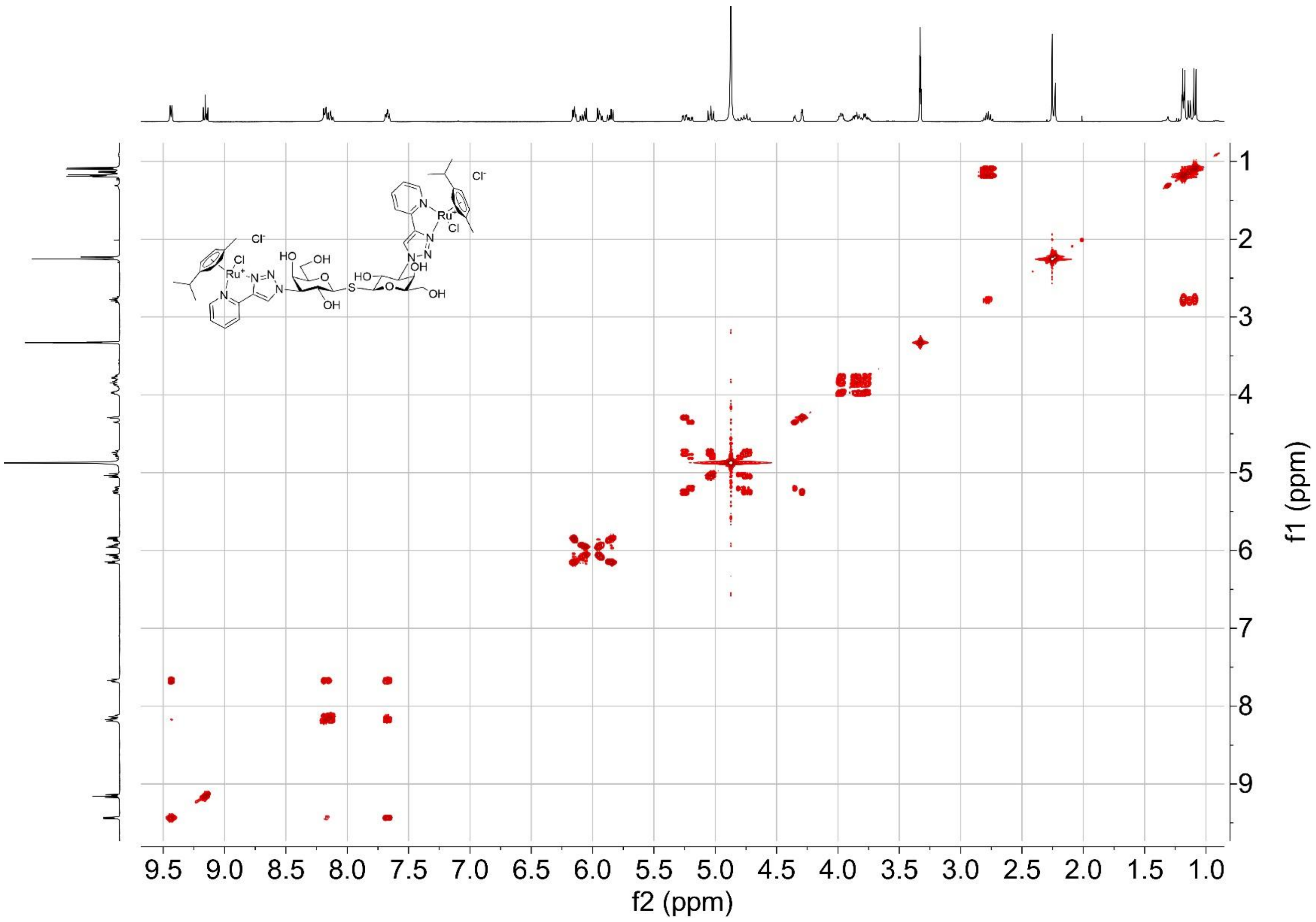

^{1}H-^{13}C HSQC 15-Cl

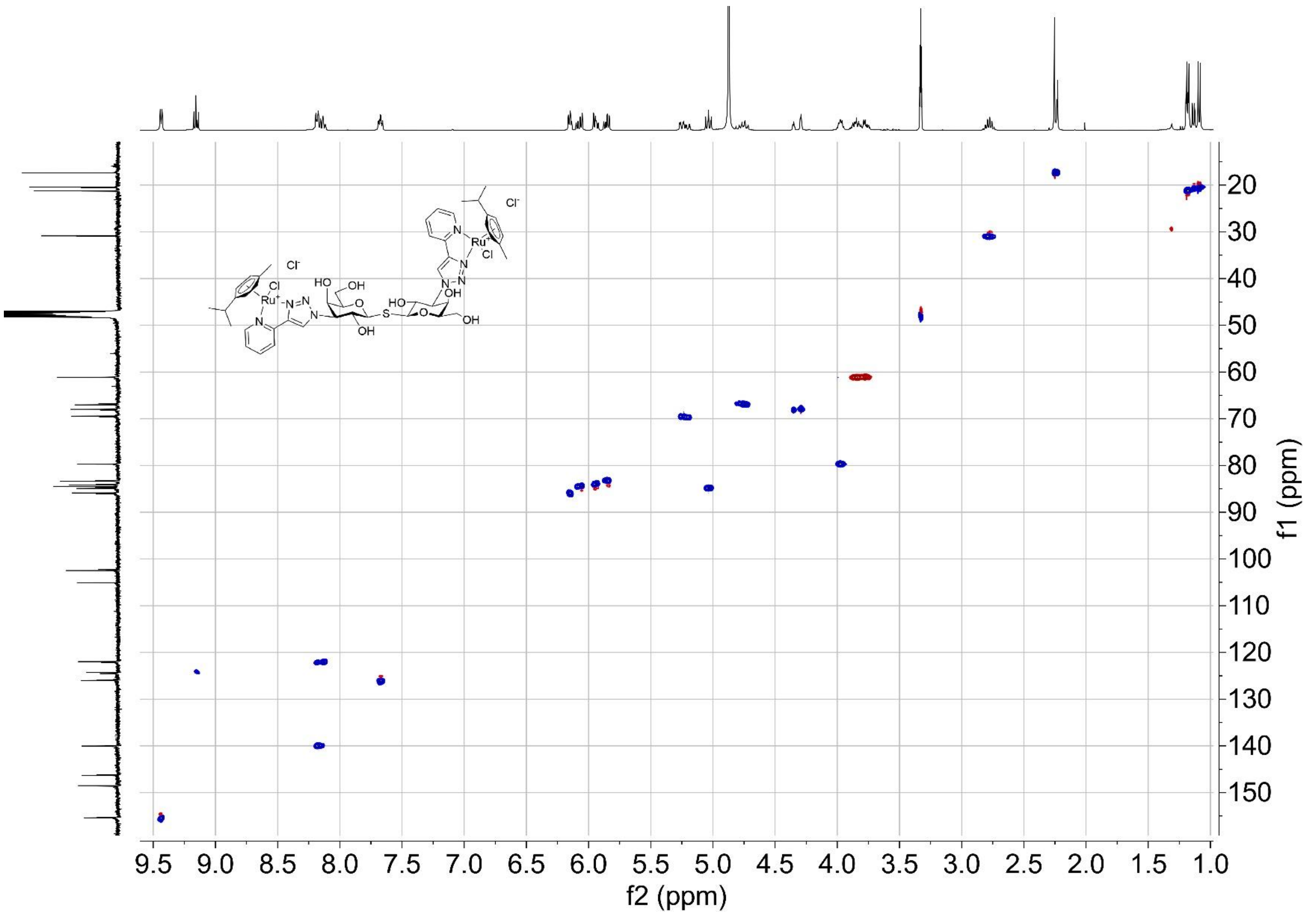

^{1}H-^{13}C HMBC 15-Cl

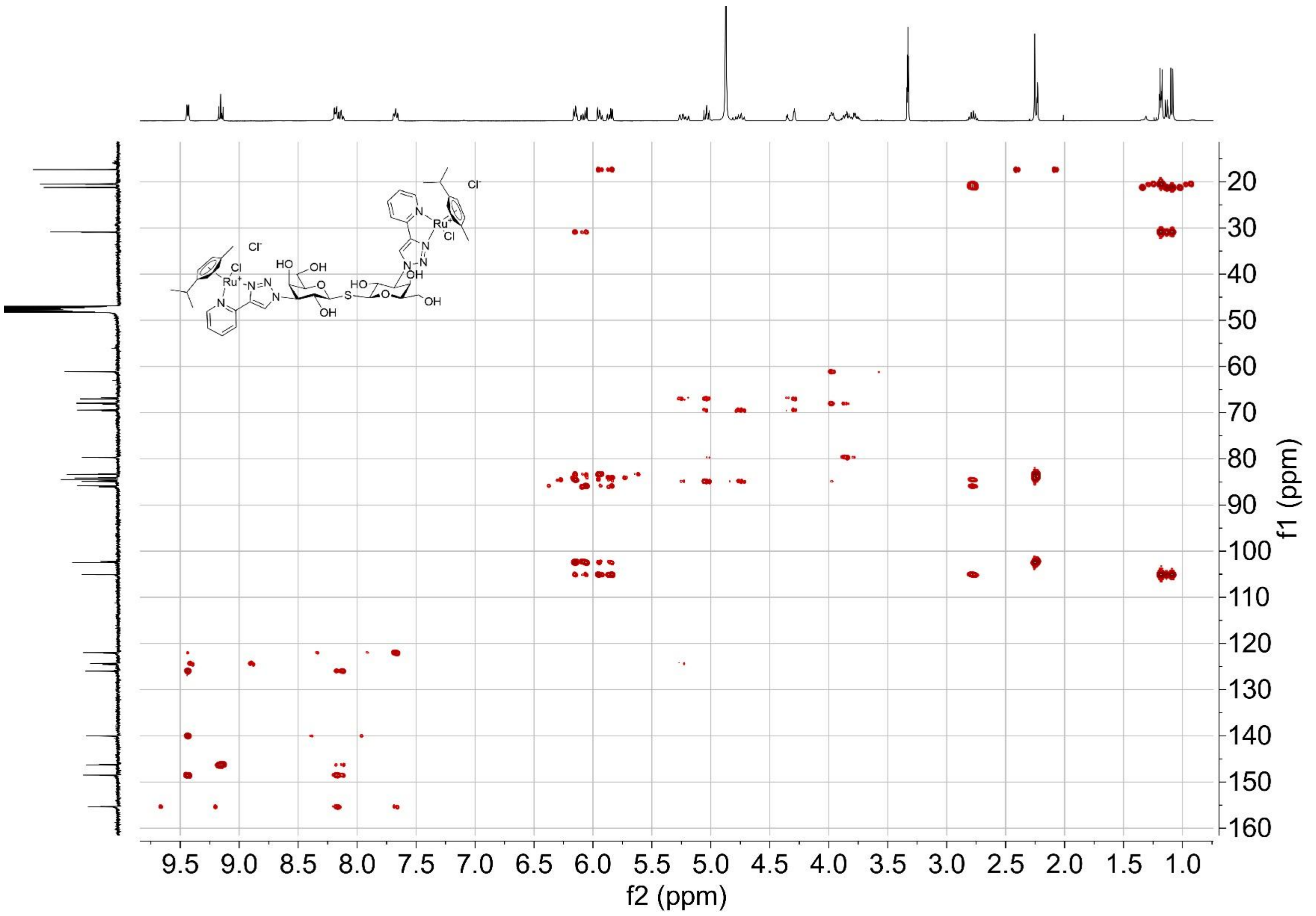

^{1}H-^{13}C HSQCTOCSY 15-Cl

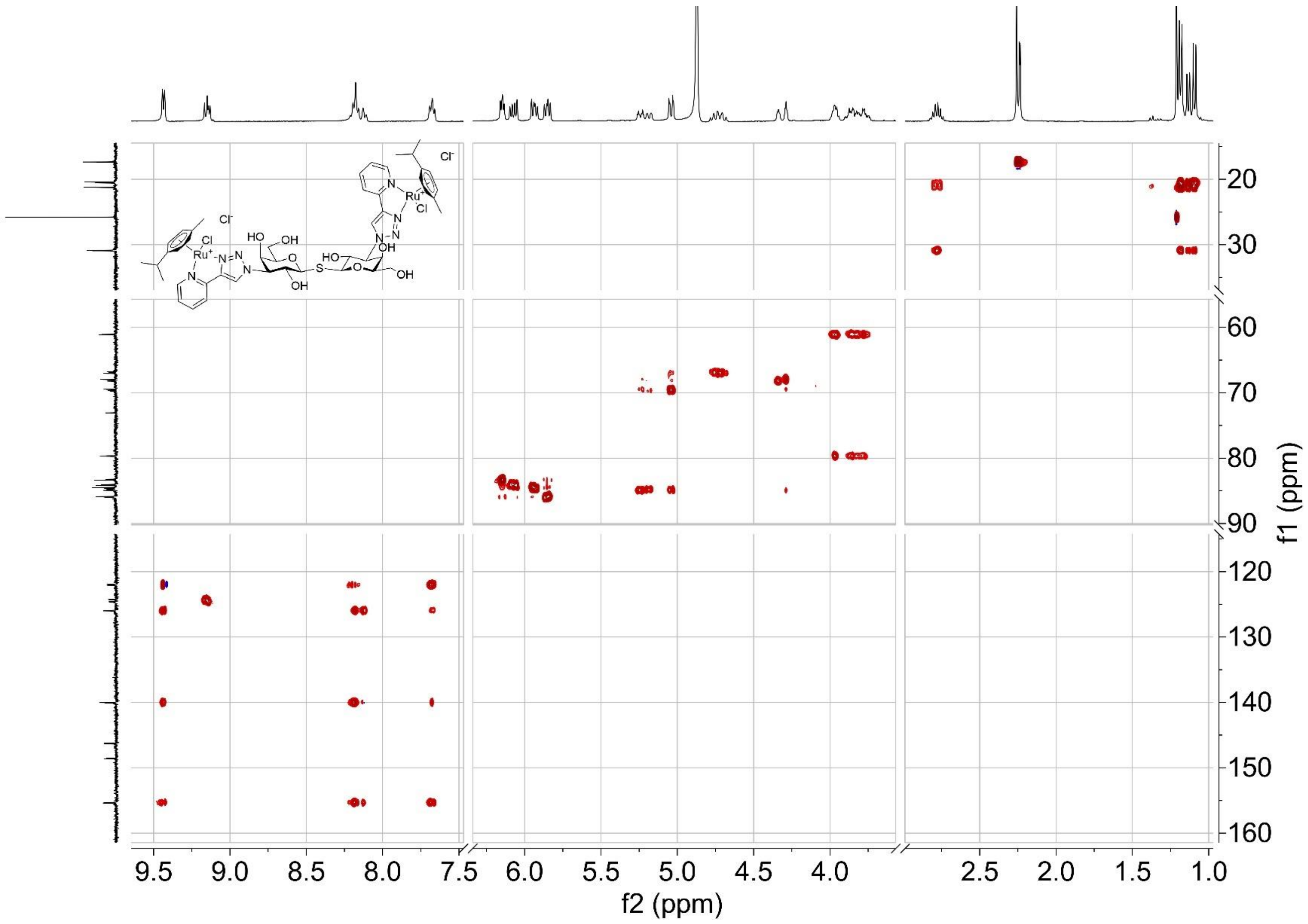

NMR COMPOUND 15-BF

^{1}H NMR (400 MHz, CD_3OD) 15-BF

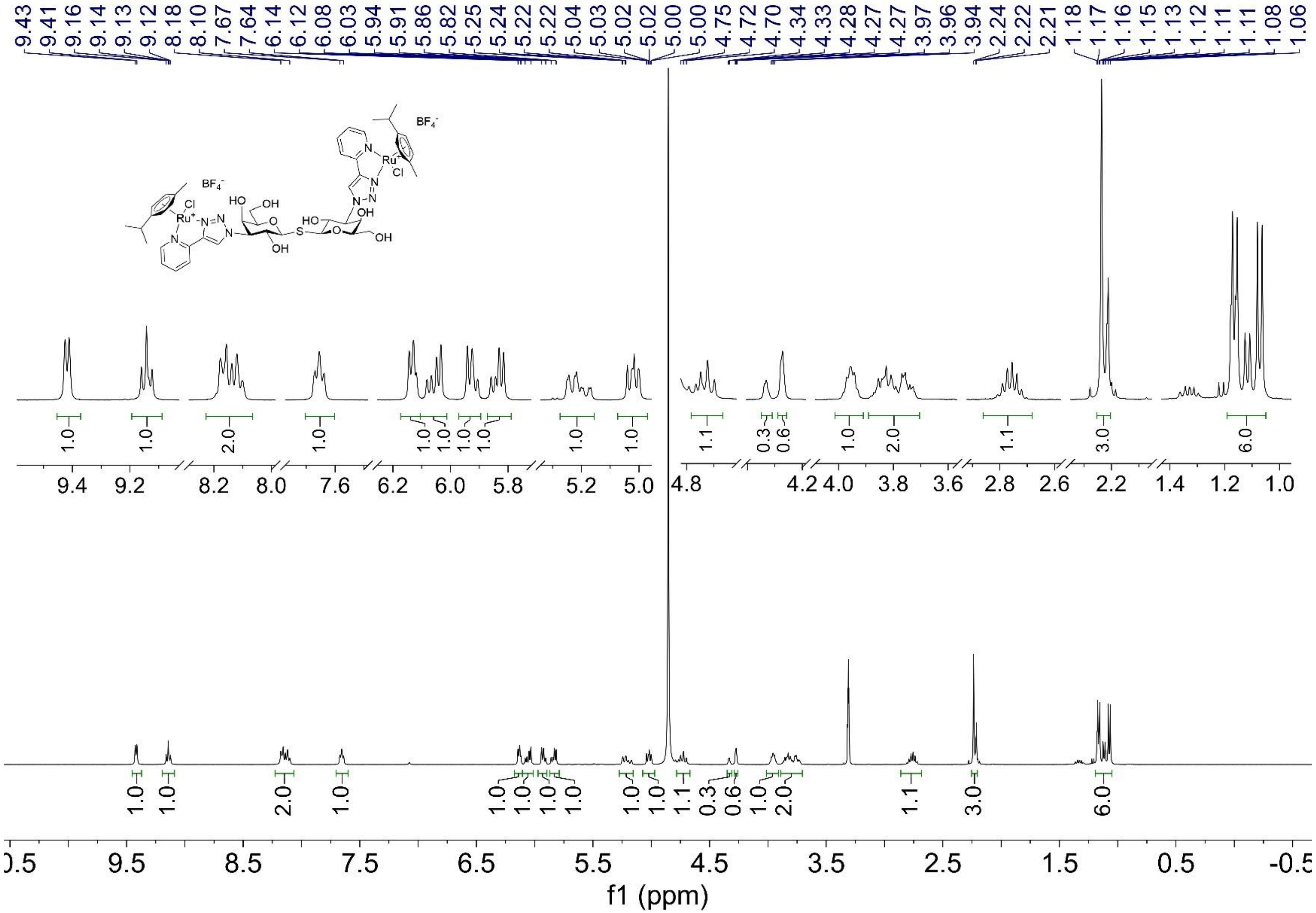

^{13}C NMR (101 MHz, CD_3OD) 15-BF

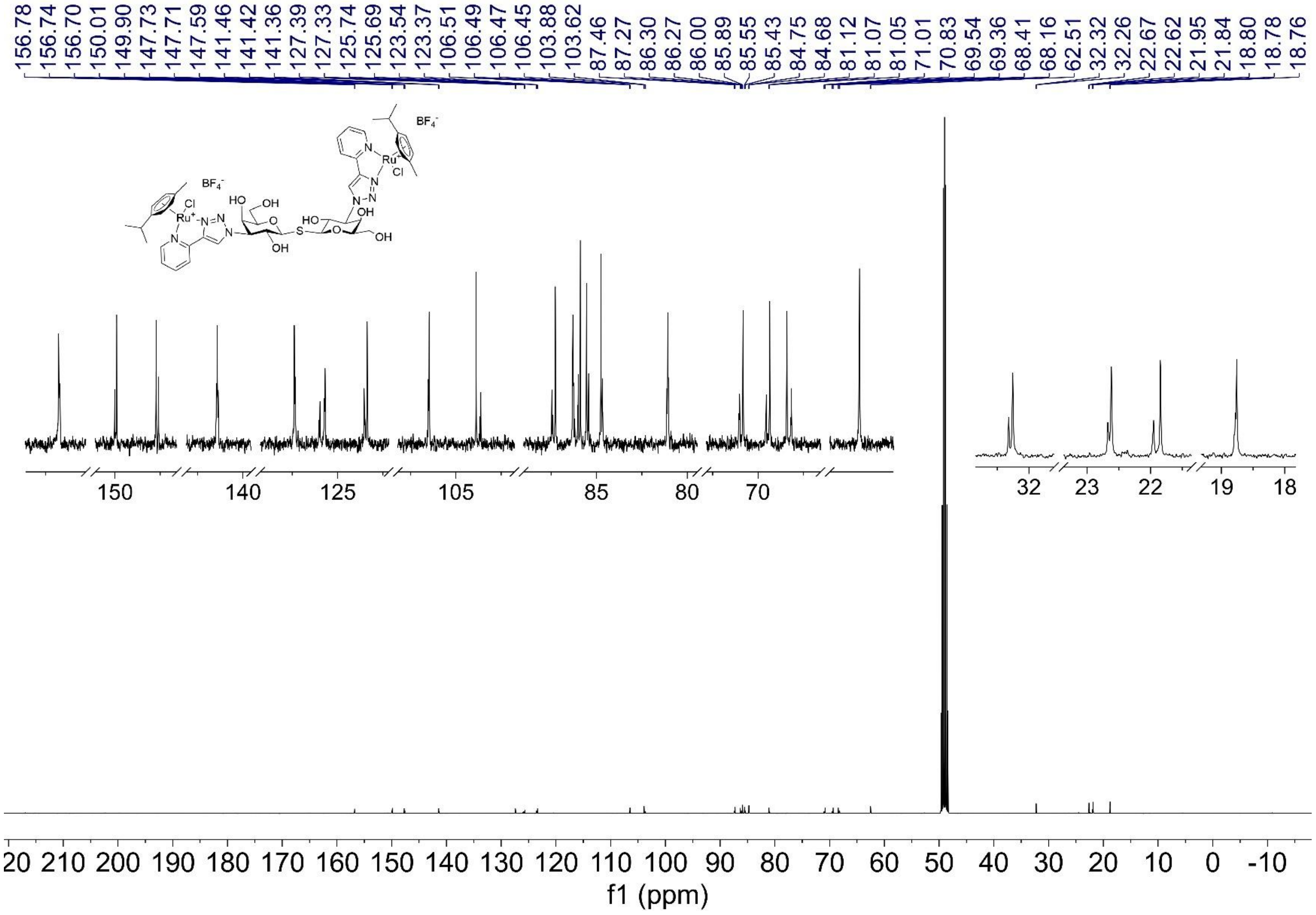

^{19}F NMR (376 MHz, CD_3OD) 15-BF

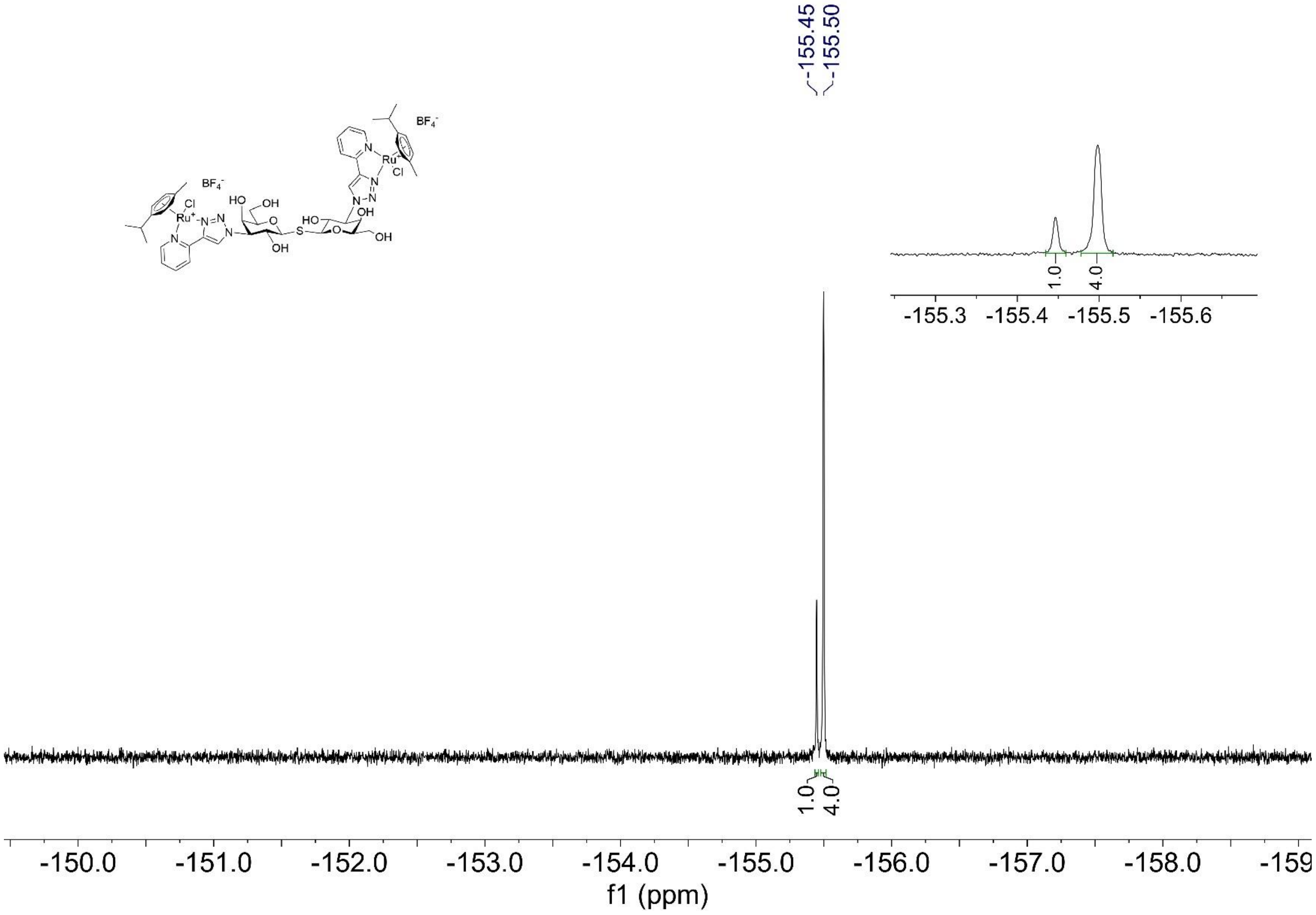

^{1}H-^{1}H COSY 15-BF

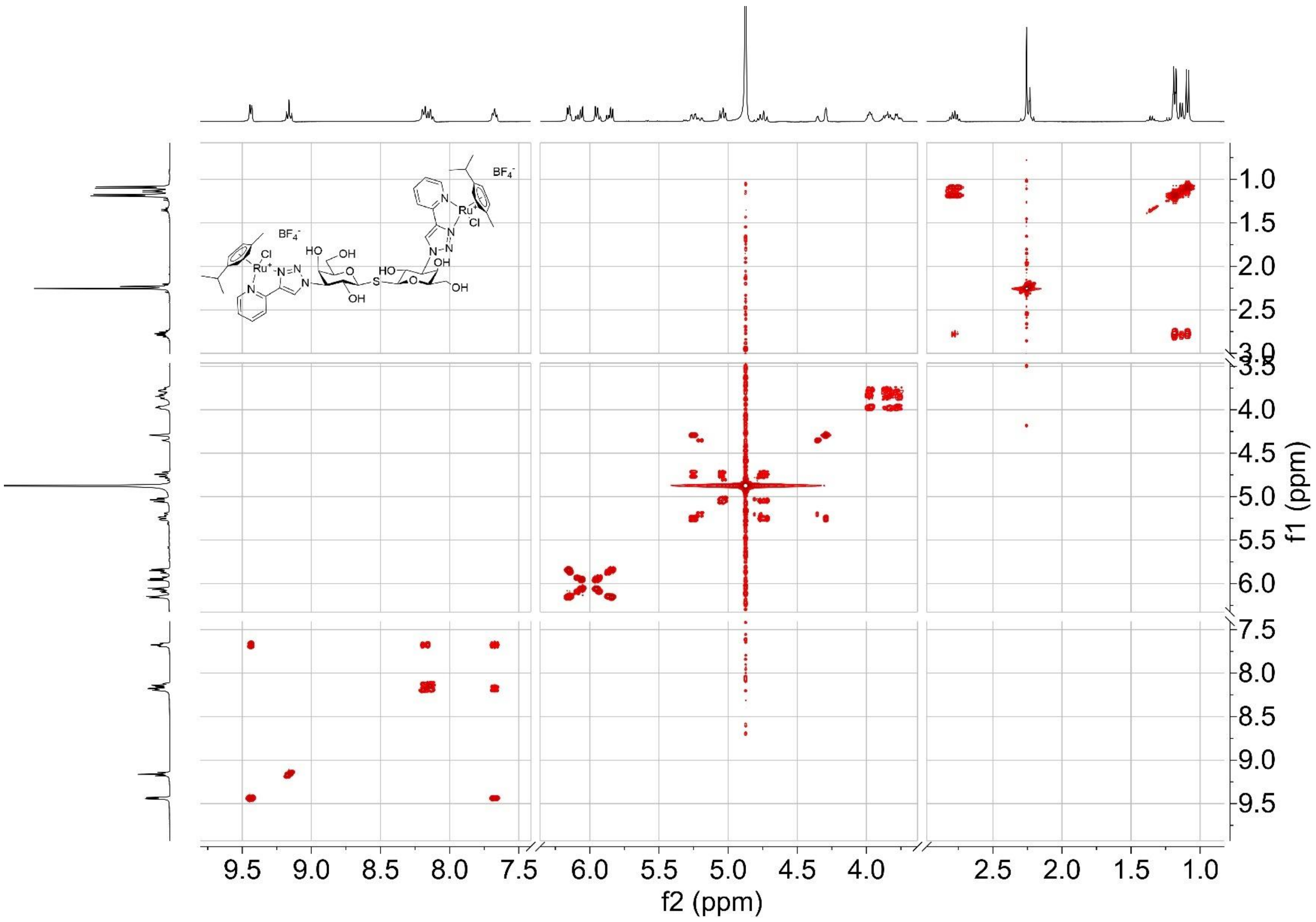

^{1}H-^{13}C HSQC 15-BF

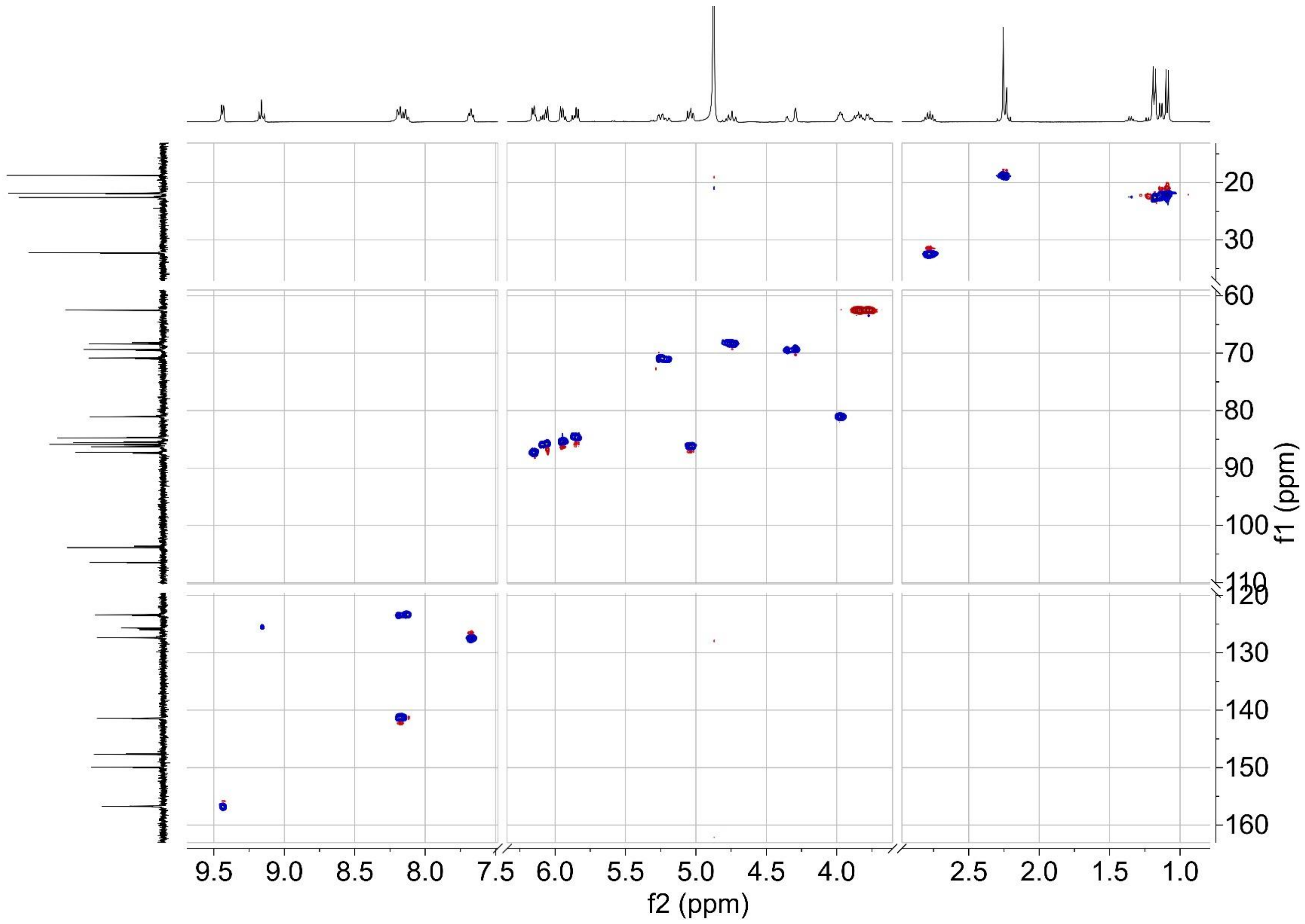

^{1}H-^{13}C HMBC 15-BF

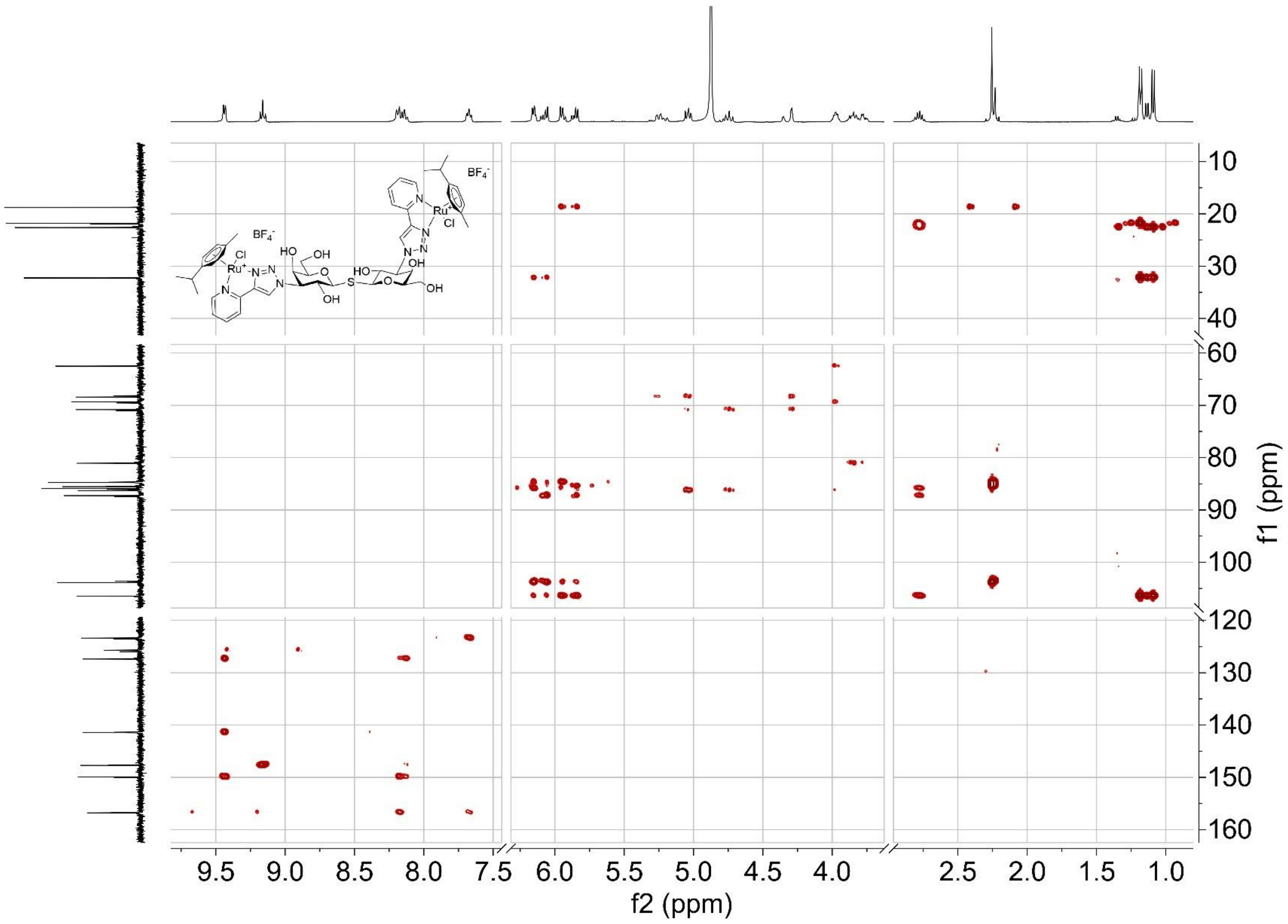

^{1}H-^{13}C HSQCTOCSY 15-BF

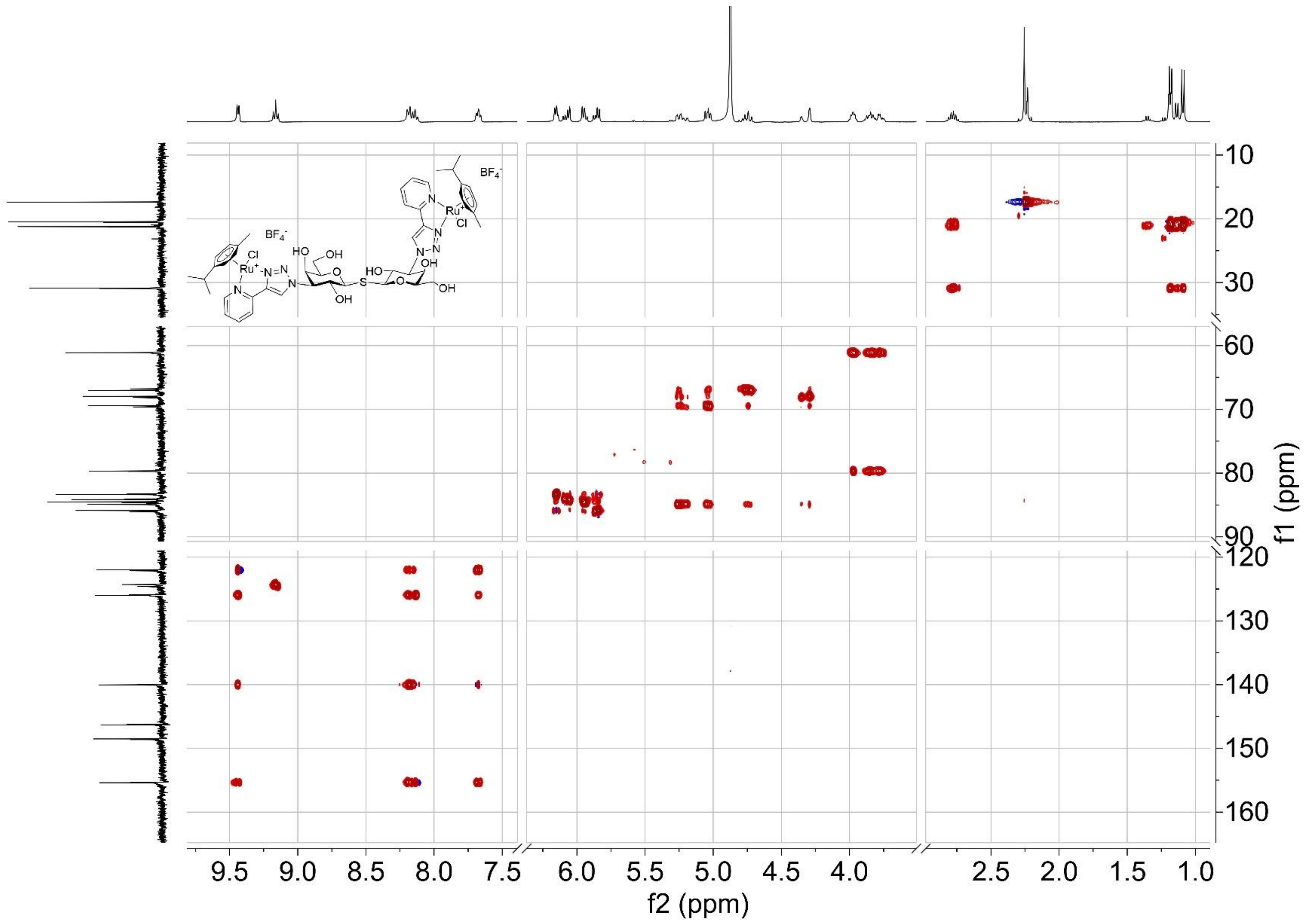

NMR COMPOUND 15-PF

^{1}H NMR (400 MHz, D_2O) 15-PF

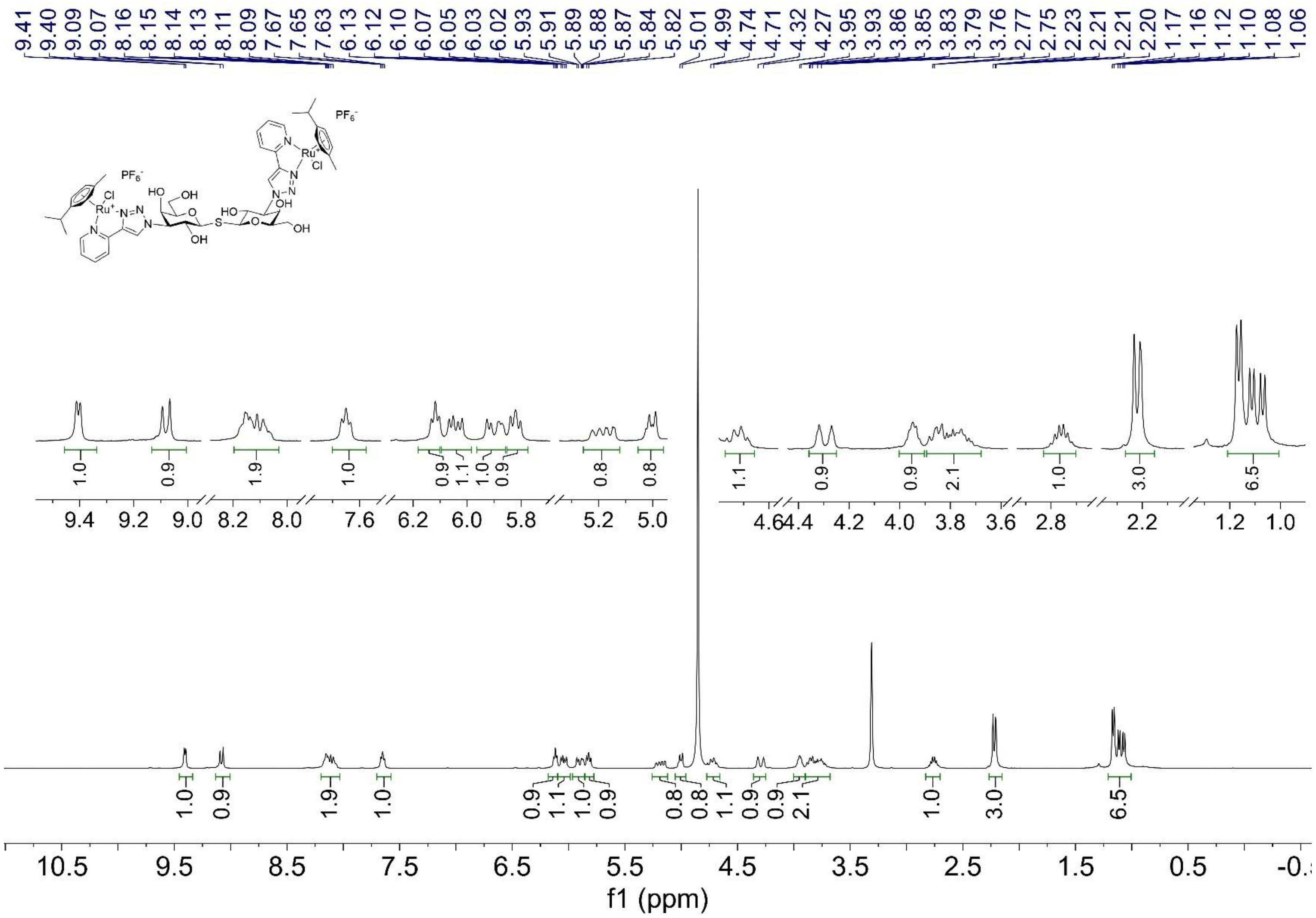

^{13}C NMR (101 MHz, D_2O) 15-PF

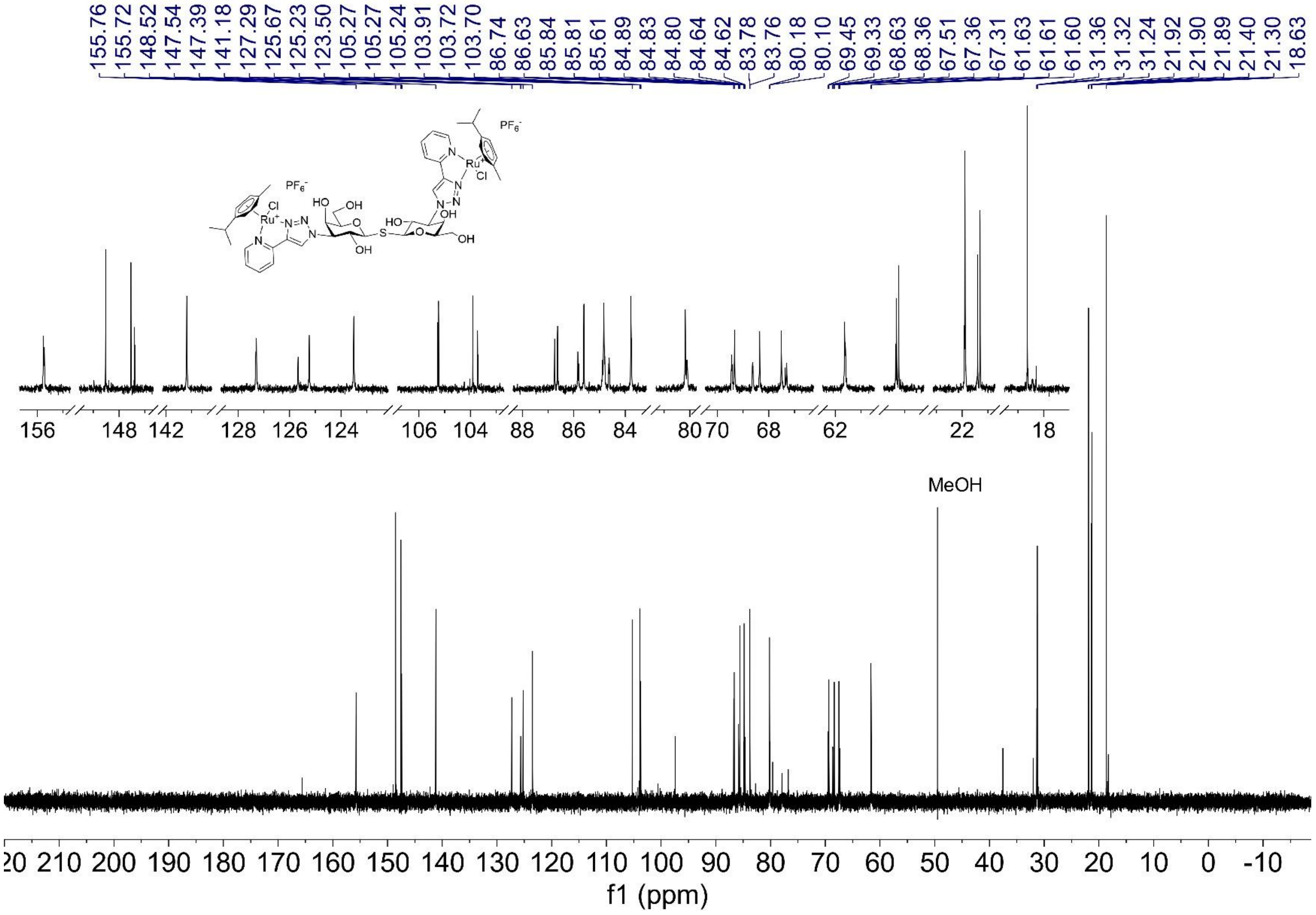

^{19}F NMR (376 MHz, D_2O) 15-PF

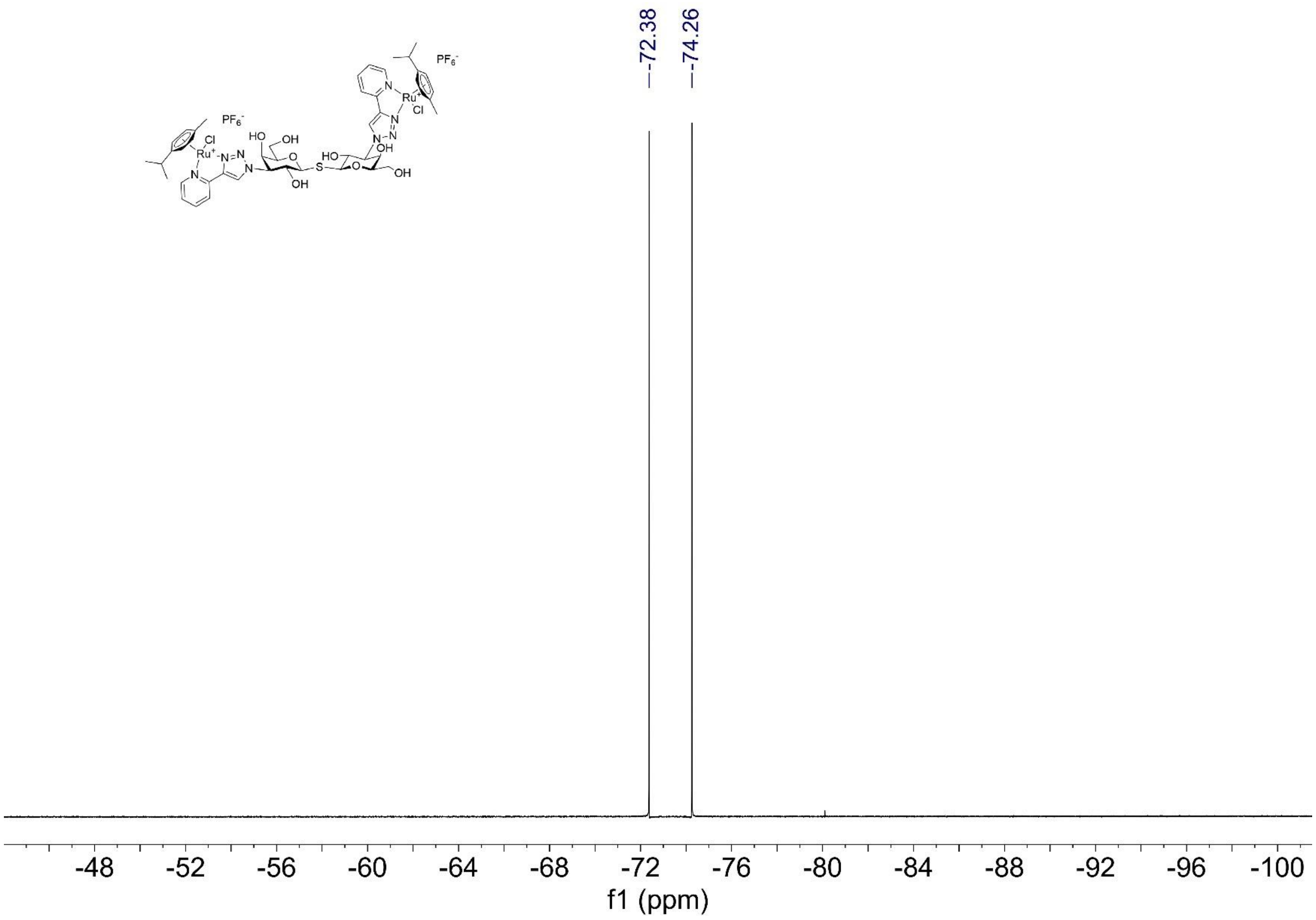

^{31}P NMR (162 MHz, D_2O) 15-PF

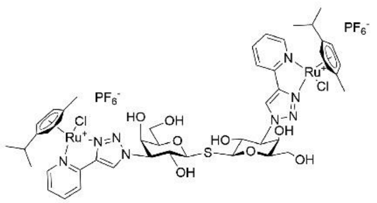

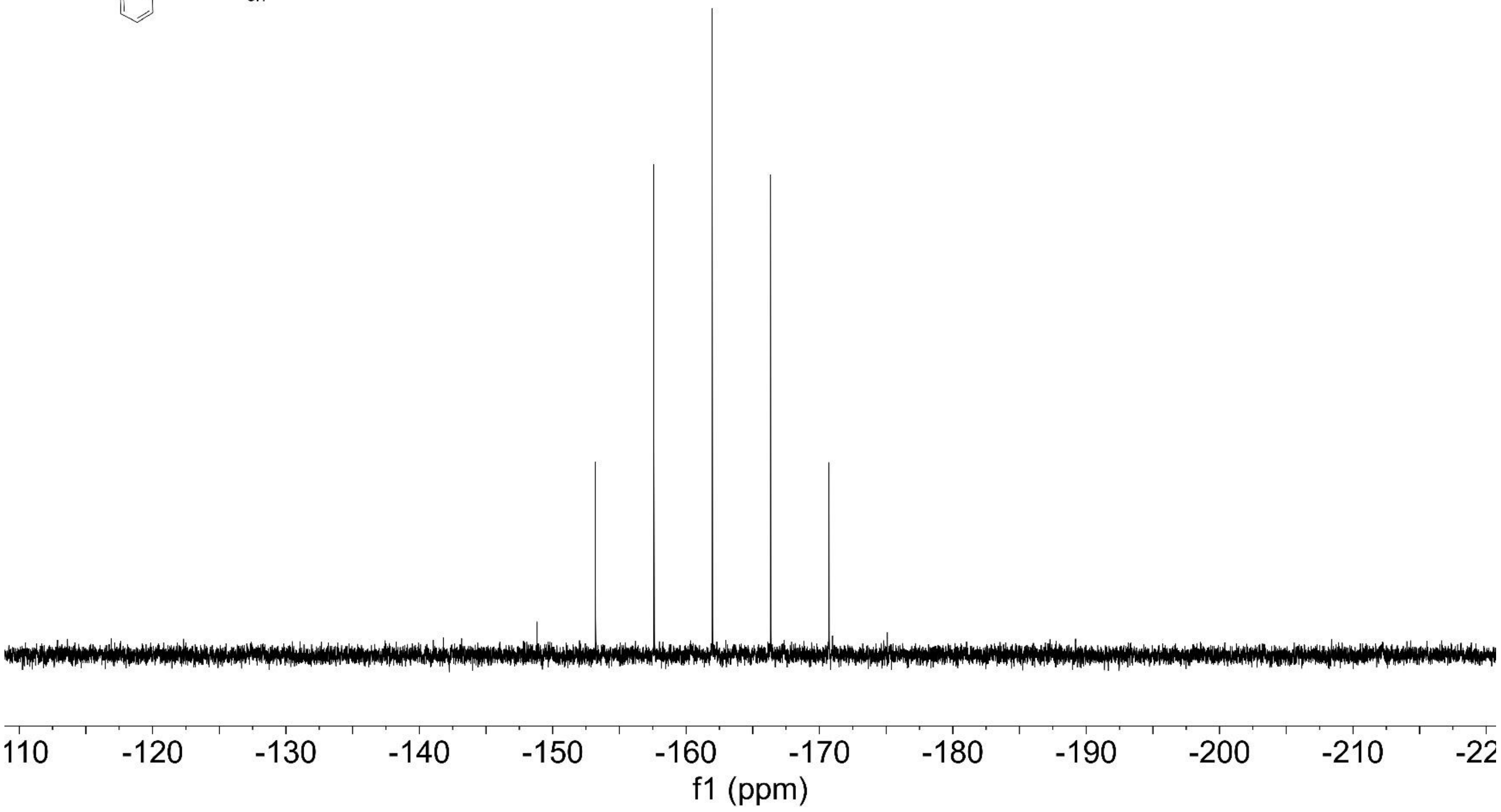

^{1}H-^{1}H COSY 15-PF

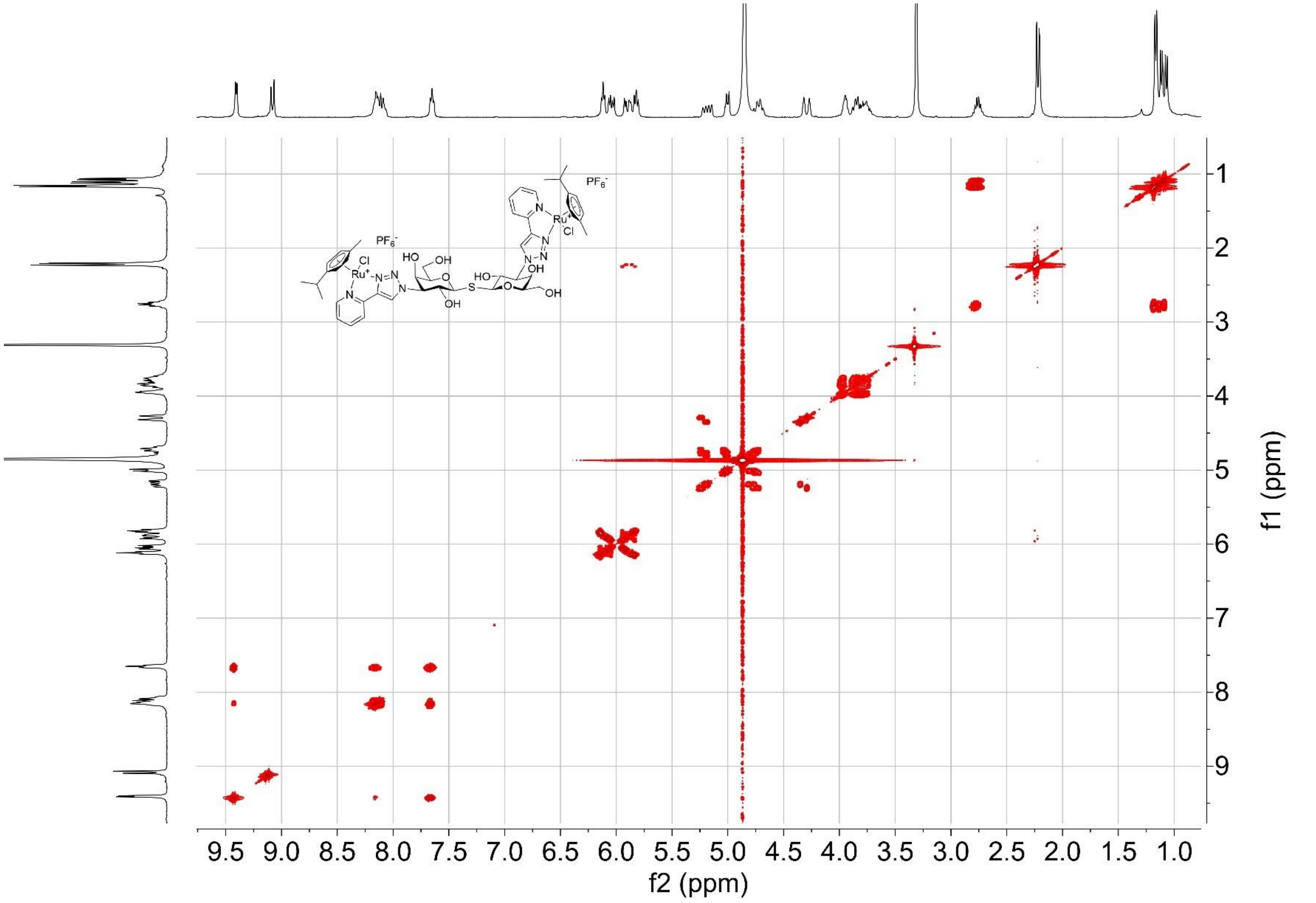

^{1}H-^{13}C HSQC 15-PF

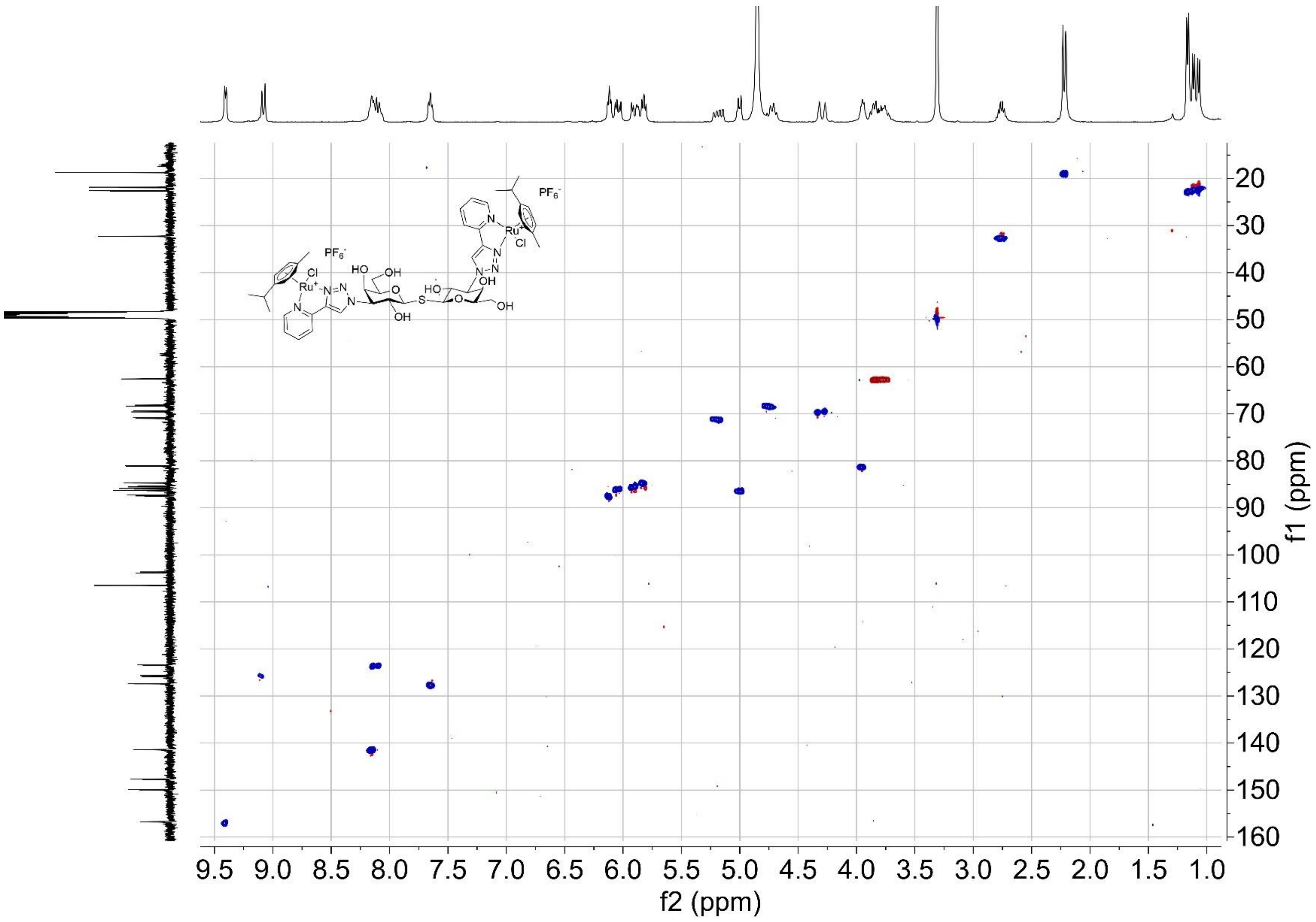

^{1}H-^{13}C HMBC 15-PF

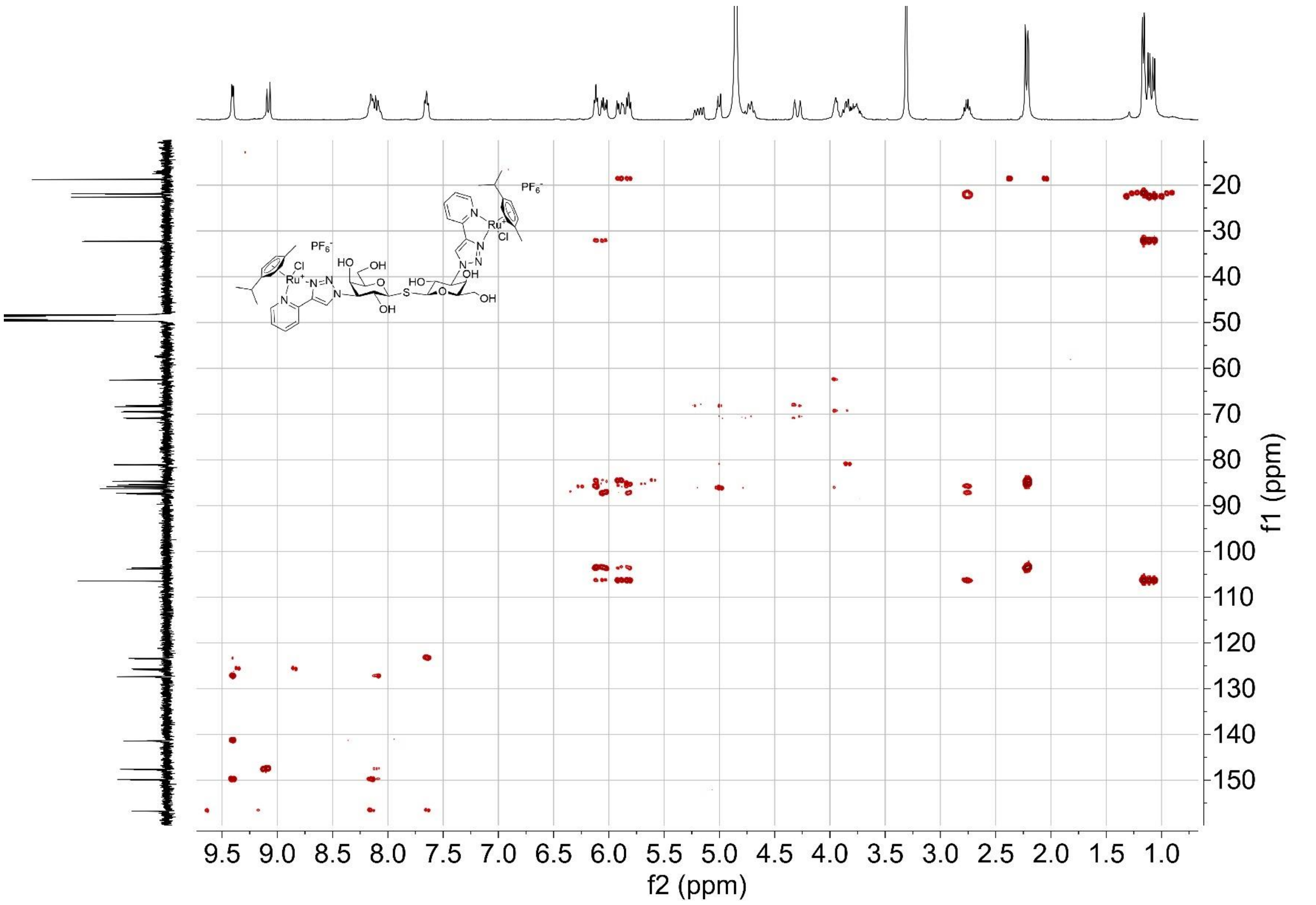

^{1}H-^{13}C HSQCTOCSY 15-PF

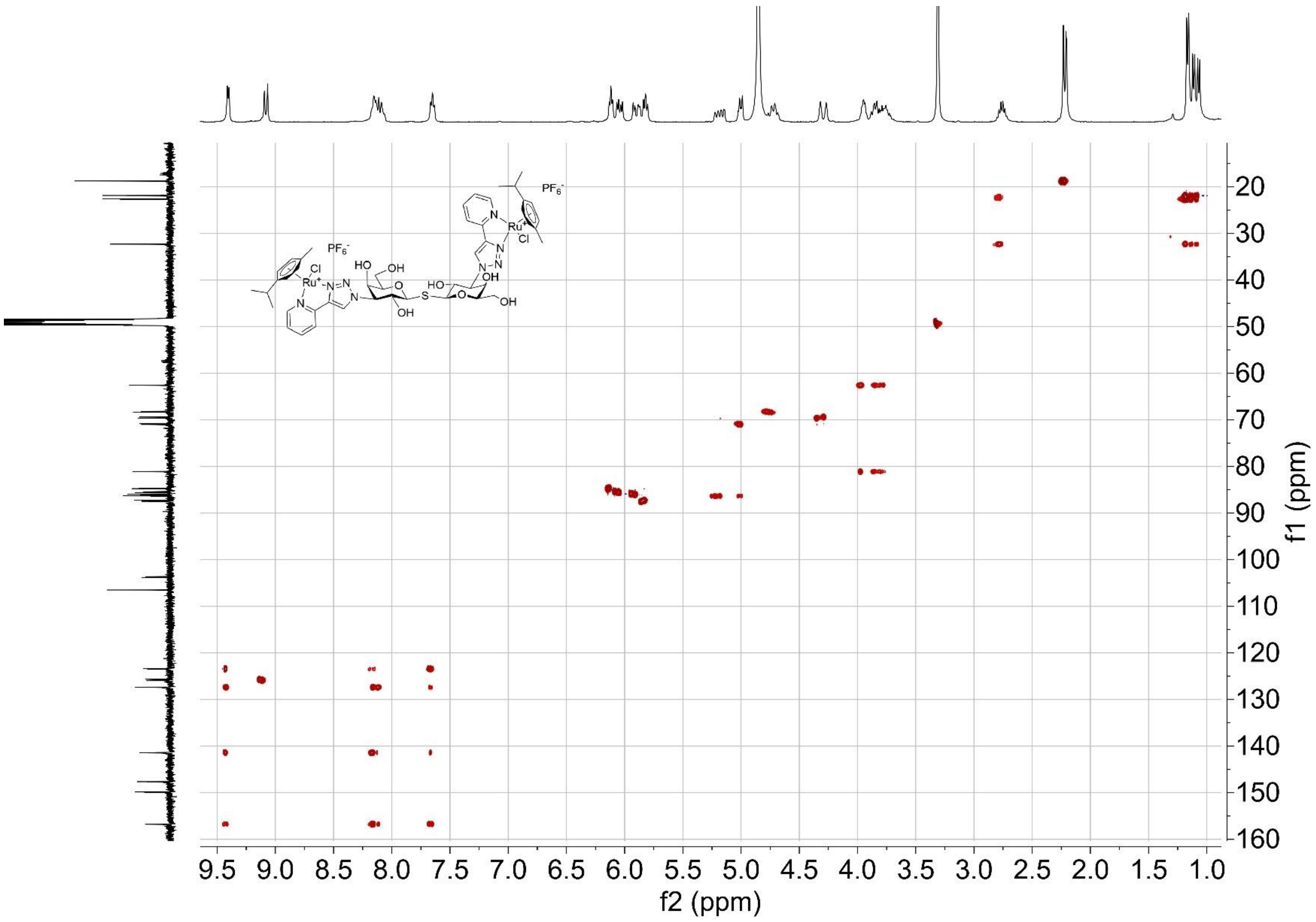

NMR COMPOUND 16-I

^{1}H NMR (400 MHz, CD_3OD) 16-I

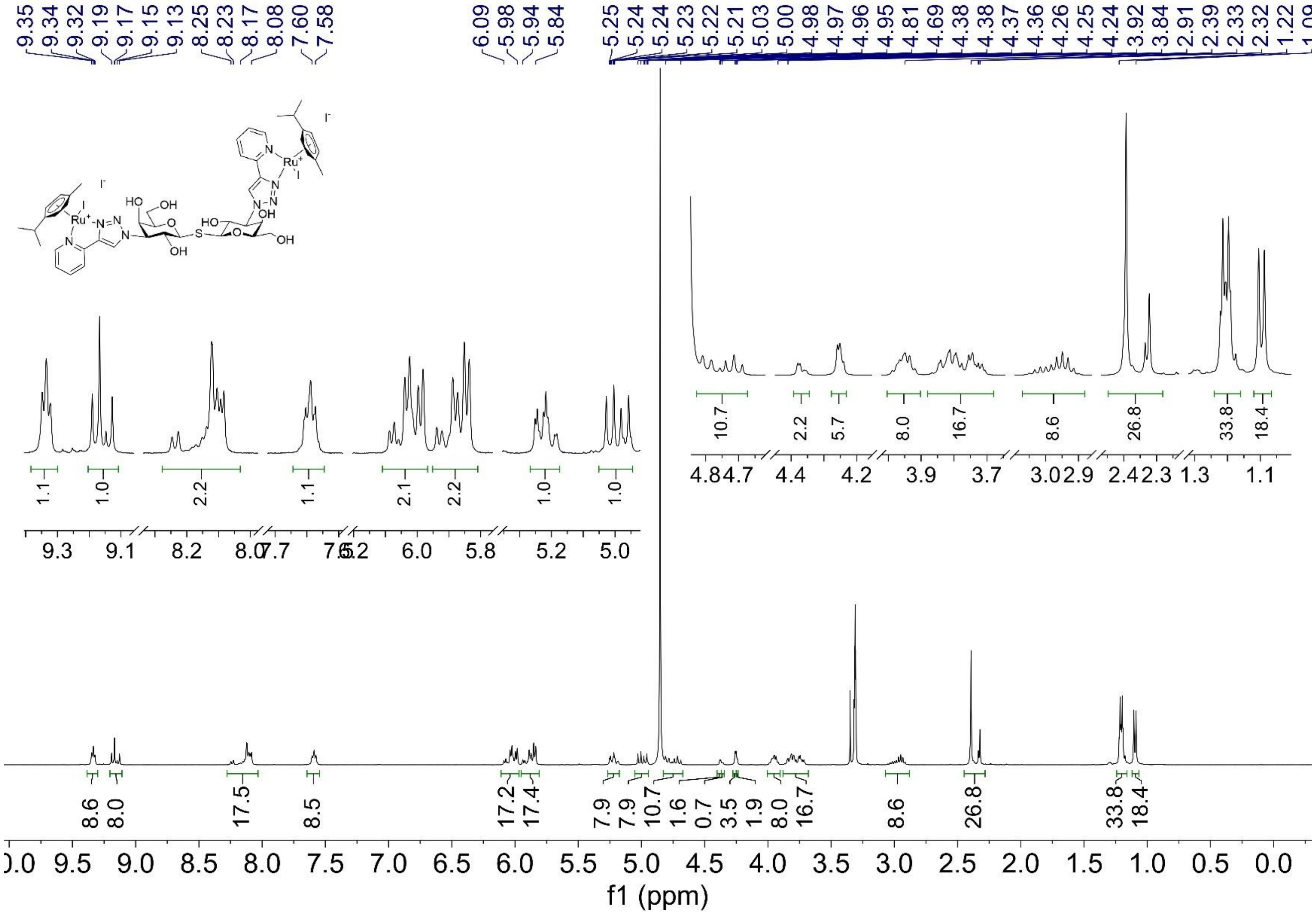

^{13}C NMR (101 MHz, CD_3OD) 16-I

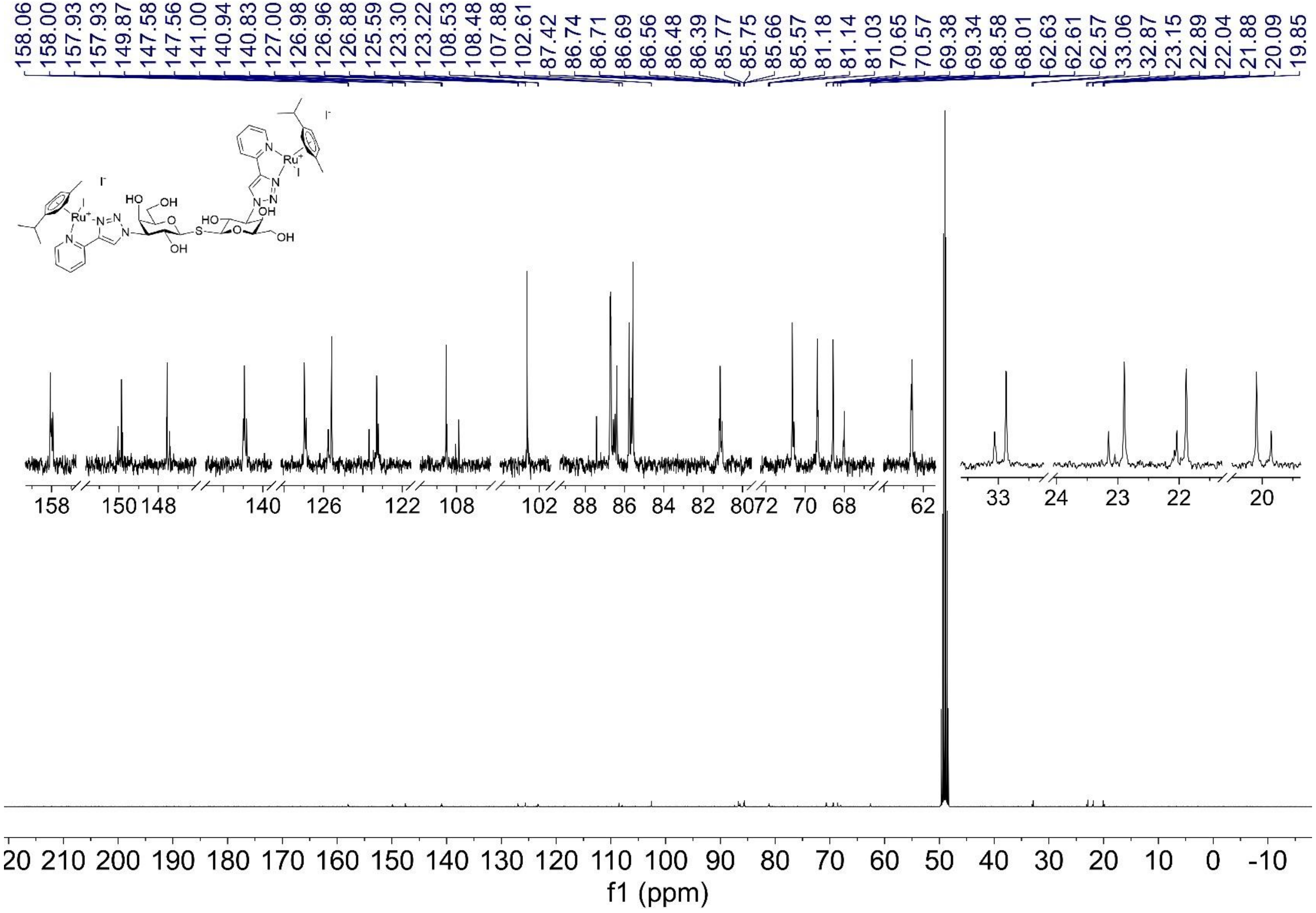

^{1}H-^{1}H COSY 16-I

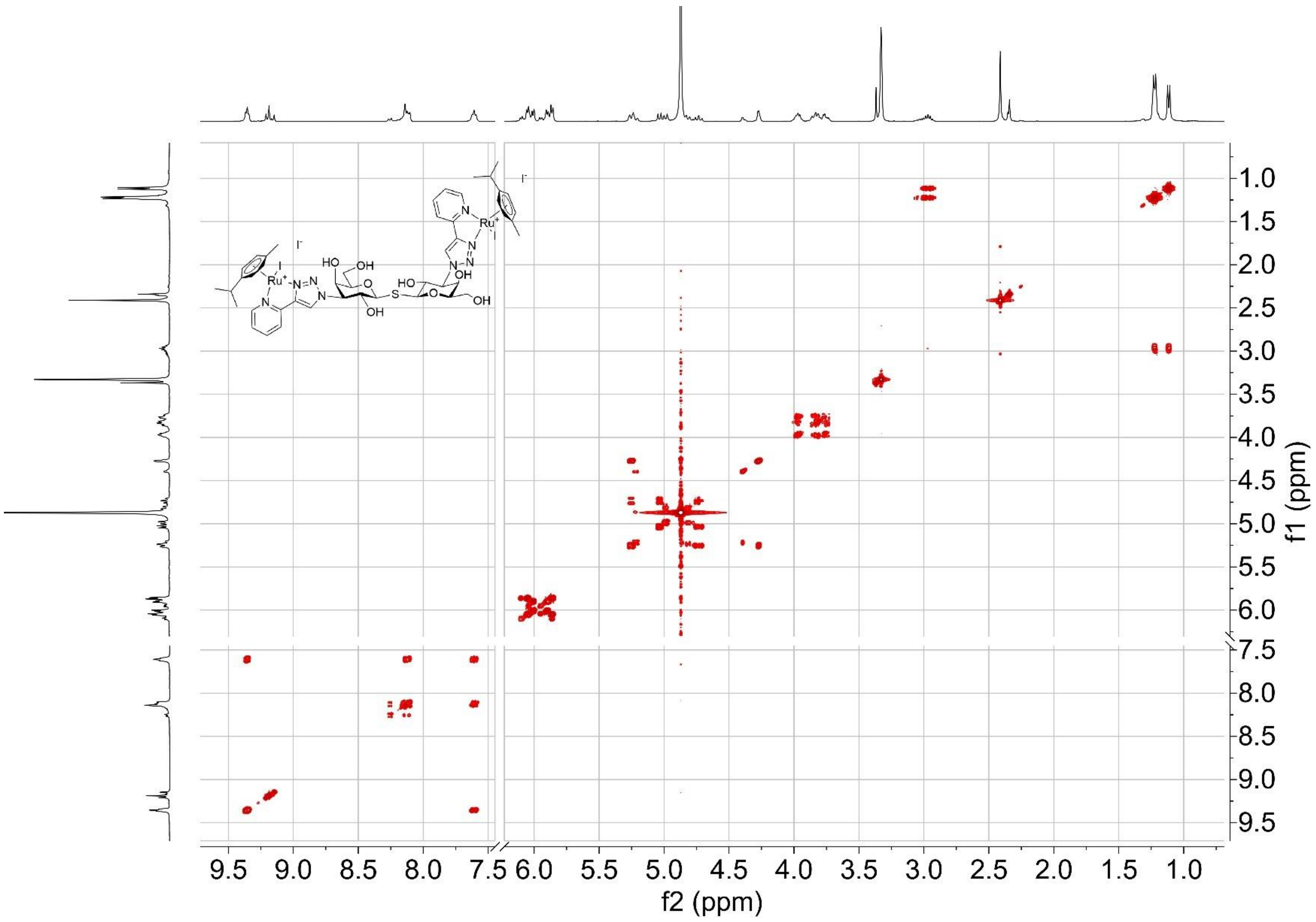

^{1}H-^{13}C HSQC 16-I

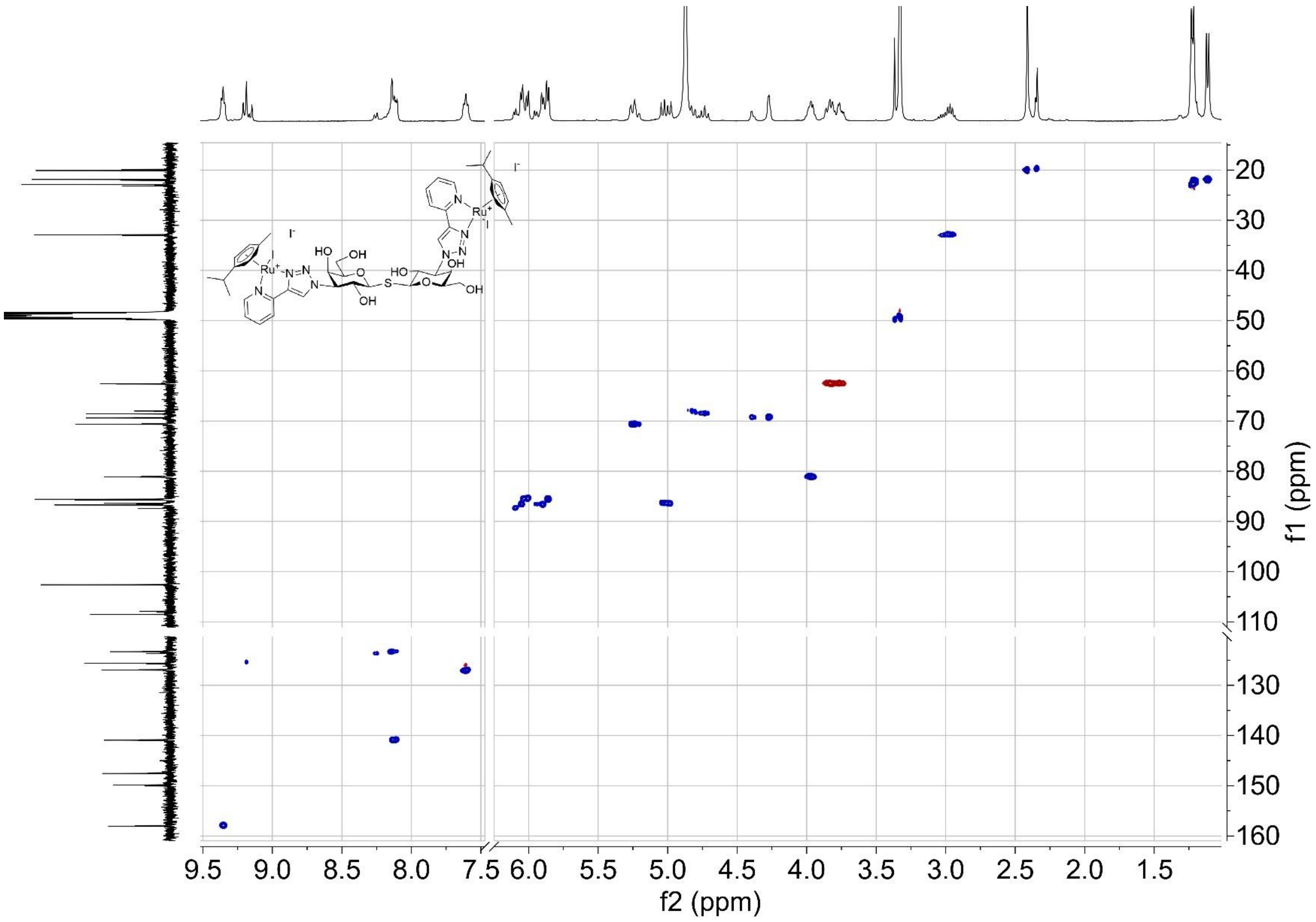

^{1}H-^{13}C HMBC 16-I

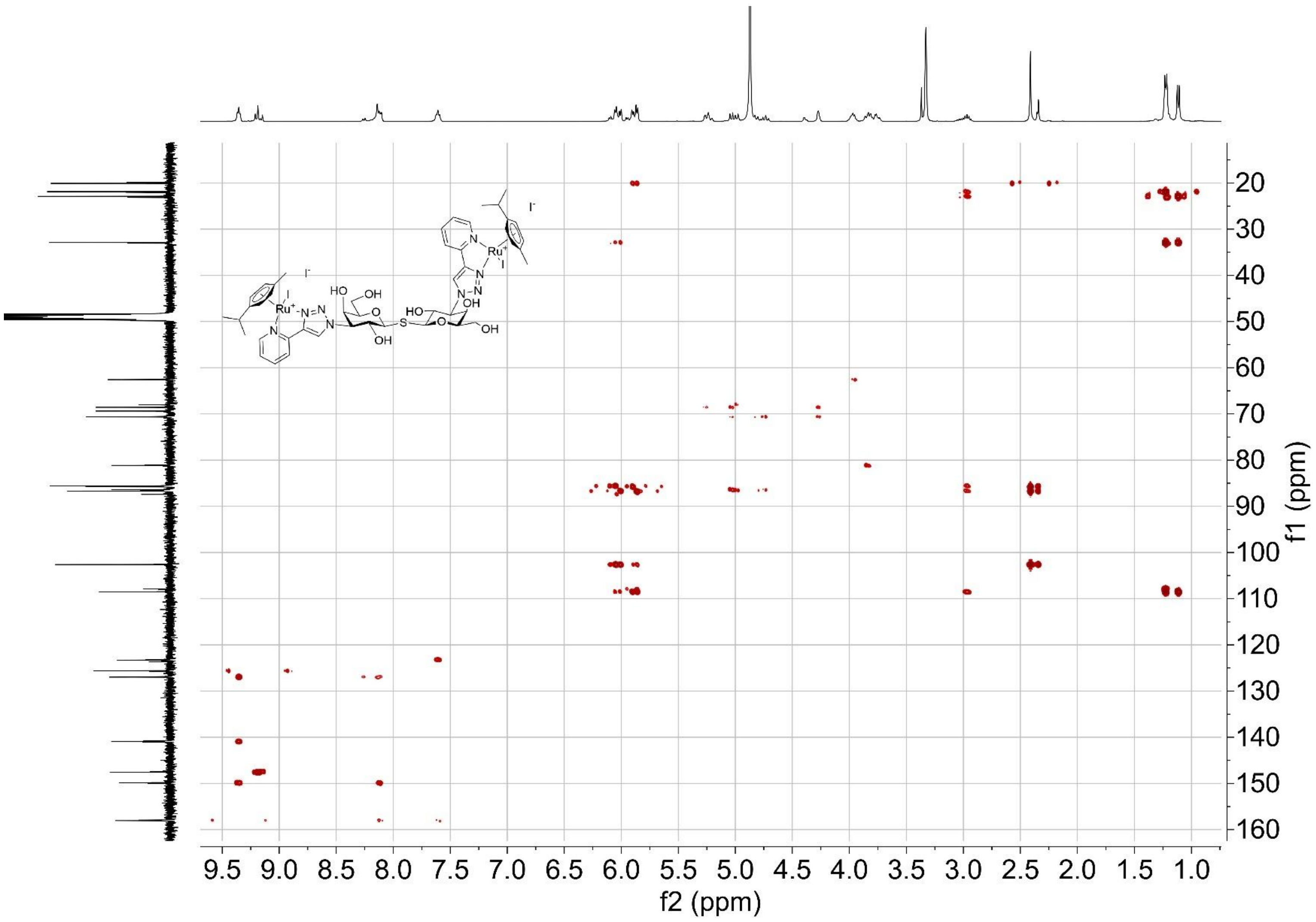

NMR COMPOUND 17-Cl

^{1}H NMR (400 MHz, DMSO-d_6) 17-Cl

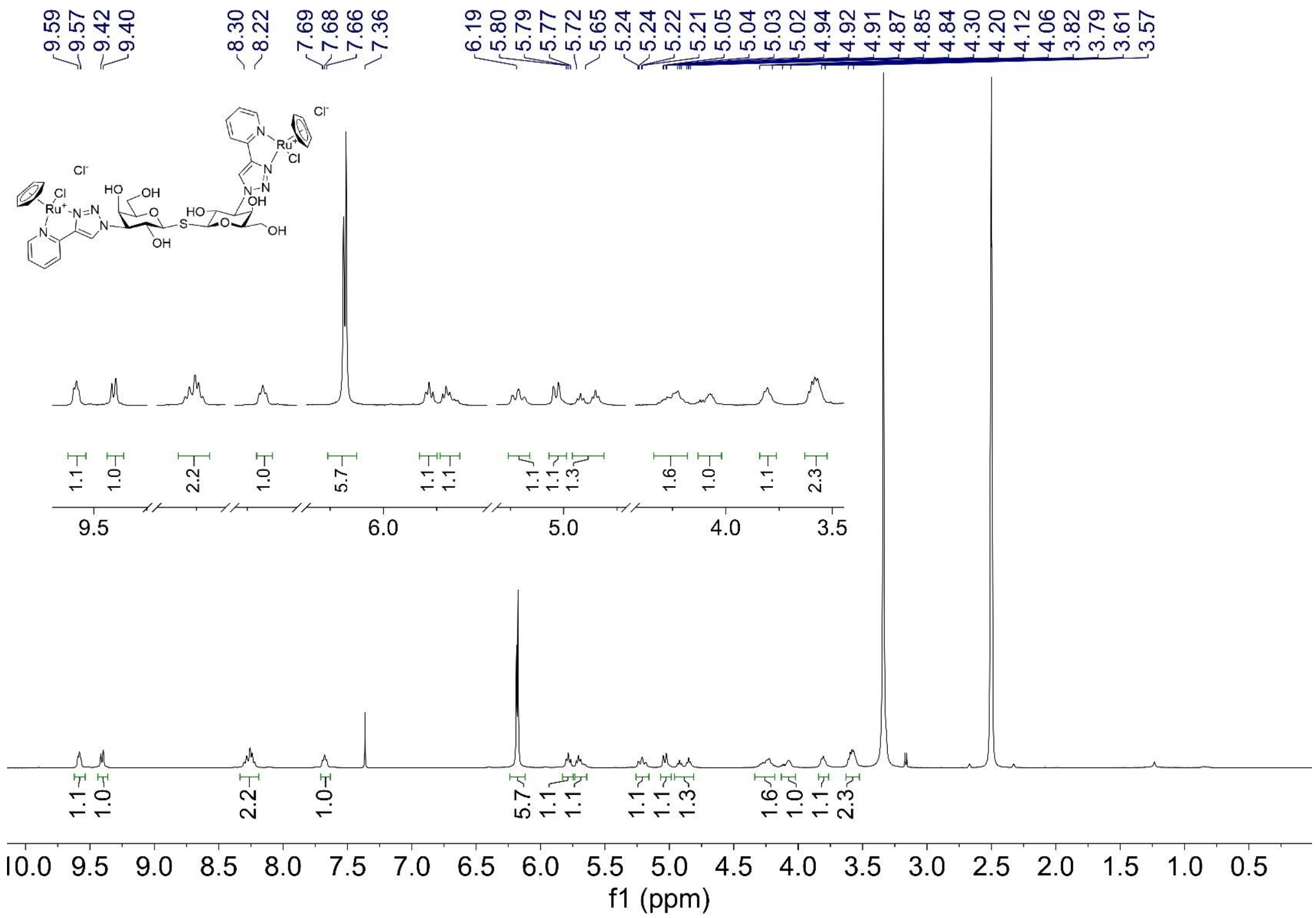

^{13}C NMR (101 MHz, DMSO-d_6) 17-Cl

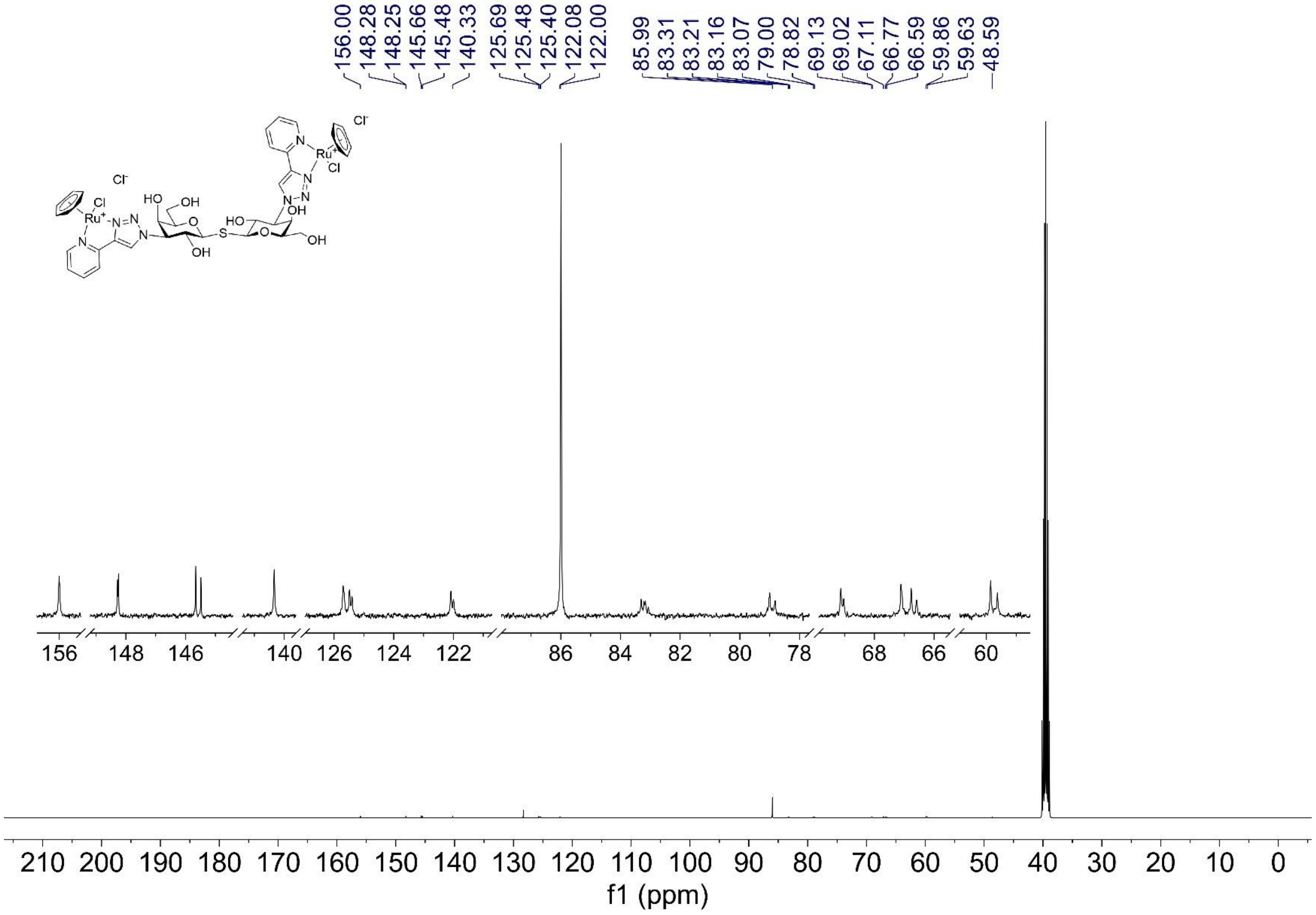

^{1}H-^{1}H COSY 17-Cl

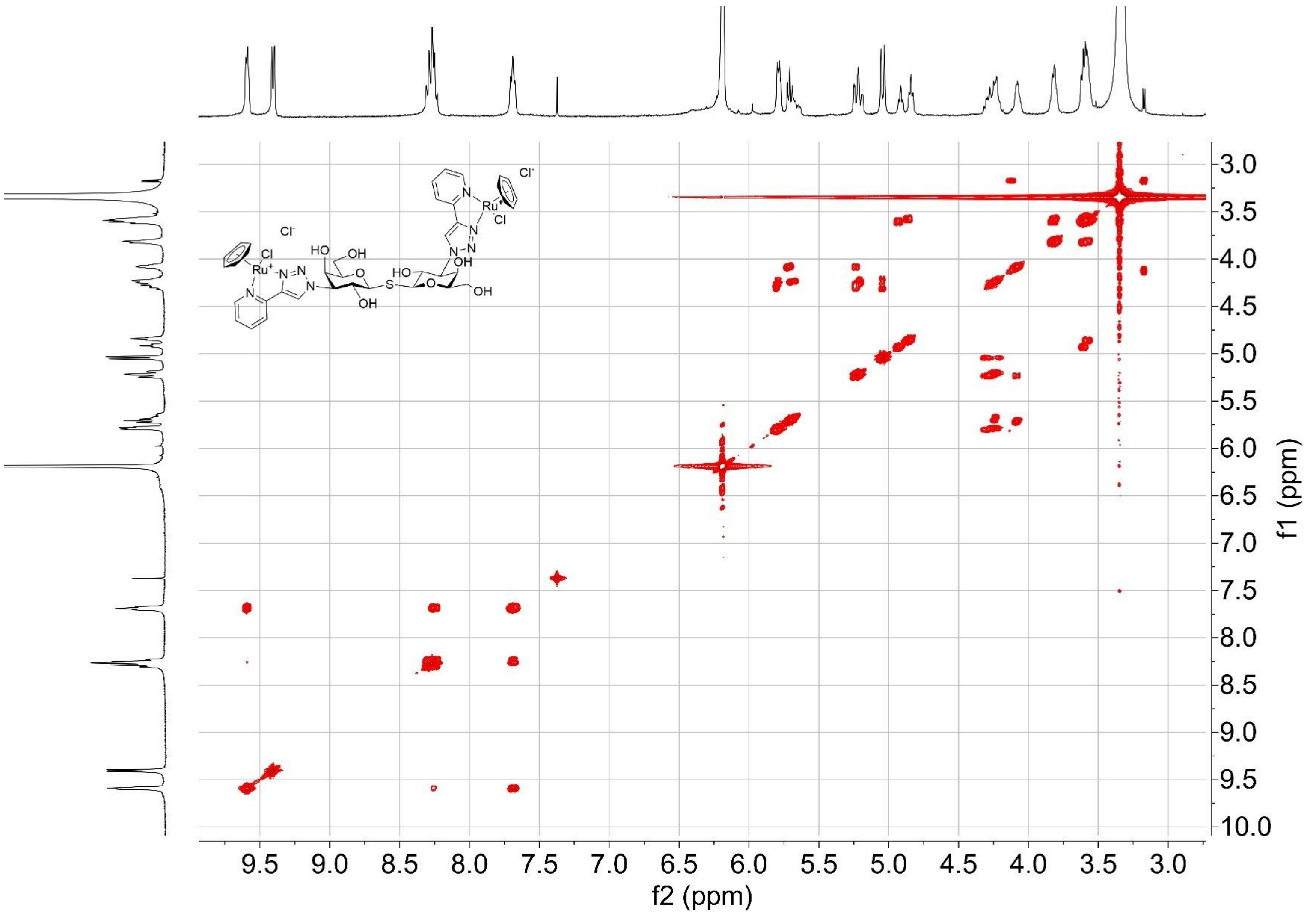

^{1}H-^{13}C HSQC 17-Cl

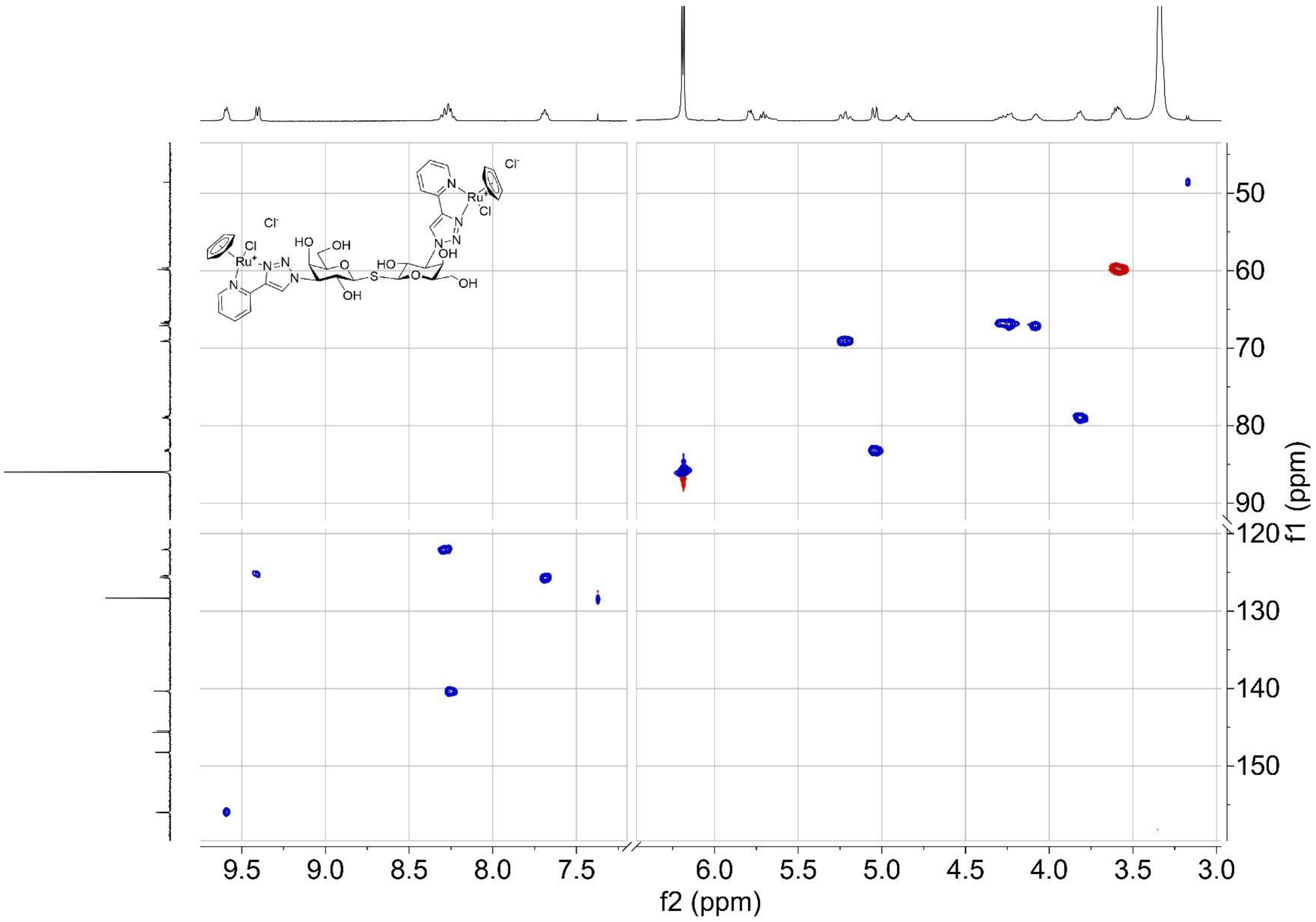

^{1}H-^{13}C HMBC 17-Cl

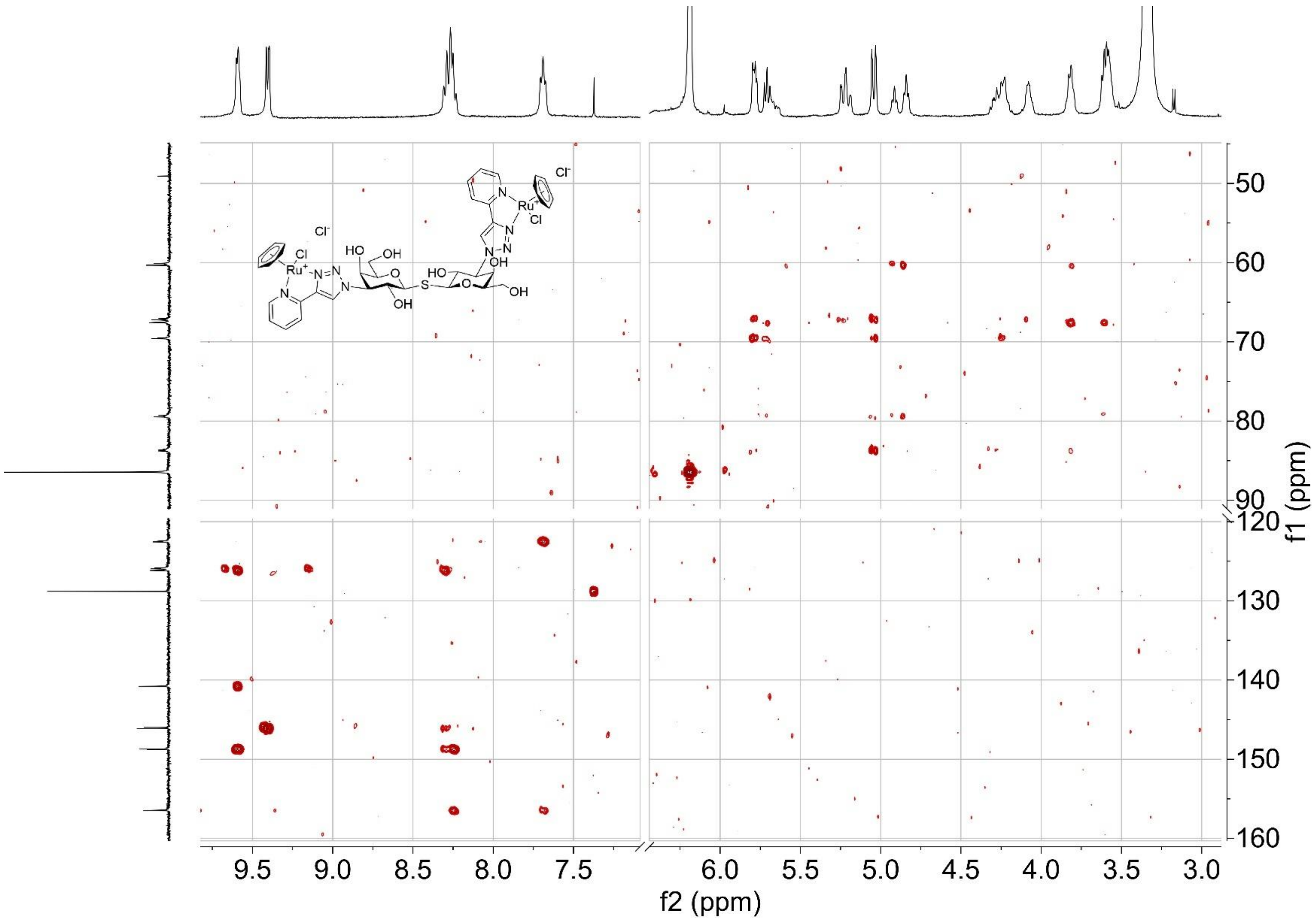

^{1}H-^{13}C HSQCTOCSY 17-Cl

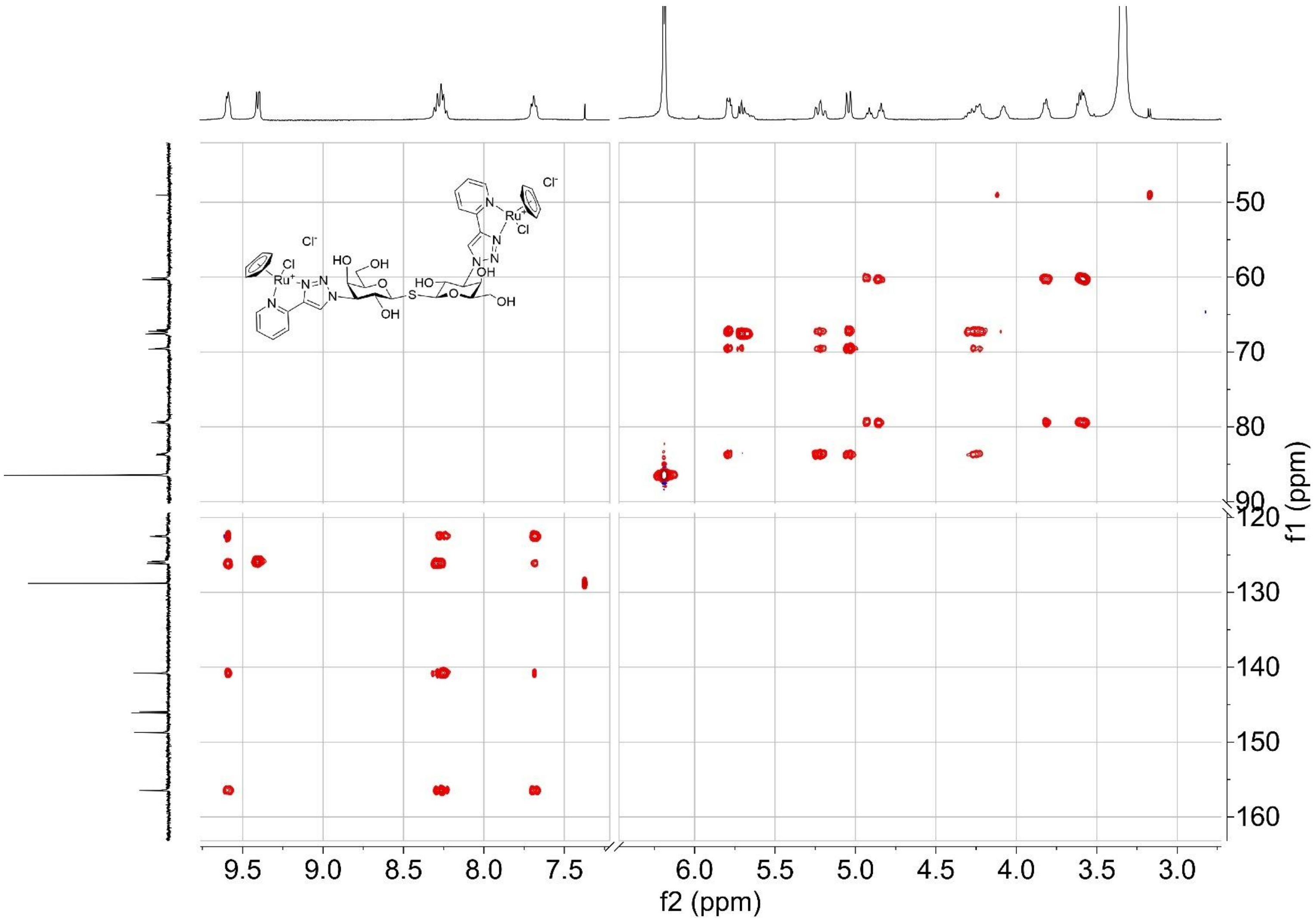

NMR COMPOUND 18-Cl

^{1}H NMR (400 MHz, DMSO-d_6) 18-Cl

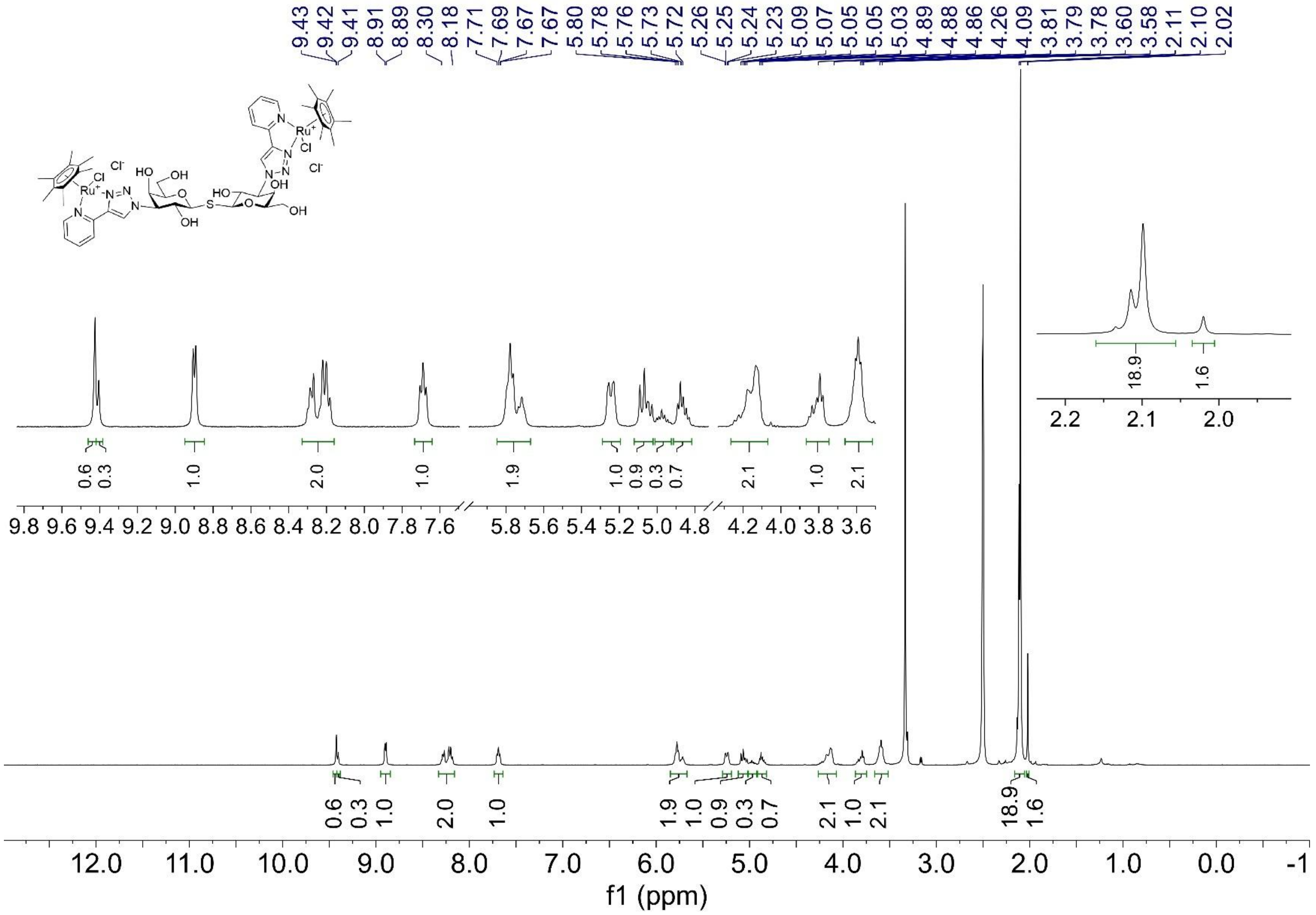

^{13}C NMR (101 MHz, DMSO-d_6) 18-Cl

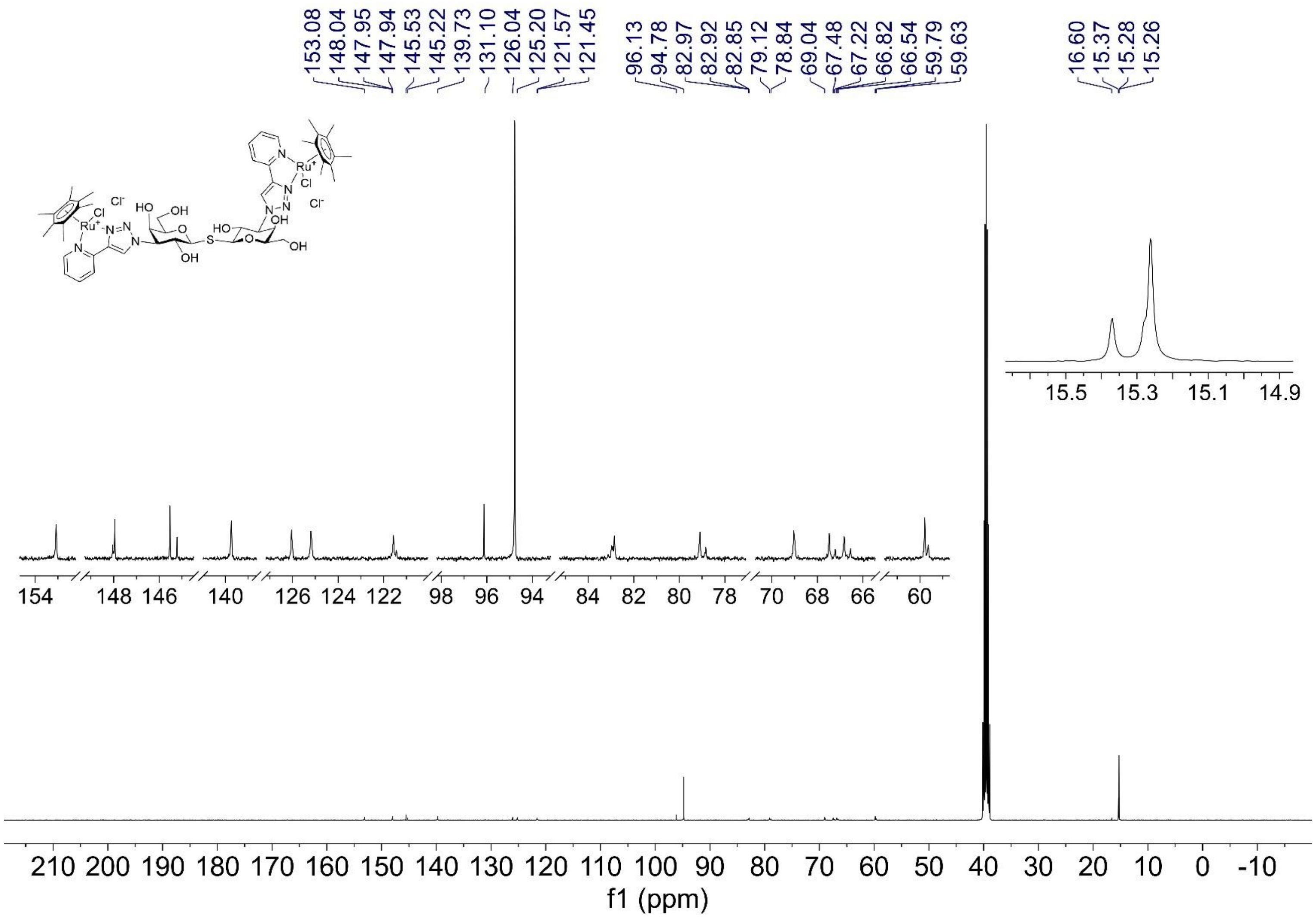

^{1}H-^{1}H COSY 18-Cl

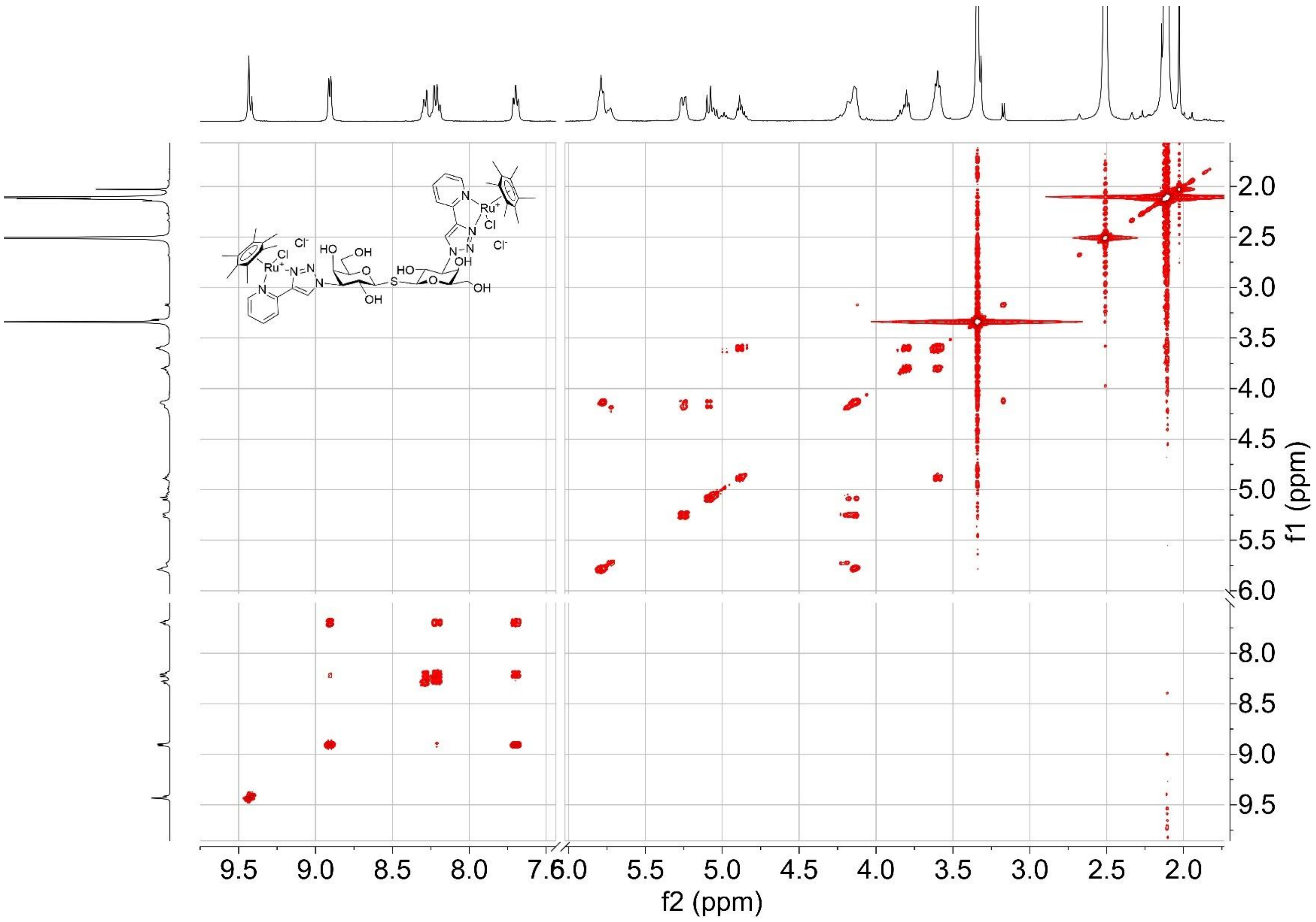

^{1}H-^{13}C HSQC 18-Cl

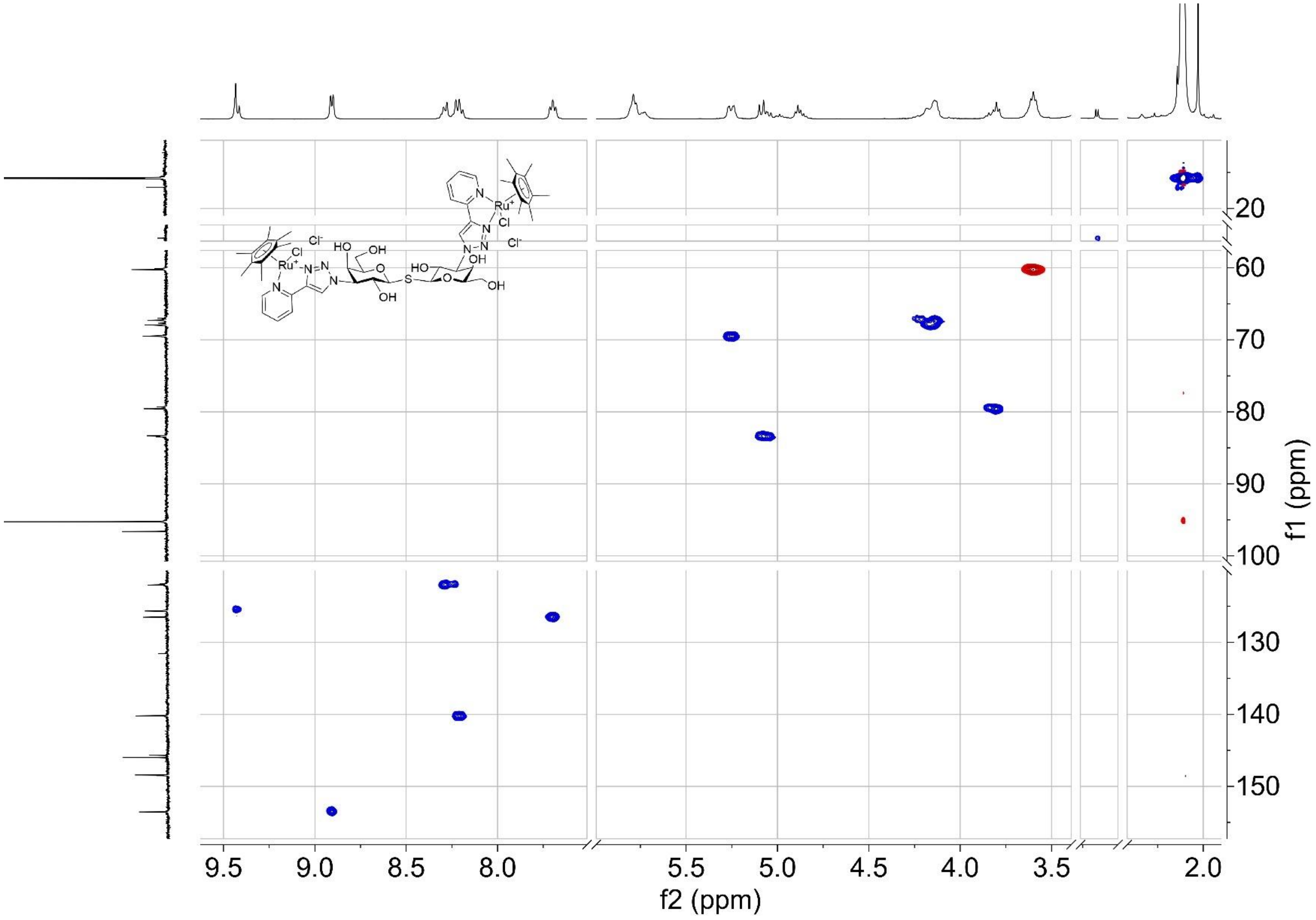

^{1}H-^{13}C HMBC 18-Cl

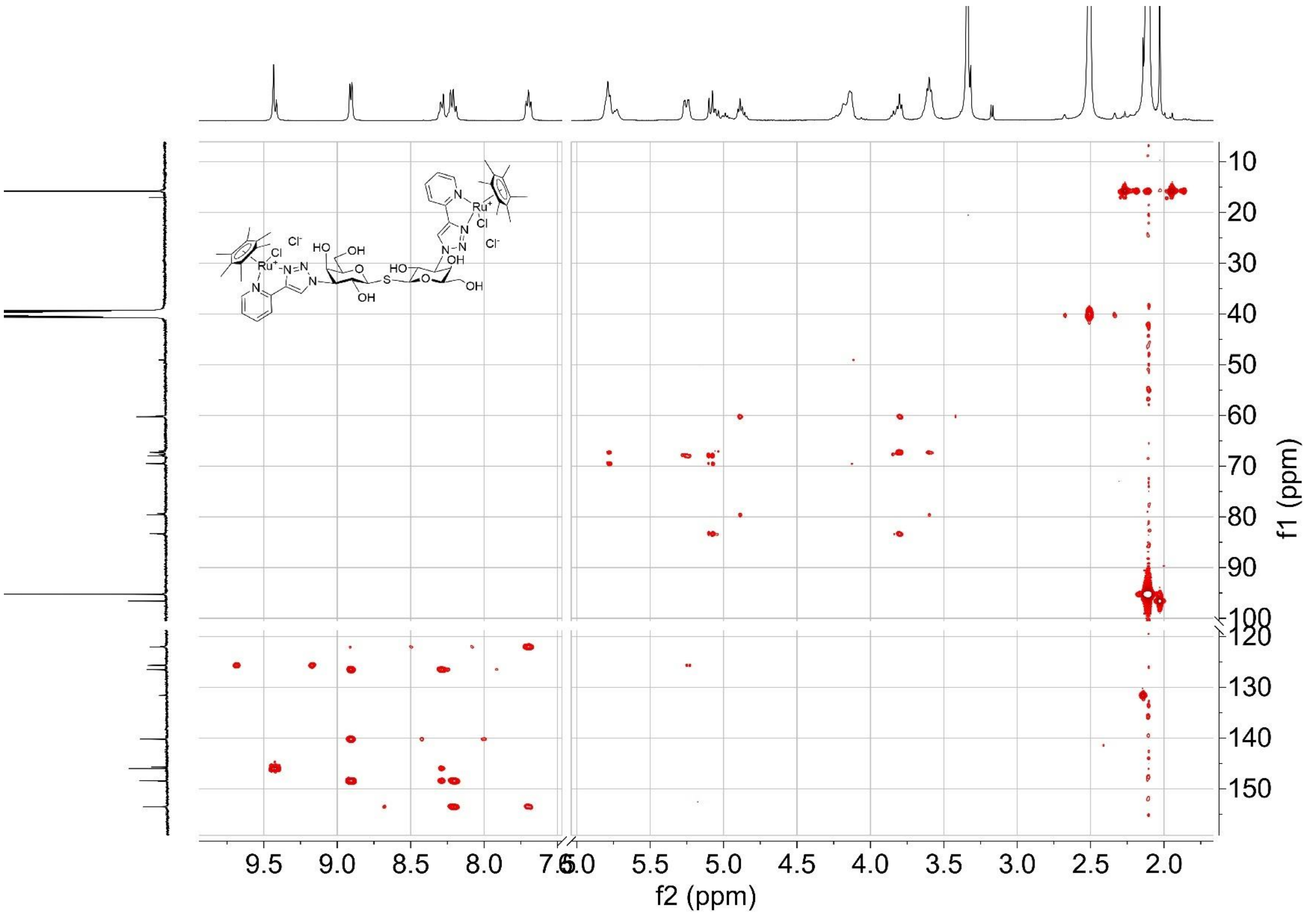

^{1}H-^{13}C HSQCTOCSY 18-Cl

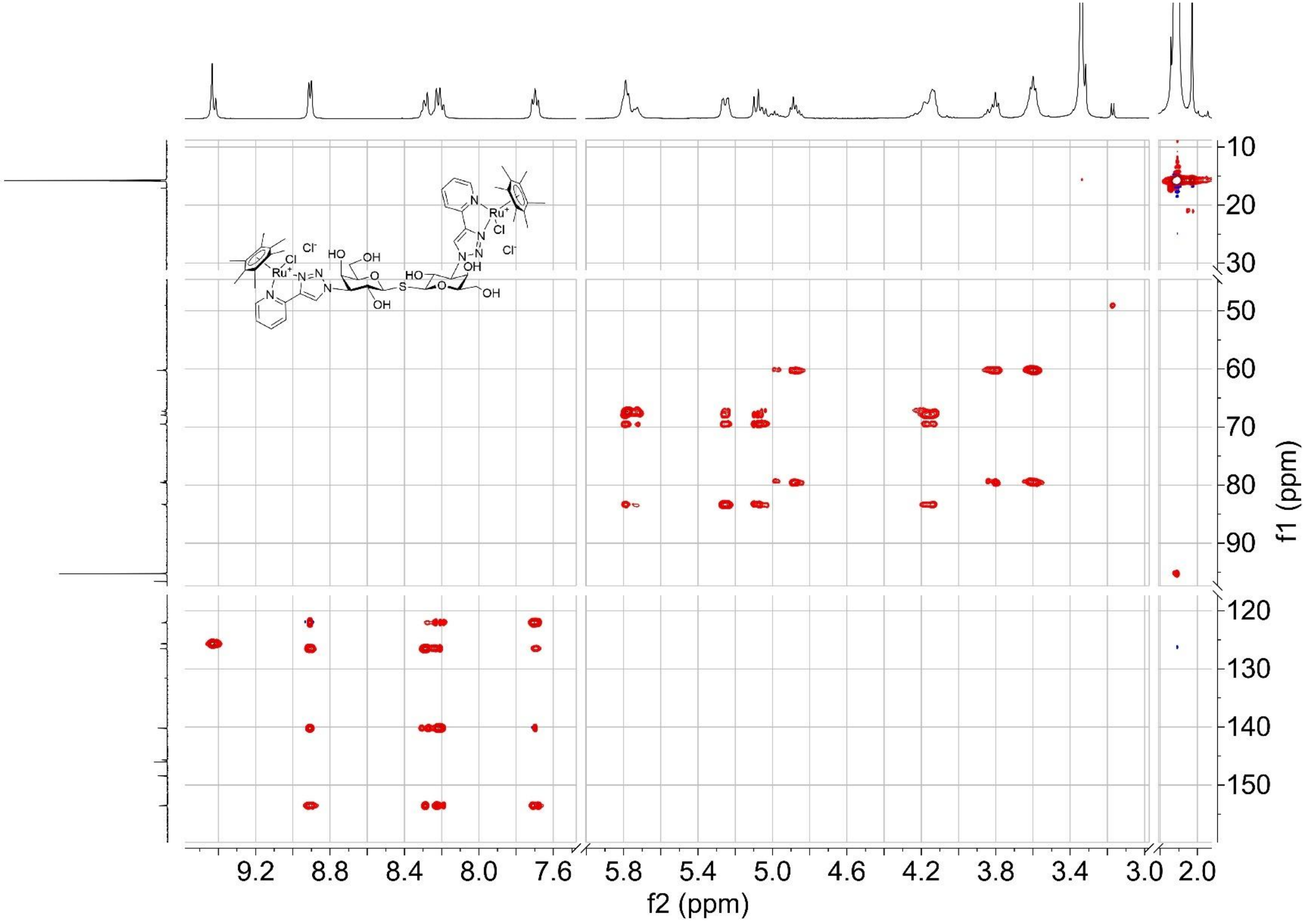

NMR COMPOUND 19

^{1}H NMR (400 MHz, CD_3OD) 19

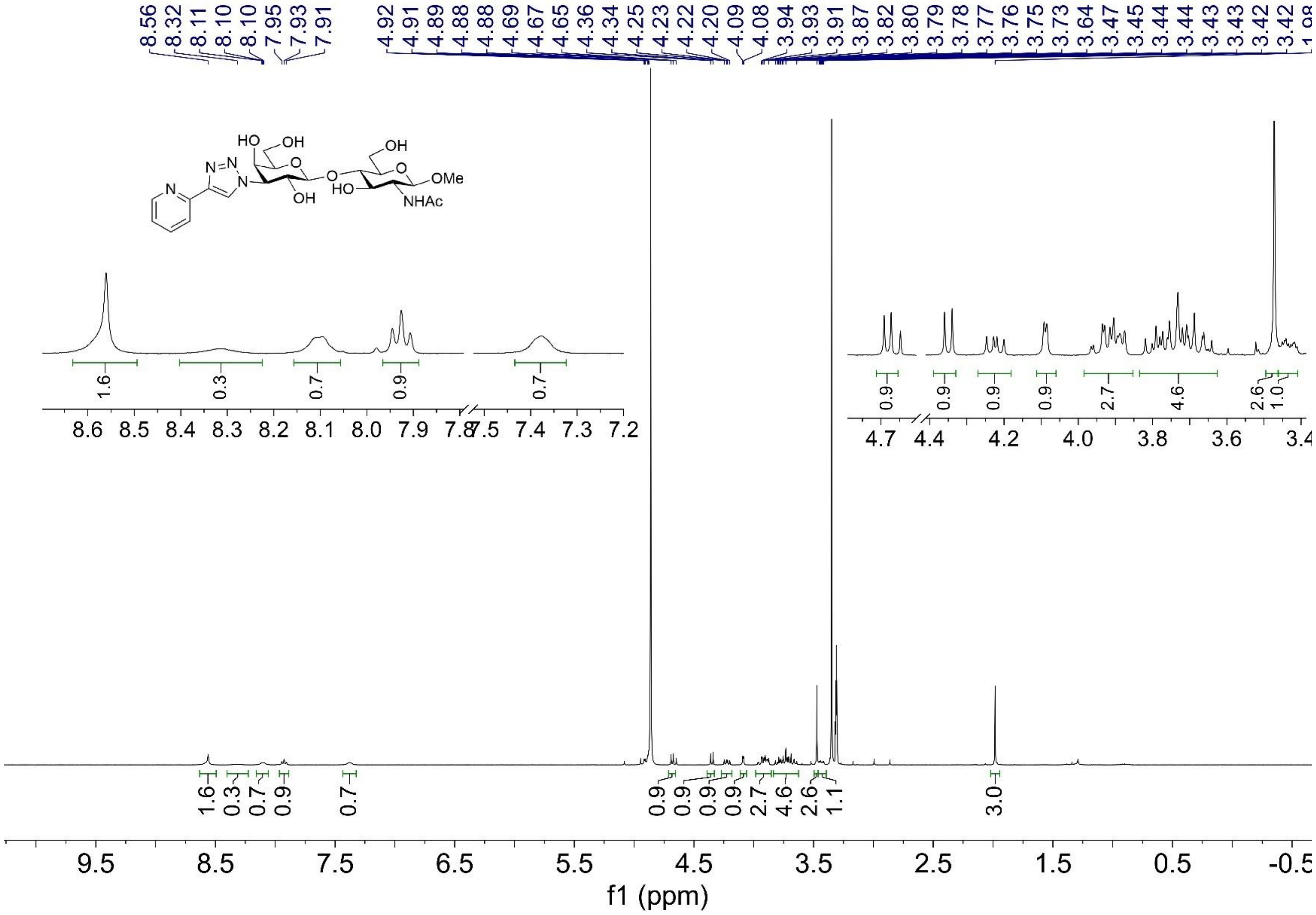

^{13}C NMR (101 MHz, CD$_3$OD) 19

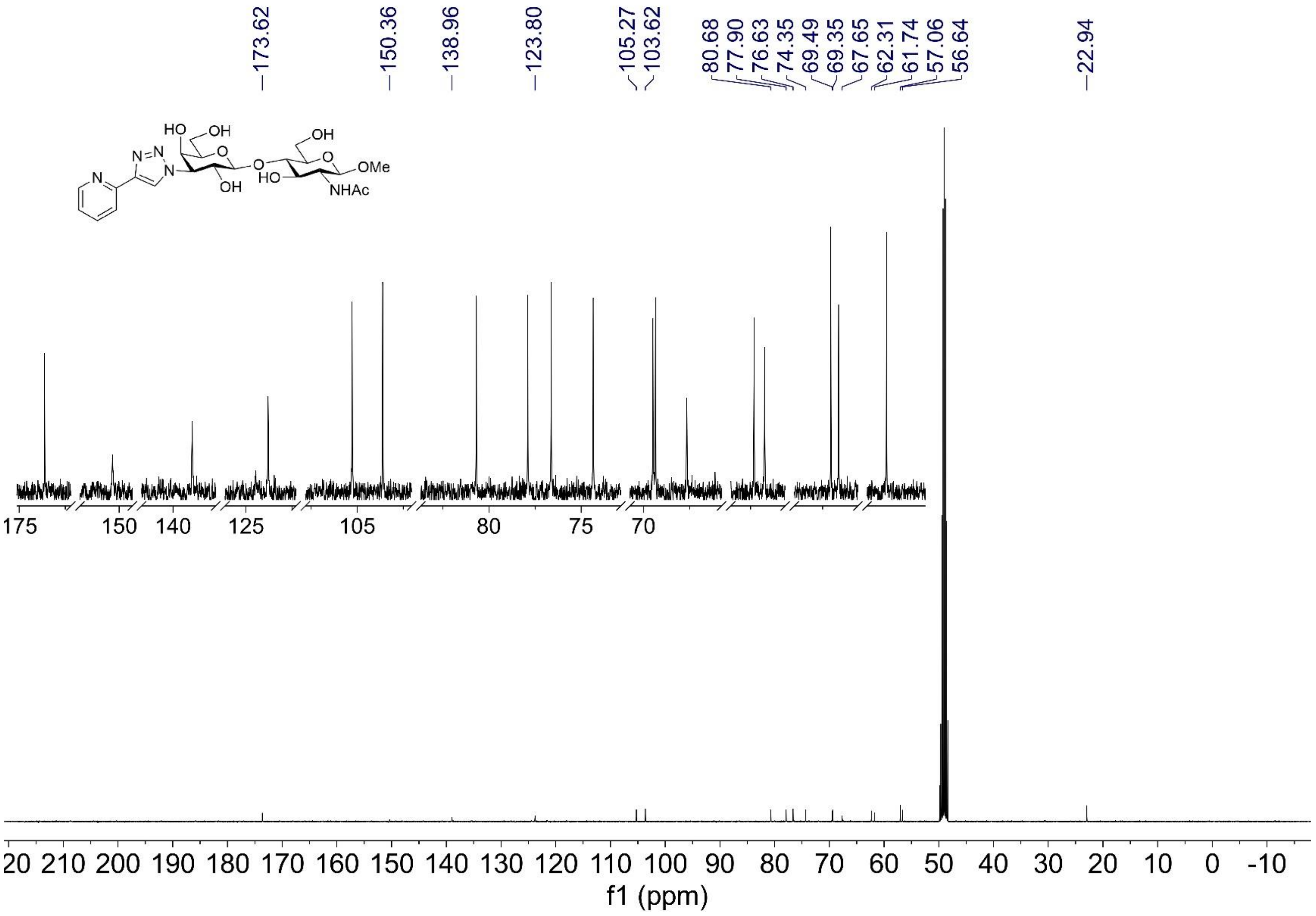

^{1}H-^{1}H COSY 19

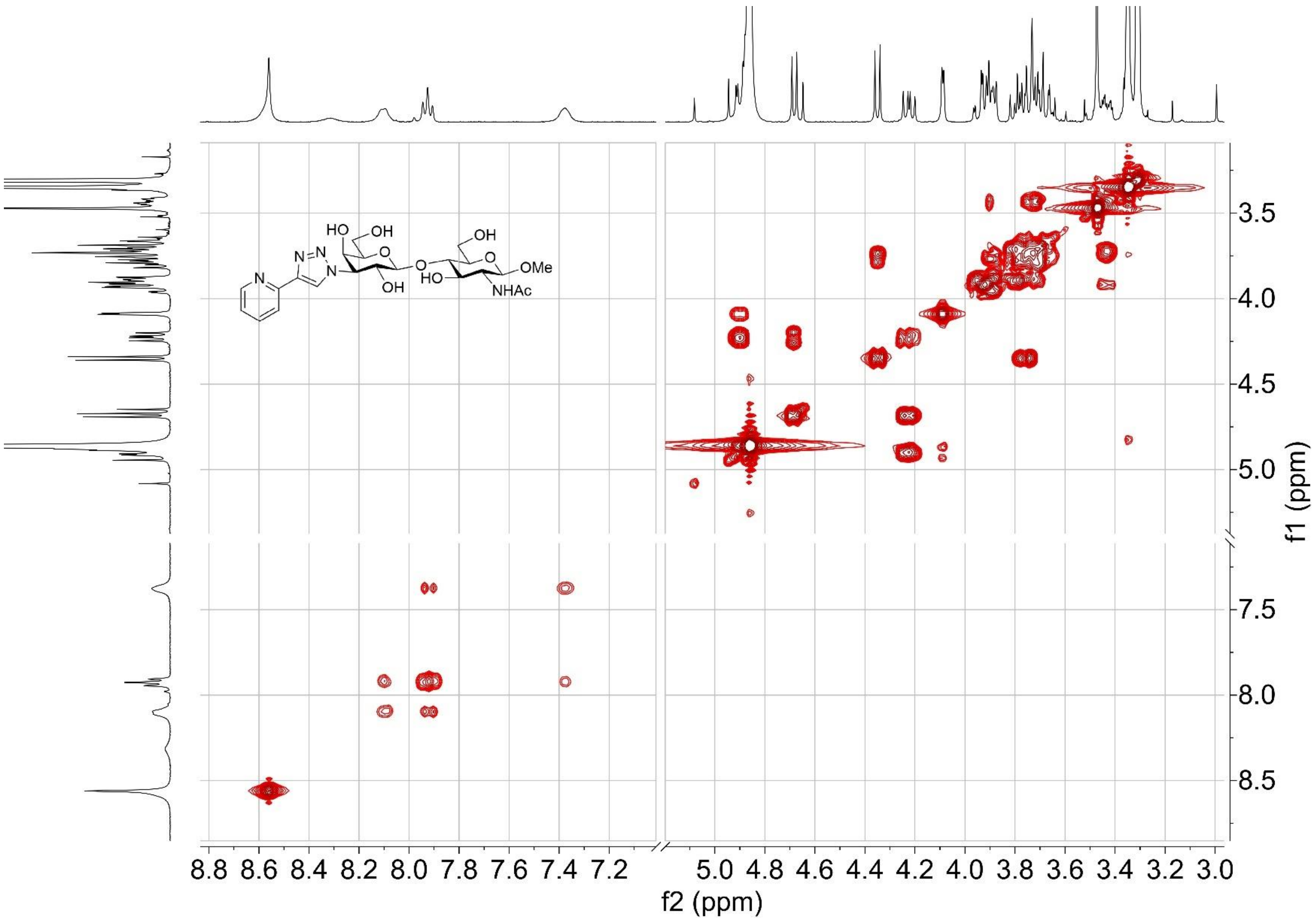

HO
OH
OH
N=N
N
N
O
O
HO
OH
OMe
HO
NHAc
OH
8.8 8.6 8.4 8.2 8.0 7.8 7.6 7.4 7.2
5.0 4.8 4.6 4.4 4.2 4.0 3.8 3.6 3.4 3.2 3.0
f2 (ppm)
3.5
4.0
4.5
5.0
7.5
8.0
8.5
f1 (ppm)

^{1}H-^{13}C HSQC 19

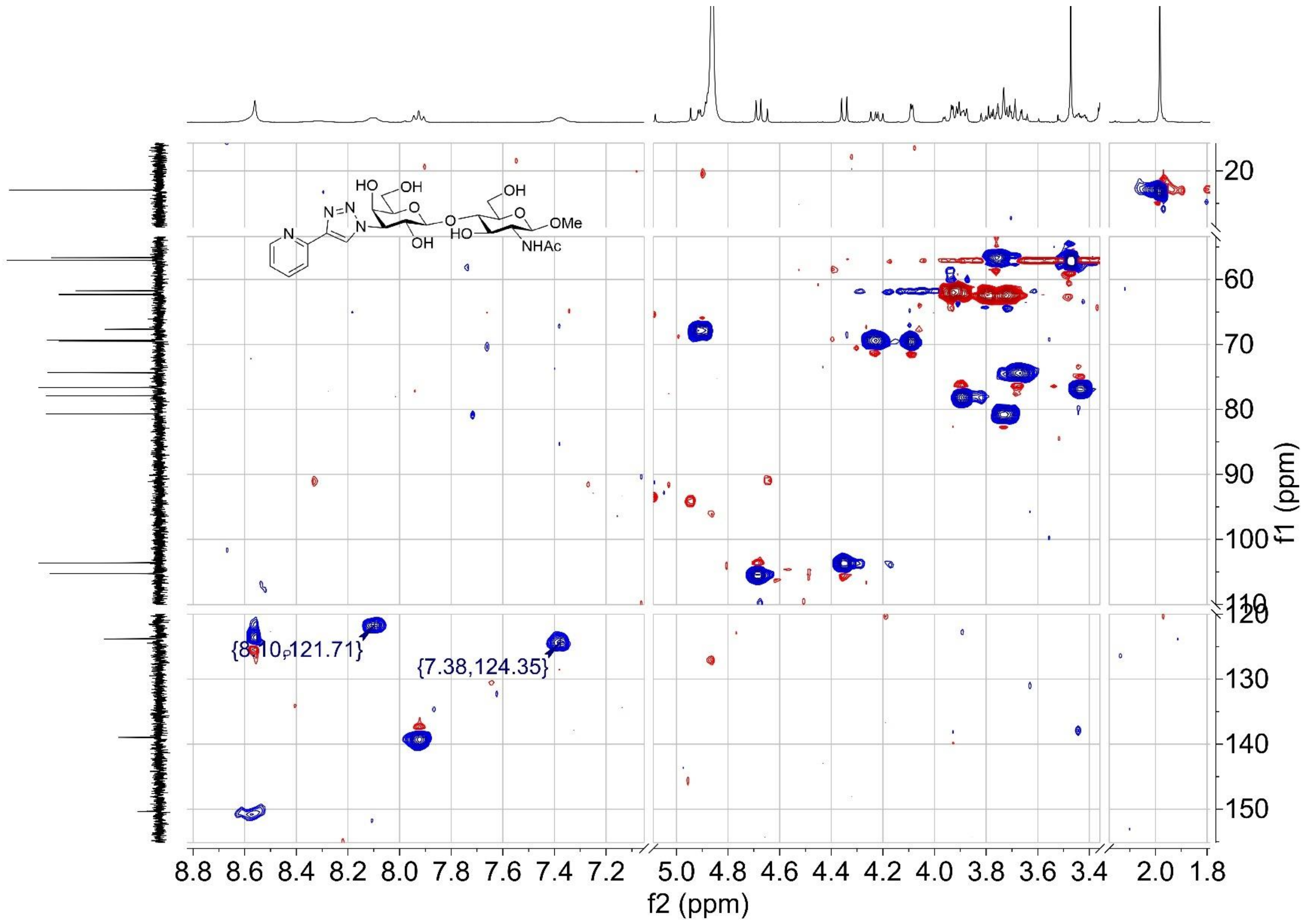

^{1}H-^{13}C HMBC 19

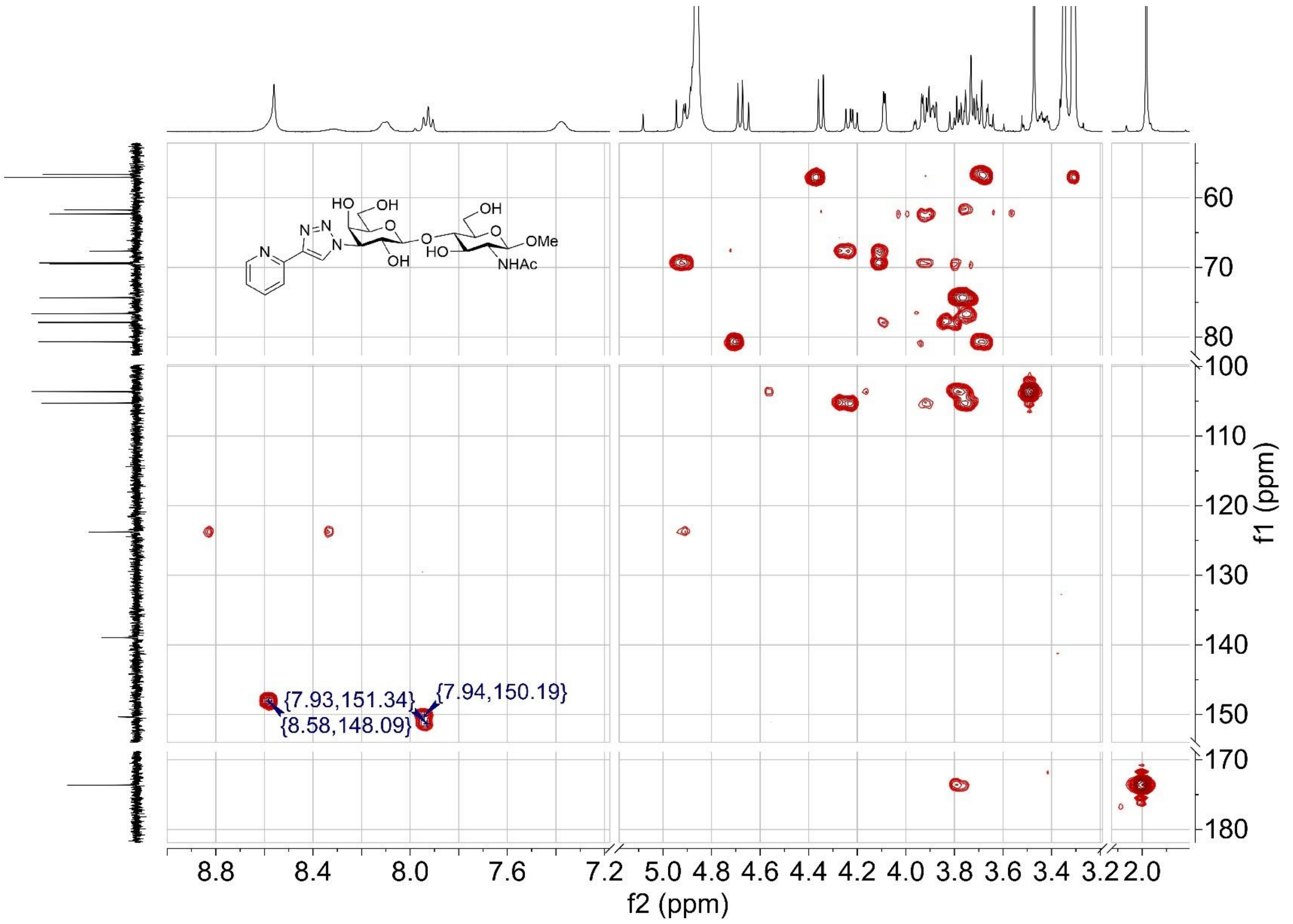

NMR COMPOUND 20

^{1}H NMR (400 MHz, DMSO-d_6) 20

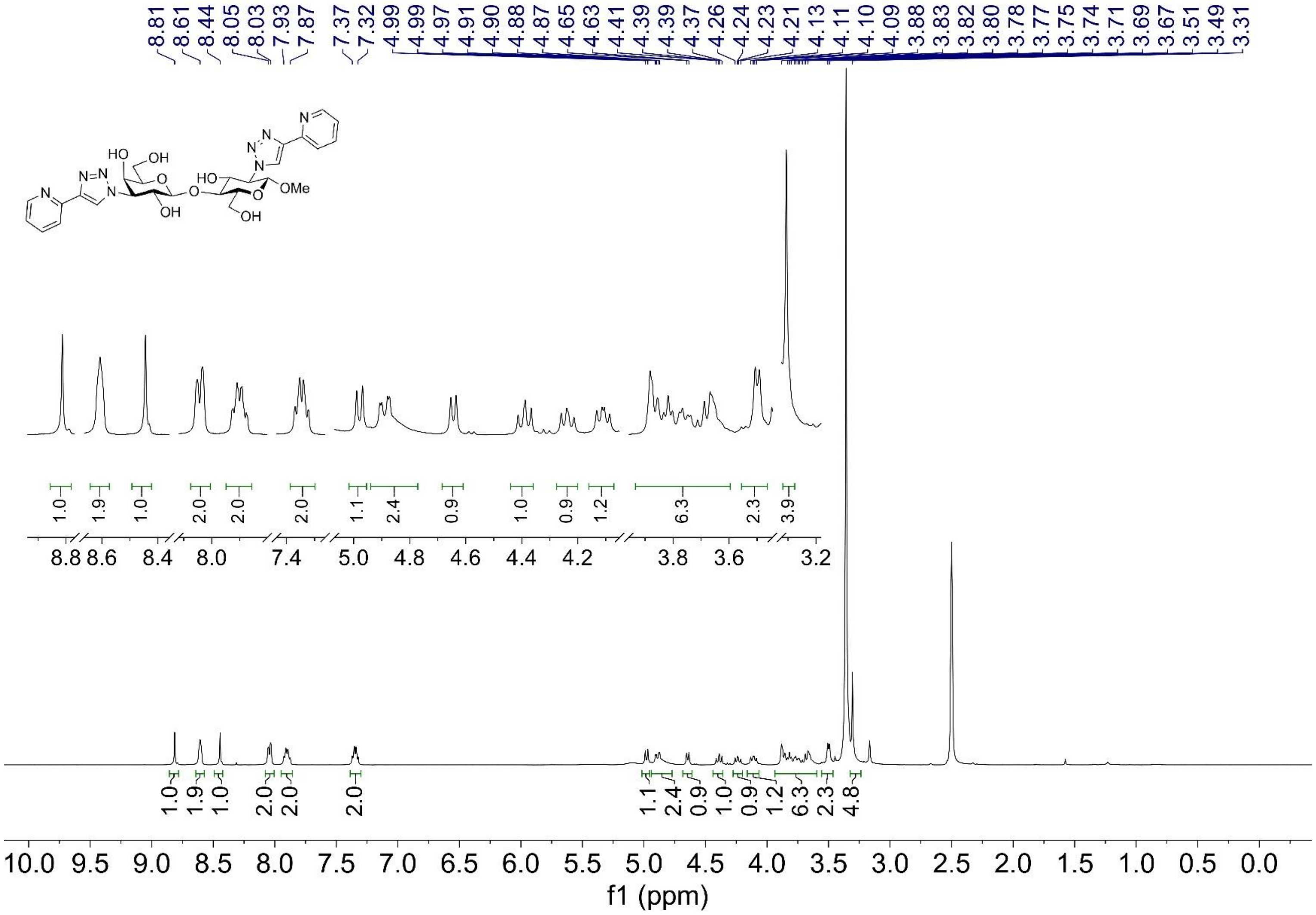

^{13}C NMR (101 MHz, DMSO-d_6) **20**

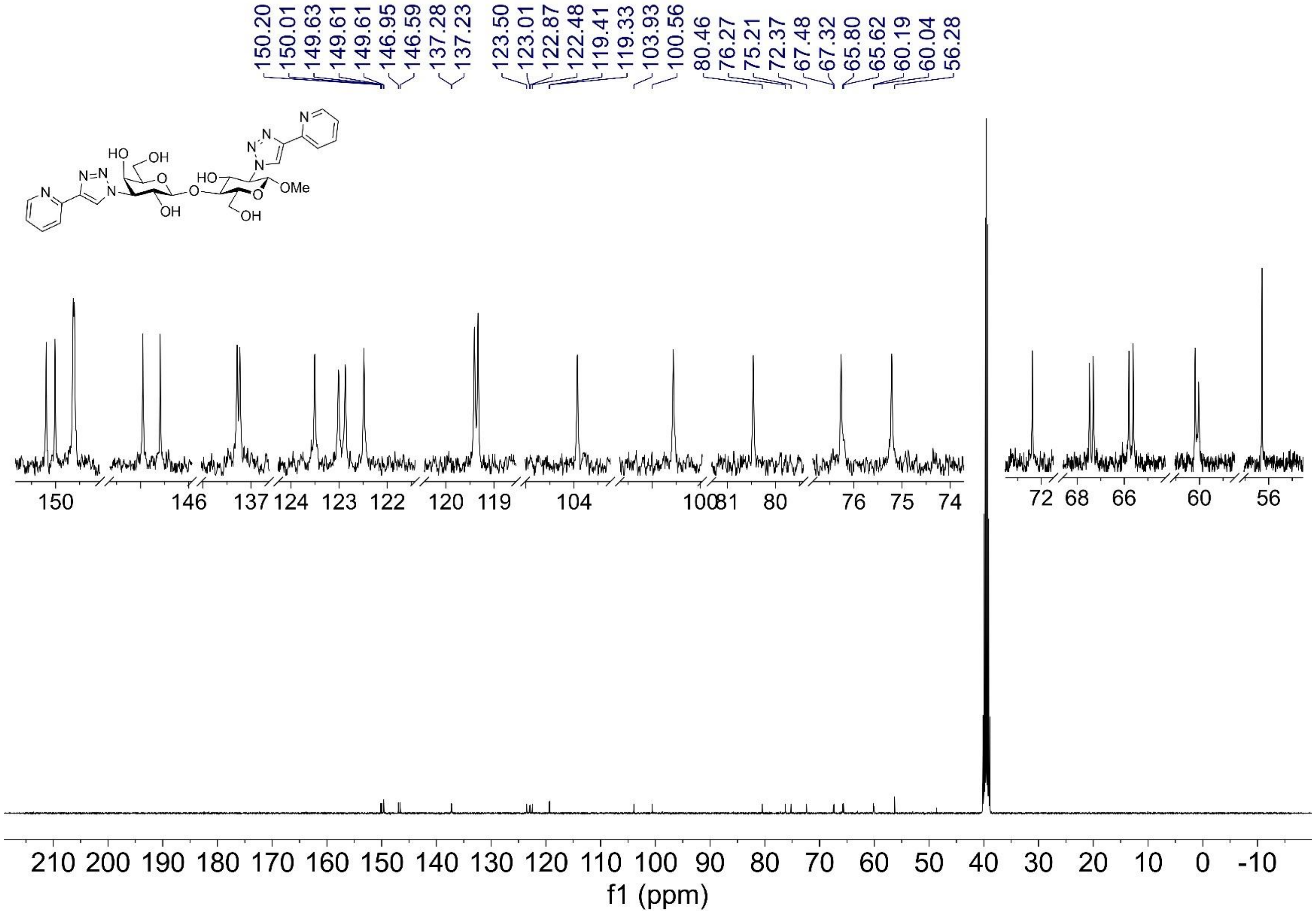

^{1}H-^{1}H COSY 20

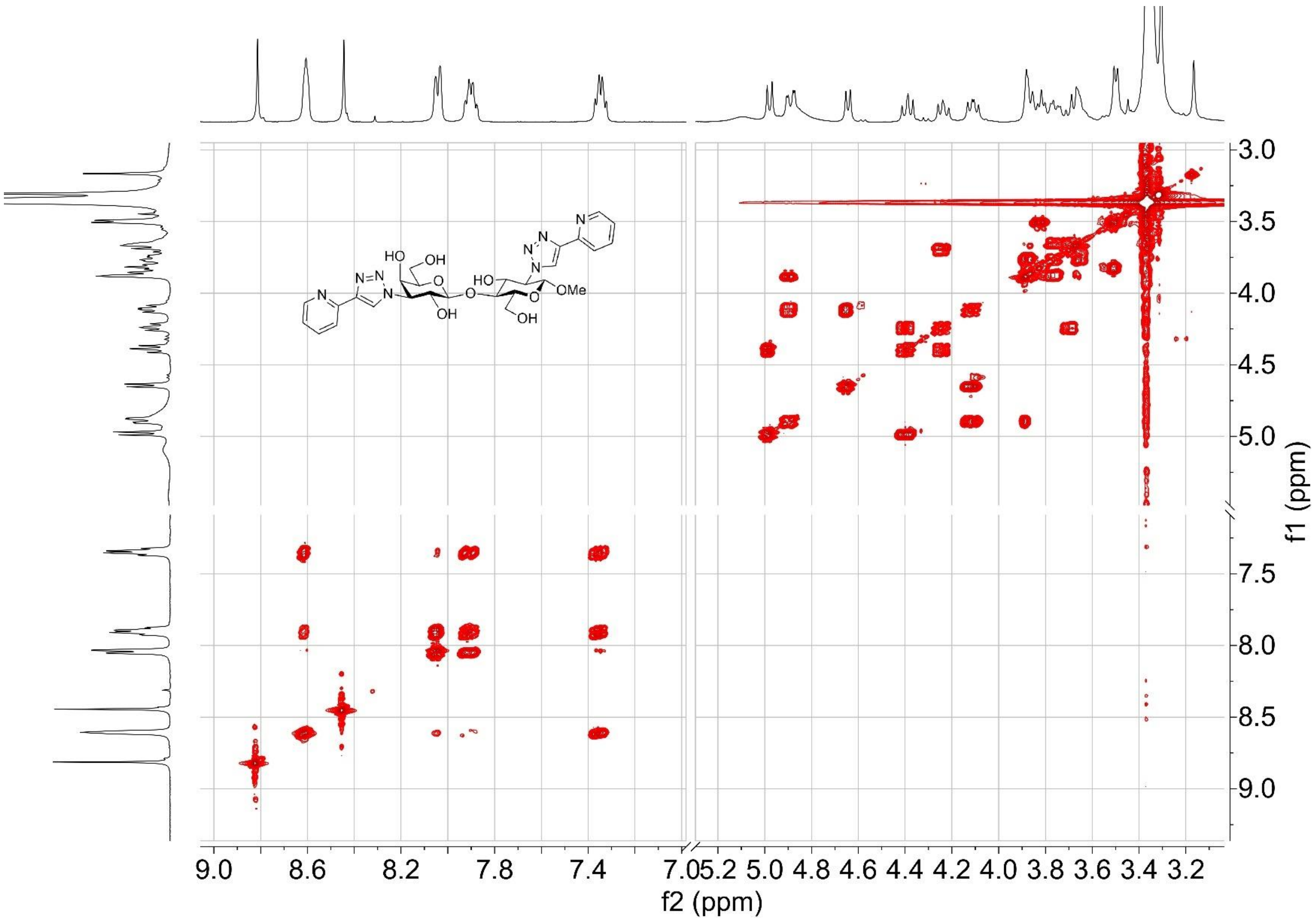

^1H-^{13}C HSQC 20

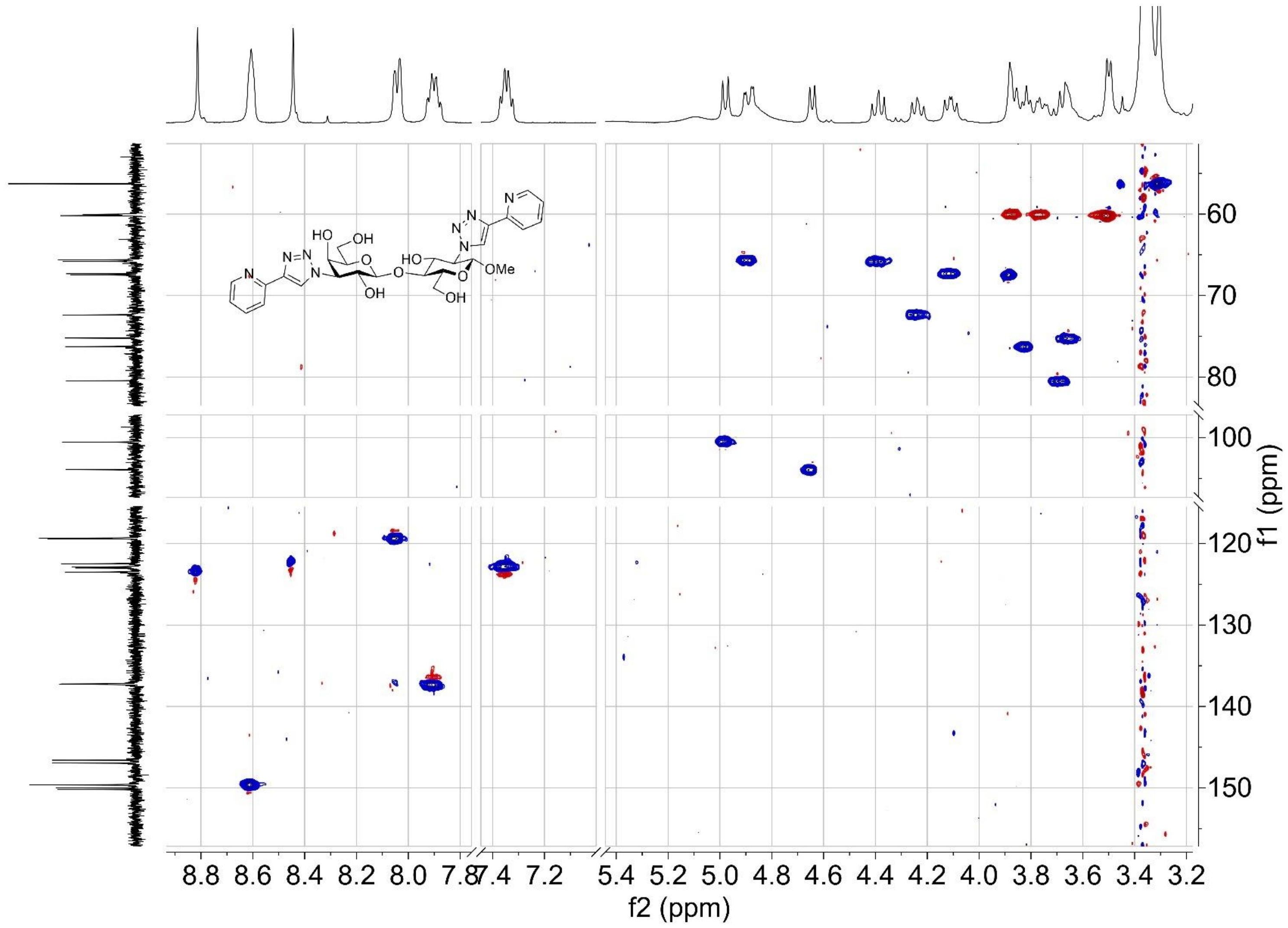

^{1}H-^{13}C HMBC 20

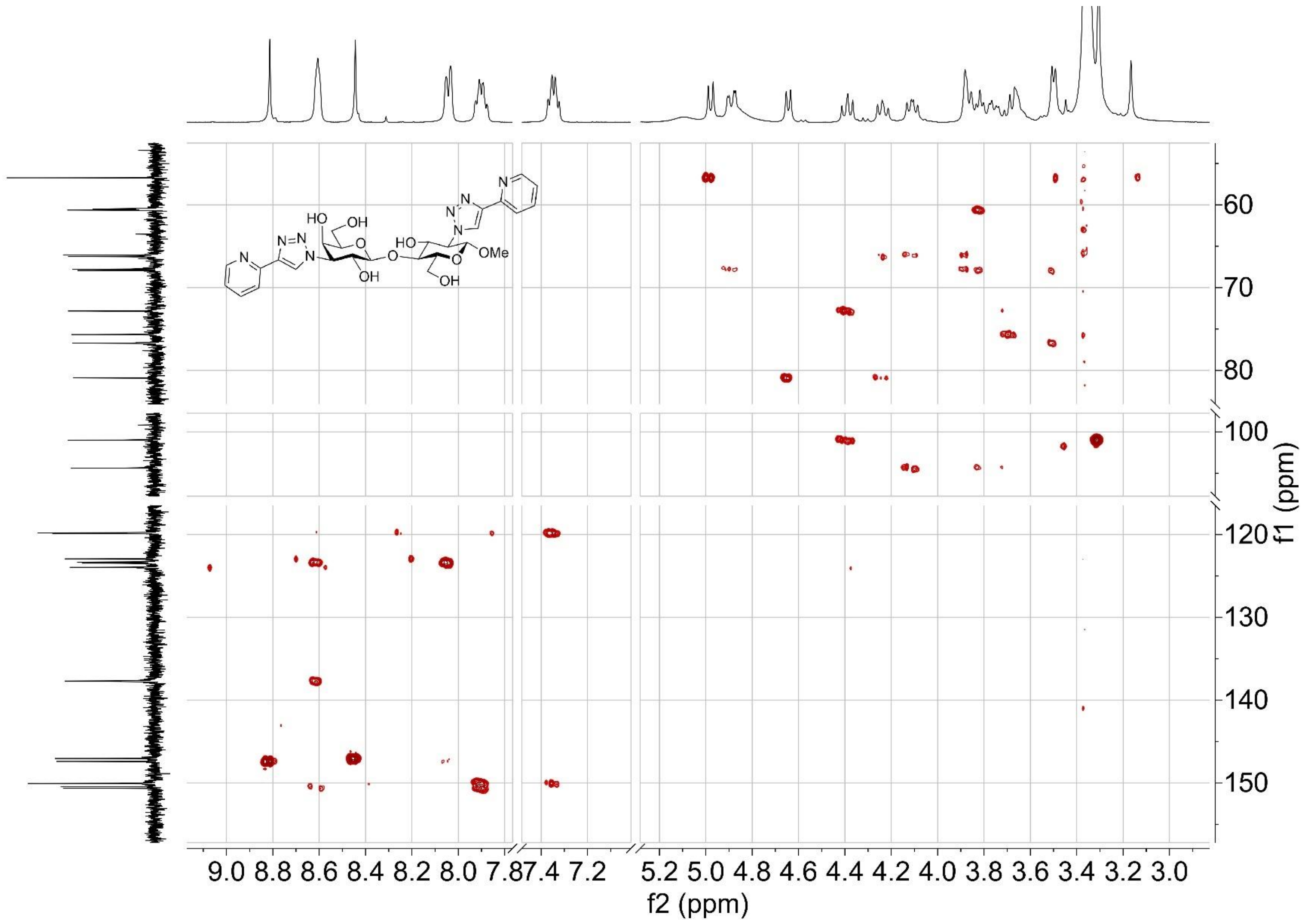

NMR COMPOUND 21

^{1}H NMR (400 MHz, DMSO-d_6) 21

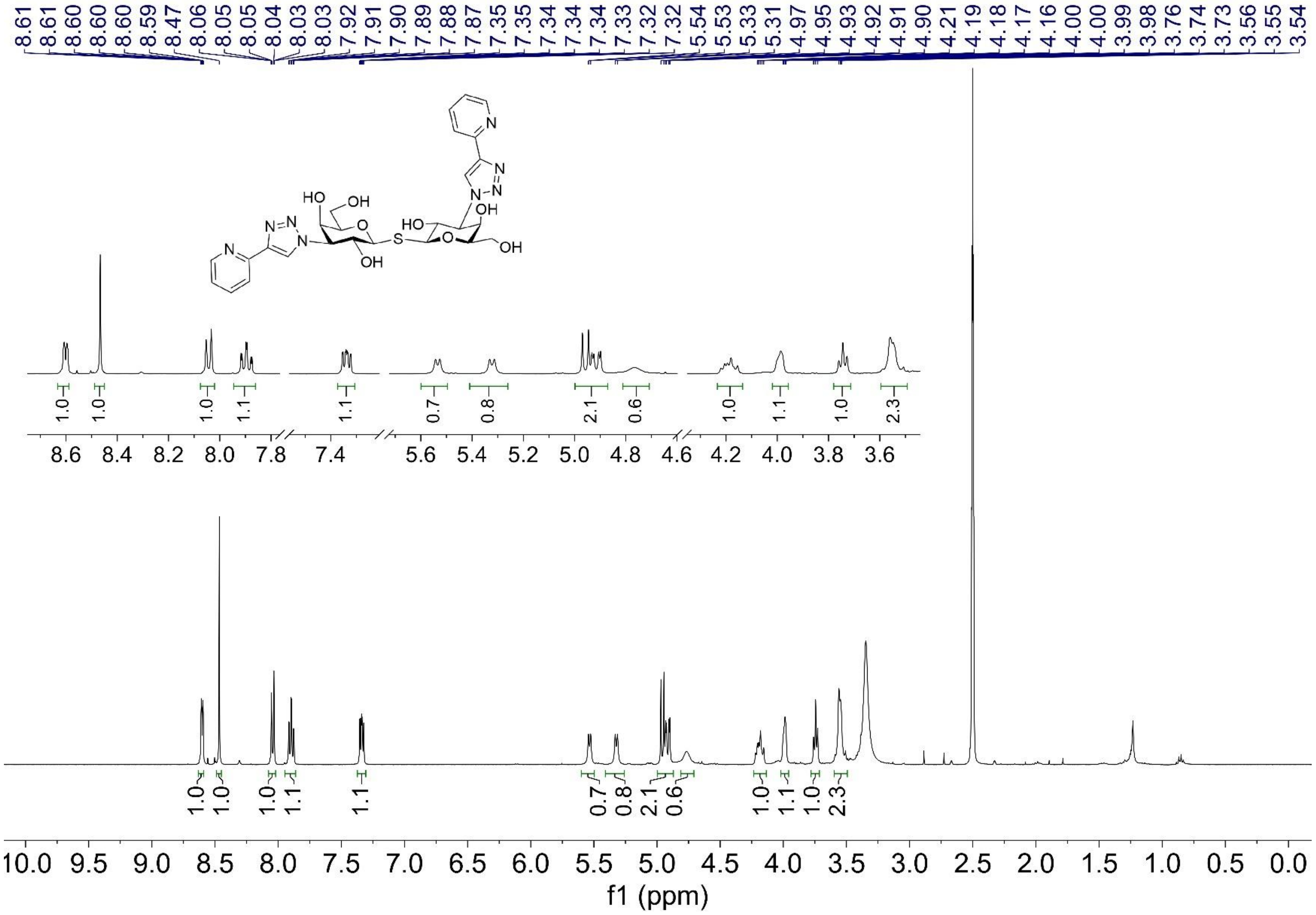

^{13}C NMR (101 MHz, DMSO-d_6) 21

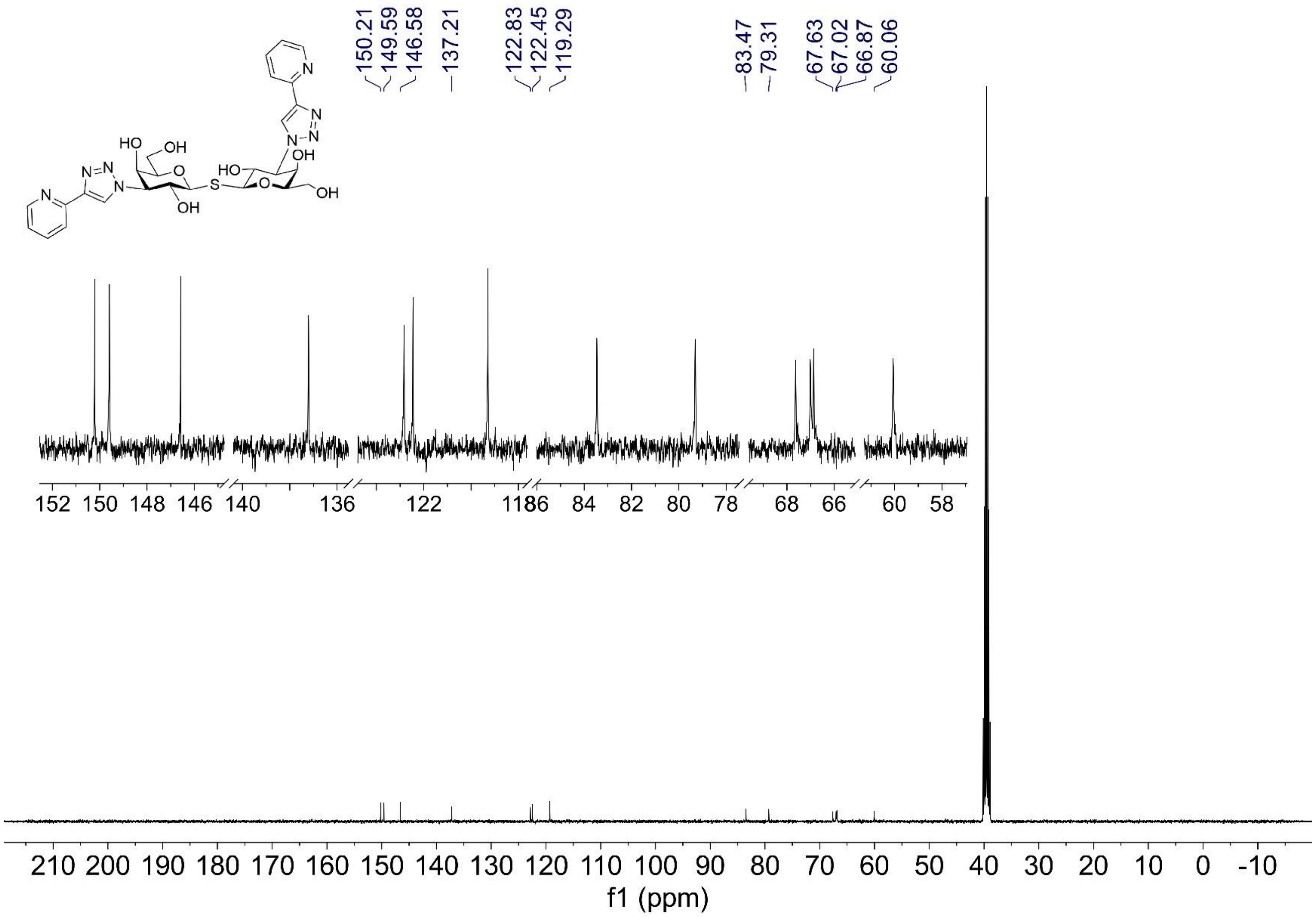

^{1}H-^{1}H COSY 21

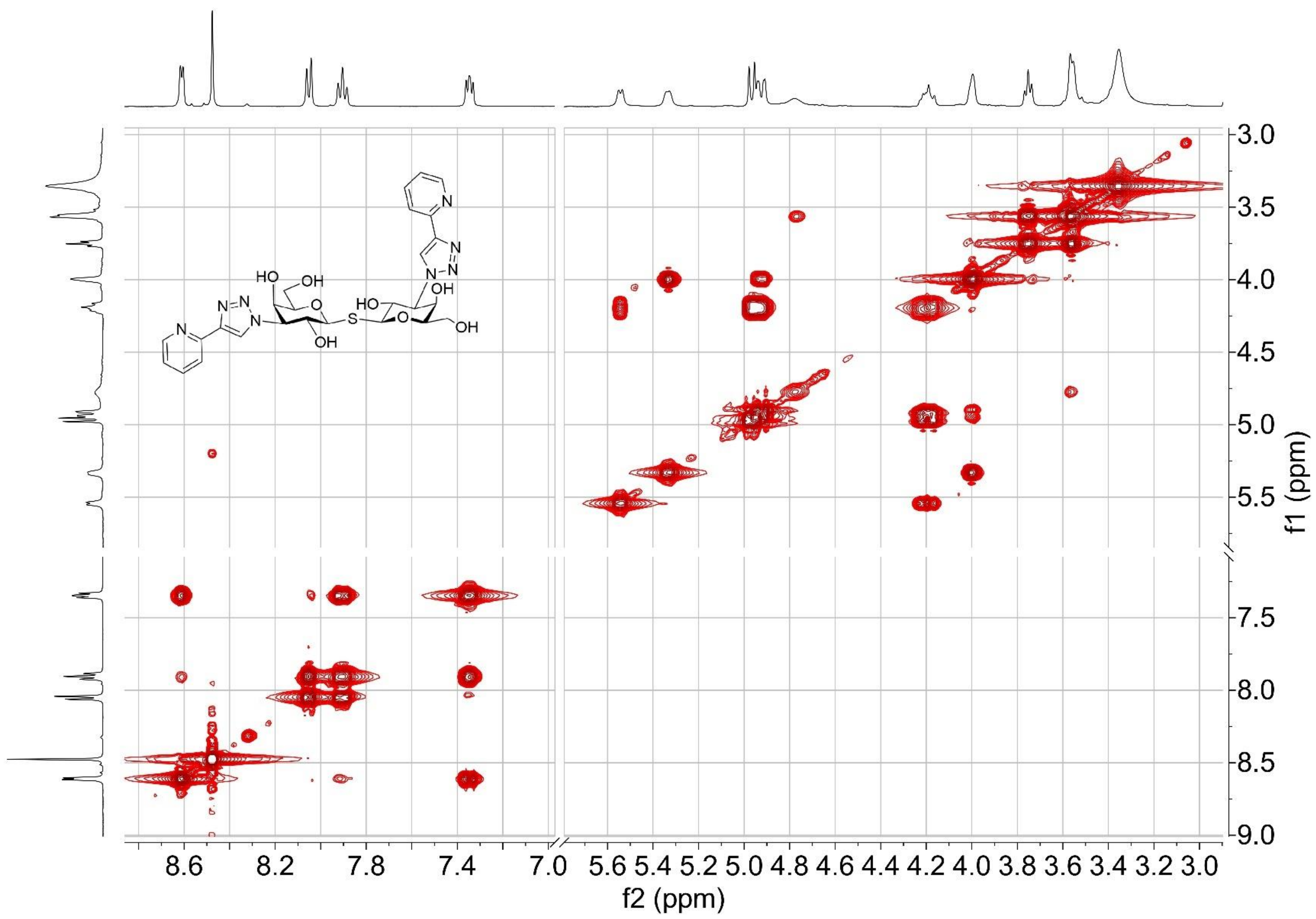

N
N
N
N
HO
OH
OH
N
N
N
N
O
S
HO
O
OH
OH
OH
N
8.6
8.2
7.8
7.4
7.0
5.6
5.4
5.2
5.0
4.8
4.6
4.4
4.2
4.0
3.8
3.6
3.4
3.2
3.0
f2 (ppm)
3.0
3.5
4.0
4.5
5.0
5.5
7.5
8.0
8.5
9.0
f1 (ppm)

^{1}H-^{13}C HSQC 21

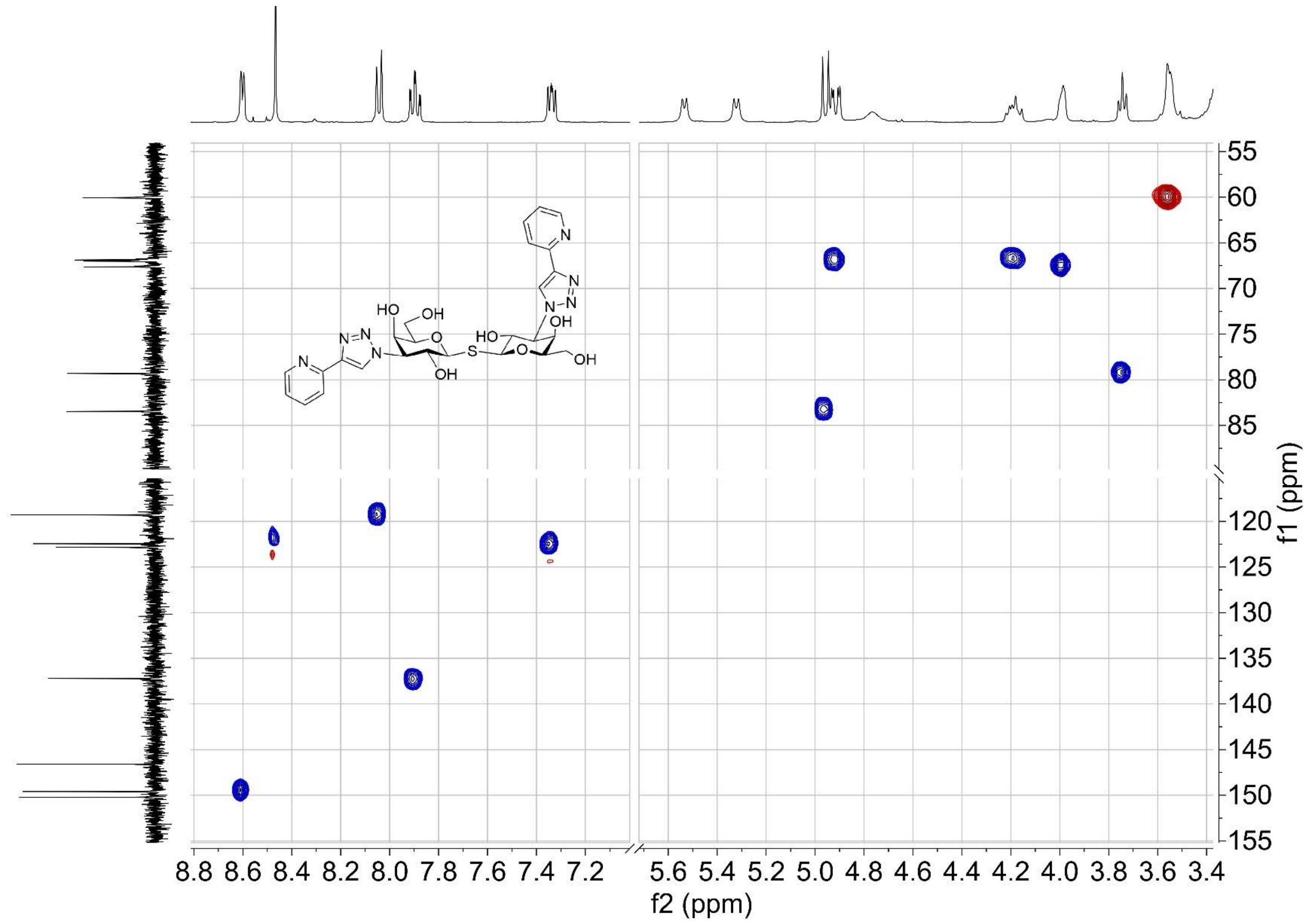

^{1}H-^{13}C HMBC 21

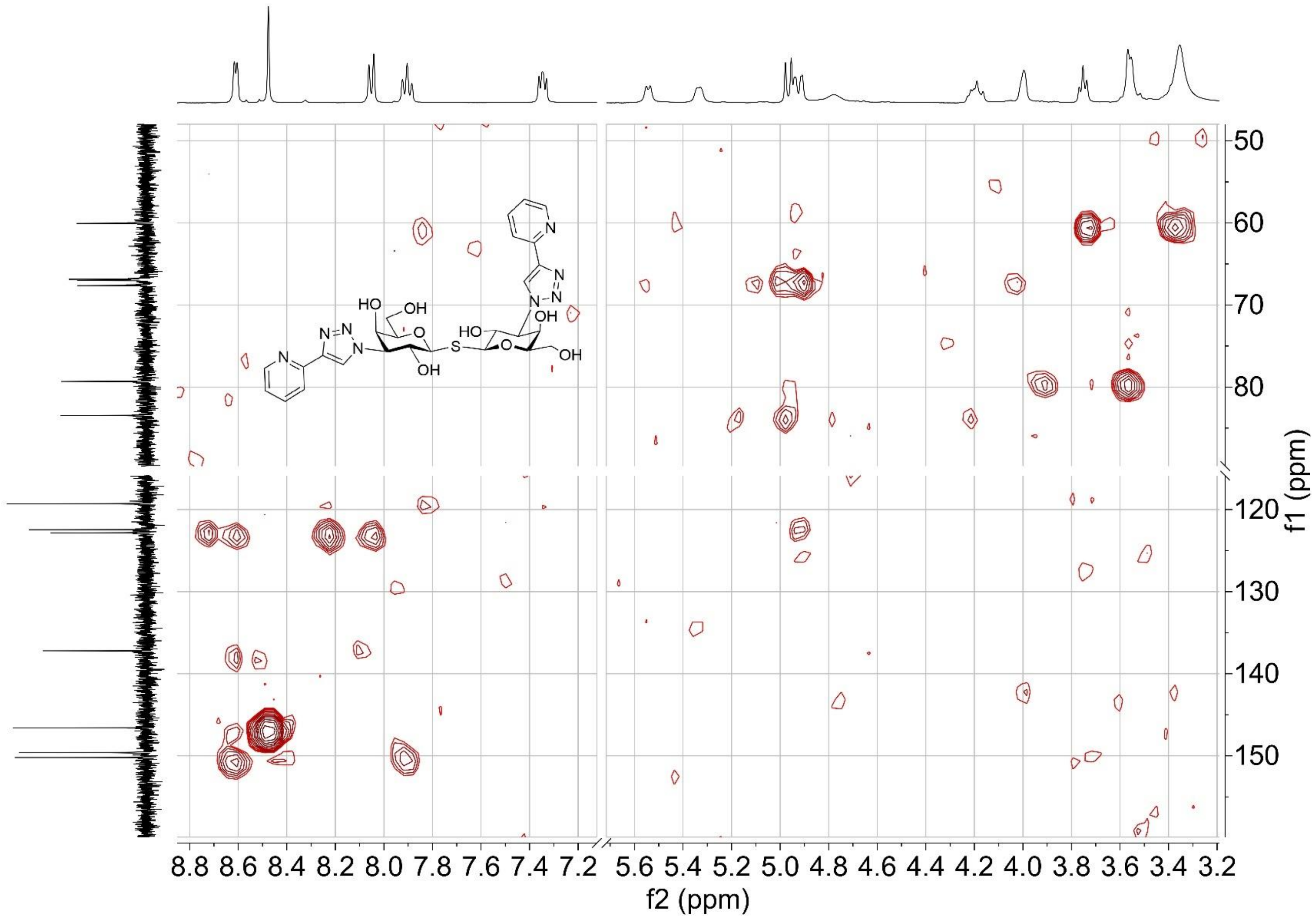

NMR COMPOUND S3

^{1}H NMR (400 MHz, CD_3OD) S3

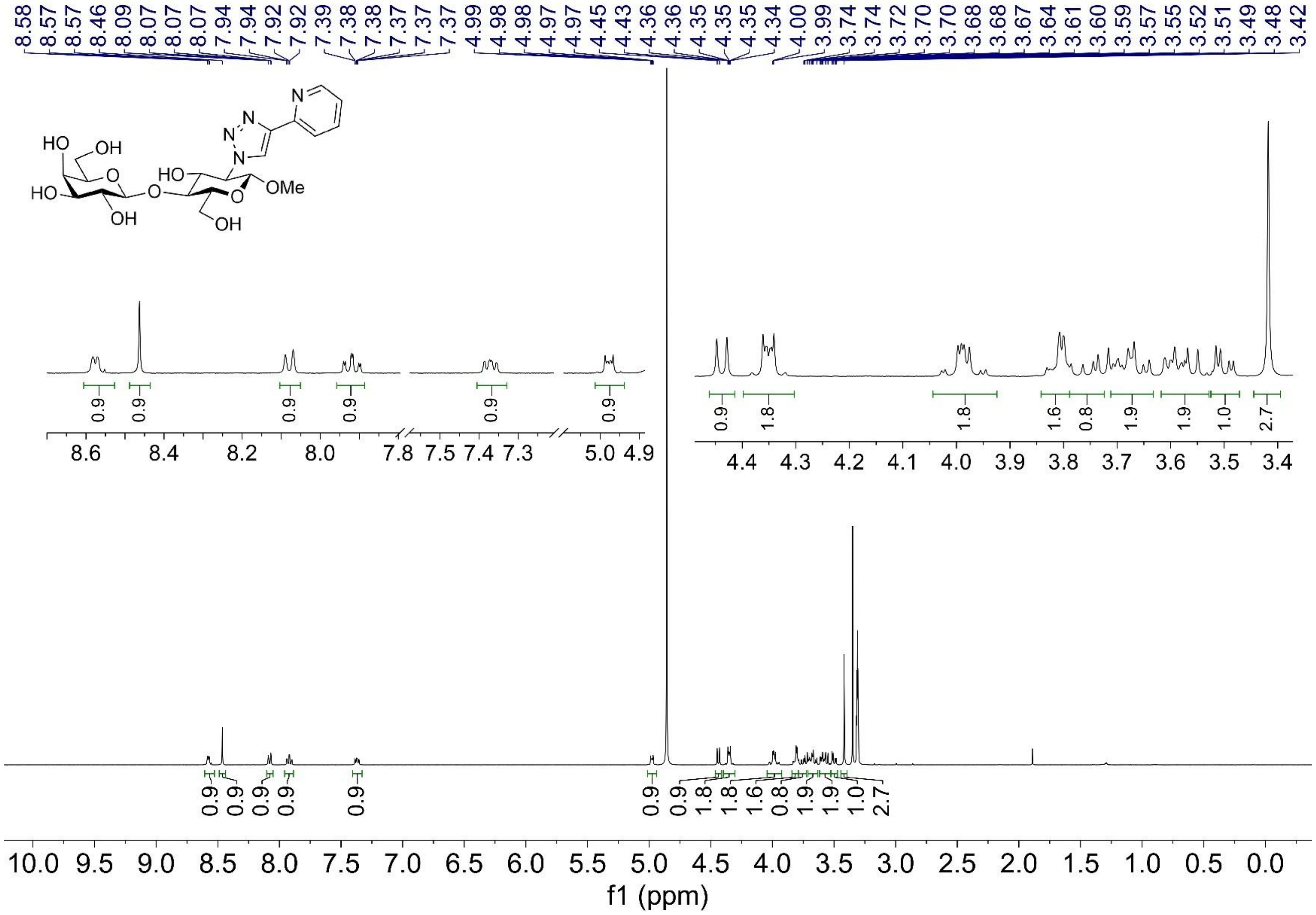

^{13}C NMR (101 MHz, CD_3OD) S3

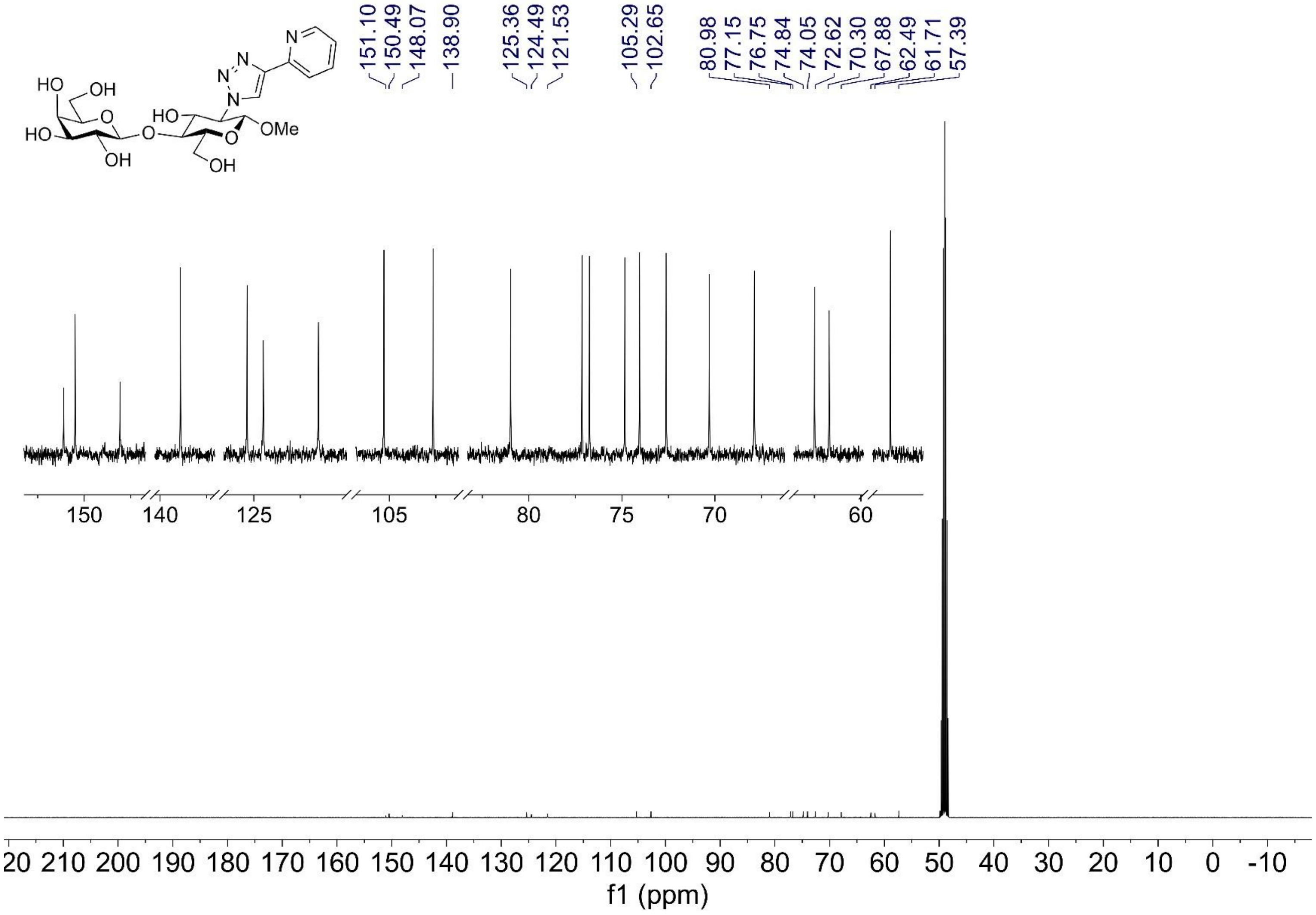

^{1}H-^{1}H COSY S3

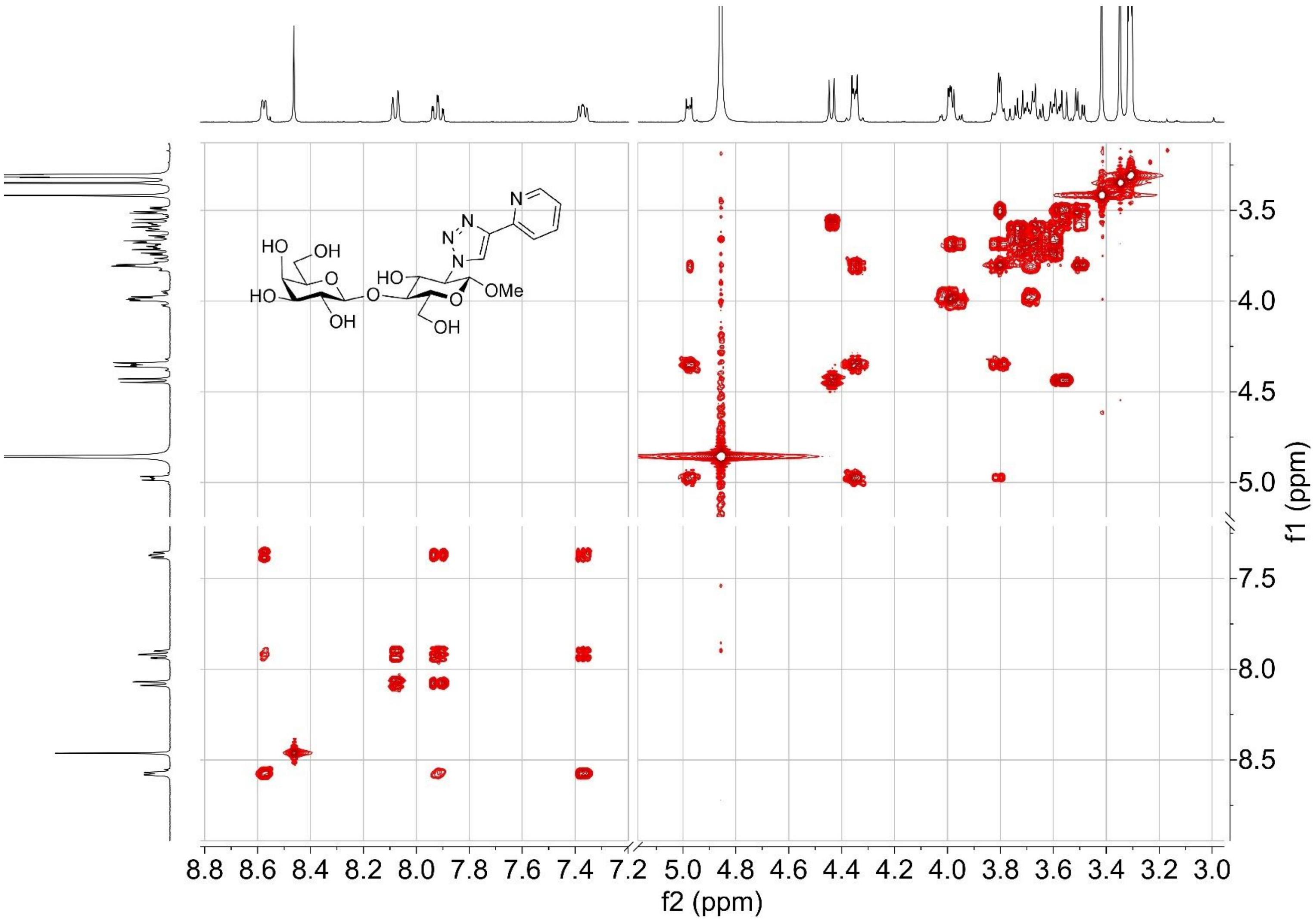
HO
OH
HO
HO
O
O
OH
HO
N
N
N
N
O
OMe
OH
3.5
4.0
4.5
5.0
7.5
8.0
8.5
f1 (ppm)
8.8 8.6 8.4 8.2 8.0 7.8 7.6 7.4 7.2 5.0 4.8 4.6 4.4 4.2 4.0 3.8 3.6 3.4 3.2 3.0
f2 (ppm)

^{1}H-^{13}C HSQC S3

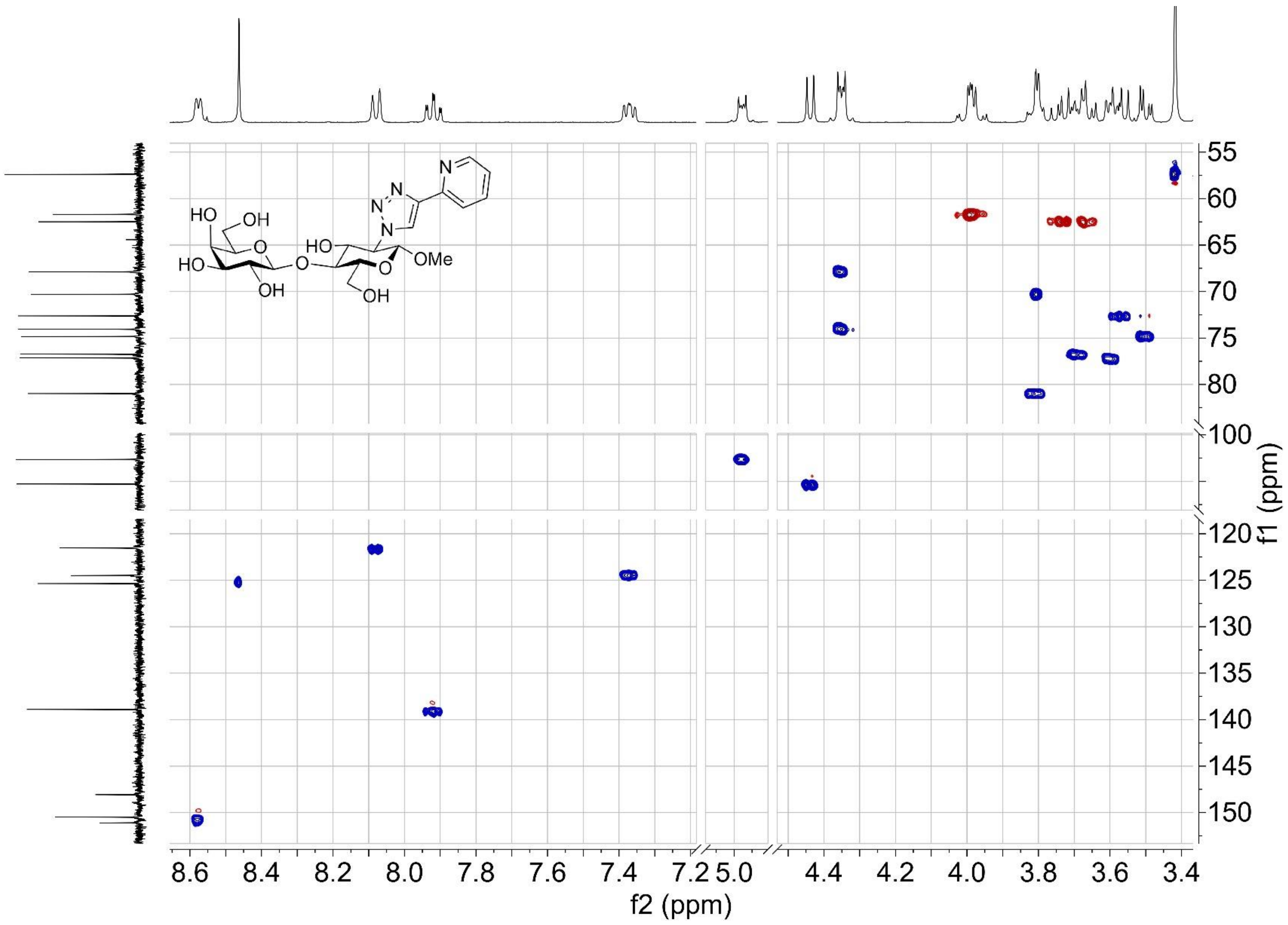

^{1}H-^{13}C HMBC S3

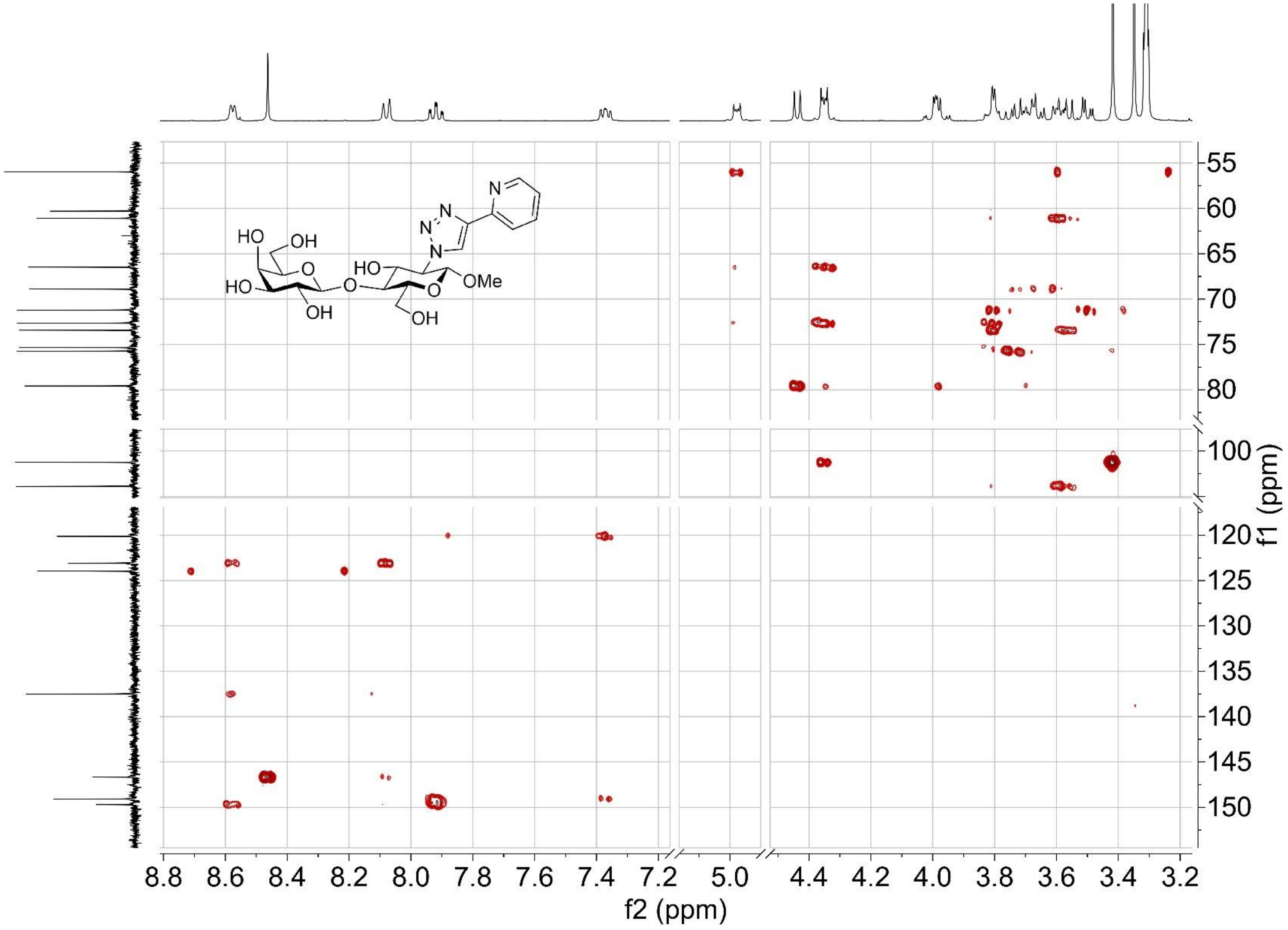

NMR COMPOUND S5

^{1}H NMR (400 MHz, $CDCl_3$) S5

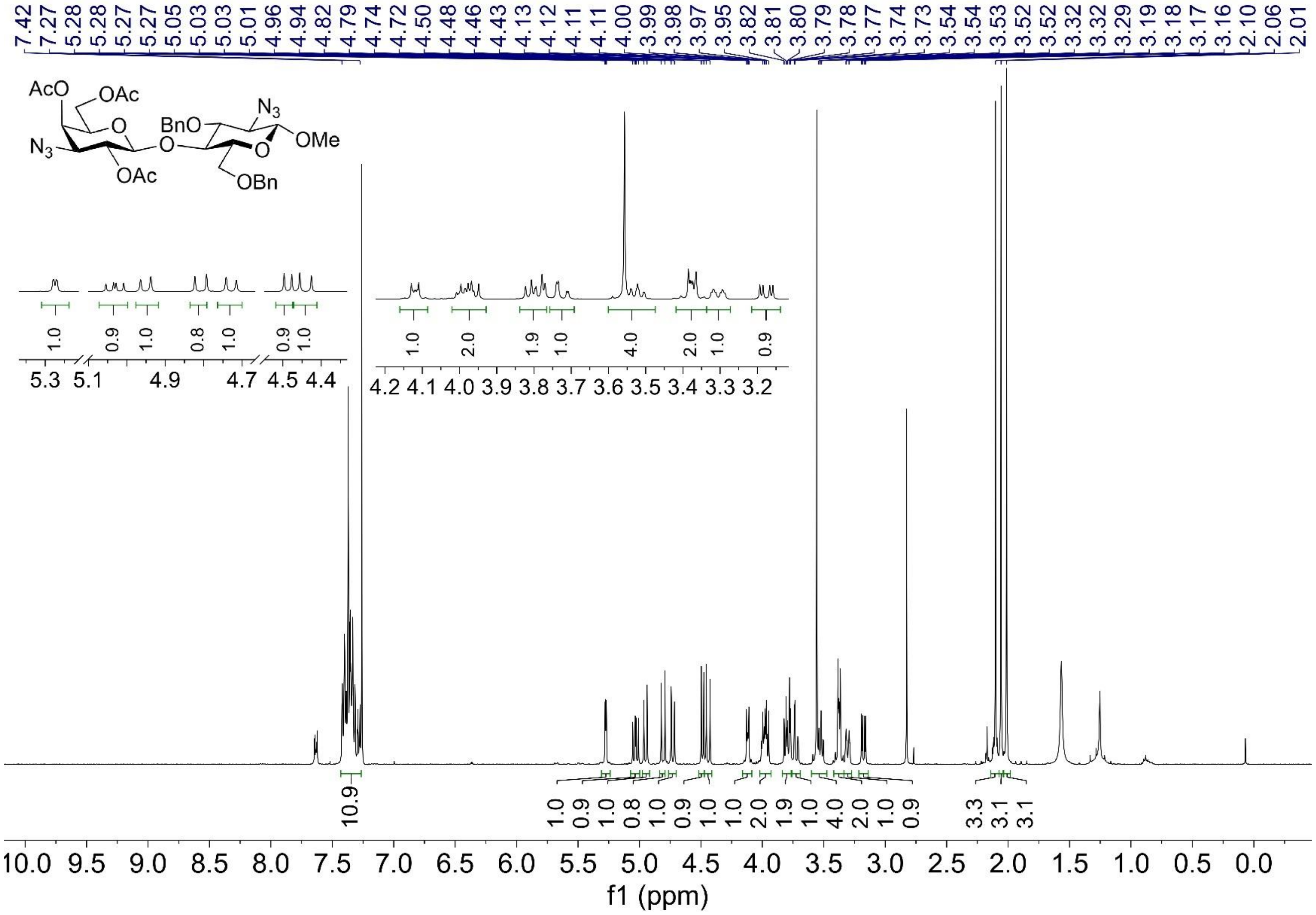

^{13}C NMR (101 MHz, CDCl$_3$) S5

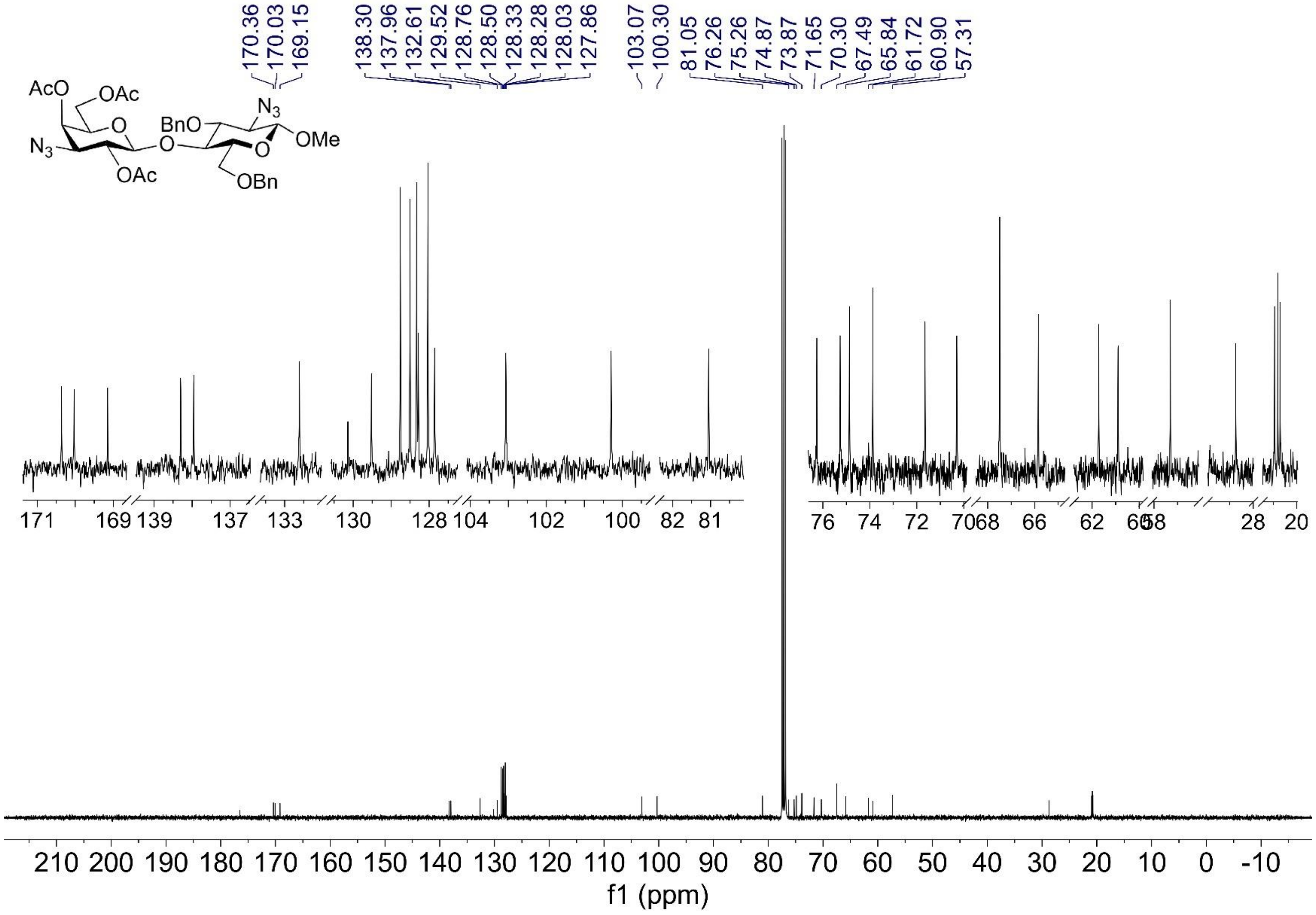

^{1}H-^{1}H COSY S5

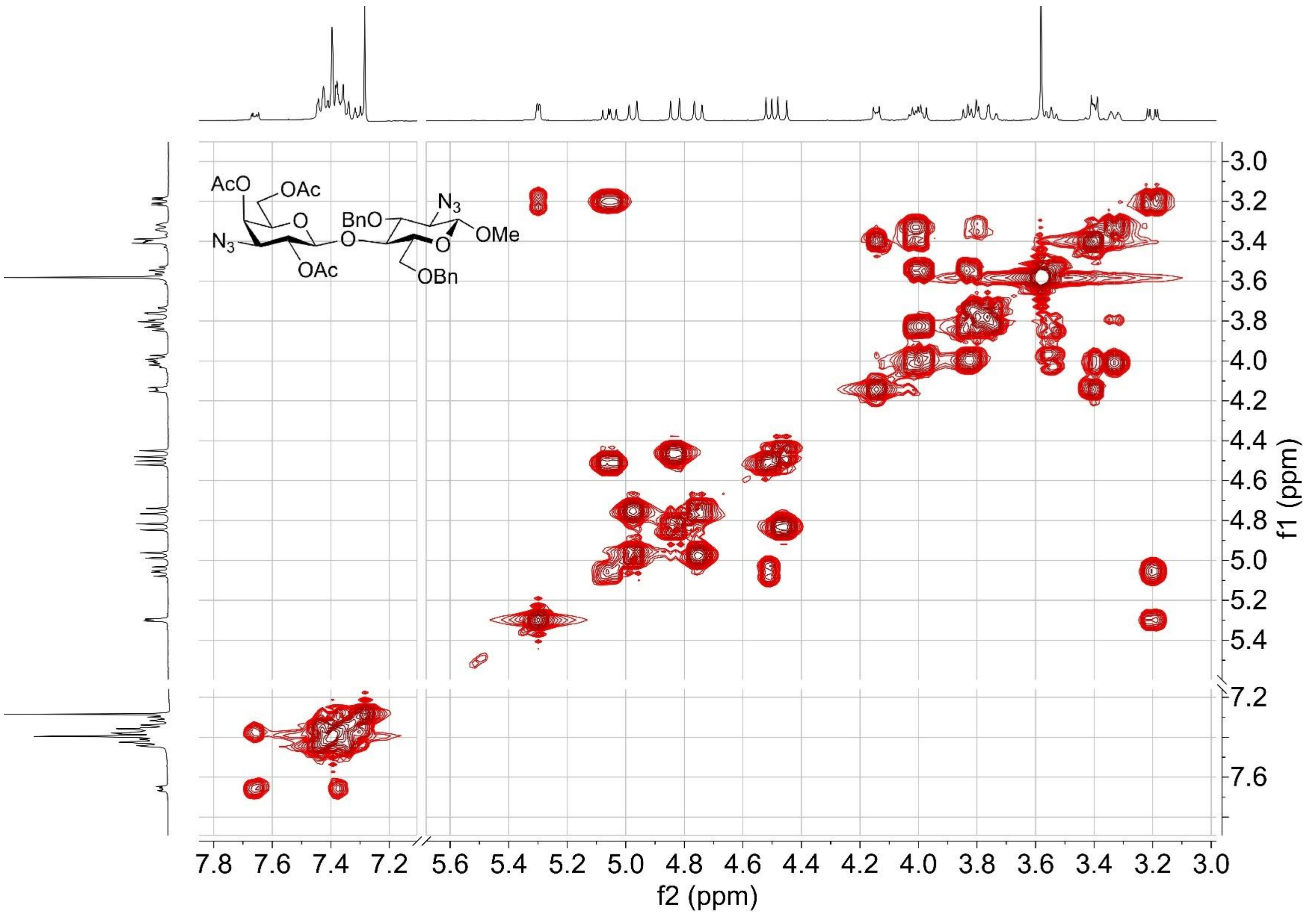

^{1}H-^{13}C HSQC S5

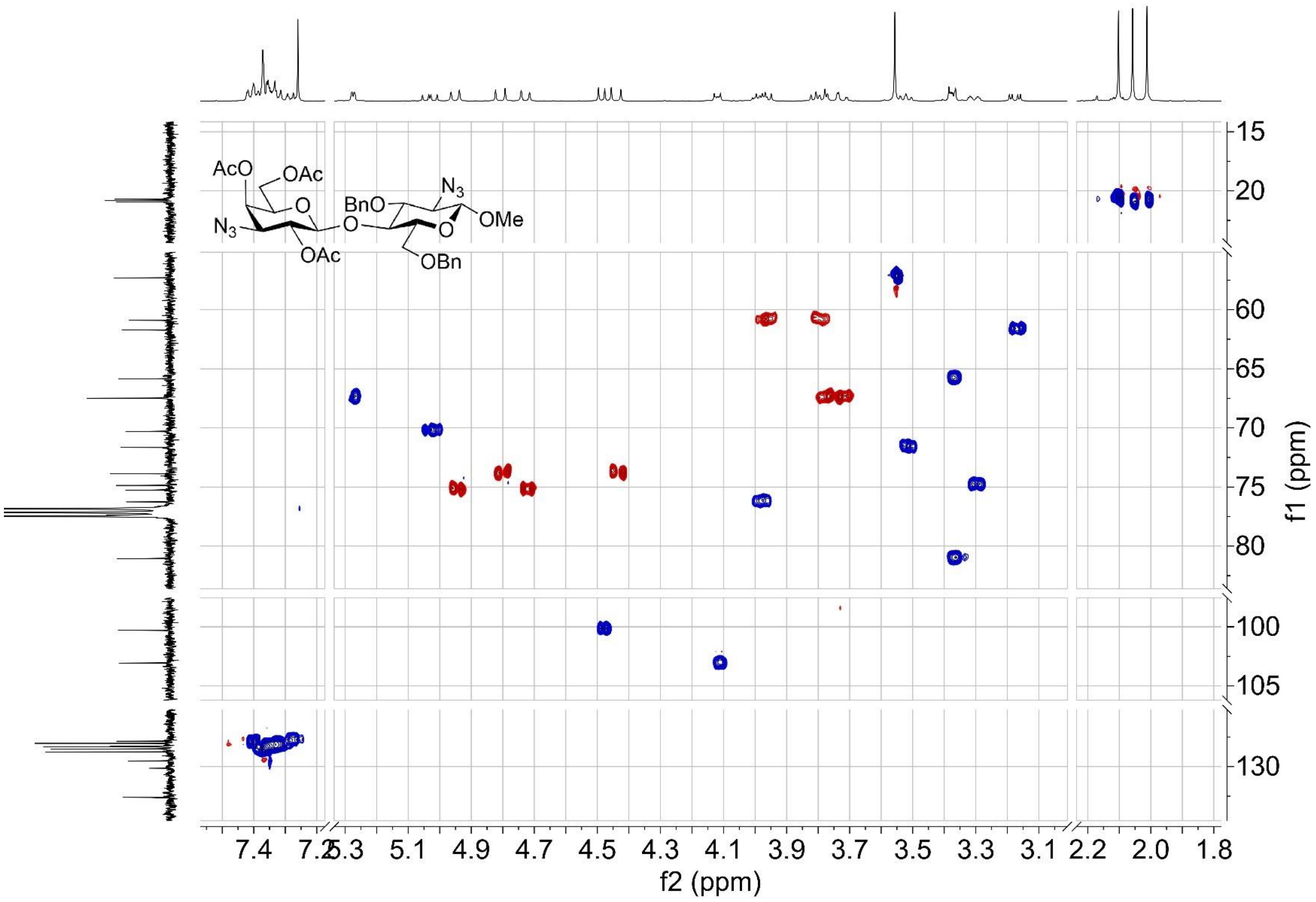

^{1}H-^{13}C HMBC S5

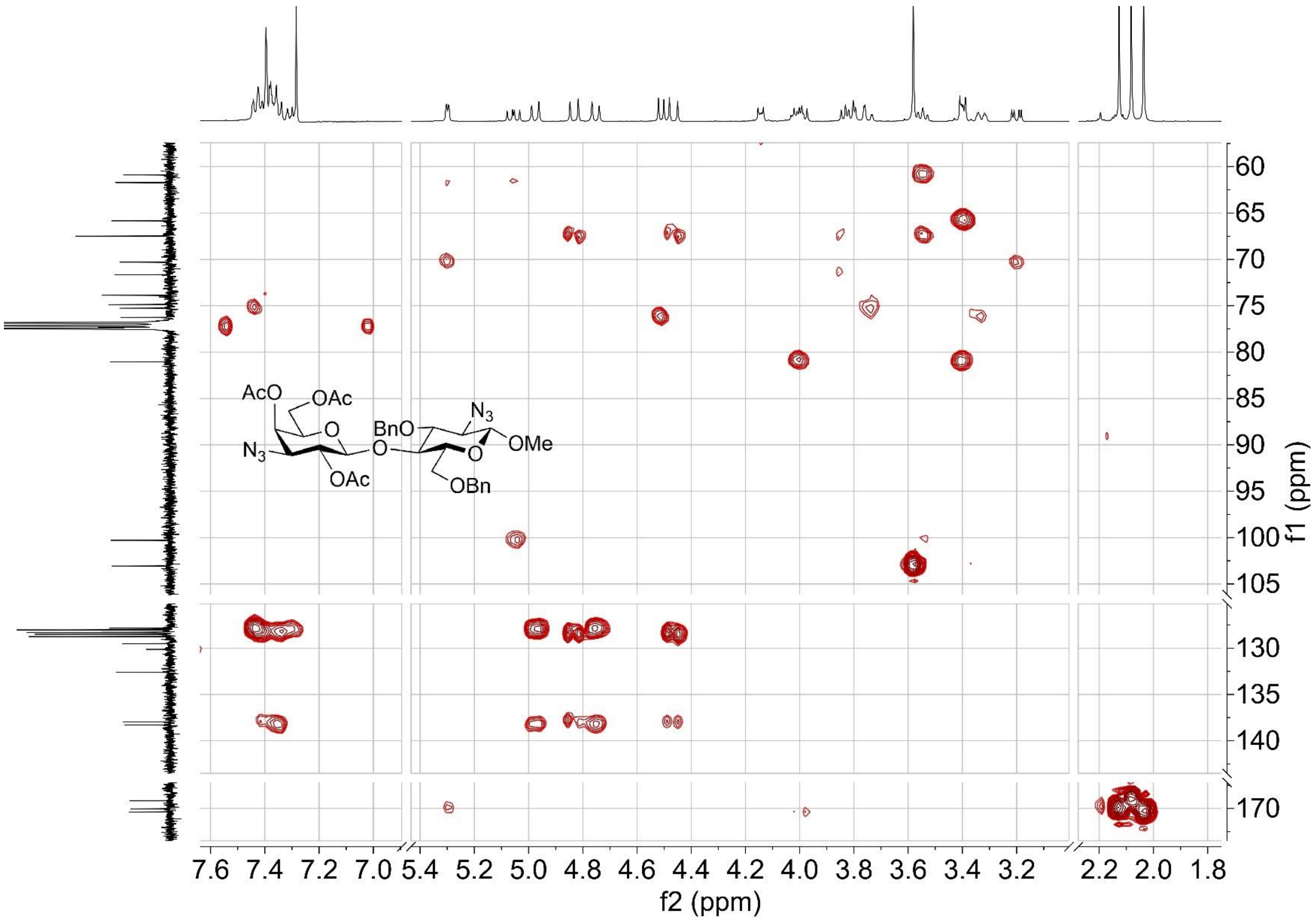

NMR COMPOUND S6

^{1}H NMR (400 MHz, $CDCl_3$) S6

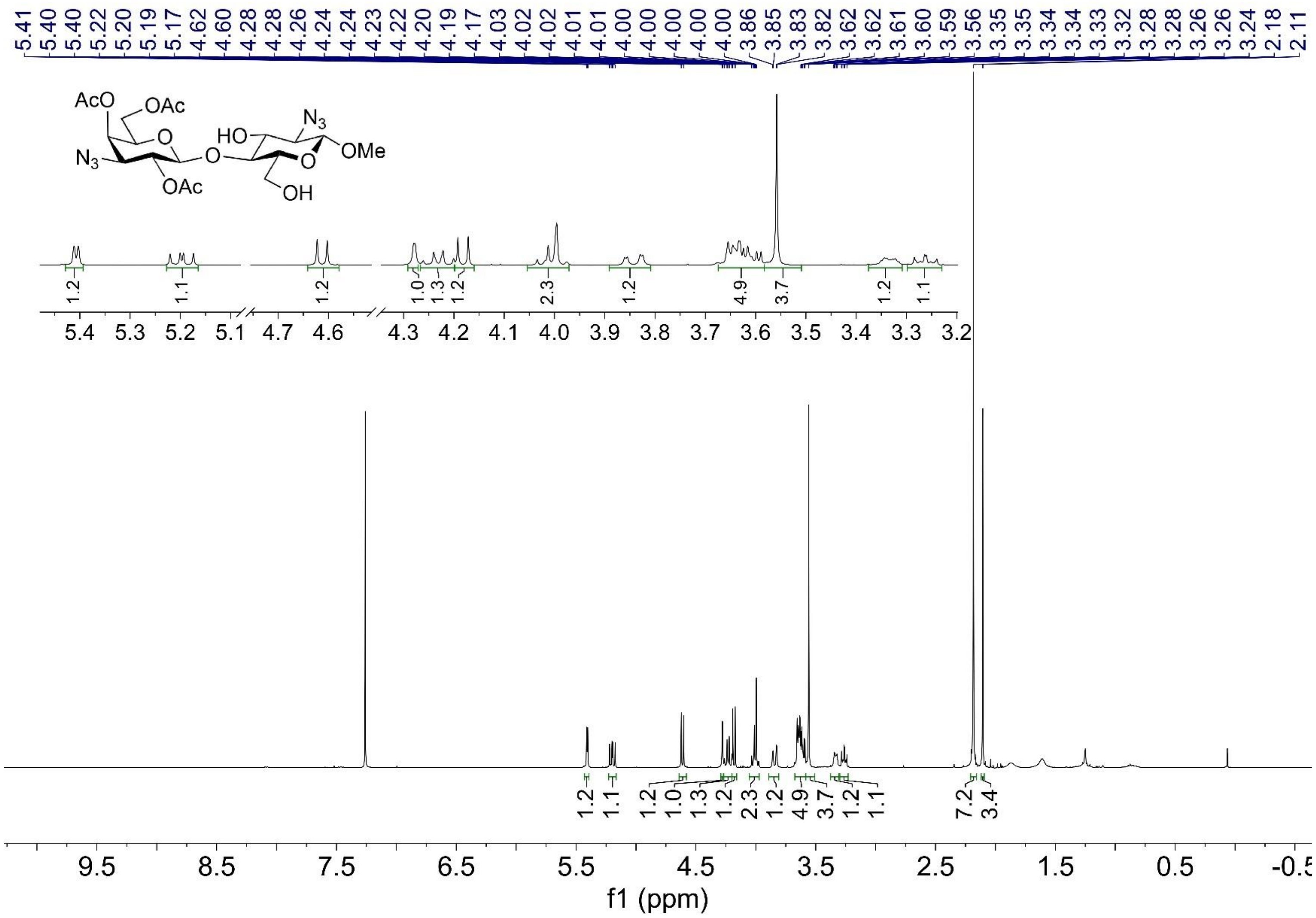

^{13}C NMR (101 MHz, CDCl$_3$) S6

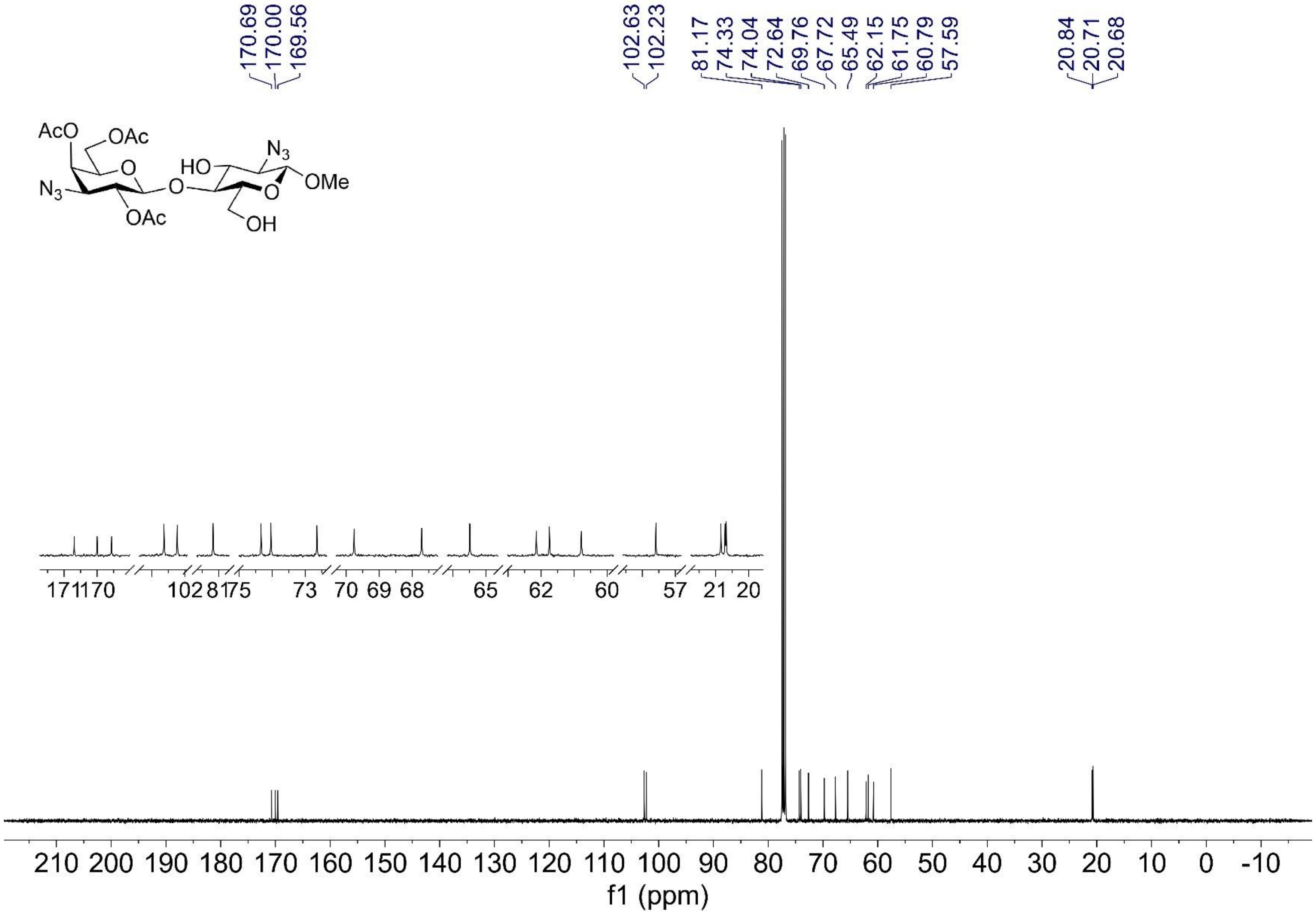

^{1}H-^{1}H COSY S6

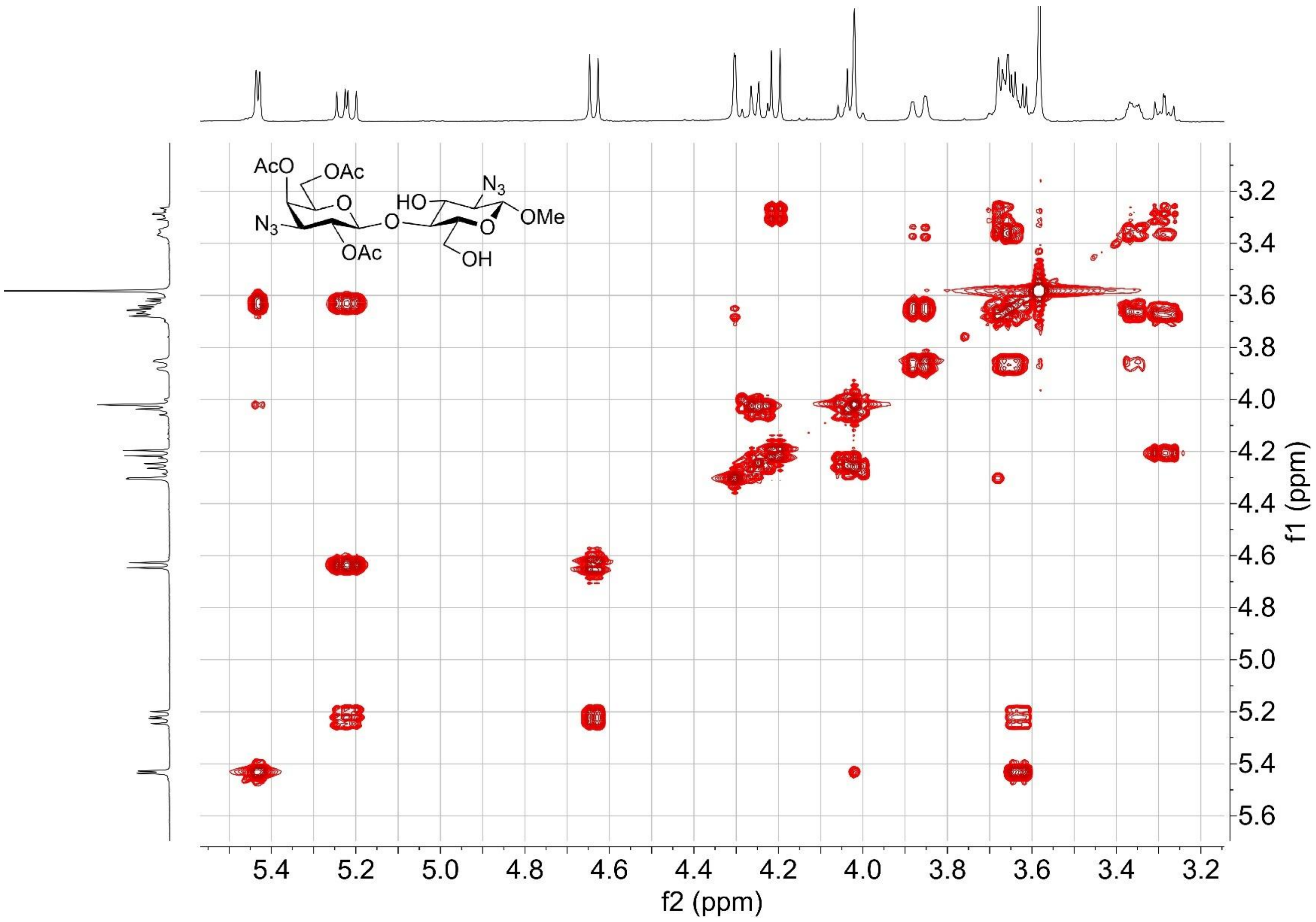

^{1}H-^{13}C HSQC S6

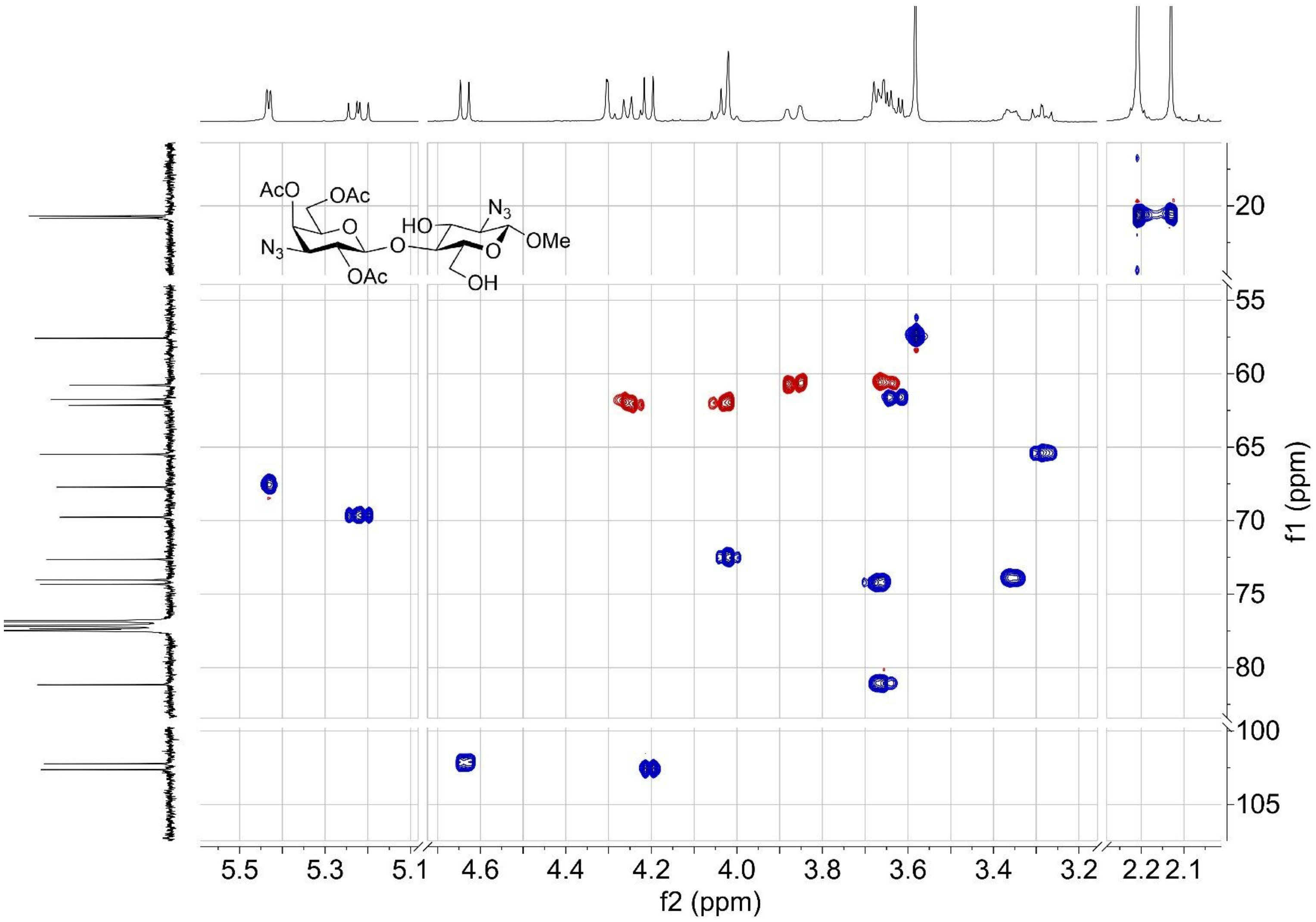

^{1}H-^{13}C HMBC S6

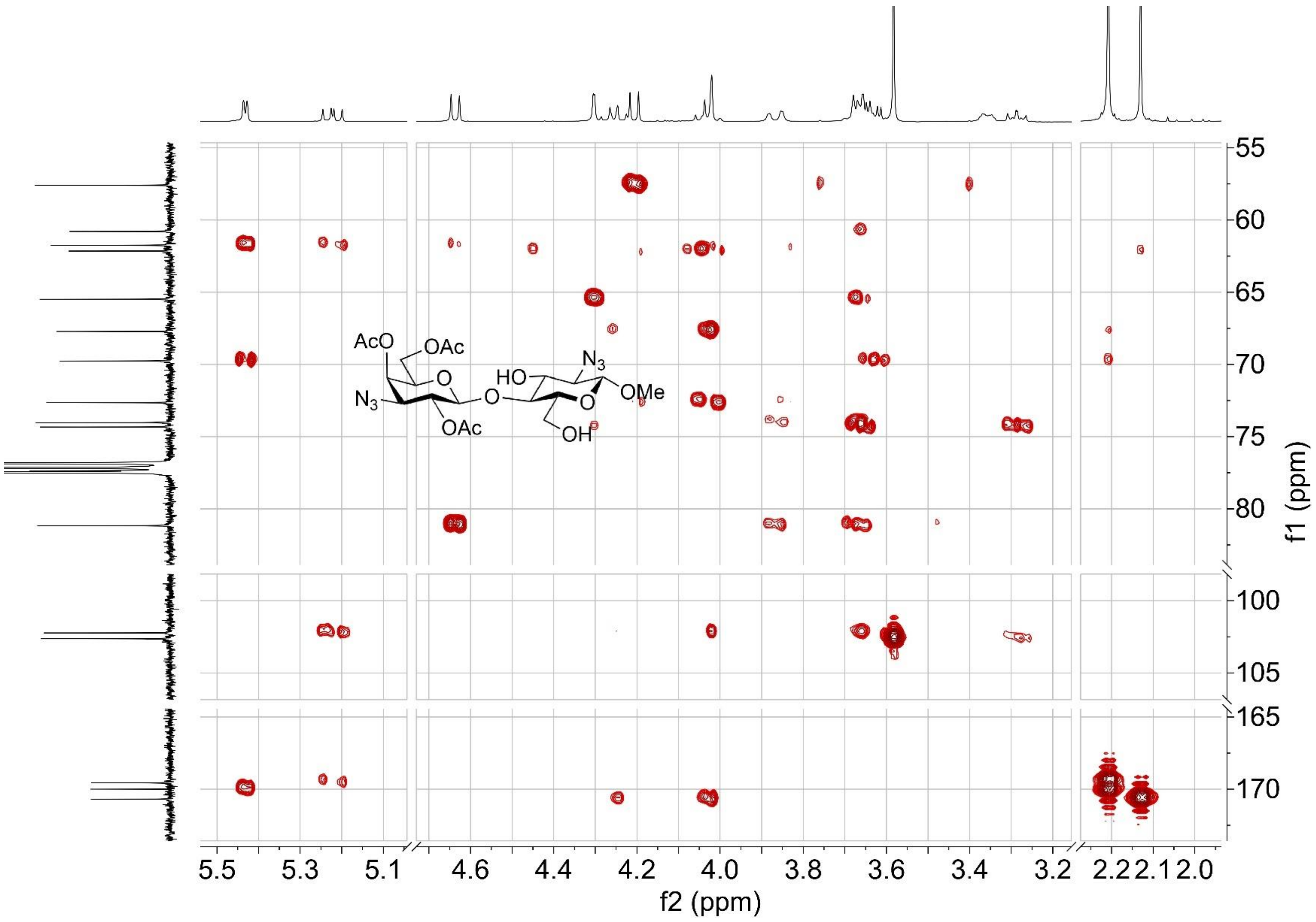

Stability of 15-PF in physiological solution

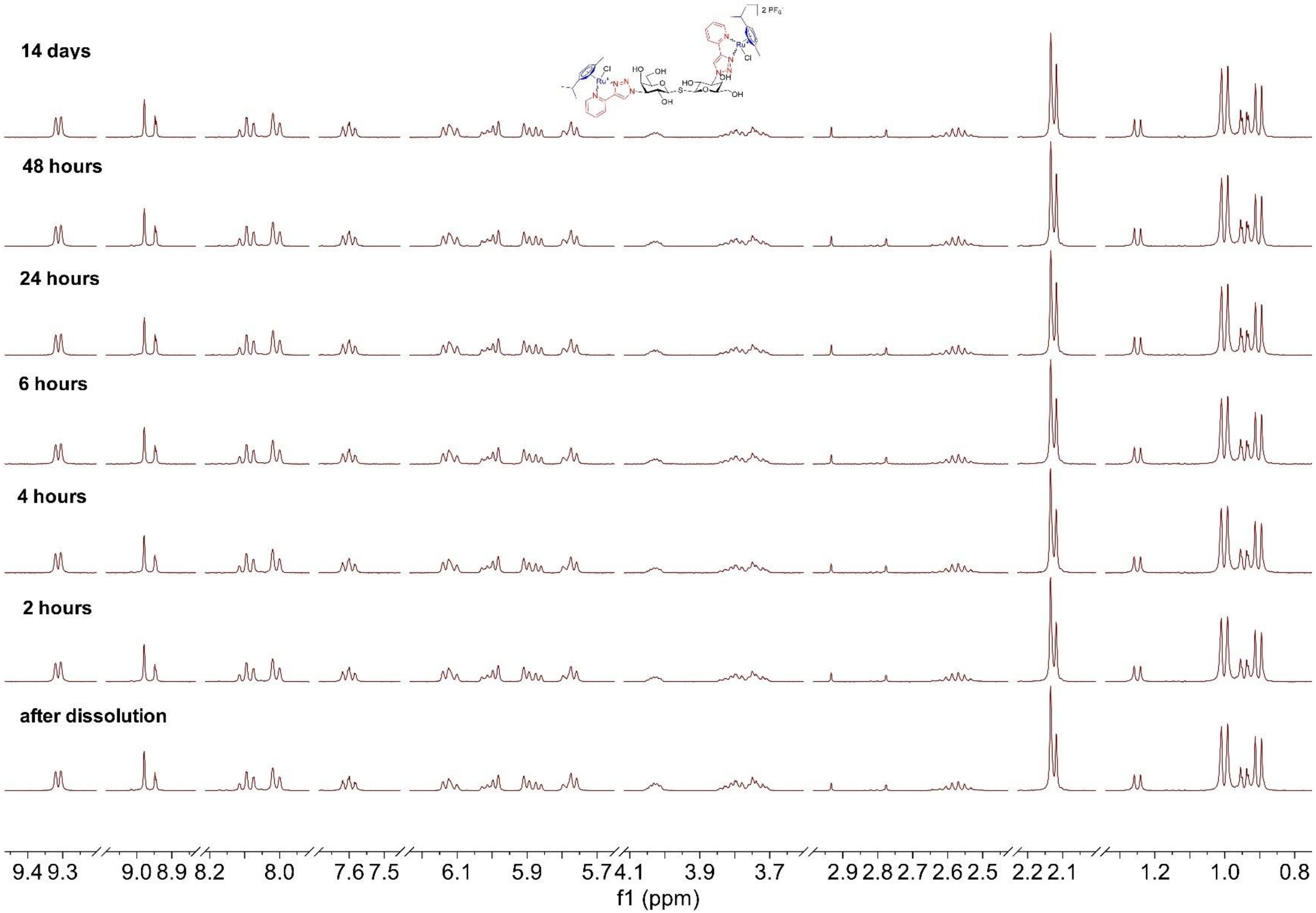

Stability of 15-PF in PBS buffer

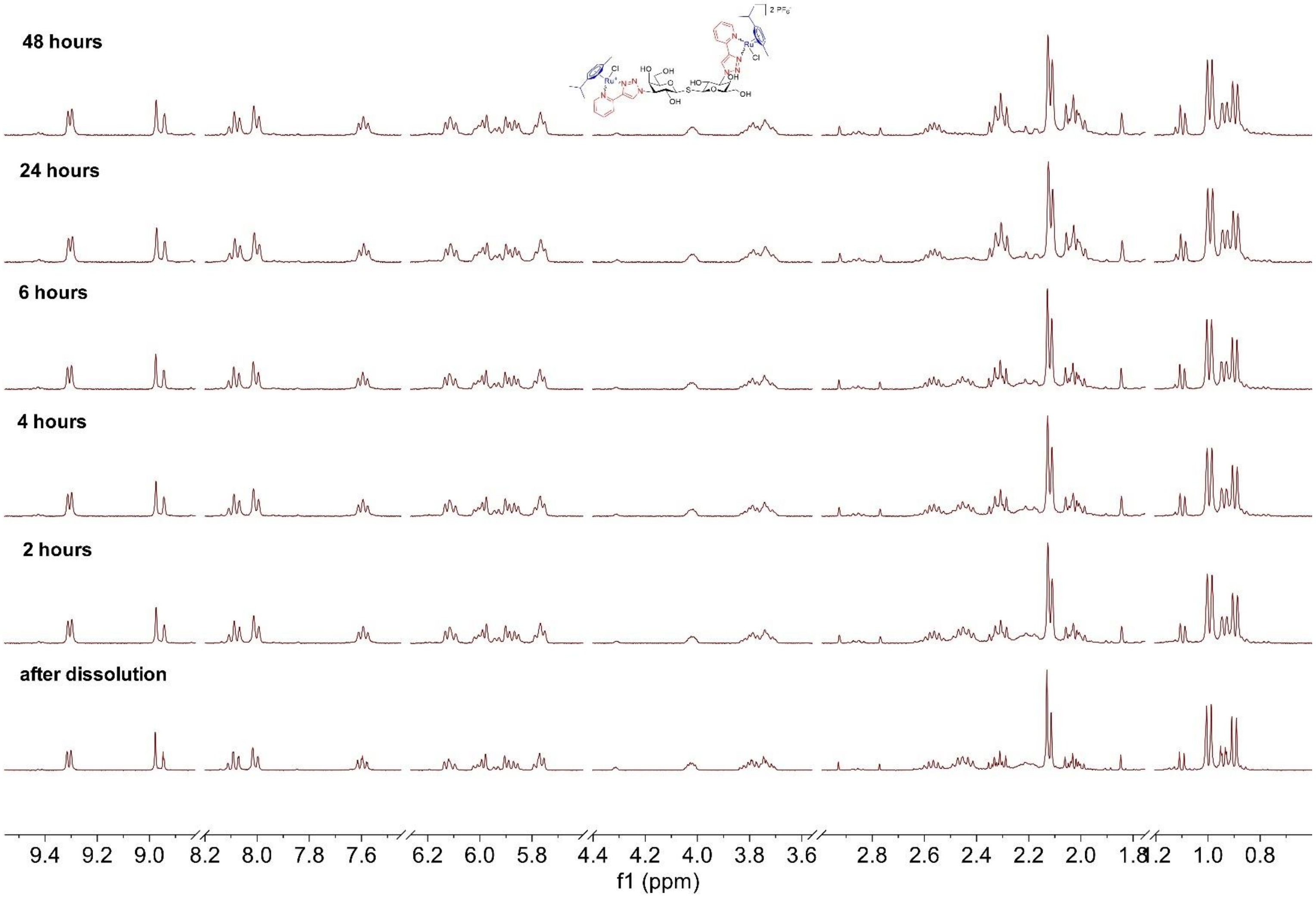

Stability of 15-PF in blood serum

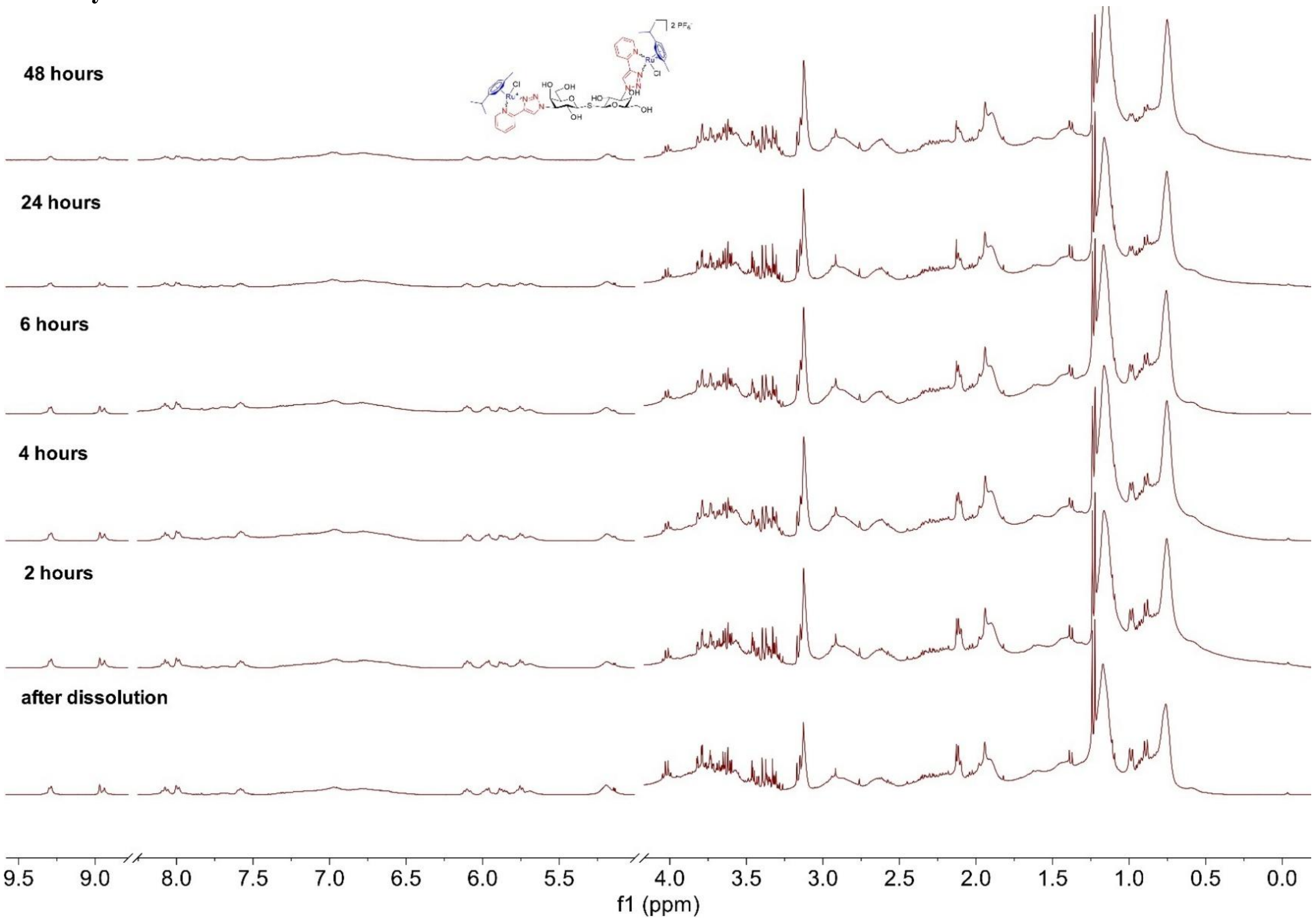

I. HPLC traces

Purity of the synthesized most potent inhibitors **14-Cl**, **15-Cl**, **15-BF**, **15-PF** and **16-I** was assessed by analytical HPLC using a Phenomenex Luna 5 µ C18(2) 100 Å column (150 × 4.6 mm, 5 µm particle size) maintained at 25 °C. Samples (5 µL injection volume) were eluted with a gradient of acetonitrile in water containing formic acid: 0–2 min, 10% MeCN; 2–15 min, linear gradient to 90% MeCN; 15–18 min, 90% MeCN; 18–20 min, return to initial conditions. UV detection was performed at 275 nm. High-resolution mass spectra were recorded using ESI+ ionization.

HPLC trace 14-Cl

UV 275 nm detection:

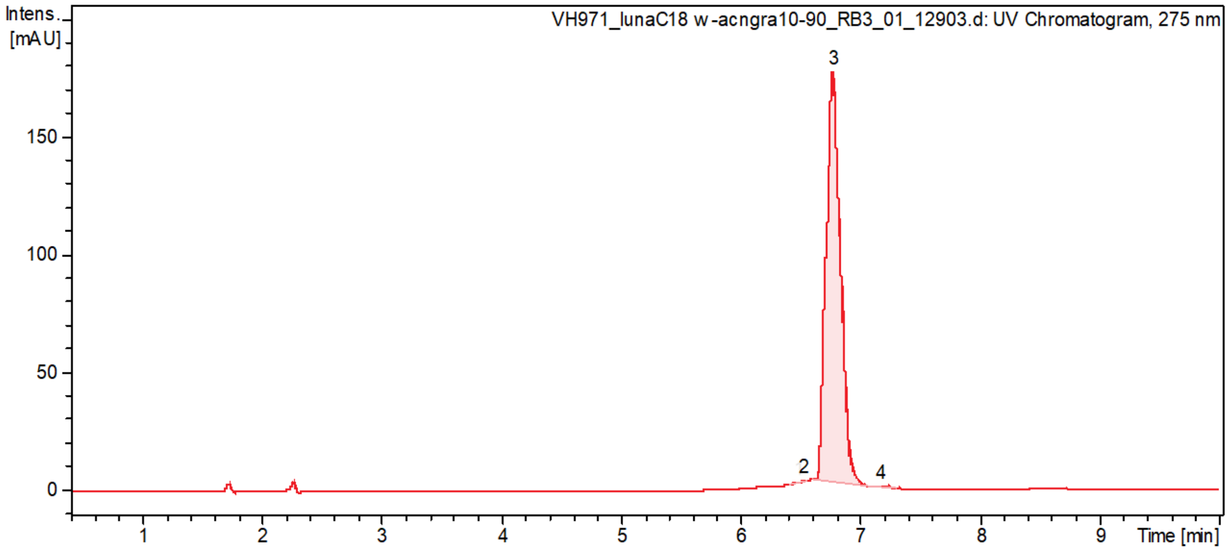

#	RT [min]	Area Frac. % (UV 275nm)
1	6.5	0.04
2	6.5	0.13
3	6.8	99.56
4	7.2	0.27

HRMS m/z 577 detection:

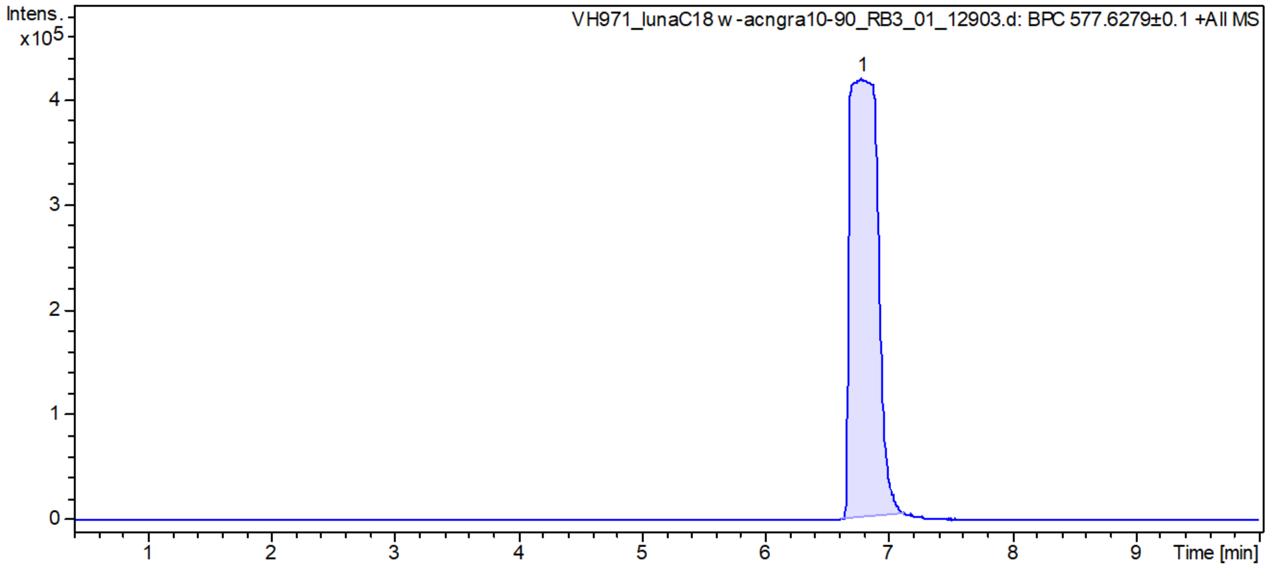

#	RT [min]	Max. m/z
1	6.8	577.1260

HPLC trace 15-Cl

UV 275 nm detection:

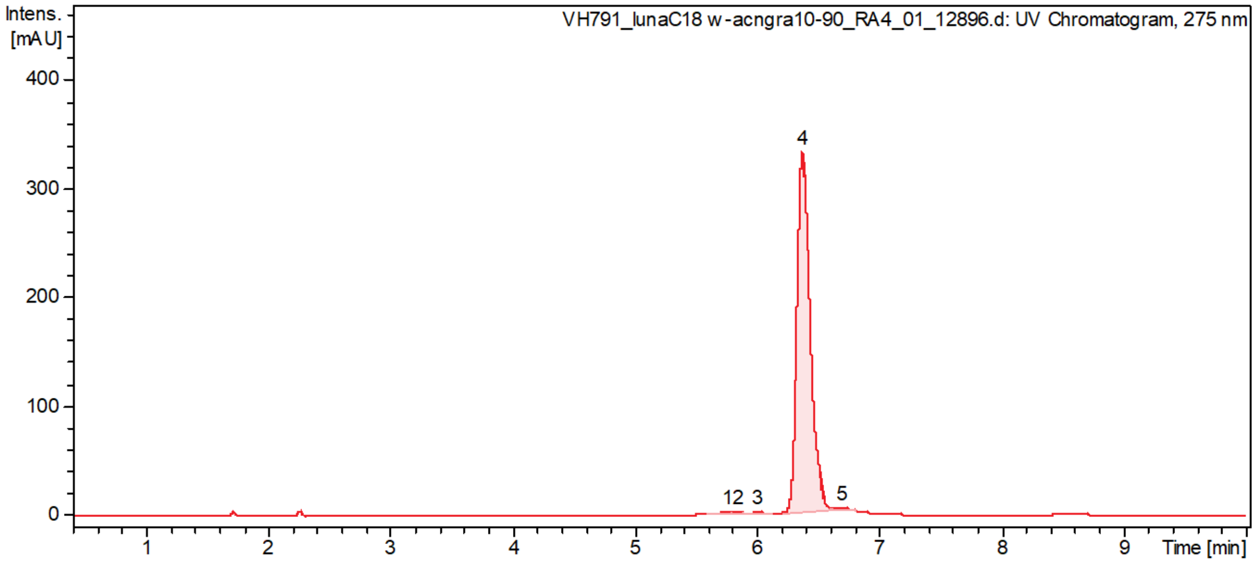

#	RT [min]	Area Frac. %	(UV 275nm)
1	5.8	0.13	
2	5.8	0.08	
3	6.0	0.12	
4	6.4	98.86	
5	6.7	0.81	

HRMS m/z 578 detection:

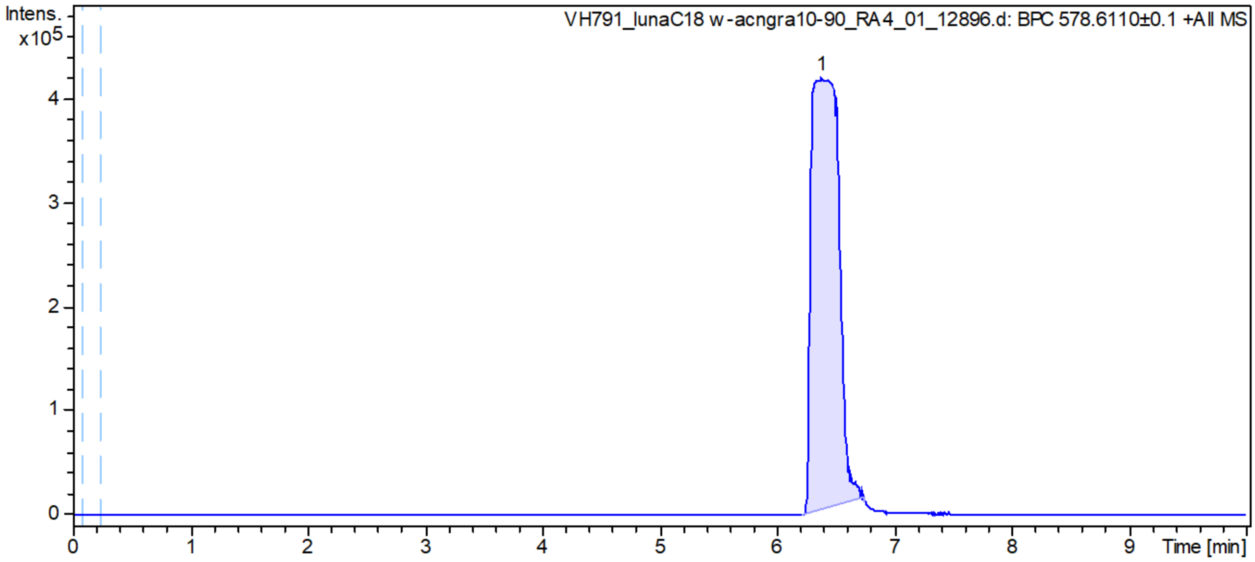

#	RT [min]	Max. m/z
1	6.4	578.1096

HPLC trace 15-BF

UV 275 nm detection:

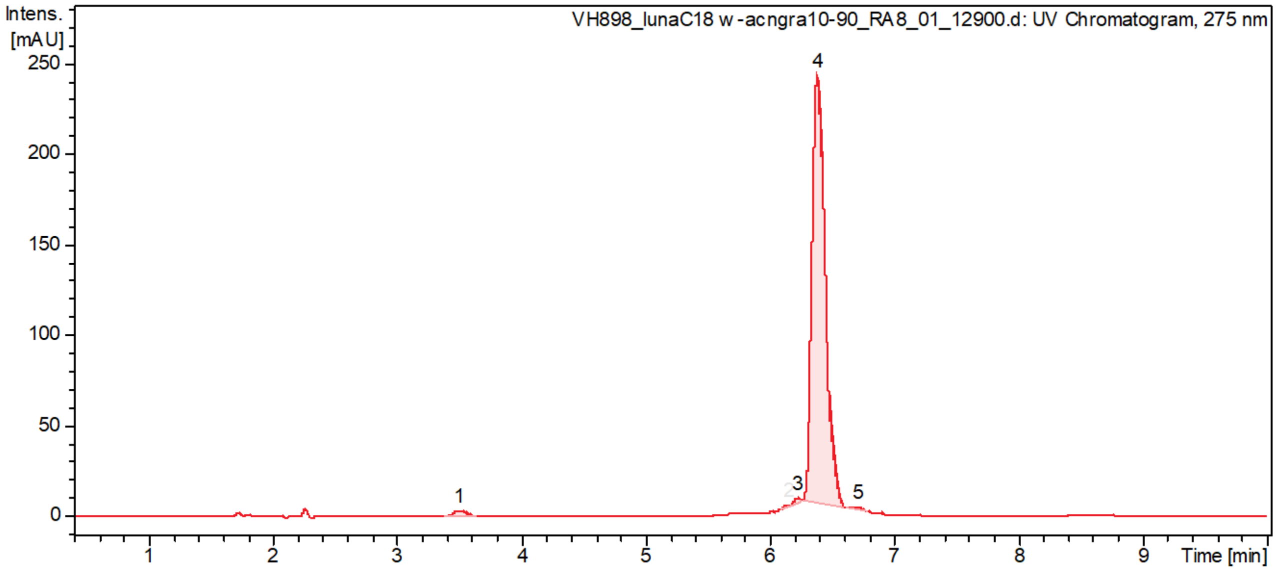

#	RT [min]	Area Frac. % (UV 275nm)
1	3.5	1.32
2	6.1	0.16
3	6.2	0.32
4	6.4	97.85
5	6.7	0.36

HRMS m/z 577 detection:

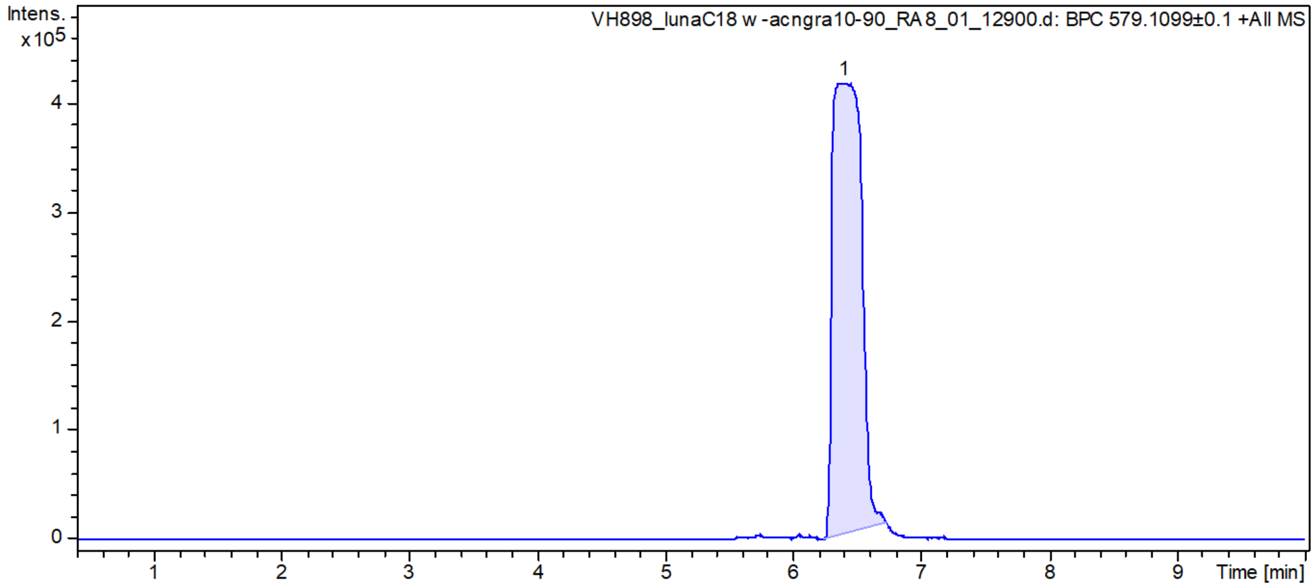

#	RT [min]	Max. m/z
1	6.4	578.1094

HPLC trace 15-PF

UV 275 nm detection:

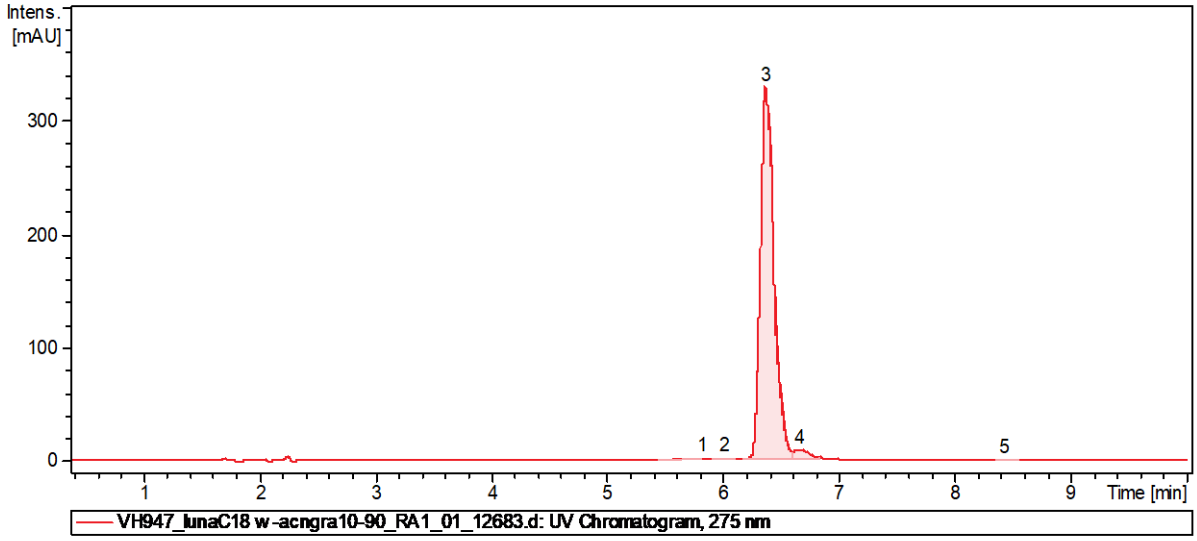

#	RT [min]	Area Frac. %
1	5.8	0.18
2	6.0	0.10
3	6.4	97.36
4	6.7	2.31
5	8.4	0.05

HRMS m/z 577 detection:

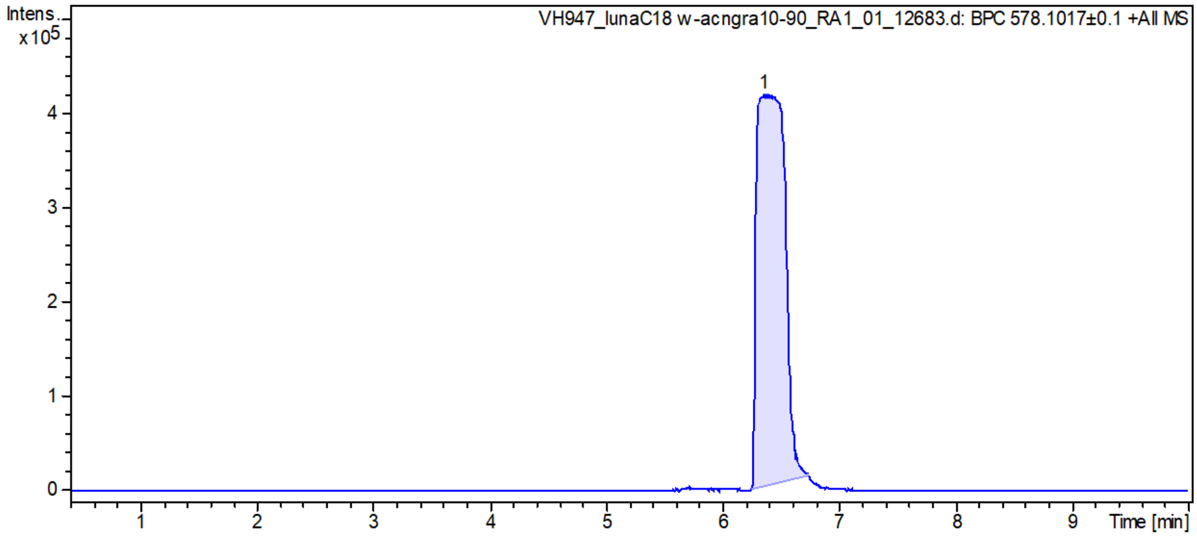

#	RT [min]	Max. m/z
1	6.4	578.1026

HPLC trace 16-I

UV 275 nm detection:

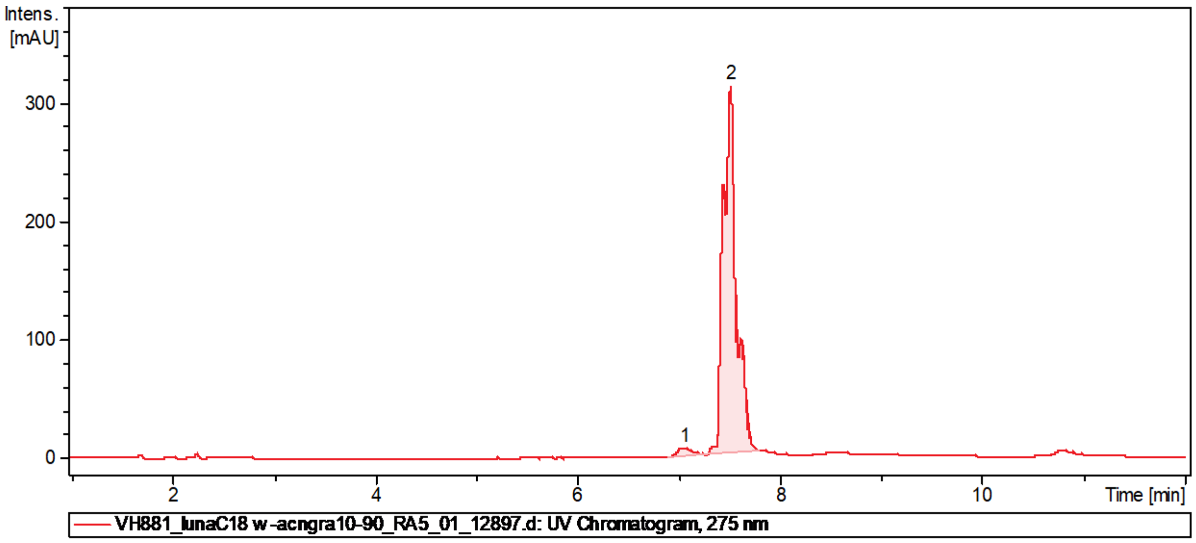

#	RT [min]	Area Frac. %
1	7.1	2.30
2	7.5	97.70

HRMS m/z 577 detection:

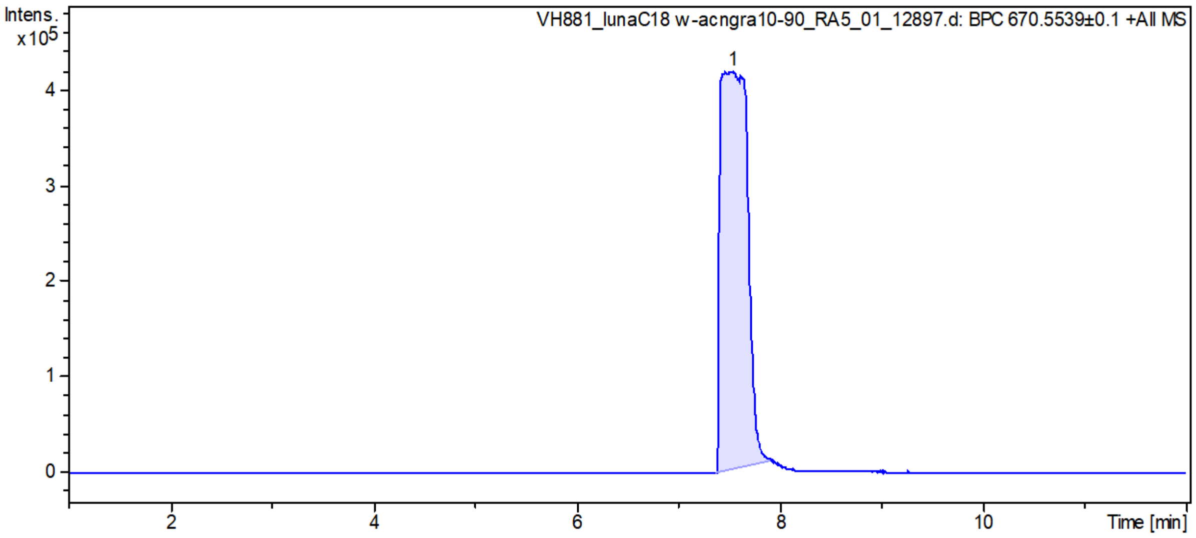

#	RT [min]	Max. m/z
1	7.5	670.0499

J. References

(1) Futera, Z.; Klenko, J.; Šponer, J. E.; Šponer, J.; Burda, J. V. Interactions of the "piano-stool" [ruthenium(II) (η^6-arene)(en)CL]$^+$ complexes with water and nucleobases; ab initio and DFT study. *J. Comput. Chem.* **2009**, *30*, 1758–1770. https://doi.org/10.1002/jcc.21179

(2) Kacsir, I.; Sipos, A.; Ujlaki, G.; Buglyó, P.; Somsák, L.; Bai, P.; Bokor, É. Ruthenium Half-Sandwich Type Complexes with Bidentate Monosaccharide Ligands Show Antineoplastic Activity in Ovarian Cancer Cell Models through Reactive Oxygen Species Production. *Int. J. Mol. Sci.* **2021**, *22*, 10454. https://doi.org/10.3390/ijms221910454

(3) Wang, F.; Habtemariam, A.; van der Geer, E. P. L.; Fernández, R.; Melchart, M.; Deeth, R. J.; Aird, R.; Guichard, S.; Fabbiani, F. P. A.; Lozano-Casal, P.; Oswald, I. D. H.; Jodrell, D. I.; Parsons, S.; Sadler, P. J. Controlling ligand substitution reactions of organometallic complexes: Tuning cancer cell cytotoxicity. *PNAS* **2005**, *102*, 18269–18274. https://doi.org/10.1073/pnas.0505798102

(4) Tropea, J. E.; Cherry, S.; Waugh, D. S. Expression and purification of soluble His 6-tagged TEV protease. In *High Throughput Protein Expression and Purification: Methods and Protocols*, Doyle, S. A. Ed.; Humana Press, **2009**, 297–307. https://doi.org/10.1007/978-1-59745-196-3_19

(5) Pace, K. E.; Hahn, H. P.; Baum, L. G. Preparation of recombinant human galectin-1 and use in T-cell death assays. In *Methods in enzymology*, Vol. 363; Academic Press, **2003**, 499–518.

(6) Hovorková, M.; Červený, J.; Bumba, L.; Pelantová, H.; Cvačka, J.; Křen, V.; Renaudet, O.; Goyard, D.; Bojarová, P. Advanced high-affinity glycoconjugate ligands of galectins. *Bioorg. Chem.* **2023**, *131*, 106279. https://doi.org/10.1016/j.bioorg.2022.106279

(7) Vrbata, D.; Červený, J.; Kulik, N.; Hovorková, M.; Balogová, S.; Vlachová, M.; Pelantová, H.; Křen, V.; Bojarová, P. Glycomimetic inhibitors of tandem-repeat galectins: Simple and efficient. *Bioorg. Chem.* **2024**, *145*, 107231. https://doi.org/10.1016/j.bioorg.2024.107231

(8) Hamala, V.; Kurfiřt, M.; Červenková Šťastná, L.; Hujerová, H.; Bernášková, J.; Parkan, K.; Kaminský, J.; Habanová, N.; Kozák, J.; Magdolenová, A.; Zavřel, M.; Staroňová, T.; Ostatná, V.; Žaloudková, L.; Daňhel, A.; Holčáková, J.; Voňka, P.; Hrstka, R.; Karban, J. Ferrocene- and ruthenium arene-containing glycomimetics as selective inhibitors of human galectin-1 and -3. *Inorg. Chem. Front.* 2024, *11*, 7588–7609. https://doi.org/10.1039/D4QI01555J

(9) Sörme, P.; Kahl-Knutsson, B.; Huflejt, M.; Nilsson, U. J.; Leffler, H. Fluorescence polarization as an analytical tool to evaluate galectin–ligand interactions. *Anal. Biochem.* **2004**, *334*, 36–47. https://doi.org/10.1016/j.ab.2004.06.042

(10) Peterson, K.; Kumar, R.; Stenström, O.; Verma, P.; Verma, P. R.; Håkansson, M.; Kahl-Knutsson, B.; Zetterberg, F.; Leffler, H.; Akke, M.; Logan, D. T.; Nilsson, U. J. Systematic Tuning of Fluoro-galectin-3 Interactions Provides Thiodigalactoside Derivatives with Single-Digit nM Affinity and High Selectivity. *J. Med. Chem.* **2018**, *61*, 1164–1175. https://doi.org/10.1021/acs.jmedchem.7b01626

(11) Nikolovska-Coleska, Z.; Wang, R.; Fang, X.; Pan, H.; Tomita, Y.; Li, P.; Roller, P. P.; Krajewski, K.; Saito, N. G.; Stuckey, J. A.; Wang, S. Development and optimization of a binding assay for the XIAP BIR3 domain using fluorescence polarization. *Anal. Biochem.* **2004**, *332*, 261–273. https://doi.org/10.1016/j.ab.2004.05.055

(12) Hamala, V.; Kurfiřt, M.; Šťastná, L. Č.; Hujerová, H.; Bernášková, J.; Parkan, K.; Kaminský, J.; Habanová, N.; Kozák, J.; Magdolenová, A. Ferrocene-and ruthenium arene-containing glycomimetics as selective inhibitors of human galectin-1 and-3. *Inorg. Chem. Front.* **2024**, *11*, 7588–7609. https://doi.org/10.1039/D4QI01555J

(13) Sindrewicz, P.; Li, X.; Yates, E. A.; Turnbull, J. E.; Lian, L.-Y.; Yu, L.-G. Intrinsic tryptophan fluorescence spectroscopy reliably determines galectin-ligand interactions. *Sci. Rep.* **2019**, *9*, 11851. https://doi.org/10.1038/s41598-019-47658-8

(14) Hodik, T.; Lamac, M.; St'astna, L. C.; Karban, J.; Koubkova, L.; Hrstka, R.; Cisarova, I.; Pinkas, J. Titanocene Dihalides and Ferrocenes Bearing a Pendant α-D-Xylofuranos-5-yl or α-D-Ribofuranos-5-yl

Moiety. Synthesis, Characterization, and Cytotoxic Activity. *Organometallics* **2014**, *33*, 2059–2070. https://doi.org/10.1021/om500200r
